# A wide landscape of morbidity and mortality risk associated with marital status in 0.5 million Chinese men and women: a prospective cohort study

**DOI:** 10.1016/j.lanwpc.2023.100948

**Published:** 2023-11-07

**Authors:** Meng Xiao, Aolin Li, Yueqing Wang, Canqing Yu, Yuanjie Pang, Pei Pei, Ling Yang, Yiping Chen, Huaidong Du, Dan Schmidt, Daniel Avery, Qiang Sun, Junshi Chen, Zhengming Chen, Liming Li, Jun Lv, Dianjianyi Sun, China Kadoorie Biobank Collaborative Group, Junshi Chen, Zhengming Chen (PI, Robert Clarke, Rory Collins, Yu Guo, Liming Li (PI, Jun Lv, Richard Peto, Robin Walters, Daniel Avery, Derrick Bennett, Ruth Boxall, Sue Burgess, Ka Hung Chan, Yumei Chang, Yiping Chen, Zhengming Chen, Johnathan Clarke, Robert Clarke, Huaidong Du, Ahmed Edris Mohamed, Zammy Fairhurst-Hunter, Hannah Fry, Mike Hill, Michael Holmes, Pek Kei Im, Andri Iona, Maria Kakkoura, Christiana Kartsonaki, Rene Kerosi, Kuang Lin, Mohsen Mazidi, Iona Millwood, Sam Morris, Qunhua Nie, Alfred Pozarickij, Paul Ryder, Saredo Said, Dan Schmidt, Paul Sherliker, Becky Stevens, Iain Turnbull, Robin Walters, Lin Wang, Neil Wright, Ling Yang, Xiaoming Yang, Pang Yao, Yu Guo, Xiao Han, Can Hou, Jun Lv, Pei Pei, Chao Liu, Canqing Yu, Qingmei Xia, Zengchang Pang, Ruqin Gao, Shanpeng Li, Haiping Duan, Shaojie Wang, Yongmei Liu, Ranran Du, Yajing Zang, Liang Cheng, Xiaocao Tian, Hua Zhang, Yaoming Zhai, Feng Ning, Xiaohui Sun, Feifei Li, Silu Lv, Junzheng Wang, Wei Hou, Wei Sun, Shichun Yan, Xiaoming Cui, Chi Wang, Zhenyuan Wu, Yanjie Li, Quan Kang, Huiming Luo, Tingting Ou, Xiangyang Zheng, Zhendong Guo, Shukuan Wu, Yilei Li, Huimei Li, Ming Wu, Yonglin Zhou, Jinyi Zhou, Ran Tao, Jie Yang, Jian Su, Fang Liu, Jun Zhang, Yihe Hu, Yan Lu, Liangcai Ma, Aiyu Tang, Shuo Zhang, Jianrong Jin, Jingchao Liu, Mei Lin, Zhenzhen Lu, Lifang Zhou, Changping Xie, Jian Lan, Tingping Zhu, Yun Liu, Liuping Wei, Liyuan Zhou, Ningyu Chen, Yulu Qin, Sisi Wang, Xianping Wu, Ningmei Zhang, Xiaofang Chen, Xiaoyu Chang, Mingqiang Yuan, Xia Wu, Xiaofang Chen, Wei Jiang, Jiaqiu Liu, Qiang Sun, Faqing Chen, Xiaolan Ren, Caixia Dong, Hui Zhang, Enke Mao, Xiaoping Wang, Tao Wang, Xi zhang, Kai Kang, Shixian Feng, Huizi Tian, Lei Fan, XiaoLin Li, Huarong Sun, Pan He, Xukui Zhang, Min Yu, Ruying Hu, Hao Wang, Xiaoyi Zhang, Yuan Cao, Kaixu Xie, Lingli Chen, Dun Shen, Xiaojun Li, Donghui Jin, Li Yin, Huilin Liu, Zhongxi Fu, Xin Xu, Hao Zhang, Jianwei Chen, Yuan Peng, Libo Zhang, Chan Qu

**Affiliations:** a Department of Epidemiology & Biostatistics, School of Public Health, Peking University, Beijing, 100191, China; b Peking University Center for Public Health and Epidemic Preparedness & Response, Beijing, 100191, China; c Key Laboratory of Epidemiology of Major Diseases (Peking University), Ministry of Education, Beijing, 100191, China; d Medical Research Council Population Health Research Unit at the University of Oxford, Oxford, United Kingdom; e Clinical Trial Service Unit and Epidemiological Studies Unit (CTSU), Nuffield Department of Population Health, University of Oxford, United Kingdom; f NCDs Prevention and Control Department, Pengzhou CDC, Pengzhou, Sichuan, 611930, China; g China National Center for Food Safety Risk Assessment, Beijing, 100022, China

**Keywords:** Marital status, Morbidity risk, Mortality risk, Phenome-wide landscape, Chinese

## Abstract

**Background:**

A comprehensive depiction of long-term health impacts of marital status is lacking.

**Methods:**

Sex-stratified phenome-wide association analyses (PheWAS) of marital status (living with vs. without a spouse) were performed using baseline (2004–2008) and follow-up information (ICD10-coded events till Dec 31, 2017) from the China Kadoorie Biobank (CKB). We estimated adjusted hazard ratios (aHRs) to evaluate the associations of marital status with morbidity risks of phenome-wide significant diseases or sex-specific top-10 death causes in China documented in 2017. Additionally, the association between marital status and mortality risks among participants with major chronic diseases at baseline was assessed.

**Findings:**

During up to 11.1 years of the median follow-up period, 1,946,380 incident health events were recorded among 210,202 men and 302,521 women aged 30–79. Marital status was found to have phenome-wide significant associations with thirteen diseases among men (*p* < 9.92 × 10^−5^) and nine diseases among women (*p* < 9.33 × 10^−5^), respectively. After adjusting for all disease-specific covariates in the final model, participants living without a spouse showed increased risks of schizophrenia, schizotypal and delusional disorders (aHR [95% CI]: 2.55, [1.83–3.56] for men; 1.49, [1.13–1.97] for women) compared with their counterparts. Additional higher risks in overall mental and behavioural disorder (1.31, 1.13–1.53), cardiovascular disease (1.07, 1.04–1.10) and cancer (1.06, 1.00–1.12) were only observed among men without a spouse, whereas women living without a spouse were at lower risks of developing genitourinary diseases (0.89, 0.85–0.93) and injury & poisoning (0.93, 0.88–0.97). Among 282,810 participants with major chronic diseases at baseline, 39,166 deaths were recorded. Increased mortality risks for those without a spouse were observed in 12 of 21 diseases among male patients and one of 23 among female patients. For patients with any self-reported disease at baseline, compared with those living with a spouse, the aHRs (95% CIs) of mortality risk were 1.29 (1.24–1.34) and 1.04 (1.00–1.07) among men and women without a spouse (*p*_interaction_<0.0001), respectively.

**Interpretation:**

Long-term associations of marital status with morbidity and mortality risks are diverse among middle-aged Chinese adults, and the adverse impacts due to living without a spouse are more profound among men. Marital status may be an influential factor for health needs.

**Funding:**

The National Natural Science Foundation of China, the 10.13039/501100017647Kadoorie Charitable Foundation, the 10.13039/501100012166National Key R&D Program of China, the Chinese Ministry of Science and Technology, and the UK Wellcome Trust.

**Research in context**

**Evidence before this study**

We searched PubMed for articles published up to June 30, 2023, using combined terms of marital status and health outcomes (any outcome of interest in this study). No restriction on study type or language was implemented. Relevant studies were also found through checking reference lists of identified articles. Previous evidence has suggested that marital status has an impact on health. However, most studies were carried out in the Western populations, with conflicting conclusions. Many diseases, such as mental disorders, have not been studied in relation to marital status. Comprehensive evidence concerning marital status and health outcomes, including morbidity and mortality risk, is still scarce.

**Added value of this study**

This is the first large cohort study on Chinese men and women showing the comprehensive wide landscapes of morbidity and mortality risks related to marital status. The results of PheWAS showed that marital status was associated with morbidity risks of thirteen and nine diseases in men and women, respectively, of which the majority of associations were hardly reported before. The prospective analyses further demonstrated that living without a spouse, regardless of men or women, was independently associated with increased risks of incident schizophrenia, schizotypal and delusional disorders, but lower risks of overall respiratory diseases and dorsalgia. Besides, there was a significant sex difference in the association between marital status and disease risk. Most diseases associated with living without a spouse showed increased risk among men. In addition, men living without a spouse and prevalent with major chronic disorders or diseases had a higher death risk compared with women.

**Implications of all the available evidence**

Marriage, a close and long-lasting social relationship in adulthood, should be considered as an essential factor related to morbidity risks of multiple diseases as well as long-term mortality risks of patients with chronic diseases. Pertinent interventions could be distinguishingly provided for men and women with different marital statuses and health issues.

## Introduction

The marriage rate (number of marriages registered per 1000 people) in China has fallen by almost half, from 9.85‰ in 2013 to 5.77‰ in 2020.^1^ Meanwhile, the divorce rate (number of divorces per 1000 people) has increased by over 1.2 folds from 2.57‰ to 3.09‰.^1^ These shifting trends in marriage and fertility rates have coincided with an increase in the average age at first marriage and divorce over the past decade. As a result, the structure of Chinese households and the population has undergone significant changes, presenting substantial challenges to health systems.^1^

Marriage, one of the most intimate and long-lasting social relationships in adulthood, can have profound effects on the health and well-being of individuals and their family members. Previous studies have associated marriage with reductions in morbidity and mortality related to cardiovascular diseases^2^^,^^3^ and cancer.^4^^,^^5^ The reasons behind these associations may extend beyond traditional disease risk factors. However, there remains a limited understanding of the long-term impacts of marital status on a whole spectrum of diseases and disorders, especially among Asian populations. Insufficient understanding of the association between marital status and health hinders efforts to address health challenges in a population with rapid demographic changes.

In the present large prospective cohort study, our objective was to establish a sex-specific phenome-wide atlas of marital status and more than 500 diseases associated with marital status among over half a million Chinese adults. Additionally, we aimed to examine the associations of marital status with phenome-wide significant diseases and the top 10 sex-specific causes of death in the Chinese population.^6^ We further analyzed mortality risk relating to marital status among patients with major chronic disorders or diseases at baseline.

## Methods

### Study participants and design

CKB is a population-based prospective study of 512,725 adults aged 30–79 recruited from five urban and five rural areas of China, chosen according to local disease patterns, exposures, population stability, quality of death and disease registries, local commitment and capacity, during 2004–2008. In the recruitment, about a third (33% in rural areas and 27% in urban areas) response rate was reached in local communities. The long-term follow-up of all study participants for morbidity and mortality started after the baseline survey. Additionally, the CKB conducted repeated surveys every five years among 5% of randomly selected participants. Details of the study design and implementation of CKB have been reported previously.^7^ All participants provided written informed consent. Ethics approvals were obtained from the Chinese Center for Disease Control and Prevention (CDC), local CDCs in the ten study areas, and Oxford University.

The present study included 512,723 participants after excluding two with missing data on BMI. All analyses were performed separately for men and women. We first explored a wide landscape of diseases among 210,202 men and 302,521 women. Then, the morbidity risks of 30 incident disease categories were assessed among participants free of the corresponding diseases at baseline. In addition, mortality risks were evaluated among 185 to 123,329 male patients and 79 to 159,481 female patients with self-reported major chronic disease(s) at baseline. Mortality risks were not assessed among patients with lung, liver, and prostate cancer at baseline (N = 129, 37 and 5) due to insufficient statistical power. Figure S1 shows a flow chart of the inclusion of study participants.

### Assessment of marital status and other covariates

All baseline information was collected through face-to-face interviews and anthropometric measurements taken by trained staff using standardized procedures. The marital status question included four options for answer: married/cohabitated (cohabitated: living together for a long time without obtaining a marriage license or having a marriage ceremony, but with having given birth to and jointly raising children), widowed, separated/divorced, and never married. This analysis categorized participants into “with a spouse” (married/cohabitated) and “without a spouse” (widowed, separated/divorced, and never married). We also compared the marital status of the same individuals who participated in the baseline, first (2008) and second (2013–14) resurveys. Other covariates included: (1) socio-demographic characteristics; (2) lifestyle behaviours and anthropometric measures; and (3) individual and family history of diseases, self-reported satisfaction level of life, and menopausal status (for women only) (detailed in Table S1).

### Ascertainment of outcomes

The incident disease and death events during follow-up were identified by linking local death and disease registries and health insurance databases till Dec 31, 2017. The primary outcomes examined in this study (Table S2) were coded following the International Classification of Diseases, Tenth Revision (ICD-10).

In the initial analysis of the landscape of diseases, self-reported diseases at baseline (also coded with ICD-10) and newly documented diseases during follow-up were used to define participants who had developed any diseases as of Dec 31, 2017. We used a tool for PheWAS in the R environment, the R PheWAS,^8^ to convert unique 6880 ICD-10 codes to PheWAS case and control groups. There were 1743 and 1784 hierarchical PheWAS codes (PheCodes) formed from grouped ICD-10 codes for men and women, respectively. After excluding diseases with less than 30 cases to maintain statistical power, 512 diseases for men and 544 diseases for women were retained for analysis. The subsequent prospective analysis only included 30 disease categories (Table S2). There are two rules for determining the disease categories: 1) the diseases were included if the marital status–disease association reached statistical significance after adjusting for all covariates in the PheWAS analysis, with the parent categories retained if there existed hierarchical relationships, or 2) the sex-specific top-10 death causes reported by the 2018 China Health Statistical Yearbook (Table S3).^6^ “Back pain” in PheWAS was uniformly changed to “dorsalgia” to ensure consistency with ICD-10 in our analyses.

### Statistical analysis

Baseline characteristics of all included participants by marital status were expressed as mean ± SD for normally distributed continuous variables, median and interquartile range (IQR) for non-normally distributed continuous variables, or n (%) for categorical variables.

The sex-specific PheWAS analyses using non-conditional logistic regression were performed to investigate the associations between marital status and morbidity risks. Three models were stepwise adjusted. In Model 1, covariates included age, study area, the highest education level, household income, and household size. Model 2 further adjusted for alcohol drinking, smoking, dietary habits, physical activity, and BMI. Model 3 additionally adjusted for the history of diabetes, hypertension, respiratory disease, CVD, or cancer at baseline, family history of the analyzed disease (adjusted for only in corresponding analysis), self-reported satisfaction level of life, and menopausal status (for women only). Volcano plots were plotted based on the results of PheWAS to visualize the increase or decrease in morbidity risks.

The Cox proportional hazards regression models with age as the time scale were used to evaluate the associations between marital status and incident risks of 30 disease categories among participants free of the related diseases at baseline and the mortality risks relating to marital status among patients with major chronic diseases at baseline. The Cox models were stratified by age at risk (five-year bands) and study areas (ten groups) and adjusted for other covariates as in the PheWAS analysis, providing estimates of adjusted hazard ratios (aHRs) and 95% confidence intervals (CIs). Person-years were calculated by the enrollment date until the onset of the outcome of interest, death, loss to follow-up, or Dec 31, 2017, whichever came first. Kaplan–Meier survival curves were drawn, and the log-rank test was used to compare the mortality risks of patients with different marital statuses. The interaction between sex and marital status on morbidity or mortality risk was examined using likelihood-ratio tests by comparing models with and without cross–product interaction terms. Proportional hazard assumptions were tested by Schoenfeld residuals, and no violation was visually detected.

All analyses were performed with R 4.0.3. PheWAS analysis was conducted using the R-package PheWAS.^8^ The phenome-wide *p*-value was Bonferroni corrected (0.05 divided by the number of diseases in the group). The level of significance was set at a two-sided *p* < 0.05.

### Role of the funding source

The founders had no role in study design, data collection, data analysis, data interpretation, or report writing. The corresponding author had full access to all data in the study or the decision to submit the article for publication.

## Results

Among 210,202 men and 302,521 women, 92.9% and 89.0% lived with a spouse at baseline, respectively. Compared with participants living with a spouse, those living without a spouse were older, with lower socioeconomic status, more physically inactive, and had worse life satisfaction and a higher prevalence of chronic diseases (Table 1). Of men living without a spouse, 56.9%, 22.6%, and 20.5% were widowed, separated or divorced, and never married, respectively (Table S4); the respective proportions among women were 84.2%, 13.7%, and 2.1% (Table S5). Of 6080 men and 8738 women who participated in the 2013–14 resurvey, only 5.3% and 2.6% changed their marital status (with vs. without a spouse) after a median of 7.4 years of interval, respectively.

**Table 1:** Baseline characteristics of participants according to marital status.

	Men		Women	
	With a spouse	Without a spouse	With a spouse	Without a spouse
**Number of participants**	195,296	14,906	269,177	33,344
**Age, yr (SD)**	52.5 (10.7)	57.7 (12.0)	50.3 (9.9)	60.5 (10.5)
**Urban area, n (%)**	86,096 (44.1)	5260 (35.3)	115,929 (43.1)	18,907 (56.7)
**Highest education, n (%)**				
Primary school and lower	79,599 (40.8)	9176 (61.6)	149,241 (55.4)	22,349 (67.0)
Middle or high school	99,931 (51.2)	4959 (33.3)	107,902 (40.1)	9590 (28.8)
College and higher	15,766 (8.1)	771 (5.2)	12,034 (4.5)	1405 (4.2)
**Household income (RMB/year), n (%)**				
<10,000	47,052 (24.1)	7671 (51.5)	74,176 (27.6)	15,836 (47.5)
10,000–19,999	55,559 (28.4)	3985 (26.7)	80,138 (29.8)	9276 (27.8)
≥20,000	92,685 (47.5)	3250 (21.8)	114,863 (42.7)	8232 (24.7)
**Household size, n (%)**				
1–2	134,847 (69.0)	6342 (42.5)	188,686 (70.1)	16,030 (48.1)
3–5	35,031 (17.9)	7613 (51.1)	45,867 (17.0)	15,415 (46.2)
≥6	25,418 (13.0)	951 (6.4)	34,624 (12.9)	1899 (5.7)
**Smoking, n (%)**				
Nonsmoker	50,046 (25.6)	3870 (26.0)	261,995 (97.3)	30,729 (92.2)
Former smoker	13,198 (6.8)	881 (5.9)	828 (0.3)	373 (1.1)
Current smoker (cigarette or equivalent per day)				
1–9	23,032 (11.8)	2347 (15.7)	3488 (1.3)	1212 (3.6)
10–19	35,400 (18.1)	2634 (17.7)	1810 (0.7)	655 (2.0)
≥20	73,620 (37.7)	5174 (34.7)	1056 (0.4)	375 (1.1)
**Alcohol consumption, n (%)**				
Not daily	137,883 (70.6)	10,647 (71.4)	264,630 (98.3)	32,407 (97.2)
Daily (pure alcohol per day)				
1–14 g/d	1700 (0.9)	116 (0.8)	672 (0.2)	135 (0.4)
15–29 g/d	7575 (3.9)	475 (3.2)	807 (0.3)	148 (0.4)
30–59 g/d	13,450 (6.9)	929 (6.2)	582 (0.2)	113 (0.3)
Ex-drinker or ≥60 g/d	34,688 (17.8)	2739 (18.4)	2486 (0.9)	541 (1.6)
**Physical activity, MET-hours/day (IQR)**	19.1 (9.9–32.7)	15.1 (8.3–28.0)	17.6 (11.2–29.1)	11.7 (8.4–19.4)
**Healthy dietary habit, n (%)**^a^	14,106 (7.2)	676 (4.5)	24,597 (9.1)	2940 (8.8)
**Body-mass index, kg/m**^**2**^**(SD)**	23.5 (3.2)	22.5 (3.2)	23.8 (3.4)	23.7 (3.7)
**Life satisfaction, n (%)**				
Very satisfied	34,644 (17.7)	2177 (14.6)	48,457 (18.0)	4598 (13.8)
Satisfied	103,179 (52.8)	6507 (43.7)	136,255 (50.6)	15,587 (46.7)
Neither satisfied nor dissatisfied	49,453 (25.3)	4910 (32.9)	74,920 (27.8)	10,704 (32.1)
Dissatisfied	7605 (3.9)	1180 (7.9)	9025 (3.4)	2246 (6.7)
Very dissatisfied	415 (0.2)	132 (0.9)	520 (0.2)	209 (0.6)
**Prevalent diseases at baseline, n (%)**				
Diabetes	10,864 (5.6)	819 (5.5)	15,432 (5.7)	3185 (9.6)
Hypertension	72,179 (37.0)	6653 (44.6)	86,214 (32.0)	15,542 (46.6)
Respiratory disease^b^	20,010 (10.2)	2473 (16.6)	18,681 (6.9)	3842 (11.5)
CVD	9220 (4.7)	851 (5.7)	10,357 (3.8)	2701 (8.1)
Cancer	881 (0.5)	87 (0.6)	1375 (0.5)	235 (0.7)
**Menopause, n (%)**	–	–	131,939 (49.0)	26,986 (80.9)

SD, standard deviation; MET, metabolic equivalent of task; IQR, interquartile range; CVD, cardiovascular disease.

a The dietary habit was scored according to the self-reported eating habits of the participants, including eating fresh fruit per day, eating fresh vegetables per day, eating red meat 1–6 days per week, eating fish at least one day per week, and eating legumes at least 4 days per week. One or zero point for each healthy or unhealthy habit, respectively, and those with a diet score of 4 or 5 were classified into the “healthy diet” group.

b Respiratory disease includes chronic obstructive pulmonary disease/emphysema pulmonum/pulmonary heart disease, tuberculosis, and asthma.

### PheWAS of marital status

**Fig. 1:**
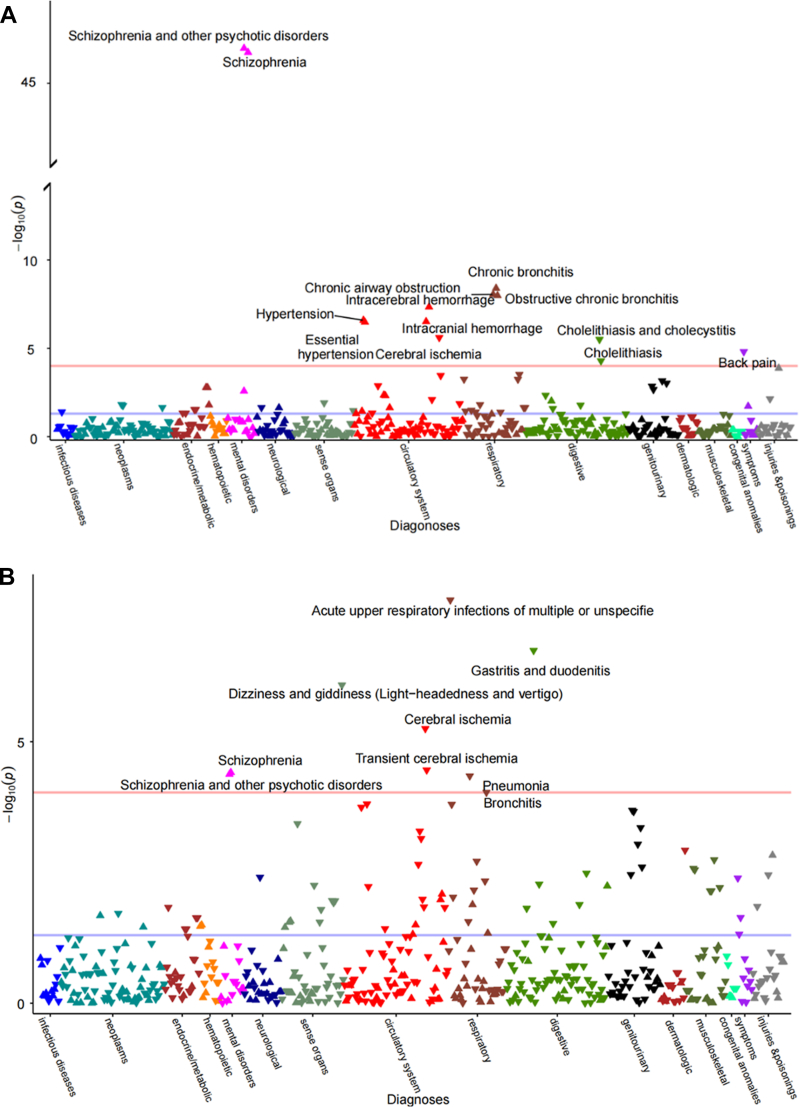
**Wide landscapes of diseases associated with marital status (living without vs. with a spouse) in Chinese men (A) and women (B)**. The x-axis represents the disease categorical group, and the y-axis represents the negative log (10) of the phenome-wide *P* value. Purple and red lines indicate the cut-off for 0.05 and phenome-wide significance levels, respectively. Upward and downward triangles indicate OR ≥ 1 and OR < 1, respectively. The models were adjusted for age, study area, highest education, household income, and household size.

In the PheWAS analyses, there were 504 and 536 diseases mapped among men and women, respectively (Tables S6 and S7). Sex-stratified landscapes of diseases with adjustment for socio-demographic characteristics were displayed in Fig. 1, with volcano plots in Figures S2 and S3. Among diseases that reached the *p* < 0.05 level, most associations were observed in circulatory and respiratory systems for both men and women. After multiple testing corrections, thirteen out of 504 diseases and nine out of 536 diseases remained phenome-wide significance for men (*p* < 9.92∗10^−5^) and women (*p* < 9.33∗10^−5^), respectively. The marital status of living without a spouse showed the strongest association with increased risk of schizophrenia and other psychotic disorders (odds ratio, OR = 3.82, 95% CI 3.64–4.01 for men; OR = 1.35, 95% CI 1.21–1.50 for women). Men without a spouse had a higher risk of hypertension (OR = 1.11, 95% CI 1.07–1.15) but a lower risk of back pain (OR = 0.76, 95% CI 0.68–0.86) than those with a spouse. Women without a spouse had a lower risk of pneumonia (OR = 0.90, 95% CI 0.86–0.95) than those with a spouse.

### Marital status and morbidity risks of 30 disease categories

In the association analyses of 30 disease categories, the shortest and longest median follow-ups were 10.6 (IQR 9.6–11.8 years; 4,125,335 person-years) and 11.1 years (IQR 10.2–12.1 years; 5,054,359 person-years), respectively. A total of 1,946,380 incident health events occurred, including 5952 mental and behavioural disorders, 114,980 CVDs, and 29,953 cancers (Table S8).

**Fig. 2:**
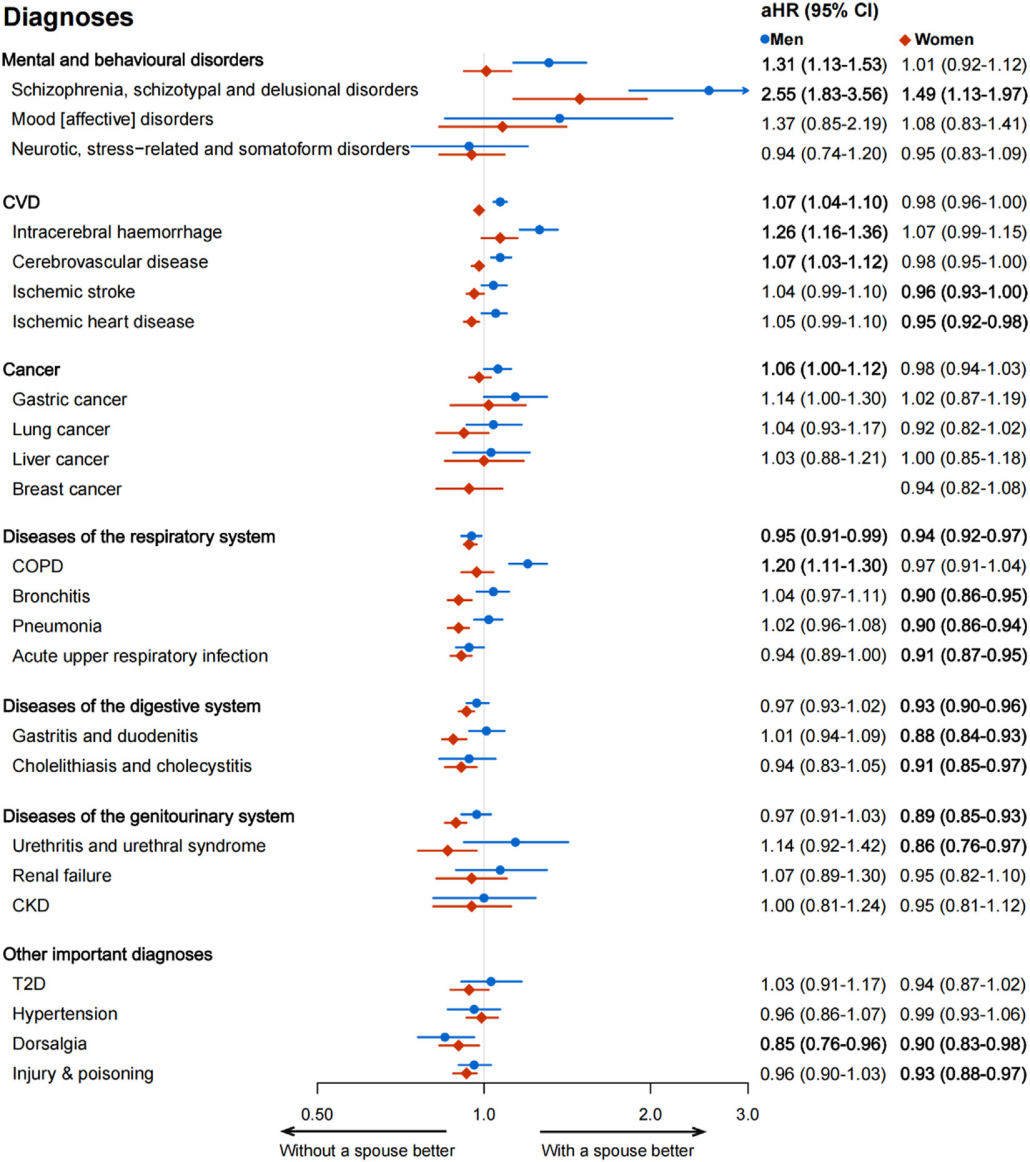
**Adjusted hazard ratios (aHRs) of selected disease categories for Chinese men (blue circle) and women (red diamond) lived without vs. with a spouse**. Models were stratified by age at risk (five-year bands) and study areas (ten groups) and further adjusted for highest education, household income, household size, alcohol drinking, smoking, dietary habits, BMI, physical activity, satisfaction level with life, history of diabetes, hypertension, respiratory disease, CVD, or cancer at baseline, family history of the analyzed disease (adjusted for only in corresponding analysis), self-reported satisfaction level of life, and menopausal status (for women only). Bold: *p* < 0.05.

After adjusting for all disease-specific covariates in the final model, compared with participants living with a spouse, those living without a spouse had higher risks of schizophrenia, schizotypal and delusional disorders (aHR = 2.55 [95% CI 1.83–3.56] for men, aHR = 1.49 [95% CI 1.13–1.97] for women), but lower risks of overall respiratory diseases (aHR = 0.95 [95% CI, 0.91–0.99] for men, aHR = 0.94 [95% CI 0.92–0.97] for women) and dorsalgia (aHR = 0.85 [95% CI 0.76–0.96] for men, aHR = 0.90 [95% CI 0.83–0.98] for women) (Fig. 2). The associations were robust in three adjusted models (Tables S9).

There was a sex difference in associations of marriage with several disease categories (Fig. 2, Tables S9). For example, we found increased risks in overall mental and behavioural disorder (aHR = 1.31, 95% CI 1.13–1.53), CVD (aHR = 1.07, 95% CI 1.04–1.10), cancer (aHR = 1.06, 95% CI 1.00–1.12), and COPD (aHR = 1.20, 95% CI 1.11–1.30) in men living without a spouse rather than in women. Women living without a spouse were at lower risks of developing genitourinary diseases (aHR = 0.89, 95% CI 0.85–0.93) and injury & poisoning (aHR = 0.93, 95% CI 0.88–0.97), but not for men.

The association results also showed differences between participants in urban and rural areas among both men and women (Table S10). For instance, the marriage-IHD and marriage-injury & poisoning associations were only observed among rural men (aHR = 1.09, 95% CI 1.02–1.16) and rural women (aHR = 0.92, 95% CI 0.87–0.97), respectively. Risk estimates did not substantially alter after excluding those who followed for no more than two years (Table S11), but the health advantages of marriage seemed more striking for those who were separated/divorced or unmarried (Table S12) and born after 1955 (Table S13).

### Marital status and mortality risk among patients

**Fig. 3:**
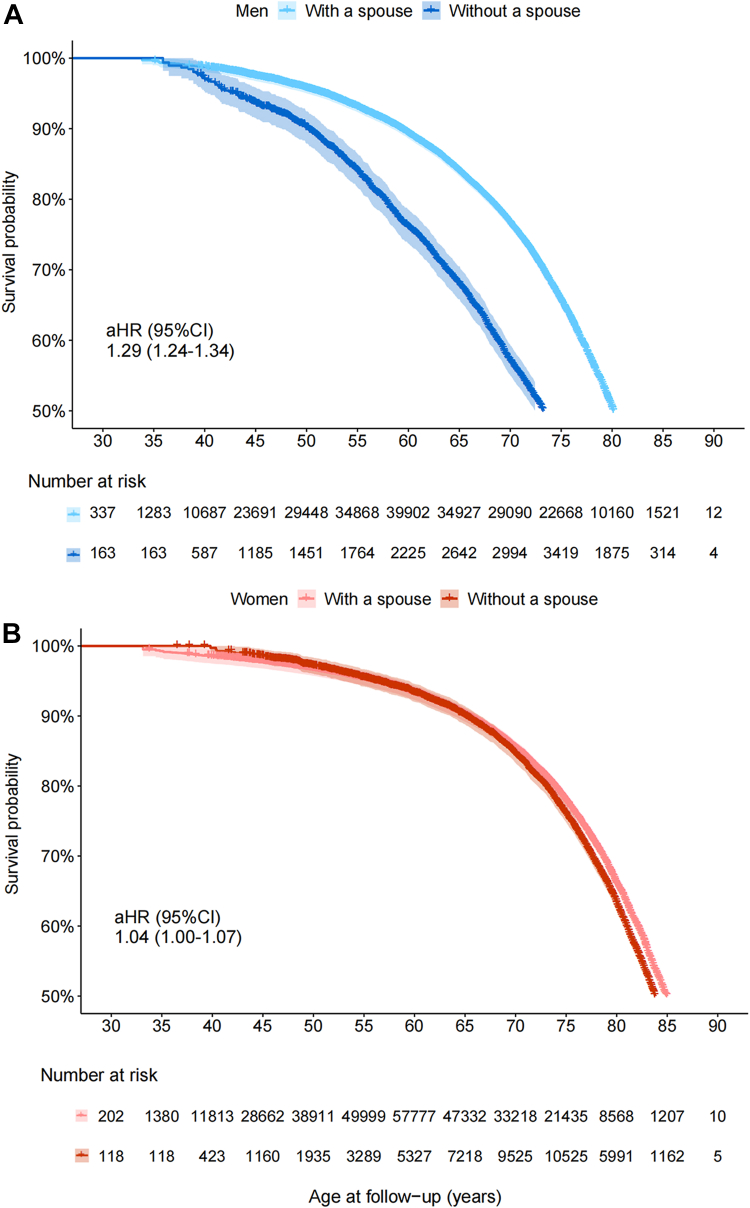
**Kaplan–Meier estimates of the mortality risk in patients live without vs. with a spouse**. Kaplan–Meier estimates of the mortality risk are shown in the male and female patients with diseases at baseline (Panel A and Panel B), respectively. In each analysis, patients lived with a spouse were taken as the reference group, and *p* values of Log-rank test were both <0.001. Adjusted hazard ratios (aHRs) were compared with those lived with a spouse; models were stratified by age at risk (five-year bands) and study areas (ten groups) and further adjusted for highest education, household income, household size, alcohol drinking, smoking, dietary habits, BMI, physical activity, satisfaction level with life, history of diabetes, hypertension, respiratory disease, CVD, or cancer at baseline, family history of the analyzed disease (adjusted for only in corresponding analysis), self-reported satisfaction level of life, and menopausal status (for women only).

During a median of 11.0 years (IQR 10.1–12.1 years; 3,008,770 person-years) of follow-up, a total of 39,166 deaths were documented among patients with self-reported common chronic diseases at baseline (Table S14). Increased mortality risks for those without a spouse were observed in 12 of 21 diseases among male patients and one of 23 among female patients (Table 2). The health advantages of marriage were found among both male and female patients with cholelithiasis/cholecystitis (aHR = 1.28 [95% CI, 1.05–1.56] for men, aHR = 1.12 [95% CI 1.00–1.25] for women). The male patients living without a spouse also had higher risks of mortality if they suffered from neurasthenia, CVD, diseases of the respiratory and digestive system, diabetes, hypertension, or fracture. For patients with any self-reported disease at baseline, compared with those living with a spouse, the aHRs of mortality risk were 1.29 (95% CI 1.24–1.34) and 1.04 (95% CI 1.00–1.07) for men and women living without a spouse (*p*_interaction_<0.0001), respectively (Fig. 3). Detailed results of sensitivity analyses can be found in Tables S15–S17.

**Table 2:** Adjusted hazard ratios (aHRs) of mortality risks for Chinese male and female patients lived without vs. with a spouse.

Diseases at baseline	Men			Women			*p*_for interaction_
	Deaths	Deaths per 1000 person-year	HR (95% CI)	Deaths	Deaths per 1000 person-year	HR (95% CI)	
**Mental and behavioural disorders**							
Psychosocial disorder							0.4762
With a spouse	75	17.92	1 (ref)	121	9.48	1 (ref)	
Without a spouse	23	15.45	0.42 (0.20, 0.92)	28	14.29	0.87 (0.52, 1.45)	
Neurasthenia							0.0252
With a spouse	167	10.78	1 (ref)	212	5.26	1 (ref)	
Without a spouse	30	25.07	**1.73 (1.10, 2.72)**	61	9.97	0.82 (0.59, 1.14)	
**CVD**							
Coronary heart disease							0.4851
With a spouse	1605	31.48	1 (ref)	1184	14.41	1 (ref)	
Without a spouse	225	52.92	**1.19 (1.03, 1.38)**	508	24.91	1.02 (0.91, 1.15)	
Stroke							0.0001
With a spouse	1787	43.99	1 (ref)	826	26.70	1 (ref)	
Without a spouse	244	76.21	**1.30 (1.13, 1.50)**	281	35.66	0.92 (0.79, 1.06)	
Rheumatic heart disease							0.5116
With a spouse	529	17.73	1 (ref)	531	7.40	1 (ref)	
Without a spouse	80	33.84	1.23 (0.95, 1.60)	206	16.89	1.08 (0.90, 1.30)	
**Cancer**							
Esophagus							0.4369
With a spouse	87	62.56	1 (ref)	20	24.03	1 (ref)	
Without a spouse	13	105.98	1.15 (0.54, 2.44)	7	57.10	2.25 (0.47, 10.82)	
Stomach							0.6961
With a spouse	64	44.47	1 (ref)	21	34.98	1 (ref)	
Without a spouse	12	101.80	0.66 (0.19, 2.38)	6	48.03	NA	
Intestine							0.3839
With a spouse	52	39.94	1 (ref)	30	25.01	1 (ref)	
Without a spouse	6	55.60	2.99 (0.33, 26.86)	10	38.83	1.64 (0.29, 9.21)	
Breast							NA
With a spouse	NA	NA	NA	102	20.50	1 (ref)	
Without a spouse	NA	NA	NA	21	31.87	1.31 (0.67, 2.57)	
Cervix							NA
With a spouse	NA	NA	NA	53	16.66	1 (ref)	
Without a spouse	NA	NA	NA	12	24.82	0.61 (0.18, 2.02)	
Others							0.5271
With a spouse	108	47.44	1 (ref)	46	16.94	1 (ref)	
Without a spouse	14	61.63	0.83 (0.30, 2.25)	17	32.17	2.51 (0.83, 7.64)	
**Diseases of the respiratory system**							
Pulmonary tuberculosis							0.0140
With a spouse	809	20.71	1 (ref)	321	10.50	1 (ref)	
Without a spouse	155	43.98	**1.51 (1.26, 1.82)**	122	20.49	1.03 (0.81, 1.30)	
Asthma							0.5087
With a spouse	284	25.86	1 (ref)	135	9.11	1 (ref)	
Without a spouse	57	50.20	**1.46 (1.04, 2.04)**	62	24.74	1.16 (0.79, 1.71)	
Chronic bronchitis/emphysema							0.0086
With a spouse	1752	30.08	1 (ref)	816	13.44	1 (ref)	
Without a spouse	325	63.09	**1.37(1.21, 1.55)**	334	31.44	1.01 (0.88, 1.17)	
**Diseases of the digestive system**							
Chronic hepatitis/cirrhosis							0.1309
With a spouse	608	17.17	1 (ref)	184	7.38	1 (ref)	
Without a spouse	71	35.77	**1.42 (1.08, 1.87)**	46	14.89	1.11 (0.75, 1.64)	
Peptic ulcer							0.3497
With a spouse	1351	12.05	1 (ref)	484	5.61	1 (ref)	
Without a spouse	160	24.09	**1.25 (1.06, 1.49)**	146	13.35	1.01 (0.82, 1.24)	
Cholelithiasis/cholecystitis							0.3038
With a spouse	1047	12.87	1 (ref)	1505	6.90	1 (ref)	
Without a spouse	123	26.85	**1.28 (1.05, 1.56)**	557	18.17	**1.12 (1.00, 1.25)**	
**Diseases of the genitourinary system**							
Chronic kidney disease							0.8447
With a spouse	382	14.70	1 (ref)	352	7.46	1 (ref)	
Without a spouse	27	17.43	0.8 (0.52, 1.21)	89	14.61	0.90 (0.69, 1.18)	
**Other important diseases**							
Diabetes							0.0041
With a spouse	2663	24.7	1 (ref)	2618	16.34	1 (ref)	
Without a spouse	357	50.00	**1.30 (1.16, 1.46)**	948	30.58	0.99 (0.91, 1.18)	
Hypertension							<0.0001
With a spouse	13,414	17.92	1 (ref)	9124	9.75	1 (ref)	
Without a spouse	2454	39.56	**1.27 (1.21, 1.33)**	3593	22.62	1.02 (0.98, 1.07)	
Fracture							0.0461
With a spouse	1823	9.79	1 (ref)	931	5.83	1 (ref)	
Without a spouse	265	22.34	**1.36 (1.19, 1.56)**	382	15.22	1.08 (0.95, 1.23)	
Head injury							0.6026
With a spouse	358	10.38	1 (ref)	104	5.05	1 (ref)	
Without a spouse	55	17.23	1.17 (0.85, 1.61)	34	10.08	0.87 (0.53, 1.43)	
Arthritis							0.5348
With a spouse	529	17.73	1 (ref)	531	7.40	1 (ref)	
Without a spouse	80	33.84	1.23 (0.95, 1.59)	206	16.89	1.09 (0.90, 1.31)	

Models were stratified by age at risk (five-year bands) and study areas (ten groups) and further adjusted for highest education, household income, household size, alcohol drinking, smoking, dietary habits, BMI, physical activity, satisfaction level with life, history of diabetes, hypertension, respiratory disease, CVD, or cancer at baseline, family history of the analyzed disease (adjusted for only in corresponding analysis), self-reported satisfaction level of life, and menopausal status (for women only). Bold: *P* < 0.05.

## Discussion

In this large prospective cohort study of Chinese adults, the results of PheWAS showed that marital status was associated with morbidity risks of thirteen and nine diseases among men and women, respectively, of which most associations were not reported before. The prospective analyses further demonstrated that living without a spouse, regardless of whether man or woman, was independently associated with increased risks of incident schizophrenia, schizotypal and delusional disorders, but lower risks of overall respiratory diseases and dorsalgia. Further, there was a significant sex difference in the association between marital status and disease risk. Most diseases associated with living without a spouse showed increased risk among men. In addition, men showed greater marital survival advantage among those prevalent with major chronic diseases at baseline than women.

Our study identified a positive association between living without a spouse and risks of schizophrenia, schizotypal and delusional disorders. The rationale of increased risk caused by living alone may lie in social support. Spouses in a successful marriage may provide emotional support and promote each other's mental health. A social network of being married may help reduce loneliness in later life, especially for men.^9^ It may help explain what we observed: men without a spouse were more likely to develop the broader category of mental and behavioural disorders than women. However, reverse causality might be an issue. Asymptomatic patients of schizophrenia were less likely to get married or more likely to get divorced or separated, due to social impairement and high heritability to the offspring.

Our analysis found that there may be a gender gap in the increased disease risk for people living without a spouse. For example, our analysis observed elevated risks of IH, cerebrovascular disease, cancer, and COPD among men but not among women who lived without a spouse. We also found that the marital status of living without a spouse was associated with an increased risk of IH, with a stronger association among men than among women. Most previous prospective studies were conducted in Western populations and did not observe an association of marital status (married/partnered vs. others) with total CVD,^10^ stroke,^11^^,^^12^ or IHD.^13^ A study based on the Swedish Twin Registry, including 10,058 pairs of twins, reported that single participants had a 22% increased risk of CVD compared with married ones (HR = 1.22, 95% CI 1.05–1.43 for men; HR = 1.22, 95% CI 1.04–1.42 for women).^2^ Results from a cohort study involving 14,853 male and 5493 female occupational participants in France showed similar results: the risk of cancer among single men increased by 86% compared with men with a spouse (HR = 1.86, 95% CI 1.12–3.07), while no such association for women (HR = 1.15, 95% CI 0.71–1.87).^4^ The potential mechanisms underlying the associations are not fully understood. Possible explanations include financial and emotional supports from spouse,^14^^,^^15^ the impacts of stress regulation on immune functions, endocrine systems, and inflammatory processes,^16^ and spousal promotion of healthy behaviours (e.g., regular diets, no smoking, or excessive drinking).^13^^,^^17^ We noticed that lower risks of CVD, COPD, and cancer associated with living with a spouse were only seen among men. It may be because the prevalence of risk factors for these diseases, such as smoking^18^ and excessive drinking,^19^ is considerably higher among Chinese men than women. The positive effect of marriage on healthy behaviours may lead to a greater attributable risk reduction among men.

However, living without a spouse does not necessarily increase risks for all diseases. Intriguingly, we discovered a lower risk of dorsalgia for both men and women without a spouse. There are several possible explanations. Married individuals were more likely to be overweight or obese among Chinese.^20^ Obesity may put an increased burden on the back. An analysis based on the English Longitudinal Study of Ageing found that a 5% and 25% increase in BMI was associated with an 11% (RR = 1.11, 95% CI 1.04–1.19) and 49% (RR = 1.49, 95% CI 1.20–1.84) higher risk of back pain, respectively.^21^ For women, the biological response to pregnancy and childbearing may also contribute to the increased risk of back pain.^22^

Our study suggested reduced risks of developing related diseases, such as injury & poisoning, bronchitis, pneumonia and diseases of the genitourinary system among women without a spouse. A recent finding from a global study,^23^ covering 90% of women worldwide, indicated that approximately 27% of women aged 15–49 had experienced physical or sexual violence from intimate partners. Women living without a spouse may have less exposure to certain behavioural or environmental hazards than their counterparts, for example, violence from spouses (especially for women in rural areas), exposure to passive smoking, and genitourinary diseases related to sexual activity coupled with the complex physiological structure of women.

Our results showed that for adults with major chronic diseases, a greater survival advantage was revealed among male patients than female patients. A latest pooled analysis of 16 Asian population cohorts found that the mortality risk of married patients was lower than that of unmarried ones, regardless of disease type (cancer, coronary heart disease, cerebrovascular disease, diabetes, or hypertension at baseline).^5^ However, the above analysis did not stratify by sex. Three early studies examined the association between marital status and adverse outcomes of IS patients within one year after discharge and obtained inconsistent results: lower all-cause mortality risk associated with being married,^3^^,^^24^ or no association.^25^ Two other studies followed up for 15 months^26^ and 20 years^27^ found that the absence of a spouse was associated with increased mortality risk among MI patients; however, with the association observed only among men^27^ or women.^26^ Possible explanations for the lower risk of long-term mortality among those with chronic illness who lived with a spouse include psychological and financial supports from the spouse contributing to improved medication and treatment adherence^28^ and healthcare services.^29^ The spouse can provide timely rescue and fulfill the role of caregiver,^30^ playing a crucial role in home care and in supporting healthcare providers. Given that women typically assume more caregiving responsibilities within the family, husbands are more likely to derive benefits from marriage.

Findings from the current study have implications for addressing health challenges in China. Consistent with the notion of social determinants of health,^31^ the findings are informative in making public policies and in designing targeted social interventions. As China has been undergoing significant demographic changes in the past few decades, such as rapid aging, increased divorce rate and reduced marriage rate, evidence-based public policies, in particular public health policies, and targeted social interventions are instrumental to achieving overall health goals in the country.

To our knowledge, this is the first study to comprehensively examine the relevance of marital status to the health landscape and yield novel associations hardly reported before in Asian populations. The main strengths of this study include the large sample size, comprehensive information collected at baseline, and complete ascertainment of death, morbidity, and hospitalization through linking to several well-established databases during a follow-up of over 10 years. These collectively enabled us to explore the sex-specific associations between marital status and long-term risk of over 500 diseases with adequate statistical power and to control comprehensively for confounding factors.

However, our analyses had several limitations. First, in addition to CVD, cancer, and COPD, the morbidity information of most diseases derived from database that included only hospitalization events. Hence, our results are more relevant to the severe status of diseases. Second, the initial Phecode system of the PheWAS was developed based on ICD-9-CM (International Classification of Diseases, Ninth Revision, Clinical Modification). The present study used ICD-10 codes, resulting in a lower than 2% of disease deletions when mapping to PheCodes. Nevertheless, a study has shown that using ICD-10 codes had limited influence on the results of PheWAS.^32^ Third, information on marital status used in the analyses was collected at baseline and our analysis did not reflect the impact of marital status changes, thus, there was a loss of information on remarriage, time of divorcing or widowing. Eventhough the marital status of most participants did not change based on information from repeated surveys. Fourth, main results were reported according to the two-group marital status (live with or without a spouse) by concerns regarding statistical power, which was relatively crude. Fifth, a small number of couples may have participated in the study, which could generate some degree of clustering. In addition, the CKB cohort, as a prospective cohort, was not designed to be representative of the general Chinese population, caution must be taken when generalising our findings to the broader population in China. Nevertheless, the inclusion of a vast number of participants from diverse populations may help generate important new discoveries about the association of many diseases, which will be applicable to other populations with different risk exposure distributions.^33^

In this large prospective cohort study of more than 0.5 million Chinese adults, marital status was independently associated with morbidity risks of multiple diseases as well as long-term mortality risks of patients with chronic diseases. Most diseases associated with living without a spouse showed increased risk among men. Men showed greater marital survival advantage among those with diseases at baseline than women. Our findings emphasise the importance of marriage on overall adulthood health. Men and women living without a spouse face more health challenges. Information from the current study may be instrumental to design targeted health policies and interventions.

## Contributors

DiS conceived of and designed the paper. LL, ZC, and JC, as the members of the CKB steering committee, designed and supervised the CKB study obtained funding, and, together with CY, YP, PP, LY, YC, HD, DaS, DA and QS acquired the data. MX, AL and YW analyzed the data, MX wrote the first draft of the manuscript. JL and DiS helped to interpret the results, and contributed to the critical revision of the manuscript for important intellectual content and approved the final version. All authors reviewed and approved the final manuscript. DiS is the guarantor.

## Data sharing statement

The access policy and procedures are available at www.ckbiobank.org.

## Declaration of interests

All the authors have indicated they have no potential conflicts of interest to disclose.

## Appendix A Supplementary data

Supplementary Material

Translated Abstract
