# Supplementary material for: A wide landscape of morbidity and mortality risk associated with marital status in 0.5 million Chinese men and women: a prospective cohort study
Source: Lancet Reg Health West Pac. 2023 Nov 7;42:100948. doi: 10.1016/j.lanwpc.2023.100948 (PMC10865043; doi:10.1016/j.lanwpc.2023.100948)
Supplement: Supplementary Material [file mmc1.docx]

**Supplementary material**

A Wide Landscape of Morbidity and Mortality Risk Associated with Marital Status in 0.5 Million Chinese Men and Women: A Prospective Cohort Study

[**Appendix Members of the China Kadoorie Biobank collaborative group** 3](#_Toc145925492)

[**Table S1 Diseases-specific adjusted covariates** 4](#_Toc145925493)

[**Table S2 Phecodes and ICD codes of included 30 disease categories** 5](#_Toc145925494)

[**Table S3 Sex-specific top-10 death causes based on 2018 China Health Statistical Yearbook** 7](#_Toc145925495)

[**Table S4 Detailed baseline characteristics of men according to marital status** 8](#_Toc145925496)

[**Table S5 Detailed baseline characteristics of women according to marital status** 10](#_Toc145925497)

[**Table S6 PheWAS results of marital status (living with vs. without a spouse) with 504 diseases in 210,202 men** 12](#_Toc145925498)

[**Table S7 PheWAS results of marital status (living with vs. without a spouse) with 536 diseases in 302,521 women** 45](#_Toc145925499)

[**Table S8 Incident cases and person-years during the follow-up** 73](#_Toc145925500)

[**Table S9 Adjusted hazard ratios of marital status (lived without vs. with a spouse) with 30 disease categories stratified by sex** 76](#_Toc145925501)

[**Table S10 Adjusted hazard ratios (aHRs) of marital status (lived without vs. with a spouse) with 30 disease categories stratified by sex and areas** 81](#_Toc145925502)

[**Table S11 Sex-stratified sensitivity analyses for associations of marital status and 30 disease categories after excluding those who followed for no more than two years (lived without vs. with a spouse)** 87](#_Toc145925503)

[**Table S12 Sex-stratified analyses for associations of four-group marital status (live with a spouse, widowed, separated/divorced, and unmarried) and 30 disease categories** 89](#_Toc145925504)

[**Table S13 Adjusted hazard ratios (aHRs) of marital status (lived without vs. with a spouse) with 30 disease categories stratified by sex and birth cohort** 93](#_Toc145925505)

[**Table S14 Death records of patients during the follow-up** 99](#_Toc145925506)

[**Table S15 Sex-stratified sensitivity analyses for associations of marital status and mortality risks of patients after excluding those who followed for no more than two years (lived without vs. with a spouse)** 101](#_Toc145925507)

[**Table S16 Sex-stratified analyses for associations of four-group marital status (married/cohabitated, widowed, separated/divorced, and unmarried) and mortality risks of patients** 102](#_Toc145925508)

[**Table S17 Adjusted hazard ratios (aHRs) of marital status (lived without vs. with a spouse) for mortality risks of patients stratified by sex and birth cohort** 105](#_Toc145925509)

[**Figure S1. Flow chart of study participants in the CKB cohort** 107](#_Toc145925510)

[**Figure S2. Volcano plot based on PheWAS results of marital status in men lived without vs. with a spouse** 108](#_Toc145925511)

[**Figure S3. Volcano plot of based on PheWAS results of marital status in women lived without vs. with a spouse** 109](#_Toc145925512)

**Appendix Members of the China Kadoorie Biobank collaborative group**

**International Steering Committee:** Junshi Chen, Zhengming Chen (PI), Robert Clarke, Rory Collins, Yu Guo, Liming Li (PI), Jun Lv, Richard Peto, Robin Walters. **International Co-ordinating Centre, Oxford:** Daniel Avery, Derrick Bennett, Ruth Boxall, Sue Burgess, Ka Hung Chan, Yumei Chang, Yiping Chen, Zhengming Chen, Johnathan Clarke; Robert Clarke, Huaidong Du, Ahmed Edris Mohamed, Zammy Fairhurst-Hunter, Hannah Fry, Mike Hill, Michael Holmes, Pek Kei Im, Andri Iona, Maria Kakkoura, Christiana Kartsonaki, Rene Kerosi, Kuang Lin, Mohsen Mazidi, Iona Millwood, Sam Morris, Qunhua Nie, Alfred Pozarickij, Paul Ryder, Saredo Said, Dan Schmidt, Paul Sherliker, Becky Stevens, Iain Turnbull, Robin Walters, Lin Wang, Neil Wright, Ling Yang, Xiaoming Yang, Pang Yao.

**National Co-ordinating Centre, Beijing:** Yu Guo, Xiao Han, Can Hou, Jun Lv, Pei Pei, Chao Liu, Canqing Yu, Qingmei Xia. **10 Regional Co-ordinating Centres: Qingdao CDC:** Zengchang Pang, Ruqin Gao, Shanpeng Li, Haiping Duan, Shaojie Wang, Yongmei Liu, Ranran Du, Yajing Zang, Liang Cheng, Xiaocao Tian, Hua Zhang, Yaoming Zhai, Feng Ning, Xiaohui Sun, Feifei Li. **Licang CDC:** Silu Lv, Junzheng Wang, Wei Hou. **Heilongjiang Provincial CDC:** Wei Sun, Shichun Yan, Xiaoming Cui. **Nangang CDC:** Chi Wang, Zhenyuan Wu,Yanjie Li, Quan Kang. **Hainan Provincial CDC:** Huiming Luo, Tingting Ou. **Meilan CDC:** Xiangyang Zheng, Zhendong Guo, Shukuan Wu, Yilei Li, Huimei Li. **Jiangsu Provincial CDC:** Ming Wu, Yonglin Zhou, Jinyi Zhou, Ran Tao, Jie Yang, Jian Su. **Suzhou CDC:** Fang Liu, Jun Zhang, Yihe Hu, Yan Lu, Liangcai Ma, Aiyu Tang, Shuo Zhang, Jianrong Jin, Jingchao Liu. **Guangxi Provincial CDC:** Mei Lin, Zhenzhen Lu. **Liuzhou CDC:** Lifang Zhou, Changping Xie, Jian Lan,Tingping Zhu,Yun Liu, Liuping Wei, Liyuan Zhou, Ningyu Chen, Yulu Qin, Sisi Wang. **Sichuan Provincial CDC:** Xianping Wu, Ningmei Zhang, Xiaofang Chen, Xiaoyu Chang. **Pengzhou CDC:** Mingqiang Yuan, Xia Wu, Xiaofang Chen, Wei Jiang, Jiaqiu Liu, Qiang Sun. **Gansu Provincial CDC:** Faqing Chen, Xiaolan Ren, Caixia Dong. **Maiji CDC:** Hui Zhang, Enke Mao, Xiaoping Wang, Tao Wang, Xi zhang. **Henan Provincial CDC:** Kai Kang, Shixian Feng, Huizi Tian, Lei Fan. **Huixian CDC:** XiaoLin Li, Huarong Sun, Pan He, Xukui Zhang. **Zhejiang Provincial CDC:** Min Yu, Ruying Hu, Hao Wang. **Tongxiang CDC**: Xiaoyi Zhang, Yuan Cao, Kaixu Xie, Lingli Chen, Dun Shen. **Hunan Provincial CDC:** Xiaojun Li, Donghui Jin, Li Yin, Huilin Liu, Zhongxi Fu. **Liuyang CDC:** Xin Xu, Hao Zhang, Jianwei Chen,Yuan Peng, Libo Zhang, Chan Qu.

**Table S1 Diseases-specific adjusted covariates**

| **Model** | **Adjusted covariates** | **Details of covariates** |
| --- | --- | --- |
| **1** | Demographic and socioeconomic characteristics | Age, sex, study area, the highest education level, household income, and household size (the number of people living together, including the participants themselves) |
| **2** | Lifestyle behaviors and anthropometric measures | Alcohol drinking (pure alcohol), smoking, dietary habits (each dietary habit was scored according to the self-reported eating habits of the participants, including eating fresh fruit per day, eating fresh vegetables per day, eating red meat 1-6 days per week, eating fish at least one day per week, and eating legumes at least 4 days per week. One or zero point for each healthy or unhealthy habit, respectively, and those with a diet score of 4 or 5 were classified into the “healthy diet” group), physical activity (metabolic equivalent task hours per day spent on activities), and BMI (calculated from weight/height2) |
| **3** | Individual and family history of diseases, self-reported satisfaction level of life, and menopausal status | History of diabetes, hypertension, respiratory disease, CVD, or cancer at baseline (based on the self-reported physician’s diagnosis; participants with measured systolic blood pressure≥140 mm/Hg or diastolic blood pressure≥90 mm/Hg, or self-reported medication were also considered to be hypertensive patients), family history of the analyzed disease (adjusted for only in corresponding analysis), self-reported satisfaction level of life, and menopausal status (for women only) |

**Table S2 Phecodes and ICD codes of included 30 disease categories**

| **No.** | **Disease categories** | **ICD-10** | **Phecodes** |
| --- | --- | --- | --- |
| 1 | Mental and behavioural disorders | F00-F69, F99 | 290-291.4, 292.2, 295-296.22, 300-306, 312.3, 315,316-317.1, 318, 324.1, 327-327.1, 327.4, 327.5-327.6, 605 |
| 2 | Schizophrenia, schizotypal and delusional disorders | F20-F29 | 291.1, 295.1-295.3, 296.22, 301.1 |
| 3 | Mood [affective] disorders | F30-F39 | 296-296.22, 300.4, 301 |
| 4 | Neurotic, stress-related and somatoform disorders | F40-F48 | 295, 300-300.3, 300.8-300.9, 303-304, 306 |
| 5 | CVD | I00-I09, I20-I99 | 41.21, 90, 217.1, 289.4, 324, 394-395.4, 401.3, 411.1-416, 420.1-426.8, 427.1-427.61, 427.8, 428.1-429.1, 430-433.3, 433.32-440.9, 442-444.2, 446.1, 446.9-456, 458.1-459, 459.9, 530.2, 747.13 |
| 6 | IH | I61 | 430.2 |
| 7 | Cerebrovascular disease | I60-I69 | 324, 401.3, 430-433.3, 433.32-433.8, 442.4, 459.9 |
| 8 | IS | I63 | 433.11, 433.2-433.21 |
| 9 | IHD | I20-I25 | 411.1-414, 440 |
| 10 | Cancer | C00-C97 | 145-157, 159-172.2, 174.11, 180.1, 182, 184.1-185, 187.1-187.2, 189.11-189.12, 189.21-190, 191.1-194, 195.1-195.3, 198-198.7, 200, 201-204.4, 230, 270.35, 277 |
| 11 | Gastric cancer | C16 | 151 |
| 12 | Lung cancer | C33-C34 | 165.1 |
| 13 | Liver cancer | C22 | 155-155.1 |
| 14 | Breast cancer | C50 | 174.11 |
| 15 | Diseases of the respiratory system | J00-J99 | 41, 41.2, 79, 117, 136, 464-473.3, 474-476, 479-497, 500-509.3, 510, 512, 513.8, 519-519.8, 1010 |
| 16 | COPD | J41-J44 | 496-496.21 |
| 17 | Bronchitis | J20-J21, J40-J42 | 41, 41.2, 79, 483, 496.2, 497 |
| 18 | Pneumonia | J12-J18 | 117, 136, 480-480.5, 503 |
| 19 | Acute upper respiratory infection | J00-J06 | 41.2, 464-465.4, 474.1, 483 |
| 20 | Diseases of the digestive system | K00-K93 | 10, 70.4-70.9, 79, 90-90.2, 133, 208, 211, 317.1-317.11, 441-441.2, 520-530.7, 531-531.5, 535-558, 560.1-560.4, 561.1, 562.1-564.1, 565-571.81, 573-573.1, 573.4, 574.1-575.8, 577-578.9, 681.2, 706.2, 785, 853, 990, 1011 |
| 21 | Gastritis and duodenitis | K29 | 317.1, 535-535.8 |
| 22 | Cholelithiasis and cholecystitis | K80-K81 | 574.1-574.3 |
| 23 | Diseases of the genitourinary system | N00-N99 | 10, 80, 90-90.3, 180.3, 184.2, 256, 269, 568.1, 580.11-585.3, 586-586.4, 588-588.1, 590-597.2, 599-599.1, 599.4, 600-609.1, 610.1-610.3, 610.8, 611.3-612.2, 613-627.4, 628, 634, 674, 709.7, 1011 |
| 24 | Urethritis and urethral syndrome | N34 | 90, 592.2-592.21 |
| 25 | Renal failure | N17-N19 | 585.1-585.3 |
| 26 | CKD | N02-N03, N07, N11, N18 | 580.11-580.14, 580.32, 585.3, 586.4, 590, 593 |
| 27 | T2D | E11 | 250.2 |
| 28 | Hypertension | I10 | 401.1 |
| 29 | Dorsalgia (Back pain in Phecode) | M54 | 760 |
| 30 | Injury, poisoning and certain other consequences of external causes | S00-S99, T00-T98 | 80, 81, 136, 316, 317, 351, 359.2, 429.1, 430, 510.2, 525, 723, 726, 728, 780, 783, 800-830, 840-851, 854-930, 941-949, 952, 958, 958.1-958.2, 960-965, 965.3, 966-990, 1000, 1001, 1006-1009, 1011-1015 |

**Abbreviations:** CVD, cardiovascular disease; IH, intracerebral hemorrhage; IHD, ischemic heart disease; IS, ischemic stroke; COPD, chronic obstructive pulmonary disease; CKD, chronic kidney disease; T2D, type 2 diabetes.

**Table S3 Sex-specific top-10 death causes based on 2018 China Health Statistical Yearbook**

| **Disease** | **Urban** | |  | **Rural** | |
| --- | --- | --- | --- | --- | --- |
|  | **Mortality in 2017 (1/100,000)** | **proportion (100%)** |  | **Mortality in 2017 (1/100,000)** | **proportion (100%)** |
| Total | 615.66 | 100 |  | 679.26 | 100 |
| Malignant neoplasms | 160.72 | 26.11 |  | 156.70 | 23.07 |
| Cerebrovascular disease | 126.58 | 20.56 |  | 157.48 | 23.18 |
| Coronary heart disease | 115.32 | 18.73 |  | 122.04 | 17.97 |
| Diseases of the respiratory system | 67.20 | 10.92 |  | 78.57 | 11.57 |
| Lung cancer | 48.42 | 7.86 |  | 43.78 | 6.45 |
| Intracerebral hemorrhage | 47.54 | 7.72 |  | 68.39 | 10.07 |
| Ischemic stroke | 40.77 | 6.62 |  | 48.85 | 7.19 |
| Injury & poisoning | 36.34 | 5.90 |  | 52.92 | 7.79 |
| Liver cancer | 22.62 | 3.67 |  | 26.48 | 3.90 |
| Diabetes | 17.95 | 2.92 |  | 13.92 | 2.05 |
| Gastric cancer | 17.45 | 2.83 |  | 21.18 | 3.12 |
| Breast cancer | 4.76 | 0.77 |  | 3.50 | 0.52 |
| Glomerular and tubulointerstitial diseases | 3.96 | 0.64 |  | 5.23 | 0.77 |

**Table S4 Detailed** **baseline characteristics of men according to marital status**

|  | **With a spouse** |  | **Without a spouse** | | | | |  |
| --- | --- | --- | --- | --- | --- | --- | --- | --- |
|  | **Married/**  **cohabitated** |  | **Widowed** | | **Separated/**  **divorced** | | **Never married** |  |
| **Number of participants** | 195,296 |  | 8,488 | 3,369 | | 3,049 | | |
| **Age, yr (SD)** | 52.5 (10.7) |  | 64.0 (8.7) | 49.0 (9.5) | | 49.8 (11.5) | | |
| **Body-mass index, kg/m^2^ (SD)** | 23.5 (3.2) |  | 22.4 (3.2) | 23.0 (3.3) | | 21.9 (3.3) | | |
| **Physical activity, MET-hours/day (IQR)** | 19.1  (9.9-32.7) |  | 12.6  (6.6-24.1) | 19.2  (11.0-32.3) | | 18.1  (10.3-30.4) | | |
| **Place of residence, n (%)** |  |  |  |  | |  | | |
| Rural | 109,200 (55.9) |  | 6,106 (71.9) | 1,378 (40.9) | | 2,162 (70.9) | | |
| Urban | 86,096 (44.1) |  | 2,382 (28.1) | 1,991 (59.1) | | 887 (29.1) | | |
| **Region code, n (%)** |  |  |  |  | |  | | |
| Qingdao (Urban) | 15,017 (7.7) |  | 268 (3.2) | 217 (6.4) | | 121 (4.0) | | |
| Harbin (Urban) | 21,598 (11.1) |  | 673 (7.9) | 720 (21.4) | | 261 (8.6) | | |
| Haikou (Urban) | 10,189 (5.2) |  | 266 (3.1) | 150 (4.5) | | 189 (6.2) | | |
| Suzhou (Urban) | 21,375 (10.9) |  | 653 (7.7) | 207 (6.1) | | 131 (4.3) | | |
| Liuzhou (Urban) | 17,917 (9.2) |  | 522 (6.1) | 697 (20.7) | | 185 (6.1) | | |
| Sichuan (Rural) | 19,604 (10.0) |  | 1,065 (12.5) | 293 (8.7) | | 353 (11.6) | | |
| Gansu (Rural) | 17,650 (9.0) |  | 1,176 (13.9) | 159 (4.7) | | 313 (10.3) | | |
| Henan (Rural) | 2,5748 (13.2) |  | 1,551 (18.3) | 233 (6.9) | | 309 (10.1) | | |
| Zhejiang (Rural) | 22,538 (11.5) |  | 903 (10.6) | 229 (6.8) | | 357 (11.7) | | |
| Hunan (Rural) | 23,660 (12.1) |  | 1,411 (16.6) | 464 (13.8) | | 830 (27.2) | | |
| **Highest education, n (%)** |  |  |  |  | |  | | |
| Primary school and lower | 79,599 (40.8) |  | 6,104 (71.9) | 1,104 (32.8) | | 1,968 (64.5) | | |
| Middle or high school | 99,931 (51.2) |  | 2,123 (25.0) | 1,943 (57.7) | | 893 (29.3) | | |
| College and higher | 15,766 (8.1) |  | 261 (3.1) | 322 (9.6) | | 188 (6.2) | | |
| **Household income (RMB/year), n (%)** |  |  |  |  | |  | | |
| <10,000 | 47,052 (24.1) |  | 4,143 (48.8) | 1,515 (45.0) | | 2,013 (66.0) | | |
| 10,000~19,999 | 55,559 (28.4) |  | 2,380 (28.0) | 1,042 (30.9) | | 563 (18.5) | | |
| ≥20,000 | 92,685 (47.5) |  | 1,965 (23.2) | 812 (24.1) | | 473 (15.5) | | |
| **Household size, n (%)** |  |  |  |  | |  | | |
| 1~2 | 134,847 (69.0) |  | 4,403 (51.9) | 1,060 (31.5) | | 879 (28.8) | | |
| 3~5 | 35,031 (17.9) |  | 3,393 (40.0) | 2,230 (66.2) | | 1,990 (65.3) | | |
| ≥6 | 25,418 (13.0) |  | 692 (8.2) | 79 (2.3) | | 180 (5.9) | | |
| **Alcohol drinking, n (%)** |  |  |  |  | |  | | |
| Not daily | 137,883 (70.6) |  | 5,921 (69.8) | 2,355 (69.9) | | 2,371 (77.8) | | |
| Daily (pure alcohol per day) |  |  |  |  | |  | | |
| 1-14 g/d | 1,700 (0.9) |  | 87 (1.0) | 19 (0.6) | | 10 (0.3) | | |
| 15-29 g/d | 7,575 (3.9) |  | 281 (3.3) | 128 (3.8) | | 66 (2.2) | | |
| 30-59 g/d | 13,450 (6.9) |  | 540 (6.4) | 230 (6.8) | | 159 (5.2) | | |
| Ex-drinker or ≥60 g/d | 34,688 (17.8) |  | 1,659 (19.5) | 637 (18.9) | | 443 (14.5) | | |
| **Smoking, n (%)** |  |  |  |  | |  | | |
| Never/occasional smoker | 50,046 (25.6) |  | 2,072 (24.4) | 781 (23.2) | | 1,017 (33.4) | | |
| Former smoker | 13,198 (6.8) |  | 606 (7.1) | 175 (5.2) | | 100 (3.3) | | |
| Current smoker (cigarette or equivalent per day) |  |  |  |  | |  | | |
| 1-9 | 23,032 (11.8) |  | 1,547 (18.2) | 356 (10.6) | | 444 (14.6) | | |
| 10-19 | 35,400 (18.1) |  | 1,568 (18.5) | 582 (17.3) | | 484 (15.9) | | |
| ≥20 | 73,620 (37.7) |  | 2,695 (31.8) | 1,475 (43.8) | | 1,004 (32.9) | | |
| **Healthy dietary habit, n (%)*** |  |  |  |  | |  | | |
| Unhealthy | 181,190 (92.8) |  | 8,108 (95.5) | 3,172 (94.2) | | 2,950 (96.8) | | |
| Healthy | 14,106 (7.2) |  | 380 (4.5) | 197 (5.8) | | 99 (3.2) | | |
| **Life satisfaction, n (%)** |  |  |  |  | |  | | |
| Very satisfied | 34,644 (17.7) |  | 1,627 (19.2) | 261 (7.7) | | 289 (9.5) | | |
| Satisfied | 103,179 (52.8) |  | 4,096 (48.3) | 1,313 (39.0) | | 1,098 (36.0) | | |
| Neither satisfied nor dissatisfied | 49,453 (25.3) |  | 2,346 (27.6) | 1,272 (37.8) | | 1,292 (42.4) | | |
| Dissatisfied | 7,605 (3.9) |  | 383 (4.5) | 466 (13.8) | | 331 (10.9) | | |
| Very dissatisfied | 415 (0.2) |  | 36 (0.4) | 57 (1.7) | | 39 (1.3) | | |
| **Prevalent diseases at baseline, n (%)** |  |  |  |  | |  | | |
| Diabetes | 10,864 (5.6) |  | 516 (6.1) | 200 (5.9) | | 103 (3.4) | | |
| Hypertension | 72,179 (37.0) |  | 4,454 (52.5) | 1,108 (32.9) | | 1,091 (35.8) | | |
| Respiratory disease† | 20,010 (10.2) |  | 1,713 (20.2) | 339 (10.1) | | 421 (13.8) | | |
| CVD | 9,220 (4.7) |  | 631 (7.4) | 143 (4.2) | | 77 (2.5) | | |
| Cancer | 881 (0.5) |  | 63 (0.7) | 18 (0.5) | | 6 (0.2) | | |

SD, standard deviation; MET, metabolic equivalent of task; IQR, interquartile range; CVD, cardiovascular disease.

***** The dietary habit was scored according to the self-reported eating habits of the participants, including eating fresh fruit per day, eating fresh vegetables per day, eating red meat 1-6 days per week, eating fish at least one day per week, and eating legumes at least 4 days per week. One or zero point for each healthy or unhealthy habit, respectively, and those with a diet score of 4 or 5 were classified into the “healthy diet” group.

† Respiratory disease includes chronic obstructive pulmonary disease/emphysema pulmonum/pulmonary heart disease, tuberculosis, and asthma.

**Table S5 Detailed baseline characteristics of women according to marital status**

|  | **With a spouse** |  | **Without a spouse** | | | |
| --- | --- | --- | --- | --- | --- | --- |
|  | **Married/**  **cohabitated** |  | **Widowed** | **Separated/**  **divorced** | **Never married** | |
| **Number of participants** | 269,177 |  | 28,069 | 4,573 | 702 |  |
| **Age, yr (SD)** | 50.3 (9.9) |  | 63.1 (8.8) | 47.1 (7.8) | 45.2 (9.2) |  |
| **Body-mass index, kg/m^2^ (SD)** | 23.8 (3.4) |  | 23.8 (3.7) | 23.5 (3.3) | 22.9 (3.6) |  |
| **Physical activity, MET-hours/day (IQR)** | 17.6  (11.2-29.1) |  | 11.2  (8.4-17.8) | 16.8  (9.8-26.9) | 14.1  (8.4-22.3) |  |
| **Place of residence, n (%)** |  |  |  |  |  |  |
| Rural | 153,248 (56.9) |  | 13,840 (49.3) | 522 (11.4) | 75 (10.7) |  |
| Urban | 115,929 (43.1) |  | 14,229 (50.7) | 4,051 (88.6) | 627 (89.3) |  |
| **Region code, n (%)** |  |  |  |  |  |  |
| Qingdao (Urban) | 17,798 (6.6) |  | 1,740 (6.2) | 312 (6.8) | 34 (4.8) |  |
| Harbin (Urban) | 28,424 (10.6) |  | 4,170 (14.9) | 1,447 (31.6) | 263 (37.5) |  |
| Haikou (Urban) | 16,323 (6.1) |  | 2,069 (7.4) | 354 (7.7) | 146 (20.8) |  |
| Suzhou (Urban) | 28,020 (10.4) |  | 2,423 (8.6) | 434 (9.5) | 26 (3.7) |  |
| Liuzhou (Urban) | 25,364 (9.4) |  | 3,827 (13.6) | 1,504 (32.9) | 158 (22.5) |  |
| Sichuan (Rural) | 30,921 (11.5) |  | 3,201 (11.4) | 225 (4.9) | 24 (3.4) |  |
| Gansu (Rural) | 28,078 (10.4) |  | 2,447 (8.7) | 51 (1.1) | 13 (1.9) |  |
| Henan (Rural) | 32,752 (12.2) |  | 2,728 (9.7) | 28 (0.6) | 7 (1.0) |  |
| Zhejiang (Rural) | 30,986 (11.5) |  | 2,567 (9.1) | 104 (2.3) | 20 (2.8) |  |
| Hunan (Rural) | 30,511 (11.3) |  | 2,897 (10.3) | 114 (2.5) | 11 (1.6) |  |
| **Highest education, n (%)** |  |  |  |  |  |  |
| Primary school and lower | 149,241 (55.4) |  | 21,490 (76.6) | 758 (16.6) | 101 (14.4) |  |
| Middle or high school | 107,902 (40.1) |  | 6,015 (21.4) | 3,174 (69.4) | 401 (57.1) |  |
| College and higher | 12,034 (4.5) |  | 564 (2.0) | 641 (14.0) | 200 (28.5) |  |
| **Household income (RMB/year), n (%)** |  |  |  |  |  |  |
| <10,000 | 74,176 (27.6) |  | 13,761 (49.0) | 1,859 (40.7) | 216 (30.8) |  |
| 10,000~19,999 | 80,138 (29.8) |  | 7,535 (26.8) | 1,505 (32.9) | 236 (33.6) |  |
| ≥20,000 | 114,863 (42.7) |  | 6,773 (24.1) | 1,209 (26.4) | 250 (35.6) |  |
| **Household size, n (%)** |  |  |  |  |  |  |
| 1~2 | 188,686 (70.1) |  | 14,530 (51.8) | 1,206 (26.4) | 294 (41.9) |  |
| 3~5 | 45,867 (17.0) |  | 11,758 (41.9) | 3,298 (72.1) | 359 (51.1) |  |
| ≥6 | 34,624 (12.9) |  | 1,781 (6.3) | 69 (1.5) | 49 (7.0) |  |
| **Alcohol drinking, n (%)** |  |  |  |  |  |  |
| Not daily | 264,630 (98.3) |  | 27,242 (97.1) | 4,480 (98.0) | 685 (97.6) |  |
| Daily (pure alcohol per day) |  |  |  |  |  |  |
| 1-14 g/d | 672 (0.2) |  | 117 (0.4) | 16 (0.3) | 2 (0.3) |  |
| 15-29 g/d | 807 (0.3) |  | 135 (0.5) | 10 (0.2) | 3 (0.4) |  |
| 30-59 g/d | 582 (0.2) |  | 103 (0.4) | 7 (0.2) | 3 (0.4) |  |
| Ex-drinker or ≥60 g/d | 2,486 (0.9) |  | 472 (1.7) | 60 (1.3) | 9 (1.3) |  |
| **Smoking, n (%)** |  |  |  |  |  |  |
| Never/occasional smoker | 261,995 (97.3) |  | 25,712 (91.6) | 4,340 (94.9) | 677 (96.4) |  |
| Former smoker | 828 (0.3) |  | 346 (1.2) | 27 (0.6) | 0 (0.0) |  |
| Current smoker (cigarette or equivalent per day) |  |  |  |  |  |  |
| 1-9 | 3,488 (1.3) |  | 1,121 (4.0) | 83 (1.8) | 8 (1.1) |  |
| 10-19 | 1,810 (0.7) |  | 570 (2.0) | 75 (1.6) | 10 (1.4) |  |
| ≥20 | 1,056 (0.4) |  | 320 (1.1) | 48 (1.0) | 7 (1.0) |  |
| **Healthy dietary habit, n (%)*** |  |  |  |  |  |  |
| Unhealthy | 244,580 (90.9) |  | 25,952 (92.5) | 3,853 (84.3) | 599 (85.3) |  |
| Healthy | 24,597 (9.1) |  | 2,117 (7.5) | 720 (15.7) | 103 (14.7) |  |
| **Life satisfaction, n (%)** |  |  |  |  |  |  |
| Very satisfied | 48,457 (18.0) |  | 4,283 (15.3) | 268 (5.9) | 47 (6.7) |  |
| Satisfied | 136,255 (50.6) |  | 13,281 (47.3) | 1,967 (43.0) | 339 (48.3) |  |
| Neither satisfied nor dissatisfied | 74,920 (27.8) |  | 8,797 (31.3) | 1,659 (36.3) | 248 (35.3) |  |
| Dissatisfied | 9,025 (3.4) |  | 1,567 (5.6) | 618 (13.5) | 61 (8.7) |  |
| Very dissatisfied | 520 (0.2) |  | 141 (0.5) | 61 (1.3) | 7 (1.0) |  |
| **Prevalent diseases at baseline, n (%)** |  |  |  |  |  |  |
| Diabetes | 15,432 (5.7) |  | 2,930 (10.4) | 223 (4.9) | 32 (4.6) |  |
| Hypertension | 86,214 (32.0) |  | 14,589 (52.0) | 825 (18.0) | 128 (18.2) |  |
| Respiratory disease† | 18,681 (6.9) |  | 3,478 (12.4) | 318 (7.0) | 46 (6.6) |  |
| CVD | 10,357 (3.8) |  | 2,529 (9.0) | 154 (3.4) | 18 (2.6) |  |
| Cancer | 1,375 (0.5) |  | 194 (0.7) | 35 (0.8) | 6 (0.9) |  |
| **Menopausal status, n (%)** |  |  |  |  |  |  |
| Premenopausal status | 123,320 (45.8) |  | 2,176 (7.8) | 2,760 (60.4) | 465 (66.2) |  |
| Currently in menopause | 13,874 (5.2) |  | 579 (2.1) | 341 (7.5) | 34 (4.8) |  |
| Postmenopausal status | 131,939 (49.0) |  | 25,312 (90.2) | 1,471 (32.2) | 203 (28.9) |  |
| Missing | 44 (0.0) |  | 2 (0.0) | 1 (0.0) | 0 (0.0) |  |

SD, standard deviation; MET, metabolic equivalent of task; IQR, interquartile range; CVD, cardiovascular disease.

* The dietary habit was scored according to the self-reported eating habits of the participants, including eating fresh fruit per day, eating fresh vegetables per day, eating red meat 1-6 days per week, eating fish at least one day per week, and eating legumes at least 4 days per week. One or zero point for each healthy or unhealthy habit, respectively, and those with a diet score of 4 or 5 were classified into the “healthy diet” group.

† Respiratory disease includes chronic obstructive pulmonary disease/emphysema pulmonum/pulmonary heart disease, tuberculosis, and asthma.

**Table S6 PheWAS results of** **marital status (living with vs. without a spouse) with 504 diseases in 210,202 men**

| **Phecode** | **Diseases** | **Group** | **Case/****control** | **Model 1** | |  | **Model 2** | |  | **Model 3** | |
| --- | --- | --- | --- | --- | --- | --- | --- | --- | --- | --- | --- |
|  |  |  |  | **OR (95%CI)** | ***P*** |  | **OR (95%CI)** | ***P*** |  | **OR (95%CI)** | ***P*** |
| 295 | Schizophrenia and other psychotic disorders | mental disorders | 862/170,474 | 3.82 (3.19, 4.59) | 2.83E-47 |  | 3.69 (3.07, 4.43) | 1.68E-44 |  | 3.46 (3.27-3.64) | 5.87E-39 |
| 295.1 | Schizophrenia | mental disorders | 859/170,474 | 3.82 (3.19, 4.59) | 4.68E-47 |  | 3.69 (3.07, 4.43) | 2.63E-44 |  | 3.46 (3.27-3.64) | 9.10E-39 |
| 496.2 | Chronic bronchitis | respiratory | 230,88/143,409 | 1.16 (1.10, 1.22) | 4.02E-09 |  | 1.12 (1.06, 1.18) | 1.22E-05 |  | 1.12 (1.03-1.20) | 0.0111 |
| 496 | Chronic airway obstruction | respiratory | 24,541/143,409 | 1.15 (1.10, 1.21) | 8.90E-09 |  | 1.00 (1.06, 1.17) | 2.72E-05 |  | 1.10 (1.02-1.17) | 0.0220 |
| 496.21 | Obstructive chronic bronchitis | respiratory | 21,041/143,409 | 1.16 (1.10, 1.22) | 1.03E-08 |  | 1.12 (1.06, 1.18) | 2.65E-05 |  | 1.10 (1.00-1.20) | 0.0588 |
| 430.2 | Intracerebral hemorrhage | circulatory system | 5,685/135,883 | 1.26 (1.16, 1.37) | 4.60E-08 |  | 1.28 (1.18, 1.39) | 6.25E-09 |  | 1.21 (1.12-1.29) | 1.23E-05 |
| 401 | Hypertension | circulatory system | 84,556/87,647 | 1.11 (1.07, 1.15) | 2.59E-07 |  | 1.21 (1.16, 1.26) | 3.56E-20 |  | 0.93 (0.82-1.05) | 0.2420 |
| 430 | Intracranial hemorrhage | circulatory system | 6,200/135,883 | 1.23 (1.14, 1.34) | 3.09E-07 |  | 1.25 (1.15, 1.35) | 5.42E-08 |  | 1.18 (1.10-1.26) | 6.36E-05 |
| 401.1 | Essential hypertension | circulatory system | 84,414/87,647 | 1.11 (1.07, 1.15) | 3.31E-07 |  | 1.21 (1.16, 1.26) | 5.76E-20 |  | 0.92 (0.80-1.03) | 0.1391 |
| 433.3 | Cerebral ischemia | circulatory system | 12,237/135,883 | 0.83 (0.77, 0.90) | 2.47E-06 |  | 0.85 (0.79, 0.92) | 3.27E-05 |  | 0.85 (0.78-0.93) | 5.06E-05 |
| 574 | Cholelithiasis and cholecystitis | digestive | 12,767/158,889 | 0.83 (0.77, 0.90) | 3.14E-06 |  | 0.85 (0.78, 0.92) | 3.30E-05 |  | 0.86 (0.78-0.94) | 0.0002 |
| 760 | Back pain | symptoms | 5,845/166,358 | 0.76 (0.68, 0.86) | 1.55E-05 |  | 0.77 (0.68, 0.87) | 3.23E-05 |  | 0.78 (0.65-0.90) | 5.64E-05 |
| 574.1 | Cholelithiasis | digestive | 10,478/158,889 | 0.84 (0.77, 0.91) | 5.14E-05 |  | 0.86 (0.78, 0.93) | 0.0004 |  | 0.87 (0.78-0.95) | 0.0013 |
| 819 | Skull and face fracture and other intercranial injury | injuries & poisonings | 3,597/168,416 | 1.27 (1.12, 1.44) | 0.0001 |  | 1.28 (1.13, 1.45) | 9.39E-05 |  | 1.26 (1.14-1.39) | 0.0002 |
| 433.31 | Transient cerebral ischemia | circulatory system | 1,078/171,119 | 0.60 (0.45, 0.79) | 0.0003 |  | 0.58 (0.44, 0.77) | 0.001 |  | 0.58 (0.30-0.87) | 0.0002 |
| 512.8 | Cough | respiratory | 3,409/135,883 | 0.76 (0.66, 0.88) | 0.0003 |  | 0.78 (0.67, 0.90) | 0.0002 |  | 0.79 (0.64-0.94) | 0.0018 |
| 465 | Acute upper respiratory infections of multiple or unspecified sites | respiratory | 17,848/154,332 | 0.87 (0.81, 0.94) | 0.0006 |  | 0.87 (0.80, 0.94) | 0.0003 |  | 0.88 (0.81-0.96) | 0.0021 |
| 512 | Other symptoms of respiratory system | respiratory | 1,084/171,119 | 0.61 (0.47, 0.81) | 0.0006 |  | 0.60 (0.46, 0.79) | 0.0003 |  | 0.60 (0.32-0.88) | 0.0003 |
| 594 | Urinary calculus | genitourinary | 6,224/165,873 | 0.83 (0.74, 0.92) | 0.0007 |  | 0.84 (0.76, 0.94) | 0.0022 |  | 0.87 (0.76-0.98) | 0.0115 |
| 594.3 | Calculus of ureter | genitourinary | 2,549/165,873 | 0.73 (0.61, 0.88) | 0.0009 |  | 0.75 (0.62, 0.90) | 0.0019 |  | 0.78 (0.59-0.96) | 0.0085 |
| 411.4 | Coronary atherosclerosis | circulatory system | 16,072/149,240 | 0.90 (0.85, 0.96) | 0.0013 |  | 0.92 (0.87, 0.98) | 0.0124 |  | 0.94 (0.88-1.01) | 0.0765 |
| 590 | Pyelonephritis | genitourinary | 3,181/166,019 | 0.78 (0.66, 0.91) | 0.0014 |  | 0.78 (0.67, 0.91) | 0.0019 |  | 0.78 (0.62-0.94) | 0.0019 |
| 276 | Disorders of fluid, electrolyte, and acid-base balance | endocrine/  metabolic | 687/171,504 | 1.45 (1.15, 1.82) | 0.0017 |  | 1.44 (1.14, 1.81) | 0.002 |  | 1.47 (1.24-1.70) | 0.0011 |
| 276.1 | Electrolyte imbalance | endocrine/  metabolic | 687/171,504 | 1.45 (1.15, 1.82) | 0.0017 |  | 1.44 (1.14, 1.81) | 0.002 |  | 1.47 (1.24-1.70) | 0.0011 |
| 591 | Urinary tract infection | genitourinary | 1,493/166,019 | 0.71 (0.57, 0.88) | 0.0021 |  | 0.72 (0.58, 0.90) | 0.0032 |  | 0.75 (0.53-0.96) | 0.0083 |
| 300 | Anxiety disorders | mental disorders | 735/170,474 | 1.46 (1.14, 1.86) | 0.0027 |  | 1.44 (1.13, 1.84) | 0.0034 |  | 1.40 (1.16-1.65) | 0.0072 |
| 415 | Pulmonary heart disease | circulatory system | 1,432/170,711 | 1.24 (1.07, 1.45) | 0.0044 |  | 1.20 (1.03, 1.39) | 0.0193 |  | 1.17 (1.01-1.32) | 0.0488 |
| 531 | Peptic ulcer (excl. esophageal) | digestive | 13,400/158,803 | 0.90 (0.83, 0.97) | 0.0046 |  | 0.86 (0.80, 0.93) | 8.18E-05 |  | 0.87 (0.80-0.95) | 0.0004 |
| 415.2 | Chronic pulmonary heart disease | circulatory system | 1,416/170,711 | 1.24 (1.07, 1.45) | 0.0048 |  | 1.20 (1.03, 1.39) | 0.0206 |  | 1.17 (1.01-1.32) | 0.0516 |
| 803.2 | Fracture of radius and ulna | injuries & poisonings | 422/168,201 | 0.50 (0.30, 0.83) | 0.0073 |  | 0.50 (0.30, 0.84) | 0.0083 |  | 0.52 (0.00-1.03) | 0.0118 |
| 433 | Cerebrovascular disease | circulatory system | 32,281/135,883 | 0.94 (0.89, 0.98) | 0.008 |  | 0.96 (0.91, 1.01) | 0.0891 |  | 0.96 (0.91-1.01) | 0.0902 |
| 531.4 | Peptic ulcer, site unspecified | digestive | 317/158,803 | 1.57 (1.12, 2.21) | 0.0097 |  | 1.52 (1.08, 2.14) | 0.0162 |  | 1.53 (1.19-1.88) | 0.0148 |
| 372 | Disorders of conjunctiva | sense organs | 967/169,380 | 0.71 (0.54, 0.93) | 0.0123 |  | 0.71 (0.54, 0.93) | 0.0126 |  | 0.73 (0.46-1.00) | 0.0223 |
| 455 | Hemorrhoids | circulatory system | 3,559/167,579 | 0.82 (0.71, 0.96) | 0.0137 |  | 0.82 (0.70, 0.95) | 0.0102 |  | 0.84 (0.69-1.00) | 0.0277 |
| 189.1 | Cancer of kidney and renal pelvis | neoplasms | 263/171,450 | 0.43 (0.22, 0.85) | 0.0154 |  | 0.45 (0.23, 0.88) | 0.0193 |  | 0.46 (-0.22-1.13) | 0.0225 |
| 475 | Chronic sinusitis | respiratory | 554/168,697 | 0.56 (0.35, 0.90) | 0.0156 |  | 0.57 (0.35, 0.90) | 0.0175 |  | 0.60 (0.13-1.07) | 0.0356 |
| 276.14 | Hypopotassemia | endocrine/  metabolic | 500/171,504 | 1.40 (1.06, 1.85) | 0.0162 |  | 1.40 (1.07, 1.85) | 0.0158 |  | 1.42 (1.15-1.70) | 0.0126 |
| 565 | Anal and rectal conditions | digestive | 1,575/170,043 | 0.74 (0.58, 0.95) | 0.0167 |  | 0.77 (0.60, 0.98) | 0.0328 |  | 0.78 (0.53-1.03) | 0.0474 |
| 189.11 | Malignant neoplasm of kidney, except pelvis | neoplasms | 248/171,450 | 0.42 (0.21, 0.86) | 0.0174 |  | 0.43 (0.21, 0.88) | 0.0214 |  | 0.44 (-0.28-1.15) | 0.0239 |
| 782.3 | Edema | symptoms | 215/171,988 | 1.62 (1.08, 2.44) | 0.0191 |  | 1.66 (1.11, 2.50) | 0.014 |  | 1.67 (1.26-2.08) | 0.0137 |
| 208 | Benign neoplasm of colon | neoplasms | 1,022/169,348 | 0.68 (0.49, 0.95) | 0.0226 |  | 0.70 (0.51, 0.97) | 0.0331 |  | 0.72 (0.39-1.04) | 0.0449 |
| 519 | Other diseases of respiratory system, not elsewhere classified | respiratory | 753/171,449 | 0.67 (0.47, 0.94) | 0.0226 |  | 0.66 (0.47, 0.94) | 0.0208 |  | 0.67 (0.32-1.02) | 0.0246 |
| 519.8 | Other diseases of respiratory system, NEC | respiratory | 754/171,449 | 0.67 (0.47, 0.94) | 0.0226 |  | 0.66 (0.47, 0.94) | 0.0208 |  | 0.67 (0.32-1.02) | 0.0246 |
| 327.4 | Insomnia | neurological | 293/171,515 | 0.51 (0.29, 0.91) | 0.0227 |  | 0.51 (0.29, 0.91) | 0.0224 |  | 0.53 (-0.04-1.11) | 0.0312 |
| 420.1 | Myocarditis | circulatory system | 217/171,261 | 1.68 (1.07, 2.63) | 0.0232 |  | 1.64 (1.05, 2.56) | 0.0311 |  | 1.64 (1.19-2.09) | 0.0318 |
| 345 | Epilepsy, recurrent seizures, convulsions | neurological | 382/170,510 | 1.46 (1.05, 2.01) | 0.0233 |  | 1.43 (1.04, 1.98) | 0.0301 |  | 1.44 (1.11-1.76) | 0.0297 |
| 535 | Gastritis and duodenitis | digestive | 11,350/159,961 | 0.91 (0.85, 0.99) | 0.0258 |  | 0.90 (0.83, 0.97) | 0.0077 |  | 0.91 (0.83-0.99) | 0.0265 |
| 272 | Disorders of lipoid metabolism | endocrine/  metabolic | 1,599/170,604 | 0.78 (0.62, 0.97) | 0.0289 |  | 0.82 (0.65, 1.03) | 0.0874 |  | 0.85 (0.62-1.08) | 0.1543 |
| 272.1 | Hyperlipidemia | endocrine/  metabolic | 1,599/170,604 | 0.78 (0.62, 0.97) | 0.0289 |  | 0.82 (0.65, 1.03) | 0.0874 |  | 0.85 (0.62-1.08) | 0.1543 |
| 472 | Chronic pharyngitis and nasopharyngitis | respiratory | 753/168,697 | 0.66 (0.45, 0.97) | 0.0324 |  | 0.66 (0.45, 0.96) | 0.0309 |  | 0.67 (0.29-1.05) | 0.0406 |
| 389 | Hearing loss | sense organs | 241/171,962 | 0.41 (0.18, 0.93) | 0.0339 |  | 0.42 (0.18, 0.96) | 0.0395 |  | 0.43 (-0.39-1.26) | 0.0469 |
| 395.2 | Nonrheumatic aortic valve disorders | circulatory system | 54/171,662 | 2.33 (1.06, 5.15) | 0.0364 |  | 2.34 (1.06, 5.19) | 0.0356 |  | 2.43 (1.63-3.23) | 0.0293 |
| 465.2 | Acute pharyngitis | respiratory | 3,093/154,332 | 0.83 (0.70, 0.99) | 0.0364 |  | 0.83 (0.70, 0.98) | 0.0293 |  | 0.87 (0.70-1.04) | 0.1083 |
| 53 | Herpes zoster | infectious diseases | 1,198/169,432 | 0.79 (0.62, 0.99) | 0.04 |  | 0.78 (0.62, 0.98) | 0.0336 |  | 0.78 (0.55-1.01) | 0.0366 |
| 474 | Acute and chronic tonsillitis | respiratory | 923/168,697 | 0.72 (0.53, 0.99) | 0.0406 |  | 0.74 (0.54, 1.01) | 0.0564 |  | 0.74 (0.43-1.06) | 0.0634 |
| 497 | Bronchitis | respiratory | 5,661/143,409 | 0.89 (0.80, 1.00) | 0.0419 |  | 0.87 (0.78, 0.97) | 0.0108 |  | 0.87 (0.75-0.98) | 0.0146 |
| 250 | Diabetes mellitus | endocrine/  metabolic | 15,711/156,334 | 0.93 (0.87, 1.00) | 0.047 |  | 0.98 (0.92, 1.05) | 0.6563 |  | 0.97 (0.84-1.11) | 0.7089 |
| 411 | Ischemic Heart Disease | circulatory system | 22,322/149,240 | 0.95 (0.90, 1.00) | 0.0472 |  | 0.97 (0.92, 1.02) | 0.2373 |  | 1.00 (0.94-1.05) | 0.9060 |
| 250.2 | Type 2 diabetes | endocrine/  metabolic | 15,655/156,334 | 0.93 (0.87, 1.00) | 0.0475 |  | 0.98 (0.92, 1.05) | 0.6545 |  | 0.97 (0.83-1.11) | 0.6903 |
| 394.2 | Mitral valve disease | circulatory system | 236/171,662 | 1.53 (1.00, 2.35) | 0.0499 |  | 1.52 (0.99, 2.33) | 0.0555 |  | 1.47 (1.04-1.90) | 0.0796 |
| 579 | Other symptoms involving abdomen and pelvis | digestive | 174/169,670 | 0.47 (0.22, 1.01) | 0.0521 |  | 0.47 (0.22, 1.01) | 0.0546 |  | 0.47 (-0.30-1.24) | 0.0545 |
| 495 | Asthma | respiratory | 1,221/143,409 | 1.21 (1.00, 1.48) | 0.0527 |  | 1.16 (0.95, 1.41) | 0.1353 |  | 1.08 (0.82-1.33) | 0.5706 |
| 344 | Other paralytic syndromes | neurological | 67/170,510 | 0.31 (0.10, 1.02) | 0.0542 |  | 0.32 (0.10, 1.06) | 0.0626 |  | 0.28 (-0.93-1.48) | 0.0359 |
| 740 | Osteoarthrosis | musculoskeletal | 5,691/166,512 | 0.90 (0.81, 1.00) | 0.0543 |  | 0.92 (0.82, 1.02) | 0.1183 |  | 0.93 (0.82-1.03) | 0.1619 |
| 536 | Disorders of function of stomach | digestive | 981/159,961 | 0.78 (0.60, 1.00) | 0.0544 |  | 0.78 (0.60, 1.01) | 0.0601 |  | 0.80 (0.54-1.06) | 0.0900 |
| 536.8 | Dyspepsia and other specified disorders of function of stomach | digestive | 981/159,961 | 0.78 (0.60, 1.00) | 0.0544 |  | 0.78 (0.60, 1.01) | 0.0601 |  | 0.80 (0.54-1.06) | 0.0900 |
| 474.1 | Acute tonsillitis | respiratory | 596/168,697 | 0.70 (0.48, 1.02) | 0.0648 |  | 0.72 (0.49, 1.05) | 0.0867 |  | 0.72 (0.34-1.10) | 0.0865 |
| 740.9 | Osteoarthrosis NOS | musculoskeletal | 5,332/166,512 | 0.90 (0.80, 1.01) | 0.0649 |  | 0.92 (0.82, 1.02) | 0.1231 |  | 0.92 (0.81-1.04) | 0.1687 |
| 480 | Pneumonia | respiratory | 12,956/156,992 | 0.94 (0.88, 1.00) | 0.0687 |  | 0.93 (0.87, 0.99) | 0.021 |  | 0.94 (0.87-1.00) | 0.0541 |
| 280 | Iron deficiency anemias | hematopoietic | 190/171,112 | 1.50 (0.97, 2.32) | 0.0694 |  | 1.47 (0.95, 2.27) | 0.0876 |  | 1.50 (1.06-1.94) | 0.0729 |
| 707 | Chronic ulcer of skin | dermatologic | 70/172,133 | 0.35 (0.11, 1.11) | 0.0746 |  | 0.35 (0.11, 1.13) | 0.0786 |  | 0.35 (-0.82-1.52) | 0.0807 |
| 707.1 | Decubitus ulcer | dermatologic | 70/172,133 | 0.35 (0.11, 1.11) | 0.0746 |  | 0.35 (0.11, 1.13) | 0.0786 |  | 0.35 (-0.82-1.52) | 0.0807 |
| 401.21 | Hypertensive heart disease | circulatory system | 888/87,647 | 1.21 (0.98, 1.49) | 0.0765 |  | 1.25 (1.01, 1.54) | 0.0406 |  | 1.55 (1.10-2.01) | 0.0584 |
| 323 | Encephalitis | neurological | 43/172,136 | 2.29 (0.91, 5.76) | 0.0787 |  | 2.25 (0.89, 5.69) | 0.0854 |  | 2.43 (1.50-3.36) | 0.0613 |
| 428.1 | Congestive heart failure (CHF) NOS | circulatory system | 1,708/170,345 | 1.14 (0.98, 1.33) | 0.0807 |  | 1.14 (0.98, 1.33) | 0.0799 |  | 1.13 (0.98-1.29) | 0.0999 |
| 687 | Symptoms affecting skin | dermatologic | 69/172,133 | 0.17 (0.02, 1.26) | 0.0833 |  | 0.17 (0.02, 1.26) | 0.0833 |  | 0.17 (-1.82-2.16) | 0.0831 |
| 371.3 | Inflammation of eyelids | sense organs | 91/169,380 | 0.28 (0.07, 1.19) | 0.0844 |  | 0.28 (0.07, 1.17) | 0.0814 |  | 0.29 (-1.15-1.72) | 0.0885 |
| 420 | Carditis | circulatory system | 519/171,261 | 1.29 (0.96, 1.72) | 0.0857 |  | 1.27 (0.95, 1.70) | 0.1032 |  | 1.28 (0.98-1.57) | 0.1012 |
| 274.11 | Gouty arthropathy | endocrine/  metabolic | 449/170,985 | 0.71 (0.48, 1.05) | 0.0877 |  | 0.77 (0.52, 1.14) | 0.1949 |  | 0.75 (0.36-1.14) | 0.1490 |
| 571.8 | Liver abscess and sequelae of chronic liver disease | digestive | 218/170,077 | 1.50 (0.94, 2.41) | 0.089 |  | 1.54 (0.96, 2.47) | 0.0736 |  | 1.59 (1.12-2.07) | 0.0537 |
| 290 | Delirium dementia and amnestic and other cognitive disorders | mental disorders | 272/171,928 | 1.32 (0.96, 1.82) | 0.0912 |  | 1.32 (0.96, 1.82) | 0.0889 |  | 1.34 (1.02-1.66) | 0.0762 |
| 594.2 | Calculus of lower urinary tract | genitourinary | 541/165,873 | 0.74 (0.52, 1.05) | 0.0947 |  | 0.75 (0.53, 1.07) | 0.1094 |  | 0.76 (0.41-1.11) | 0.1215 |
| 153.3 | Malignant neoplasm of rectum, rectosigmoid junction, and anus | neoplasms | 1,061/162,522 | 0.81 (0.64, 1.04) | 0.0966 |  | 0.82 (0.64, 1.04) | 0.1045 |  | 0.82 (0.57-1.06) | 0.1100 |
| 480.11 | Pneumococcal pneumonia | respiratory | 216/156,992 | 0.54 (0.26, 1.12) | 0.0974 |  | 0.52 (0.25, 1.07) | 0.076 |  | 0.51 (-0.22-1.25) | 0.0746 |
| 365 | Glaucoma | sense organs | 556/171,122 | 0.74 (0.52, 1.06) | 0.0979 |  | 0.74 (0.52, 1.05) | 0.0956 |  | 0.75 (0.40-1.11) | 0.1138 |
| 293.1 | Swelling, mass, or lump in head and neck [Space-occupying lesion, intracranial NOS] | mental disorders | 190/171,998 | 0.55 (0.27, 1.13) | 0.104 |  | 0.55 (0.27, 1.14) | 0.1097 |  | 0.55 (-0.18-1.28) | 0.1065 |
| 574.3 | Cholecystitis without cholelithiasis | digestive | 2,968/158,889 | 0.89 (0.77, 1.03) | 0.1059 |  | 0.90 (0.78, 1.04) | 0.152 |  | 0.91 (0.77-1.06) | 0.2311 |
| 454 | Varicose veins | circulatory system | 1,078/167,579 | 0.79 (0.59, 1.05) | 0.1064 |  | 0.81 (0.60, 1.08) | 0.1443 |  | 0.82 (0.53-1.11) | 0.1748 |
| 540 | Appendiceal conditions | digestive | 923/171,280 | 0.78 (0.58, 1.05) | 0.1074 |  | 0.79 (0.59, 1.06) | 0.1119 |  | 0.80 (0.50-1.10) | 0.1403 |
| 303.1 | Dissociative disorder | mental disorders | 44/170,474 | 2.13 (0.85, 5.38) | 0.1081 |  | 1.99 (0.79, 5.05) | 0.1453 |  | 1.86 (0.92-2.80) | 0.1961 |
| 585.1 | Acute renal failure | genitourinary | 70/166,533 | 1.73 (0.88, 3.41) | 0.111 |  | 1.75 (0.89, 3.46) | 0.1043 |  | 1.82 (1.14-2.51) | 0.0833 |
| 339 | Other headache syndromes | neurological | 2945/169,121 | 0.89 (0.76, 1.03) | 0.112 |  | 0.89 (0.77, 1.04) | 0.1336 |  | 0.91 (0.76-1.06) | 0.2368 |
| 458.9 | Hypotension NOS | circulatory system | 354/171,843 | 1.35 (0.93, 1.97) | 0.1121 |  | 1.30 (0.89, 1.89) | 0.1735 |  | 1.35 (0.98-1.73) | 0.1155 |
| 189 | Cancer of urinary organs (incl. kidney and bladder) | neoplasms | 753/171,450 | 0.79 (0.58, 1.06) | 0.1129 |  | 0.79 (0.59, 1.07) | 0.1254 |  | 0.80 (0.50-1.10) | 0.1393 |
| 565.1 | Anal and rectal polyp | digestive | 273/170,043 | 0.59 (0.31, 1.13) | 0.1143 |  | 0.60 (0.31, 1.15) | 0.1221 |  | 0.61 (-0.04-1.26) | 0.1365 |
| 296 | Mood disorders | mental disorders | 224/170,474 | 1.47 (0.91, 2.39) | 0.1155 |  | 1.46 (0.90, 2.37) | 0.124 |  | 1.40 (0.92-1.89) | 0.1738 |
| 530.14 | Reflux esophagitis | digestive | 248/171,639 | 0.65 (0.38, 1.11) | 0.1156 |  | 0.66 (0.39, 1.13) | 0.1327 |  | 0.70 (0.16-1.24) | 0.1968 |
| 560.4 | Other intestinal obstruction | digestive | 1,362/170,358 | 0.85 (0.70, 1.04) | 0.1188 |  | 0.84 (0.68, 1.02) | 0.0834 |  | 0.84 (0.64-1.05) | 0.0980 |
| 788 | Syncope and collapse | symptoms | 196/172,007 | 0.62 (0.34, 1.13) | 0.1204 |  | 0.66 (0.36, 1.19) | 0.1652 |  | 0.66 (0.06-1.26) | 0.1725 |
| 686 | Other local infections of skin and subcutaneous tissue | dermatologic | 57/172,141 | 0.39 (0.12, 1.29) | 0.1224 |  | 0.41 (0.12, 1.35) | 0.1408 |  | 0.42 (-0.78-1.61) | 0.1504 |
| 507 | Pleurisy; pleural effusion | respiratory | 823/170,426 | 0.82 (0.63, 1.06) | 0.1234 |  | 0.80 (0.62, 1.04) | 0.0915 |  | 0.82 (0.56-1.08) | 0.1314 |
| 351 | Other peripheral nerve disorders | neurological | 212/171,360 | 1.43 (0.91, 2.26) | 0.1251 |  | 1.42 (0.90, 2.25) | 0.1305 |  | 1.44 (0.98-1.90) | 0.1223 |
| 537 | Other disorders of stomach and duodenum | digestive | 99/159,961 | 0.45 (0.16, 1.25) | 0.127 |  | 0.42 (0.15, 1.17) | 0.0973 |  | 0.42 (-0.60-1.44) | 0.0975 |
| 289.4 | Lymphadenitis | hematopoietic | 265/171,681 | 0.59 (0.30, 1.16) | 0.1278 |  | 0.59 (0.30, 1.17) | 0.1339 |  | 0.64 (-0.05-1.32) | 0.1950 |
| 414 | Other forms of chronic heart disease | circulatory system | 1,333/149,240 | 0.86 (0.70, 1.05) | 0.1316 |  | 0.87 (0.71, 1.06) | 0.1542 |  | 0.87 (0.67-1.07) | 0.1893 |
| 454.1 | Varicose veins of lower extremity | circulatory system | 1,063/167,579 | 0.80 (0.60, 1.07) | 0.1338 |  | 0.82 (0.62, 1.10) | 0.1829 |  | 0.83 (0.54-1.12) | 0.2175 |
| 395 | Heart valve disorders | circulatory system | 84/171,662 | 1.70 (0.85, 3.40) | 0.1344 |  | 1.74 (0.87, 3.48) | 0.1191 |  | 1.75 (1.05-2.45) | 0.1159 |
| 170.1 | Bone cancer | neoplasms | 173/171,958 | 1.43 (0.89, 2.30) | 0.1372 |  | 1.42 (0.88, 2.27) | 0.1513 |  | 1.44 (0.96-1.91) | 0.1369 |
| 571 | Chronic liver disease and cirrhosis | digestive | 1,208/170,077 | 1.17 (0.95, 1.45) | 0.1381 |  | 1.21 (0.98, 1.50) | 0.0748 |  | 1.25 (1.04-1.46) | 0.0407 |
| 458 | Hypotension | circulatory system | 360/171,843 | 1.33 (0.91, 1.92) | 0.1388 |  | 1.27 (0.87, 1.85) | 0.2097 |  | 1.33 (0.95-1.70) | 0.1401 |
| 225 | Benign neoplasm of brain and other parts of nervous system | neoplasms | 104/171,621 | 0.46 (0.17, 1.29) | 0.1408 |  | 0.47 (0.17, 1.30) | 0.1447 |  | 0.49 (-0.53-1.51) | 0.1716 |
| 939 | Atopic/contact dermatitis due to other or unspecified | dermatologic | 1,510/170,419 | 0.85 (0.68, 1.06) | 0.1417 |  | 0.85 (0.69, 1.06) | 0.1607 |  | 0.87 (0.65-1.09) | 0.2323 |
| 375 | Disorders of lacrimal system | sense organs | 99/171,572 | 0.47 (0.17, 1.30) | 0.1433 |  | 0.47 (0.17, 1.30) | 0.1455 |  | 0.49 (-0.54-1.52) | 0.1720 |
| 296.2 | Depression | mental disorders | 213/170,474 | 1.44 (0.88, 2.37) | 0.1471 |  | 1.43 (0.87, 2.36) | 0.1539 |  | 1.37 (0.87-1.88) | 0.2130 |
| 296.22 | Major depressive disorder | mental disorders | 213/170,474 | 1.44 (0.88, 2.37) | 0.1471 |  | 1.43 (0.87, 2.36) | 0.1539 |  | 1.37 (0.87-1.88) | 0.2130 |
| 401.2 | Hypertensive heart and/or renal disease | circulatory system | 1,027/87,647 | 1.16 (0.95, 1.41) | 0.148 |  | 1.20 (0.98, 1.47) | 0.0785 |  | 1.46 (1.02-1.89) | 0.0896 |
| 280.1 | Iron deficiency anemias, unspecified or not due to blood loss | hematopoietic | 129/171,112 | 1.50 (0.87, 2.58) | 0.1481 |  | 1.45 (0.84, 2.50) | 0.1859 |  | 1.48 (0.93-2.02) | 0.1648 |
| 560 | Intestinal obstruction without mention of hernia | digestive | 1,416/170,358 | 0.87 (0.71, 1.05) | 0.15 |  | 0.85 (0.70, 1.04) | 0.1076 |  | 0.86 (0.66-1.06) | 0.1328 |
| 244.4 | Hypothyroidism NOS | endocrine/  metabolic | 43/171,651 | 1.94 (0.78, 4.83) | 0.154 |  | 1.87 (0.75, 4.65) | 0.1777 |  | 1.84 (0.92-2.77) | 0.1939 |
| 428 | Congestive heart failure; nonhypertensive | circulatory system | 1,854/170,345 | 1.00 (0.96, 1.28) | 0.1597 |  | 1.00 (0.96, 1.29) | 0.1436 |  | 1.10 (0.96-1.25) | 0.1814 |
| 170 | Cancer of bone and connective tissue | neoplasms | 245/171,958 | 1.35 (0.89, 2.04) | 0.1601 |  | 1.32 (0.87, 2.01) | 0.1857 |  | 1.33 (0.91-1.75) | 0.1836 |
| 225.1 | Benign neoplasm of brain, cranial nerves, meninges | neoplasms | 101/171,621 | 0.48 (0.17, 1.34) | 0.1625 |  | 0.49 (0.17, 1.35) | 0.1659 |  | 0.51 (-0.51-1.53) | 0.1954 |
| 540.1 | Appendicitis | digestive | 885/171,280 | 0.81 (0.60, 1.09) | 0.1664 |  | 0.81 (0.60, 1.09) | 0.1681 |  | 0.83 (0.53-1.13) | 0.2146 |
| 575.8 | Other disorders of biliary tract | digestive | 149/158,889 | 0.60 (0.29, 1.24) | 0.1672 |  | 0.61 (0.29, 1.26) | 0.181 |  | 0.63 (-0.10-1.36) | 0.2182 |
| 244 | Hypothyroidism | endocrine/  metabolic | 44/171,651 | 1.89 (0.76, 4.69) | 0.1684 |  | 1.82 (0.73, 4.50) | 0.1972 |  | 1.79 (0.87-2.71) | 0.2161 |
| 150 | Cancer of esophagus | neoplasms | 1,972/162,514 | 1.00 (0.96, 1.27) | 0.1685 |  | 1.08 (0.94, 1.25) | 0.2728 |  | 1.15 (1.00-1.30) | 0.0649 |
| 947 | Urticaria | dermatologic | 336/170,419 | 0.70 (0.41, 1.17) | 0.1728 |  | 0.70 (0.41, 1.18) | 0.1793 |  | 0.71 (0.18-1.23) | 0.2006 |
| 198.2 | Secondary malignancy of respiratory organs | neoplasms | 78/171,033 | 0.37 (0.09, 1.55) | 0.1747 |  | 0.38 (0.09, 1.56) | 0.177 |  | 0.41 (-1.01-1.84) | 0.2225 |
| 427.3 | Other specified cardiac dysrhythmias | circulatory system | 292/168,673 | 0.71 (0.44, 1.16) | 0.1753 |  | 0.70 (0.43, 1.15) | 0.1576 |  | 0.75 (0.26-1.25) | 0.2603 |
| 365.2 | Primary angle-closure glaucoma | sense organs | 109/171,122 | 0.53 (0.21, 1.33) | 0.176 |  | 0.53 (0.21, 1.33) | 0.1787 |  | 0.55 (-0.37-1.47) | 0.2070 |
| 509.2 | Respiratory insufficiency | respiratory | 550/170,426 | 0.80 (0.58, 1.11) | 0.1775 |  | 0.77 (0.56, 1.06) | 0.1076 |  | 0.75 (0.43-1.08) | 0.0884 |
| 568 | Other disorders of peritoneum | digestive | 82/170,043 | 0.49 (0.18, 1.38) | 0.1777 |  | 0.50 (0.18, 1.40) | 0.1855 |  | 0.52 (-0.51-1.55) | 0.2087 |
| 370 | Keratitis | sense organs | 562/169,380 | 0.78 (0.55, 1.12) | 0.1781 |  | 0.78 (0.55, 1.12) | 0.1793 |  | 0.81 (0.45-1.16) | 0.2421 |
| 483 | Acute bronchitis and bronchiolitis | respiratory | 3,036/156,992 | 0.91 (0.79, 1.04) | 0.1785 |  | 0.90 (0.79, 1.04) | 0.1544 |  | 0.92 (0.78-1.06) | 0.2550 |
| 386.21 | Central origin vertigo | sense organs | 108/167,296 | 0.56 (0.24, 1.31) | 0.1817 |  | 0.58 (0.24, 1.36) | 0.206 |  | 0.58 (-0.28-1.44) | 0.2155 |
| 411.8 | Other chronic ischemic heart disease, unspecified | circulatory system | 9,289/149,240 | 0.95 (0.88, 1.02) | 0.1821 |  | 0.98 (0.91, 1.06) | 0.6021 |  | 1.08 (0.99-1.18) | 0.1014 |
| 199 | Neoplasm of uncertain behavior | neoplasms | 576/171,033 | 0.79 (0.55, 1.12) | 0.1833 |  | 0.78 (0.54, 1.11) | 0.1625 |  | 0.79 (0.44-1.15) | 0.2078 |
| 349 | Other and unspecified disorders of the nervous system | neurological | 51/170,510 | 1.77 (0.76, 4.12) | 0.1883 |  | 1.75 (0.75, 4.09) | 0.1959 |  | 1.65 (0.78-2.52) | 0.2577 |
| 509.1 | Respiratory failure | respiratory | 558/170,426 | 0.81 (0.59, 1.11) | 0.1937 |  | 0.77 (0.56, 1.06) | 0.1144 |  | 0.76 (0.44-1.08) | 0.0913 |
| 504 | Other alveolar and parietoalveolar pneumonopathy | respiratory | 142/170,426 | 0.62 (0.30, 1.28) | 0.1969 |  | 0.60 (0.29, 1.25) | 0.1751 |  | 0.61 (-0.12-1.34) | 0.1903 |
| 251.1 | Hypoglycemia | endocrine/  metabolic | 159/156,243 | 0.66 (0.35, 1.24) | 0.1979 |  | 0.65 (0.35, 1.22) | 0.1783 |  | 0.57 (-0.21-1.36) | 0.1631 |
| 586.2 | Cyst of kidney, acquired | genitourinary | 411/166,533 | 0.76 (0.50, 1.16) | 0.2002 |  | 0.77 (0.50, 1.18) | 0.2263 |  | 0.77 (0.34-1.19) | 0.2238 |
| 260 | Protein-calorie malnutrition | endocrine/  metabolic | 516/171,680 | 0.80 (0.57, 1.12) | 0.2029 |  | 0.80 (0.57, 1.11) | 0.1825 |  | 0.83 (0.49-1.17) | 0.2875 |
| 420.2 | Pericarditis | circulatory system | 81/171,261 | 1.54 (0.79, 2.99) | 0.2044 |  | 1.50 (0.77, 2.91) | 0.2358 |  | 1.57 (0.90-2.24) | 0.1842 |
| 805 | Fracture of vertebral column without mention of spinal cord injury | injuries & poisonings | 511/168,201 | 0.80 (0.56, 1.13) | 0.2046 |  | 0.79 (0.56, 1.13) | 0.2044 |  | 0.82 (0.46-1.18) | 0.2732 |
| 571.5 | Other chronic nonalcoholic liver disease | digestive | 1,009/170,077 | 1.16 (0.92, 1.46) | 0.2052 |  | 1.20 (0.95, 1.52) | 0.119 |  | 1.24 (1.00-1.47) | 0.0747 |
| 250.4 | Abnormal glucose | endocrine/  metabolic | 355/156,334 | 0.73 (0.44, 1.20) | 0.212 |  | 0.75 (0.45, 1.24) | 0.2652 |  | 0.76 (0.13-1.39) | 0.3904 |
| 477 | Epistaxis or throat hemorrhage | respiratory | 340/168,697 | 0.76 (0.49, 1.17) | 0.2123 |  | 0.78 (0.50, 1.21) | 0.2626 |  | 0.77 (0.33-1.21) | 0.2457 |
| 568.1 | Peritoneal adhesions (postoperative) (postinfection) | digestive | 78/170,043 | 0.52 (0.19, 1.45) | 0.2123 |  | 0.52 (0.19, 1.47) | 0.2182 |  | 0.54 (-0.49-1.58) | 0.2489 |
| 740.12 | Osteoarthrosis, localized, secondary | musculoskeletal | 45/166,512 | 1.78 (0.71, 4.46) | 0.2151 |  | 1.74 (0.69, 4.35) | 0.2386 |  | 1.74 (0.82-2.67) | 0.2386 |
| 430.1 | Subarachnoid hemorrhage | circulatory system | 336/135,883 | 0.76 (0.50, 1.17) | 0.2196 |  | 0.78 (0.51, 1.19) | 0.2505 |  | 0.74 (0.31-1.17) | 0.1720 |
| 191.1 | Cancer of brain and nervous system | neoplasms | 262/171,621 | 0.72 (0.43, 1.22) | 0.2208 |  | 0.73 (0.44, 1.23) | 0.2392 |  | 0.74 (0.22-1.26) | 0.2581 |
| 285.2 | Anemia of chronic disease | hematopoietic | 30/171,112 | 1.98 (0.66, 5.99) | 0.2242 |  | 2.17 (0.72, 6.58) | 0.1712 |  | 2.04 (0.93-3.16) | 0.2092 |
| 362 | Other retinal disorders | sense organs | 500/171,122 | 0.78 (0.53, 1.16) | 0.2245 |  | 0.80 (0.54, 1.19) | 0.2718 |  | 0.82 (0.43-1.22) | 0.3255 |
| 870 | Open wounds of head; neck; and trunk | injuries & poisonings | 30/172,154 | 0.29 (0.04, 2.19) | 0.2282 |  | 0.27 (0.04, 2.11) | 0.2142 |  | 0.29 (-1.76-2.33) | 0.2305 |
| 870.1 | Open wound or laceration of eye or eyelid | injuries & poisonings | 30/172,154 | 0.29 (0.04, 2.19) | 0.2282 |  | 0.27 (0.04, 2.11) | 0.2142 |  | 0.29 (-1.76-2.33) | 0.2305 |
| 394 | Rheumatic disease of the heart valves | circulatory system | 474/171,662 | 1.21 (0.89, 1.64) | 0.23 |  | 1.20 (0.88, 1.63) | 0.2574 |  | 1.18 (0.87-1.49) | 0.2852 |
| 840 | Sprains and strains | injuries & poisonings | 178/172,023 | 1.44 (0.79, 2.64) | 0.232 |  | 1.49 (0.81, 2.73) | 0.1953 |  | 1.53 (0.92-2.14) | 0.1722 |
| 332 | Parkinson's disease | neurological | 395/170,510 | 0.79 (0.54, 1.16) | 0.2358 |  | 0.81 (0.55, 1.19) | 0.2831 |  | 0.83 (0.44-1.21) | 0.3291 |
| 560.1 | Paralytic ileus | digestive | 36/170,358 | 1.68 (0.71, 3.98) | 0.2385 |  | 1.68 (0.71, 3.99) | 0.2416 |  | 1.79 (0.92-2.66) | 0.1900 |
| 280.2 | Iron deficiency anemia secondary to blood loss (chronic) | hematopoietic | 65/171,112 | 1.52 (0.76, 3.05) | 0.2386 |  | 1.51 (0.75, 3.04) | 0.245 |  | 1.53 (0.83-2.24) | 0.2327 |
| 198.4 | Secondary malignant neoplasm of liver | neoplasms | 71/171,033 | 0.43 (0.10, 1.77) | 0.2402 |  | 0.41 (0.10, 1.68) | 0.2148 |  | 0.43 (-0.99-1.85) | 0.2431 |
| 800.2 | Fracture of unspecified part of femur | injuries & poisonings | 383/168,201 | 1.22 (0.87, 1.70) | 0.2433 |  | 1.20 (0.86, 1.67) | 0.2918 |  | 1.18 (0.84-1.51) | 0.3416 |
| 172.1 | Melanomas of skin, dx or hx | neoplasms | 52/172,069 | 1.70 (0.70, 4.13) | 0.2446 |  | 1.76 (0.72, 4.30) | 0.2137 |  | 1.74 (0.85-2.64) | 0.2243 |
| 172.11 | Melanomas of skin | neoplasms | 52/172,069 | 1.70 (0.70, 4.13) | 0.2446 |  | 1.76 (0.72, 4.30) | 0.2137 |  | 1.74 (0.85-2.64) | 0.2243 |
| 200 | Myeloproliferative disease | neoplasms | 71/171,241 | 0.50 (0.15, 1.63) | 0.2497 |  | 0.51 (0.16, 1.65) | 0.26 |  | 0.49 (-0.69-1.67) | 0.2406 |
| 729 | Other disorders of soft tissues | musculoskeletal | 480/171,316 | 0.81 (0.56, 1.17) | 0.2531 |  | 0.81 (0.56, 1.17) | 0.2524 |  | 0.79 (0.42-1.16) | 0.2083 |
| 729.1 | Rheumatism, unspecified and fibrositis | musculoskeletal | 480/171,316 | 0.81 (0.56, 1.17) | 0.2531 |  | 0.81 (0.56, 1.17) | 0.2524 |  | 0.79 (0.42-1.16) | 0.2083 |
| 433.2 | Occlusion of cerebral arteries | circulatory system | 22,883/135,883 | 0.97 (0.92, 1.02) | 0.2541 |  | 0.99 (0.94, 1.05) | 0.82 |  | 0.99 (0.93-1.05) | 0.7246 |
| 580.13 | Acute glomerulonephritis, NOS | genitourinary | 53/166,533 | 1.63 (0.70, 3.76) | 0.2542 |  | 1.84 (0.79, 4.25) | 0.1554 |  | 2.10 (1.26-2.94) | 0.0835 |
| 535.1 | Acute gastritis | digestive | 57/159,961 | 0.50 (0.15, 1.65) | 0.2555 |  | 0.51 (0.16, 1.68) | 0.2685 |  | 0.51 (-0.68-1.70) | 0.2664 |
| 41 | Bacterial infection NOS | infectious diseases | 46/167,304 | 0.50 (0.15, 1.66) | 0.2586 |  | 0.50 (0.15, 1.65) | 0.2555 |  | 0.47 (-0.73-1.68) | 0.2206 |
| 274 | Gout and other crystal arthropathies | endocrine/  metabolic | 1,218/170,985 | 0.88 (0.71, 1.10) | 0.2609 |  | 0.93 (0.75, 1.16) | 0.5282 |  | 0.93 (0.71-1.15) | 0.5163 |
| 274.1 | Gout | endocrine/  metabolic | 1,218/170,985 | 0.88 (0.71, 1.10) | 0.2609 |  | 0.93 (0.75, 1.16) | 0.5282 |  | 0.93 (0.71-1.15) | 0.5163 |
| 586 | Other disorders of the kidney and ureters | genitourinary | 574/166,533 | 0.82 (0.58, 1.16) | 0.2621 |  | 0.83 (0.58, 1.18) | 0.2881 |  | 0.83 (0.47-1.18) | 0.2872 |
| 374 | Other disorders of eyelids | sense organs | 239/169,380 | 1.28 (0.83, 1.95) | 0.2626 |  | 1.29 (0.84, 1.98) | 0.239 |  | 1.32 (0.90-1.75) | 0.2000 |
| 574.12 | Cholelithiasis with other cholecystitis | digestive | 1,367/158,889 | 0.88 (0.70, 1.10) | 0.2678 |  | 0.94 (0.75, 1.17) | 0.5654 |  | 0.96 (0.73-1.18) | 0.6929 |
| 275 | Disorders of mineral metabolism | endocrine/  metabolic | 148/172,055 | 0.66 (0.32, 1.38) | 0.268 |  | 0.65 (0.31, 1.35) | 0.2446 |  | 0.66 (-0.07-1.40) | 0.2771 |
| 411.1 | Unstable angina (intermediate coronary syndrome) | circulatory system | 358/149,240 | 0.78 (0.51, 1.21) | 0.2688 |  | 0.84 (0.54, 1.29) | 0.422 |  | 0.87 (0.43-1.31) | 0.5300 |
| 433.21 | Cerebral artery occlusion, with cerebral infarction | circulatory system | 20,722/135,883 | 0.97 (0.92, 1.03) | 0.271 |  | 0.99 (0.94, 1.05) | 0.8601 |  | 0.99 (0.93-1.05) | 0.7292 |
| 801.1 | Fracture of foot | injuries & poisonings | 110/168,201 | 0.56 (0.20, 1.58) | 0.2727 |  | 0.57 (0.20, 1.60) | 0.2821 |  | 0.55 (-0.49-1.59) | 0.2611 |
| 275.5 | Disorders of calcium/phosphorus metabolism | endocrine/  metabolic | 145/172,055 | 0.67 (0.32, 1.39) | 0.2761 |  | 0.65 (0.31, 1.35) | 0.25 |  | 0.67 (-0.07-1.41) | 0.2909 |
| 151 | Cancer of stomach | neoplasms | 2,650/162,514 | 1.08 (0.94, 1.23) | 0.2823 |  | 1.06 (0.93, 1.21) | 0.4006 |  | 1.10 (0.97-1.24) | 0.1574 |
| 38 | Septicemia | infectious diseases | 110/167,304 | 0.65 (0.30, 1.43) | 0.283 |  | 0.68 (0.31, 1.49) | 0.3329 |  | 0.71 (-0.07-1.50) | 0.3956 |
| 574.2 | Calculus of bile duct | digestive | 109/158,889 | 0.65 (0.30, 1.43) | 0.2852 |  | 0.67 (0.30, 1.46) | 0.3109 |  | 0.68 (-0.11-1.47) | 0.3398 |
| 994 | Sepsis and SIRS | injuries & poisonings | 109/172,094 | 0.65 (0.30, 1.43) | 0.2896 |  | 0.68 (0.31, 1.50) | 0.3416 |  | 0.72 (-0.07-1.51) | 0.4107 |
| 994.2 | Sepsis | injuries & poisonings | 109/172,094 | 0.65 (0.30, 1.43) | 0.2896 |  | 0.68 (0.31, 1.50) | 0.3416 |  | 0.72 (-0.07-1.51) | 0.4107 |
| 191.11 | Cancer of brain | neoplasms | 252/171,621 | 0.76 (0.45, 1.27) | 0.2919 |  | 0.76 (0.45, 1.29) | 0.3111 |  | 0.78 (0.26-1.30) | 0.3445 |
| 153.2 | Colon cancer | neoplasms | 1,171/162,522 | 0.89 (0.71, 1.11) | 0.2937 |  | 0.90 (0.72, 1.13) | 0.3514 |  | 0.90 (0.67-1.14) | 0.3987 |
| 530.11 | GERD | digestive | 31/171,639 | 0.34 (0.04, 2.58) | 0.2942 |  | 0.34 (0.04, 2.63) | 0.3025 |  | 0.36 (-1.68-2.40) | 0.3247 |
| 470 | Septal Deviations/Turbinate Hypertrophy | respiratory | 250/168,697 | 0.72 (0.39, 1.33) | 0.297 |  | 0.72 (0.39, 1.33) | 0.2956 |  | 0.70 (0.07-1.32) | 0.2563 |
| 70.9 | Hepatitis NOS | infectious diseases | 507/169,432 | 0.81 (0.54, 1.21) | 0.2971 |  | 0.79 (0.53, 1.18) | 0.2558 |  | 0.81 (0.40-1.21) | 0.2944 |
| 159.3 | Malignant neoplasm of gallbladder and extrahepatic bile ducts | neoplasms | 160/166,533 | 0.69 (0.35, 1.38) | 0.2989 |  | 0.73 (0.36, 1.46) | 0.2701 |  | 0.71 (0.01-1.41) | 0.3377 |
| 585.32 | End stage renal disease | genitourinary | 325/162,514 | 1.21 (0.84, 1.73) | 0.2989 |  | 1.22 (0.85, 1.75) | 0.3723 |  | 1.24 (0.88-1.60) | 0.2466 |
| 592.2 | Urethritis and urethral syndrome | genitourinary | 1,312/166,019 | 1.12 (0.90, 1.40) | 0.3031 |  | 1.12 (0.90, 1.40) | 0.3186 |  | 1.15 (0.93-1.38) | 0.2070 |
| 153 | Colorectal cancer | neoplasms | 1,909/162,522 | 0.91 (0.77, 1.09) | 0.3047 |  | 0.92 (0.77, 1.10) | 0.3566 |  | 0.93 (0.75-1.11) | 0.4205 |
| 801 | Fracture of ankle and foot | injuries & poisonings | 120/168,201 | 0.61 (0.24, 1.56) | 0.3055 |  | 0.62 (0.24, 1.58) | 0.3173 |  | 0.61 (-0.33-1.55) | 0.3049 |
| 502 | Postinflammatory pulmonary fibrosis | respiratory | 74/170,426 | 0.59 (0.21, 1.64) | 0.3094 |  | 0.60 (0.21, 1.67) | 0.3262 |  | 0.62 (-0.41-1.65) | 0.3575 |
| 480.1 | Bacterial pneumonia | respiratory | 420/156,992 | 0.81 (0.54, 1.22) | 0.3134 |  | 0.80 (0.54, 1.21) | 0.2907 |  | 0.81 (0.40-1.22) | 0.3061 |
| 136 | Other infectious and parasitic diseases | infectious diseases | 30/172,152 | 0.47 (0.11, 2.04) | 0.3154 |  | 0.44 (0.10, 1.90) | 0.2721 |  | 0.43 (-1.06-1.91) | 0.2611 |
| 427.8 | Sinoatrial node dysfunction (Bradycardia) | circulatory system | 78/168,673 | 0.62 (0.25, 1.57) | 0.3178 |  | 0.64 (0.25, 1.61) | 0.3379 |  | 0.67 (-0.26-1.60) | 0.3958 |
| 696 | Psoriasis and related disorders | dermatologic | 61/171,374 | 0.55 (0.17, 1.80) | 0.3212 |  | 0.59 (0.18, 1.94) | 0.3864 |  | 0.63 (-0.56-1.83) | 0.4517 |
| 696.4 | Psoriasis | dermatologic | 61/171,374 | 0.55 (0.17, 1.80) | 0.3212 |  | 0.59 (0.18, 1.94) | 0.3864 |  | 0.63 (-0.56-1.83) | 0.4517 |
| 696.41 | Psoriasis vulgaris | dermatologic | 61/171,374 | 0.55 (0.17, 1.80) | 0.3212 |  | 0.59 (0.18, 1.94) | 0.3864 |  | 0.63 (-0.56-1.83) | 0.4517 |
| 287 | Purpura and other hemorrhagic conditions | hematopoietic | 237/171,965 | 0.77 (0.46, 1.29) | 0.3213 |  | 0.75 (0.45, 1.26) | 0.2796 |  | 0.79 (0.27-1.31) | 0.3707 |
| 317 | Alcohol-related disorders | mental disorders | 236/171,966 | 1.26 (0.80, 1.99) | 0.3249 |  | 1.21 (0.77, 1.92) | 0.4098 |  | 1.23 (0.76-1.69) | 0.3843 |
| 803.3 | Fracture of clavicle or scapula | injuries & poisonings | 430/168,201 | 1.21 (0.83, 1.77) | 0.3257 |  | 1.17 (0.80, 1.72) | 0.4113 |  | 1.17 (0.78-1.55) | 0.4321 |
| 427.6 | Premature beats | circulatory system | 106/168,673 | 1.38 (0.72, 2.64) | 0.3259 |  | 1.36 (0.71, 2.61) | 0.3462 |  | 1.43 (0.78-2.08) | 0.2773 |
| 446 | Polyarteritis nodosa and allied conditions | circulatory system | 59/171,602 | 1.51 (0.66, 3.45) | 0.3272 |  | 1.57 (0.69, 3.60) | 0.2832 |  | 1.59 (0.76-2.41) | 0.2758 |
| 804 | Fracture of hand or wrist | injuries & poisonings | 120/168,201 | 0.63 (0.25, 1.60) | 0.3294 |  | 0.63 (0.25, 1.60) | 0.3312 |  | 0.68 (-0.25-1.62) | 0.4248 |
| 204 | Leukemia | neoplasms | 465/171,241 | 0.83 (0.57, 1.20) | 0.33 |  | 0.83 (0.58, 1.21) | 0.3362 |  | 0.85 (0.48-1.22) | 0.3938 |
| 454.11 | Varicose veins of lower extremity, symptomtic | circulatory system | 110/167,579 | 0.60 (0.22, 1.68) | 0.3306 |  | 0.61 (0.22, 1.71) | 0.3501 |  | 0.61 (-0.42-1.64) | 0.3439 |
| 433.5 | Cerebral aneurysm | circulatory system | 56/135,883 | 0.49 (0.12, 2.08) | 0.3325 |  | 0.48 (0.11, 2.06) | 0.3258 |  | 0.48 (-0.97-1.93) | 0.3219 |
| 158 | Neoplasm of unspecified nature of digestive system | neoplasms | 40/162,514 | 0.37 (0.05, 2.78) | 0.3332 |  | 0.36 (0.05, 2.75) | 0.3282 |  | 0.36 (-1.66-2.39) | 0.3299 |
| 287.3 | Thrombocytopenia | hematopoietic | 236/171,965 | 0.77 (0.46, 1.30) | 0.3335 |  | 0.76 (0.45, 1.27) | 0.2914 |  | 0.79 (0.27-1.31) | 0.3826 |
| 578.9 | Hemorrhage of gastrointestinal tract | digestive | 2249/169,670 | 1.08 (0.93, 1.25) | 0.3351 |  | 1.07 (0.92, 1.25) | 0.3537 |  | 1.07 (0.92-1.22) | 0.3794 |
| 509 | Respiratory failure, insufficiency, arrest | respiratory | 578/170,426 | 0.86 (0.64, 1.17) | 0.3411 |  | 0.82 (0.61, 1.12) | 0.2163 |  | 0.81 (0.51-1.12) | 0.1864 |
| 362.3 | Other nondiabetic retinopathy | sense organs | 252/171,122 | 0.76 (0.42, 1.35) | 0.3434 |  | 0.75 (0.42, 1.35) | 0.3424 |  | 0.76 (0.18-1.34) | 0.3592 |
| 362.31 | Separation of retinal layers | sense organs | 252/171,122 | 0.76 (0.42, 1.35) | 0.3434 |  | 0.75 (0.42, 1.35) | 0.3424 |  | 0.76 (0.18-1.34) | 0.3592 |
| 362.8 | Retinal hemorrhage/ischemia | sense organs | 59/171,122 | 0.56 (0.17, 1.85) | 0.3449 |  | 0.59 (0.18, 1.94) | 0.3848 |  | 0.59 (-0.60-1.79) | 0.3913 |
| 580 | Nephritis; nephrosis; renal sclerosis | genitourinary | 927/166,533 | 0.89 (0.69, 1.14) | 0.3479 |  | 0.88 (0.69, 1.14) | 0.3435 |  | 0.86 (0.61-1.12) | 0.2608 |
| 416 | Cardiomegaly | circulatory system | 66/170,711 | 1.38 (0.70, 2.69) | 0.3519 |  | 1.43 (0.73, 2.80) | 0.2991 |  | 1.43 (0.75-2.11) | 0.3023 |
| 727 | Other disorders of synovium, tendon, and bursa | musculoskeletal | 139/171,316 | 0.67 (0.29, 1.57) | 0.3573 |  | 0.64 (0.27, 1.50) | 0.3063 |  | 0.68 (-0.17-1.54) | 0.3820 |
| 727.4 | Ganglion and cyst of synovium, tendon, and bursa | musculoskeletal | 139/171,316 | 0.67 (0.29, 1.57) | 0.3573 |  | 0.64 (0.27, 1.50) | 0.3063 |  | 0.68 (-0.17-1.54) | 0.3820 |
| 726 | Peripheral enthesopathies and allied syndromes | musculoskeletal | 181/171,316 | 1.27 (0.77, 2.09) | 0.3575 |  | 1.25 (0.76, 2.08) | 0.3788 |  | 1.34 (0.83-1.84) | 0.2578 |
| 726.1 | Enthesopathy | musculoskeletal | 181/171,316 | 1.27 (0.77, 2.09) | 0.3575 |  | 1.25 (0.76, 2.08) | 0.3788 |  | 1.34 (0.83-1.84) | 0.2578 |
| 580.12 | Non-proliferative glomerulonephritis | genitourinary | 37/166,533 | 0.39 (0.05, 2.95) | 0.3627 |  | 0.38 (0.05, 2.85) | 0.3444 |  | 0.35 (-1.68-2.38) | 0.3107 |
| 594.1 | Calculus of kidney | genitourinary | 2,545/165,873 | 0.93 (0.79, 1.09) | 0.3629 |  | 0.95 (0.80, 1.11) | 0.5035 |  | 0.98 (0.81-1.14) | 0.7894 |
| 172 | Skin cancer | neoplasms | 134/172,069 | 1.31 (0.73, 2.32) | 0.364 |  | 1.29 (0.73, 2.30) | 0.3811 |  | 1.32 (0.74-1.89) | 0.3527 |
| 211 | Benign neoplasm of other parts of digestive system | neoplasms | 371/163,114 | 0.81 (0.51, 1.28) | 0.3648 |  | 0.81 (0.51, 1.29) | 0.3704 |  | 0.84 (0.37-1.30) | 0.4491 |
| 525 | Other diseases of the teeth and supporting structures | digestive | 32/168,875 | 0.39 (0.05, 2.98) | 0.3648 |  | 0.37 (0.05, 2.82) | 0.3362 |  | 0.46 (-1.58-2.49) | 0.4501 |
| 530 | Diseases of esophagus | digestive | 564/171,639 | 0.86 (0.61, 1.20) | 0.3671 |  | 0.86 (0.61, 1.20) | 0.3736 |  | 0.90 (0.56-1.23) | 0.5258 |
| 578 | Gastrointestinal hemorrhage | digestive | 2,372/169,670 | 1.07 (0.92, 1.24) | 0.3729 |  | 1.07 (0.92, 1.23) | 0.3939 |  | 1.06 (0.91-1.21) | 0.4234 |
| 427.9 | Palpitations | circulatory system | 567/168,673 | 0.87 (0.63, 1.19) | 0.3759 |  | 0.86 (0.63, 1.19) | 0.3722 |  | 0.87 (0.55-1.20) | 0.4081 |
| 290.12 | Dementia with cerebral degenerations | mental disorders | 71/171,928 | 1.31 (0.72, 2.40) | 0.378 |  | 1.29 (0.71, 2.36) | 0.4059 |  | 1.25 (0.64-1.86) | 0.4696 |
| 377.1 | Optic atrophy | sense organs | 45/171,572 | 1.50 (0.61, 3.69) | 0.3788 |  | 1.44 (0.58, 3.55) | 0.4283 |  | 1.42 (0.51-2.33) | 0.4493 |
| 401.22 | Hypertensive chronic kidney disease | circulatory system | 145/87,647 | 0.73 (0.37, 1.46) | 0.379 |  | 0.80 (0.40, 1.59) | 0.518 |  | 0.95 (-0.56-2.47) | 0.9506 |
| 728.7 | Fasciitis | musculoskeletal | 90/171,316 | 1.44 (0.64, 3.25) | 0.3796 |  | 1.42 (0.63, 3.21) | 0.3973 |  | 1.62 (0.81-2.44) | 0.2447 |
| 574.11 | Cholelithiasis with acute cholecystitis | digestive | 355/158,889 | 0.83 (0.54, 1.26) | 0.3809 |  | 0.87 (0.57, 1.33) | 0.5189 |  | 0.83 (0.41-1.25) | 0.3844 |
| 10 | Tuberculosis | infectious diseases | 4,754/167,304 | 1.05 (0.94, 1.16) | 0.3836 |  | 1.00 (0.90, 1.11) | 0.9711 |  | 0.91 (0.80-1.03) | 0.1108 |
| 427 | Cardiac dysrhythmias | circulatory system | 3,439/168,673 | 0.95 (0.83, 1.07) | 0.384 |  | 0.94 (0.83, 1.07) | 0.3709 |  | 0.97 (0.84-1.09) | 0.5883 |
| 189.21 | Malignant neoplasm of bladder | neoplasms | 426/171,450 | 0.85 (0.58, 1.23) | 0.3842 |  | 0.84 (0.58, 1.23) | 0.3757 |  | 0.84 (0.46-1.22) | 0.3685 |
| 798 | Malaise and fatigue | symptoms | 3,889/168,314 | 1.06 (0.93, 1.22) | 0.39 |  | 1.06 (0.92, 1.21) | 0.4439 |  | 1.06 (0.92-1.20) | 0.3868 |
| 577.2 | Chronic pancreatitis | digestive | 71/171,480 | 0.60 (0.18, 1.96) | 0.3945 |  | 0.57 (0.17, 1.86) | 0.3488 |  | 0.56 (-0.63-1.75) | 0.3411 |
| 535.2 | Atrophic gastritis | digestive | 150/159,961 | 1.27 (0.73, 2.23) | 0.3946 |  | 1.24 (0.71, 2.17) | 0.457 |  | 1.34 (0.78-1.91) | 0.3053 |
| 290.1 | Dementias | mental disorders | 239/171,928 | 1.17 (0.82, 1.66) | 0.3977 |  | 1.17 (0.82, 1.67) | 0.3866 |  | 1.18 (0.83-1.54) | 0.3525 |
| 224 | Benign neoplasm of eye | neoplasms | 176/171,621 | 0.74 (0.37, 1.48) | 0.3979 |  | 0.74 (0.37, 1.48) | 0.3996 |  | 0.74 (0.04-1.43) | 0.3947 |
| 348.8 | Encephalopathy, not elsewhere classified | neurological | 217/170,510 | 0.79 (0.45, 1.38) | 0.4088 |  | 0.77 (0.44, 1.36) | 0.3684 |  | 0.79 (0.22-1.36) | 0.4128 |
| 580.14 | Chronic glomerulonephritis, NOS | genitourinary | 423/166,533 | 0.86 (0.60, 1.24) | 0.4164 |  | 0.85 (0.59, 1.23) | 0.3967 |  | 0.82 (0.45-1.18) | 0.2845 |
| 149.4 | Cancer of larynx | neoplasms | 88/172,112 | 1.37 (0.64, 2.94) | 0.4165 |  | 1.28 (0.60, 2.76) | 0.3883 |  | 1.27 (0.50-2.04) | 0.5395 |
| 747.1 | Cardiac congenital anomalies | congenital anomalies | 190/171,523 | 0.79 (0.45, 1.39) | 0.4165 |  | 0.78 (0.45, 1.37) | 0.5206 |  | 0.80 (0.24-1.36) | 0.4458 |
| 535.8 | Other specified gastritis | digestive | 4,815/159,961 | 1.05 (0.94, 1.17) | 0.4175 |  | 1.02 (0.92, 1.14) | 0.709 |  | 1.04 (0.93-1.15) | 0.5074 |
| 516.1 | Hemoptysis | respiratory | 239/171,963 | 1.20 (0.77, 1.88) | 0.4181 |  | 1.15 (0.73, 1.80) | 0.5411 |  | 1.15 (0.70-1.60) | 0.5432 |
| 522.1 | Pulpitis and necrosis of tooth pulp | digestive | 336/168,875 | 0.82 (0.50, 1.34) | 0.4187 |  | 0.81 (0.49, 1.33) | 0.4054 |  | 0.84 (0.34-1.33) | 0.4780 |
| 740.1 | Osteoarthritis; localized | musculoskeletal | 432/166,512 | 0.86 (0.60, 1.24) | 0.4192 |  | 0.92 (0.64, 1.32) | 0.6485 |  | 0.93 (0.57-1.30) | 0.7115 |
| 331.9 | Cerebral degeneration, unspecified | neurological | 359/170,510 | 1.14 (0.83, 1.58) | 0.4205 |  | 1.12 (0.81, 1.55) | 0.4777 |  | 1.13 (0.80-1.45) | 0.4744 |
| 700 | Corns and callosities | dermatologic | 31/172,172 | 0.43 (0.06, 3.31) | 0.4207 |  | 0.43 (0.06, 3.28) | 0.4156 |  | 0.41 (-1.63-2.45) | 0.3888 |
| 747 | Cardiac and circulatory congenital anomalies | congenital anomalies | 91/172,112 | 1.37 (0.64, 2.92) | 0.4222 |  | 1.28 (0.60, 2.75) | 0.5195 |  | 1.27 (0.50-2.04) | 0.5367 |
| 285 | Other anemias | hematopoietic | 915/166,533 | 0.90 (0.70, 1.16) | 0.4228 |  | 0.93 (0.73, 1.19) | 0.5084 |  | 0.90 (0.65-1.15) | 0.4004 |
| 585.3 | Chronic renal failure [CKD] | genitourinary | 859/171,112 | 1.00 (0.87, 1.38) | 0.4228 |  | 1.08 (0.86, 1.36) | 0.5672 |  | 1.08 (0.85-1.31) | 0.5297 |
| 290.13 | Senile dementia | mental disorders | 133/171,928 | 1.22 (0.75, 1.98) | 0.4236 |  | 1.23 (0.76, 1.99) | 0.4092 |  | 1.24 (0.76-1.73) | 0.3801 |
| 594.8 | Renal colic | genitourinary | 877/165,873 | 0.90 (0.69, 1.17) | 0.4273 |  | 0.92 (0.71, 1.20) | 0.5388 |  | 0.96 (0.70-1.23) | 0.7791 |
| 371.1 | Uveitis, noninfectious or NOS | sense organs | 112/169,380 | 1.32 (0.67, 2.59) | 0.4284 |  | 1.31 (0.66, 2.58) | 0.4424 |  | 1.29 (0.61-1.98) | 0.4601 |
| 327.3 | Sleep apnea | neurological | 54/171,515 | 1.55 (0.52, 4.65) | 0.4313 |  | 1.75 (0.58, 5.27) | 0.3192 |  | 1.88 (0.78-2.97) | 0.2618 |
| 241.1 | Nontoxic uninodular goiter | endocrine/  metabolic | 212/168,673 | 1.23 (0.73, 2.06) | 0.4325 |  | 1.26 (0.75, 2.11) | 0.4132 |  | 1.32 (0.80-1.84) | 0.2968 |
| 427.1 | Paroxysmal tachycardia, unspecified | circulatory system | 48/171,651 | 0.44 (0.06, 3.36) | 0.4325 |  | 0.43 (0.06, 3.25) | 0.3828 |  | 0.43 (-1.59-2.46) | 0.4209 |
| 516 | Abnormal sputum | respiratory | 240/171,963 | 1.20 (0.76, 1.87) | 0.434 |  | 1.14 (0.73, 1.79) | 0.5592 |  | 1.14 (0.69-1.59) | 0.5647 |
| 367 | Disorders of refraction and accommodation; blindness and low vision | sense organs | 518/166,533 | 0.88 (0.63, 1.22) | 0.4399 |  | 0.88 (0.64, 1.22) | 0.4771 |  | 0.86 (0.53-1.19) | 0.3701 |
| 580.1 | Glomerulonephritis | genitourinary | 71/172,132 | 0.62 (0.19, 2.07) | 0.4399 |  | 0.65 (0.19, 2.15) | 0.4521 |  | 0.72 (-0.48-1.92) | 0.5931 |
| 193 | Thyroid cancer | neoplasms | 99/172,025 | 0.67 (0.24, 1.87) | 0.4421 |  | 0.69 (0.25, 1.93) | 0.4793 |  | 0.70 (-0.34-1.73) | 0.4901 |
| 155.1 | Malignant neoplasm of liver, primary | neoplasms | 281/162,514 | 1.19 (0.77, 1.83) | 0.4422 |  | 1.18 (0.76, 1.82) | 0.4667 |  | 1.19 (0.75-1.62) | 0.4456 |
| 800 | Fracture of lower limb | injuries & poisonings | 1,452/168,201 | 1.08 (0.89, 1.30) | 0.4437 |  | 1.06 (0.88, 1.28) | 0.5263 |  | 1.06 (0.87-1.25) | 0.5316 |
| 580.2 | Nephrotic syndrome without mention of glomerulonephritis | genitourinary | 243/166,533 | 0.82 (0.48, 1.38) | 0.4466 |  | 0.83 (0.49, 1.40) | 0.4852 |  | 0.82 (0.30-1.35) | 0.4666 |
| 198.1 | Secondary malignancy of lymph nodes | neoplasms | 116/171,033 | 1.28 (0.67, 2.44) | 0.4481 |  | 1.25 (0.66, 2.38) | 0.4962 |  | 1.35 (0.70-1.99) | 0.3682 |
| 427.42 | Cardiac arrest | circulatory system | 67/168,673 | 1.37 (0.61, 3.09) | 0.4504 |  | 1.44 (0.64, 3.25) | 0.383 |  | 1.45 (0.63-2.28) | 0.3742 |
| 567 | Peritonitis and retroperitoneal infections | digestive | 148/170,043 | 0.78 (0.40, 1.50) | 0.4519 |  | 0.76 (0.39, 1.47) | 0.4101 |  | 0.75 (0.09-1.42) | 0.4052 |
| 149 | Cancer of larynx, pharynx, nasal cavities | neoplasms | 534/171,523 | 0.88 (0.63, 1.23) | 0.4534 |  | 0.86 (0.62, 1.20) | 0.3726 |  | 0.88 (0.54-1.21) | 0.4383 |
| 189.4 | Malignant neoplasm of other urinary organs | neoplasms | 55/171,450 | 1.40 (0.58, 3.39) | 0.4543 |  | 1.42 (0.59, 3.45) | 0.4346 |  | 1.45 (0.56-2.34) | 0.4119 |
| 331 | Other cerebral degenerations | neurological | 429/170,510 | 1.12 (0.83, 1.52) | 0.4593 |  | 1.00 (0.82, 1.50) | 0.5093 |  | 1.11 (0.80-1.42) | 0.5022 |
| 250.42 | Other abnormal glucose | endocrine/  metabolic | 312/156,334 | 0.82 (0.49, 1.39) | 0.4601 |  | 0.84 (0.50, 1.42) | 0.5205 |  | 0.82 (0.15-1.49) | 0.5583 |
| 531.3 | Duodenal ulcer | digestive | 540/158,803 | 1.13 (0.81, 1.58) | 0.4636 |  | 1.00 (0.79, 1.54) | 0.5675 |  | 1.14 (0.81-1.47) | 0.4416 |
| 550 | Abdominal hernia | digestive | 30/172,173 | 0.47 (0.06, 3.56) | 0.4644 |  | 0.48 (0.06, 3.66) | 0.4806 |  | 0.47 (-1.56-2.49) | 0.4605 |
| 426.4 | Anomalous atrioventricular excitation | circulatory system | 31/168,673 | 0.47 (0.06, 3.58) | 0.4648 |  | 0.44 (0.06, 3.41) | 0.4358 |  | 0.55 (-1.49-2.58) | 0.5621 |
| 474.2 | Chronic tonsillitis and adenoiditis | respiratory | 294/168,697 | 0.80 (0.45, 1.44) | 0.4665 |  | 0.84 (0.47, 1.50) | 0.5467 |  | 0.83 (0.24-1.42) | 0.5392 |
| 300.11 | Generalized anxiety disorder | mental disorders | 160/170,474 | 1.22 (0.71, 2.10) | 0.4694 |  | 1.12 (0.65, 1.94) | 0.6724 |  | 1.06 (0.50-1.61) | 0.8463 |
| 523.32 | Chronic periodontitis | digestive | 2,853/168,875 | 1.06 (0.90, 1.26) | 0.4695 |  | 1.06 (0.90, 1.26) | 0.4734 |  | 1.08 (0.91-1.25) | 0.3871 |
| 348 | Other conditions of brain | neurological | 269/170,510 | 0.83 (0.51, 1.37) | 0.471 |  | 0.81 (0.50, 1.33) | 0.415 |  | 0.83 (0.33-1.32) | 0.4494 |
| 334 | Degenerative disease of the spinal cord | neurological | 31/170,510 | 0.47 (0.06, 3.65) | 0.4714 |  | 0.47 (0.06, 3.67) | 0.4746 |  | 0.52 (-1.53-2.57) | 0.5342 |
| 427.2 | Atrial fibrillation and flutter | circulatory system | 74/168,673 | 0.69 (0.25, 1.92) | 0.4739 |  | 0.70 (0.25, 1.96) | 0.5002 |  | 0.74 (-0.29-1.77) | 0.5625 |
| 226 | Benign neoplasm of thyroid glands | neoplasms | 45/172,025 | 0.58 (0.13, 2.54) | 0.474 |  | 0.58 (0.13, 2.54) | 0.4716 |  | 0.60 (-0.87-2.08) | 0.5036 |
| 442 | Other aneurysm | circulatory system | 253/171,602 | 0.83 (0.50, 1.38) | 0.4756 |  | 0.84 (0.51, 1.40) | 0.502 |  | 0.82 (0.31-1.33) | 0.4392 |
| 284 | Aplastic anemia | hematopoietic | 61/171,112 | 0.69 (0.24, 1.94) | 0.479 |  | 0.66 (0.23, 1.88) | 0.4405 |  | 0.71 (-0.34-1.75) | 0.5140 |
| 189.2 | Cancer of bladder | neoplasms | 463/171,450 | 0.88 (0.62, 1.25) | 0.4798 |  | 0.88 (0.61, 1.25) | 0.4682 |  | 0.88 (0.52-1.23) | 0.4696 |
| 523.3 | Periodontitis (acute or chronic) | digestive | 2,956/168,875 | 1.06 (0.90, 1.25) | 0.4834 |  | 1.06 (0.90, 1.25) | 0.489 |  | 1.08 (0.91-1.24) | 0.3740 |
| 204.11 | Lymphoid leukemia, acute | neoplasms | 40/171,241 | 0.59 (0.14, 2.55) | 0.4839 |  | 0.61 (0.14, 2.61) | 0.5036 |  | 0.62 (-0.83-2.08) | 0.5271 |
| 170.2 | Cancer of connective tissue | neoplasms | 78/171,958 | 1.33 (0.59, 2.98) | 0.4859 |  | 1.29 (0.58, 2.90) | 0.5318 |  | 1.25 (0.44-2.07) | 0.5843 |
| 290.11 | Alzheimer's disease | mental disorders | 37/171,928 | 0.69 (0.24, 1.98) | 0.4866 |  | 0.71 (0.24, 2.04) | 0.5199 |  | 0.77 (-0.30-1.83) | 0.6229 |
| 803 | Fracture of upper limb | injuries & poisonings | 1,102/168,201 | 0.91 (0.71, 1.18) | 0.4883 |  | 0.91 (0.70, 1.17) | 0.4589 |  | 0.92 (0.67-1.18) | 0.5486 |
| 721.2 | Spondylosis with myelopathy | musculoskeletal | 74/171,526 | 0.69 (0.24, 1.97) | 0.4893 |  | 0.66 (0.23, 1.89) | 0.4415 |  | 0.65 (-0.40-1.71) | 0.4304 |
| 575.7 | Other disorders of gallbladder | digestive | 242/158,889 | 0.81 (0.44, 1.48) | 0.4939 |  | 0.81 (0.44, 1.48) | 0.4873 |  | 0.83 (0.22-1.43) | 0.5356 |
| 721 | Spondylosis and allied disorders | musculoskeletal | 75/171,526 | 0.70 (0.25, 1.98) | 0.4964 |  | 0.67 (0.24, 1.91) | 0.4549 |  | 0.66 (-0.39-1.72) | 0.4468 |
| 427.4 | Cardiac arrest and ventricular fibrillation | circulatory system | 68/168,673 | 1.33 (0.59, 2.99) | 0.4967 |  | 1.38 (0.61, 3.12) | 0.4337 |  | 1.40 (0.57-2.22) | 0.4274 |
| 754 | Congenital musculoskeletal deformities of spine | congenital anomalies | 75/172,128 | 0.72 (0.28, 1.86) | 0.4972 |  | 0.76 (0.29, 1.95) | 0.562 |  | 0.78 (-0.17-1.73) | 0.6062 |
| 752 | Nervous system congenital anomalies | congenital anomalies | 75/172,121 | 0.72 (0.28, 1.86) | 0.4973 |  | 0.76 (0.29, 1.95) | 0.5619 |  | 0.78 (-0.17-1.73) | 0.6061 |
| 752.1 | Neural tube defects | congenital anomalies | 75/172,121 | 0.72 (0.28, 1.86) | 0.4973 |  | 0.76 (0.29, 1.95) | 0.5619 |  | 0.78 (-0.17-1.73) | 0.6061 |
| 425.12 | Other hypertrophic cardiomyopathy | circulatory system | 31/171,261 | 0.49 (0.06, 3.81) | 0.499 |  | 0.50 (0.07, 3.86) | 0.5076 |  | 0.45 (-1.60-2.51) | 0.4501 |
| 378.5 | Paralytic strabismus | sense organs | 56/171,572 | 1.36 (0.56, 3.33) | 0.5009 |  | 1.38 (0.56, 3.38) | 0.4829 |  | 1.45 (0.55-2.34) | 0.4168 |
| 530.1 | Esophagitis, GERD and related diseases | digestive | 550/171,639 | 0.89 (0.64, 1.25) | 0.5063 |  | 0.89 (0.64, 1.25) | 0.5012 |  | 0.93 (0.59-1.27) | 0.6743 |
| 783 | Fever of unknown origin | symptoms | 579/171,624 | 0.90 (0.65, 1.23) | 0.5068 |  | 0.90 (0.66, 1.24) | 0.5166 |  | 0.93 (0.61-1.24) | 0.6345 |
| 191 | Manlignant and unknown neoplasms of brain and nervous system | neoplasms | 315/171,621 | 0.86 (0.56, 1.34) | 0.5087 |  | 0.88 (0.57, 1.36) | 0.5641 |  | 0.89 (0.45-1.33) | 0.5900 |
| 327 | Sleep disorders | neurological | 638/171,515 | 0.90 (0.66, 1.23) | 0.5102 |  | 0.89 (0.65, 1.21) | 0.4483 |  | 0.89 (0.57-1.20) | 0.4518 |
| 430.3 | Subdural hemorrhage | circulatory system | 249/135,883 | 0.87 (0.56, 1.34) | 0.5134 |  | 0.88 (0.57, 1.36) | 0.5645 |  | 0.87 (0.43-1.31) | 0.5339 |
| 70 | Viral hepatitis | infectious diseases | 937/169,432 | 0.91 (0.69, 1.21) | 0.518 |  | 0.89 (0.67, 1.19) | 0.4429 |  | 0.90 (0.62-1.19) | 0.4792 |
| 300.1 | Anxiety disorder | mental disorders | 183/170,474 | 1.19 (0.70, 2.00) | 0.5187 |  | 1.00 (0.65, 1.86) | 0.7151 |  | 1.05 (0.51-1.58) | 0.8687 |
| 710 | Osteomyelitis, periostitis, and other infections involving bone | musculoskeletal | 68/171,324 | 1.31 (0.58, 2.96) | 0.52 |  | 1.33 (0.59, 3.02) | 0.492 |  | 1.33 (0.51-2.16) | 0.4929 |
| 710.1 | Osteomyelitis | musculoskeletal | 68/171,324 | 1.31 (0.58, 2.96) | 0.52 |  | 1.33 (0.59, 3.02) | 0.492 |  | 1.33 (0.51-2.16) | 0.4929 |
| 710.19 | Unspecified osteomyelitis | musculoskeletal | 68/171,324 | 1.31 (0.58, 2.96) | 0.52 |  | 1.33 (0.59, 3.02) | 0.492 |  | 1.33 (0.51-2.16) | 0.4929 |
| 204.4 | Multiple myeloma | neoplasms | 157/171,241 | 0.81 (0.42, 1.56) | 0.5254 |  | 0.81 (0.42, 1.58) | 0.5423 |  | 0.83 (0.17-1.50) | 0.5918 |
| 830 | Dislocation | injuries & poisonings | 176/172,020 | 0.82 (0.43, 1.54) | 0.531 |  | 0.83 (0.44, 1.57) | 0.5703 |  | 0.86 (0.22-1.49) | 0.6330 |
| 371 | Inflammation of the eye | sense organs | 232/169,380 | 0.84 (0.48, 1.47) | 0.5342 |  | 0.83 (0.47, 1.45) | 0.5121 |  | 0.85 (0.28-1.41) | 0.5636 |
| 555 | Inflammatory bowel disease and other gastroenteritis and colitis | digestive | 1,624/166,019 | 1.06 (0.87, 1.30) | 0.5344 |  | 1.06 (0.87, 1.29) | 0.4388 |  | 1.10 (0.90-1.30) | 0.3452 |
| 555.2 | Ulcerative colitis | digestive | 132/170,358 | 0.79 (0.38, 1.66) | 0.5344 |  | 0.75 (0.35, 1.57) | 0.4388 |  | 0.77 (0.02-1.52) | 0.4930 |
| 592 | Cystitis and urethritis | genitourinary | 132/170,358 | 0.79 (0.38, 1.66) | 0.5344 |  | 0.75 (0.35, 1.57) | 0.5569 |  | 0.77 (0.02-1.52) | 0.4930 |
| 575 | Other biliary tract disease | digestive | 839/158,889 | 0.92 (0.71, 1.20) | 0.5391 |  | 0.93 (0.71, 1.21) | 0.5869 |  | 0.96 (0.69-1.22) | 0.7455 |
| 379 | Other disorders of eye | sense organs | 323/171,572 | 1.14 (0.75, 1.73) | 0.5457 |  | 1.15 (0.76, 1.75) | 0.5109 |  | 1.16 (0.74-1.58) | 0.4836 |
| 411.9 | Other acute and subacute forms of ischemic heart disease | circulatory system | 185/168,673 | 1.18 (0.68, 2.05) | 0.5511 |  | 1.20 (0.69, 2.09) | 0.4749 |  | 1.25 (0.70-1.81) | 0.4273 |
| 427.11 | Paroxysmal supraventricular tachycardia | circulatory system | 657/149,240 | 1.09 (0.82, 1.43) | 0.5511 |  | 1.00 (0.84, 1.46) | 0.5103 |  | 1.11 (0.83-1.39) | 0.4704 |
| 564 | Functional digestive disorders | digestive | 55/170,358 | 0.70 (0.21, 2.30) | 0.5547 |  | 0.69 (0.21, 2.29) | 0.5493 |  | 0.69 (-0.51-1.89) | 0.5410 |
| 740.11 | Osteoarthrosis, localized, primary | musculoskeletal | 313/166,512 | 0.88 (0.58, 1.34) | 0.5567 |  | 0.94 (0.62, 1.43) | 0.7793 |  | 0.95 (0.53-1.37) | 0.8185 |
| 352 | Disorders of other cranial nerves | neurological | 614/171,360 | 0.91 (0.67, 1.24) | 0.558 |  | 0.94 (0.69, 1.28) | 0.7049 |  | 0.94 (0.63-1.24) | 0.6777 |
| 159 | Malignant neoplasm of other and ill-defined sites within the digestive organs and peritoneum | neoplasms | 487/162,514 | 1.00 (0.80, 1.50) | 0.5623 |  | 1.00 (0.81, 1.51) | 0.5362 |  | 1.11 (0.79-1.42) | 0.5218 |
| 202.2 | Non-Hodgkins lymphoma | neoplasms | 467/171,241 | 1.00 (0.79, 1.55) | 0.5628 |  | 1.00 (0.79, 1.56) | 0.5363 |  | 1.17 (0.83-1.51) | 0.3635 |
| 202 | Cancer of other lymphoid, histiocytic tissue | neoplasms | 469/171,241 | 1.00 (0.79, 1.55) | 0.5636 |  | 1.00 (0.79, 1.56) | 0.5386 |  | 1.17 (0.83-1.51) | 0.3641 |
| 501 | Pneumonitis due to inhalation of food or vomitus | respiratory | 71/170,426 | 0.76 (0.30, 1.93) | 0.5638 |  | 0.75 (0.29, 1.91) | 0.5449 |  | 0.73 (-0.20-1.67) | 0.5174 |
| 366.2 | Senile cataract | sense organs | 2,072/166,238 | 0.96 (0.82, 1.11) | 0.5654 |  | 0.96 (0.82, 1.12) | 0.5978 |  | 0.96 (0.81-1.12) | 0.6395 |
| 428.2 | Heart failure NOS | circulatory system | 181/170,345 | 0.86 (0.52, 1.43) | 0.5687 |  | 0.92 (0.56, 1.52) | 0.7364 |  | 0.90 (0.39-1.40) | 0.6774 |
| 386.2 | Peripheral or central vertigo | sense organs | 173/167,296 | 0.83 (0.44, 1.57) | 0.5716 |  | 0.82 (0.44, 1.55) | 0.5476 |  | 0.84 (0.21-1.47) | 0.5894 |
| 530.12 | Ulcer of esophagus | digestive | 53/171,639 | 1.32 (0.50, 3.45) | 0.5725 |  | 1.36 (0.52, 3.56) | 0.5309 |  | 1.47 (0.51-2.44) | 0.4331 |
| 572 | Ascites (non malignant) | digestive | 159/170,077 | 1.16 (0.69, 1.97) | 0.5734 |  | 1.18 (0.70, 2.00) | 0.5387 |  | 1.21 (0.68-1.73) | 0.4885 |
| 593 | Hematuria | genitourinary | 242/166,019 | 0.87 (0.54, 1.41) | 0.5745 |  | 0.89 (0.55, 1.45) | 0.6398 |  | 0.90 (0.41-1.38) | 0.6590 |
| 585.2 | Renal failure NOS | genitourinary | 931/166,533 | 1.07 (0.85, 1.34) | 0.5853 |  | 1.08 (0.86, 1.35) | 0.5252 |  | 1.06 (0.83-1.29) | 0.6412 |
| 377.3 | Optic neuritis/neuropathy | sense organs | 71/171,572 | 0.75 (0.26, 2.13) | 0.5896 |  | 0.76 (0.27, 2.16) | 0.6083 |  | 0.73 (-0.32-1.78) | 0.5502 |
| 523 | Gingival and periodontal diseases | digestive | 3,084/168,875 | 1.05 (0.89, 1.23) | 0.5908 |  | 1.04 (0.89, 1.23) | 0.6075 |  | 1.06 (0.90-1.22) | 0.4828 |
| 500 | Lung disease due to external agents | respiratory | 71/170,426 | 0.80 (0.36, 1.79) | 0.5916 |  | 0.78 (0.35, 1.73) | 0.5369 |  | 0.79 (-0.01-1.59) | 0.5591 |
| 337 | Disorders of the autonomic nervous system | neurological | 48/170,510 | 0.72 (0.22, 2.39) | 0.5929 |  | 0.73 (0.22, 2.42) | 0.604 |  | 0.71 (-0.51-1.92) | 0.5736 |
| 198 | Secondary malignant neoplasm | neoplasms | 398/171,033 | 0.90 (0.60, 1.34) | 0.5937 |  | 0.89 (0.59, 1.32) | 0.5526 |  | 0.94 (0.53-1.34) | 0.7451 |
| 352.2 | Facial nerve disorders [CN7] | neurological | 496/171,360 | 0.91 (0.64, 1.29) | 0.599 |  | 0.95 (0.67, 1.34) | 0.7567 |  | 0.93 (0.58-1.28) | 0.6990 |
| 362.2 | Degeneration of macula and posterior pole of retina | sense organs | 155/171,122 | 0.83 (0.42, 1.66) | 0.6012 |  | 0.86 (0.43, 1.71) | 0.6606 |  | 0.88 (0.19-1.58) | 0.7241 |
| 362.29 | Macular degeneration (senile) of retina NOS | sense organs | 155/171,122 | 0.83 (0.42, 1.66) | 0.6012 |  | 0.86 (0.43, 1.71) | 0.6606 |  | 0.88 (0.19-1.58) | 0.7241 |
| 577 | Diseases of pancreas | digestive | 723/171,480 | 0.92 (0.69, 1.24) | 0.6031 |  | 0.96 (0.71, 1.29) | 0.7717 |  | 0.98 (0.69-1.28) | 0.9095 |
| 172.2 | Other non-epithelial cancer of skin | neoplasms | 90/172,069 | 1.19 (0.60, 2.36) | 0.6094 |  | 1.16 (0.59, 2.30) | 0.671 |  | 1.19 (0.50-1.87) | 0.6208 |
| 496.3 | Bronchiectasis | respiratory | 627/143,409 | 1.08 (0.81, 1.42) | 0.6116 |  | 1.00 (0.76, 1.34) | 0.9524 |  | 1.06 (0.77-1.35) | 0.6837 |
| 715 | Other inflammatory spondylopathies | musculoskeletal | 58/171,355 | 1.29 (0.47, 3.58) | 0.6187 |  | 1.16 (0.42, 3.23) | 0.7725 |  | 1.17 (0.15-2.19) | 0.7618 |
| 715.2 | Ankylosing spondylitis | musculoskeletal | 58/171,355 | 1.29 (0.47, 3.58) | 0.6187 |  | 1.16 (0.42, 3.23) | 0.7725 |  | 1.17 (0.15-2.19) | 0.7618 |
| 386.9 | Dizziness and giddiness (Light-headedness and vertigo) | sense organs | 3,839/167,296 | 0.97 (0.85, 1.10) | 0.6188 |  | 0.96 (0.85, 1.09) | 0.573 |  | 0.98 (0.85-1.11) | 0.7630 |
| 379.1 | Scleritis and episcleritis | sense organs | 35/171,572 | 1.37 (0.39, 4.88) | 0.6242 |  | 1.40 (0.39, 4.97) | 0.6045 |  | 1.26 (-0.02-2.54) | 0.7238 |
| 531.1 | Hemorrhage from gastrointestinal ulcer | digestive | 541/158,803 | 0.92 (0.67, 1.28) | 0.6384 |  | 0.91 (0.66, 1.26) | 0.5705 |  | 0.92 (0.59-1.24) | 0.6047 |
| 204.1 | Lymphoid leukemia | neoplasms | 92/171,241 | 0.82 (0.35, 1.92) | 0.6408 |  | 0.83 (0.35, 1.95) | 0.6661 |  | 0.85 (-0.01-1.71) | 0.7031 |
| 427.21 | Atrial fibrillation | circulatory system | 68/168,673 | 0.78 (0.28, 2.20) | 0.6423 |  | 0.80 (0.29, 2.26) | 0.6785 |  | 0.84 (-0.20-1.87) | 0.7393 |
| 446.9 | Arteritis NOS | circulatory system | 45/171,602 | 1.25 (0.48, 3.30) | 0.6457 |  | 1.31 (0.50, 3.45) | 0.5888 |  | 1.31 (0.34-2.28) | 0.5871 |
| 317.1 | Alcoholism | mental disorders | 227/171,966 | 1.12 (0.69, 1.82) | 0.6461 |  | 1.08 (0.66, 1.76) | 0.7574 |  | 1.10 (0.61-1.59) | 0.7108 |
| 317.11 | Alcoholic liver damage | mental disorders | 227/171,966 | 1.12 (0.69, 1.82) | 0.6461 |  | 1.08 (0.66, 1.76) | 0.7574 |  | 1.10 (0.61-1.59) | 0.7108 |
| 558 | Noninfectious gastroenteritis | digestive | 263/170,358 | 1.12 (0.69, 1.81) | 0.6554 |  | 1.08 (0.67, 1.75) | 0.7452 |  | 1.10 (0.62-1.59) | 0.6983 |
| 361 | Retinal detachments and defects | sense organs | 212/171,122 | 0.87 (0.48, 1.60) | 0.6555 |  | 0.87 (0.47, 1.59) | 0.651 |  | 0.88 (0.27-1.48) | 0.6689 |
| 579.2 | Splenomegaly | digestive | 48/169,670 | 0.77 (0.23, 2.53) | 0.6615 |  | 0.82 (0.25, 2.72) | 0.7456 |  | 0.86 (-0.35-2.06) | 0.8008 |
| 500.2 | Pneumoconiosis | respiratory | 70/170,426 | 0.84 (0.38, 1.86) | 0.662 |  | 0.81 (0.36, 1.80) | 0.6009 |  | 0.82 (0.01-1.62) | 0.6192 |
| 595 | Hydronephrosis | genitourinary | 189/165,873 | 0.86 (0.45, 1.67) | 0.6636 |  | 0.91 (0.47, 1.76) | 0.7765 |  | 0.96 (0.29-1.62) | 0.8933 |
| 523.31 | Acute periodontitis | digestive | 145/168,875 | 1.15 (0.62, 2.13) | 0.6643 |  | 1.15 (0.62, 2.15) | 0.6488 |  | 1.26 (0.64-1.88) | 0.4670 |
| 473.1 | Chronic laryngitis | respiratory | 99/168,697 | 1.18 (0.55, 2.54) | 0.6645 |  | 1.19 (0.55, 2.56) | 0.6546 |  | 1.16 (0.39-1.94) | 0.7012 |
| 709 | Diffuse diseases of connective tissue | dermatologic | 31/172,073 | 0.72 (0.17, 3.18) | 0.6686 |  | 0.75 (0.17, 3.33) | 0.7095 |  | 0.79 (-0.69-2.28) | 0.7602 |
| 800.4 | Fracture of patella | injuries & poisonings | 354/168,201 | 0.91 (0.59, 1.40) | 0.6704 |  | 0.89 (0.58, 1.36) | 0.5834 |  | 0.89 (0.46-1.32) | 0.6077 |
| 288.1 | Decreased white blood cell count | hematopoietic | 235/171,681 | 1.12 (0.67, 1.87) | 0.6719 |  | 1.07 (0.64, 1.79) | 0.8017 |  | 1.06 (0.54-1.58) | 0.8226 |
| 288.11 | Neutropenia | hematopoietic | 235/171,681 | 1.12 (0.67, 1.87) | 0.6719 |  | 1.07 (0.64, 1.79) | 0.8017 |  | 1.06 (0.54-1.58) | 0.8226 |
| 70.4 | Chronic hepatitis | infectious diseases | 234/169,432 | 0.88 (0.49, 1.58) | 0.6725 |  | 0.85 (0.47, 1.52) | 0.5807 |  | 0.85 (0.27-1.44) | 0.5969 |
| 433.1 | Occlusion and stenosis of precerebral arteries | circulatory system | 1,525/135,883 | 0.96 (0.81, 1.15) | 0.674 |  | 0.98 (0.82, 1.17) | 0.8428 |  | 0.99 (0.82-1.17) | 0.9496 |
| 523.1 | Gingivitis | digestive | 114/168,875 | 1.16 (0.57, 2.37) | 0.6749 |  | 1.14 (0.56, 2.33) | 0.7143 |  | 1.18 (0.46-1.89) | 0.6575 |
| 53.1 | Herpes zoster with nervous system complications | infectious diseases | 33/169,432 | 0.73 (0.17, 3.18) | 0.6754 |  | 0.73 (0.17, 3.2) | 0.6771 |  | 0.74 (-0.74-2.22) | 0.6946 |
| 592.11 | Acute cystitis | genitourinary | 76/166,019 | 1.17 (0.55, 2.51) | 0.6811 |  | 1.17 (0.55, 2.51) | 0.6781 |  | 1.24 (0.47-2.00) | 0.5836 |
| 198.6 | Secondary malignancy of bone | neoplasms | 84/171,033 | 1.18 (0.53, 2.63) | 0.6815 |  | 1.18 (0.53, 2.61) | 0.6901 |  | 1.20 (0.40-2.01) | 0.6522 |
| 411.2 | Myocardial infarction | circulatory system | 1,121/149,240 | 1.04 (0.85, 1.28) | 0.6818 |  | 1.06 (0.86, 1.29) | 0.6066 |  | 1.10 (0.89-1.31) | 0.3719 |
| 522 | Diseases of pulp and periapical tissues | digestive | 359/168,875 | 0.91 (0.57, 1.44) | 0.6844 |  | 0.91 (0.57, 1.44) | 0.6804 |  | 0.93 (0.47-1.40) | 0.7706 |
| 433.12 | Cerebral atherosclerosis | circulatory system | 1,237/135,883 | 0.96 (0.79, 1.17) | 0.6963 |  | 0.98 (0.81, 1.19) | 0.8358 |  | 1.00 (0.81-1.19) | 0.9984 |
| 790.6 | Other abnormal blood chemistry | symptoms | 129/172,058 | 1.12 (0.62, 2.03) | 0.6966 |  | 1.21 (0.67, 2.20) | 0.5277 |  | 1.21 (0.61-1.81) | 0.5370 |
| 444 | Arterial embolism and thrombosis | circulatory system | 46/171,602 | 0.79 (0.24, 2.61) | 0.6992 |  | 0.81 (0.24, 2.69) | 0.7317 |  | 0.81 (-0.39-2.01) | 0.7339 |
| 165 | Cancer within the respiratory system | neoplasms | 4,006/168,150 | 0.98 (0.87, 1.10) | 0.704 |  | 0.96 (0.86, 1.08) | 0.4753 |  | 0.98 (0.86-1.09) | 0.6664 |
| 394.4 | Acute rheumatic heart disease | circulatory system | 265/171,662 | 1.09 (0.71, 1.65) | 0.7042 |  | 1.06 (0.69, 1.61) | 0.8013 |  | 1.04 (0.62-1.47) | 0.8481 |
| 803.1 | Fracture of humerus | injuries & poisonings | 265/168,201 | 1.09 (0.69, 1.74) | 0.7056 |  | 1.09 (0.69, 1.74) | 0.7104 |  | 1.13 (0.66-1.59) | 0.6175 |
| 599.2 | Retention of urine | genitourinary | 585/171,420 | 0.95 (0.73, 1.24) | 0.708 |  | 0.96 (0.74, 1.25) | 0.7556 |  | 0.97 (0.70-1.24) | 0.8228 |
| 569 | Other disorders of intestine | digestive | 382/170,043 | 0.92 (0.59, 1.43) | 0.711 |  | 0.90 (0.58, 1.40) | 0.6365 |  | 0.91 (0.46-1.36) | 0.6823 |
| 379.2 | Disorders of vitreous body | sense organs | 197/171,572 | 1.00 (0.66, 1.85) | 0.7127 |  | 1.12 (0.67, 1.88) | 0.6723 |  | 1.15 (0.63-1.67) | 0.5977 |
| 242 | Thyrotoxicosis with or without goiter | endocrine/  metabolic | 172/171,651 | 0.88 (0.45, 1.72) | 0.7156 |  | 0.85 (0.44, 1.67) | 0.6413 |  | 0.86 (0.19-1.53) | 0.6555 |
| 358 | Myoneural disorders | neurological | 36/171,929 | 1.25 (0.36, 4.30) | 0.7208 |  | 1.32 (0.38, 4.52) | 0.6629 |  | 1.27 (0.03-2.51) | 0.7043 |
| 573.7 | Abnormal results of function study of liver | digestive | 408/170,077 | 1.07 (0.73, 1.58) | 0.7224 |  | 1.09 (0.74, 1.61) | 0.6582 |  | 1.09 (0.70-1.48) | 0.6694 |
| 401.3 | Other hypertensive complications | circulatory system | 168/87,647 | 1.00 (0.64, 1.88) | 0.7226 |  | 1.16 (0.68, 1.99) | 0.5797 |  | 0.91 (-0.35-2.16) | 0.8760 |
| 70.2 | Viral hepatitis B | infectious diseases | 496/169,432 | 1.07 (0.73, 1.58) | 0.7277 |  | 1.05 (0.71, 1.55) | 0.7966 |  | 1.06 (0.67-1.45) | 0.7738 |
| 766 | Neuralgia, neuritis, and radiculitis NOS | symptoms | 1,108/170,730 | 0.96 (0.74, 1.23) | 0.7284 |  | 0.96 (0.74, 1.24) | 0.7351 |  | 0.98 (0.73-1.24) | 0.8940 |
| 149.9 | Cancer of of nasal cavities | neoplasms | 37/171,523 | 1.21 (0.41, 3.58) | 0.7299 |  | 1.20 (0.41, 3.56) | 0.7369 |  | 1.15 (0.04-2.26) | 0.8019 |
| 250.1 | Type 1 diabetes | endocrine/  metabolic | 204/156,334 | 0.91 (0.52, 1.57) | 0.7319 |  | 0.97 (0.56, 1.69) | 0.9248 |  | 1.49 (0.71-2.27) | 0.3194 |
| 195.1 | Malignant neoplasm, other | neoplasms | 178/171,033 | 0.91 (0.53, 1.57) | 0.7385 |  | 0.91 (0.53, 1.56) | 0.7233 |  | 0.94 (0.39-1.48) | 0.8209 |
| 165.1 | Cancer of bronchus; lung | neoplasms | 3,980/168,150 | 0.98 (0.88, 1.10) | 0.7419 |  | 0.96 (0.86, 1.08) | 0.5092 |  | 0.98 (0.86-1.09) | 0.7014 |
| 426.2 | Atrioventricular [AV] block | circulatory system | 70/168,673 | 0.88 (0.40, 1.95) | 0.7473 |  | 0.91 (0.41, 2.02) | 0.8133 |  | 0.93 (0.13-1.73) | 0.8576 |
| 374.1 | Ectropion or entropion | sense organs | 140/169,380 | 1.00 (0.63, 1.91) | 0.7476 |  | 1.09 (0.63, 1.90) | 0.7608 |  | 1.13 (0.57-1.69) | 0.6666 |
| 465.4 | Acute laryngitis and tracheitis | respiratory | 2,030/154,332 | 0.97 (0.81, 1.16) | 0.7515 |  | 0.96 (0.80, 1.15) | 0.6392 |  | 0.96 (0.78-1.14) | 0.6762 |
| 241 | Nontoxic nodular goiter | endocrine/  metabolic | 303/171,651 | 0.92 (0.54, 1.56) | 0.7518 |  | 0.92 (0.54, 1.56) | 0.7622 |  | 0.91 (0.38-1.44) | 0.7234 |
| 578.8 | Hemorrhage of rectum and anus | digestive | 132/169,670 | 0.89 (0.44, 1.81) | 0.7538 |  | 0.89 (0.44, 1.80) | 0.7446 |  | 0.87 (0.16-1.58) | 0.7099 |
| 510 | Other diseases of lung | respiratory | 108/172,095 | 1.00 (0.57, 2.19) | 0.7576 |  | 1.08 (0.55, 2.13) | 0.8182 |  | 1.09 (0.41-1.77) | 0.8068 |
| 612 | Breast conditions, congenital or relating to hormones | genitourinary | 39/172,098 | 0.84 (0.25, 2.82) | 0.7765 |  | 0.84 (0.25, 2.83) | 0.7793 |  | 0.91 (-0.31-2.13) | 0.8770 |
| 612.2 | Hypertrophy of breast (Gynecomastia) | genitourinary | 39/172,098 | 0.84 (0.25, 2.82) | 0.7765 |  | 0.84 (0.25, 2.83) | 0.7793 |  | 0.91 (-0.31-2.13) | 0.8770 |
| 155 | Cancer of liver and intrahepatic bile duct | neoplasms | 2113/162,514 | 0.98 (0.83, 1.14) | 0.7767 |  | 0.97 (0.83, 1.13) | 0.6765 |  | 0.98 (0.82-1.14) | 0.8420 |
| 352.1 | Trigeminal nerve disorders [CN5] | neurological | 120/171,360 | 0.91 (0.48, 1.74) | 0.7767 |  | 0.91 (0.48, 1.74) | 0.7864 |  | 0.95 (0.30-1.59) | 0.8670 |
| 764 | Sciatica | symptoms | 405/170,730 | 0.95 (0.65, 1.38) | 0.7792 |  | 0.97 (0.66, 1.41) | 0.866 |  | 0.98 (0.60-1.36) | 0.9265 |
| 340 | Migraine | neurological | 174/169,121 | 0.92 (0.50, 1.69) | 0.7801 |  | 0.90 (0.49, 1.66) | 0.7431 |  | 0.93 (0.31-1.54) | 0.8072 |
| 575.2 | Obstruction of bile duct | digestive | 189/158,889 | 0.93 (0.56, 1.56) | 0.7893 |  | 0.95 (0.57, 1.59) | 0.8579 |  | 1.00 (0.48-1.51) | 0.9917 |
| 586.4 | Stricture/obstruction of ureter | genitourinary | 125/166,533 | 1.00 (0.55, 2.17) | 0.7934 |  | 1.09 (0.55, 2.15) | 0.8143 |  | 1.11 (0.42-1.79) | 0.7690 |
| 471 | Nasal polyps | respiratory | 289/168,697 | 1.07 (0.65, 1.75) | 0.7969 |  | 1.09 (0.67, 1.79) | 0.7233 |  | 1.15 (0.66-1.64) | 0.5735 |
| 369 | Infection of the eye | sense organs | 993/169,380 | 0.97 (0.74, 1.26) | 0.7989 |  | 0.97 (0.74, 1.26) | 0.816 |  | 0.95 (0.69-1.22) | 0.7139 |
| 913 | Toxic effect of venom | injuries & poisonings | 179/172,024 | 0.93 (0.54, 1.62) | 0.807 |  | 0.91 (0.52, 1.59) | 0.7475 |  | 0.93 (0.37-1.48) | 0.7888 |
| 250.23 | Type 2 diabetes with ophthalmic manifestations | endocrine/  metabolic | 35/156,334 | 1.16 (0.34, 3.98) | 0.808 |  | 1.29 (0.38, 4.42) | 0.6845 |  | NA | NA |
| 427.5 | Arrhythmia (cardiac) NOS | circulatory system | 2164/168,673 | 0.98 (0.84, 1.15) | 0.8083 |  | 0.98 (0.84, 1.14) | 0.7883 |  | 1.00 (0.84-1.15) | 0.9682 |
| 145.3 | Cancer of major salivary glands | neoplasms | 37/171,523 | 0.84 (0.19, 3.65) | 0.8201 |  | 0.88 (0.20, 3.82) | 0.8647 |  | 0.83 (-0.64-2.31) | 0.8054 |
| 797 | Shock | symptoms | 158/172,045 | 1.06 (0.63, 1.78) | 0.8282 |  | 1.09 (0.65, 1.84) | 0.739 |  | 1.05 (0.53-1.58) | 0.8454 |
| 425 | Cardiomyopathy | circulatory system | 458/171,261 | 0.96 (0.68, 1.36) | 0.8324 |  | 0.99 (0.70, 1.40) | 0.9504 |  | 0.98 (0.63-1.33) | 0.8961 |
| 228 | Hemangioma and lymphangioma, any site | neoplasms | 157/172,046 | 1.07 (0.57, 2.03) | 0.8325 |  | 1.05 (0.56, 1.99) | 0.8763 |  | 1.10 (0.46-1.74) | 0.7698 |
| 427.7 | Tachycardia NOS | circulatory system | 191/168,673 | 1.05 (0.64, 1.74) | 0.8345 |  | 1.00 (0.61, 1.68) | 0.9544 |  | 1.05 (0.55-1.56) | 0.8363 |
| 386 | Vertiginous syndromes and other disorders of vestibular system | sense organs | 1274/167,296 | 0.98 (0.79, 1.21) | 0.8353 |  | 0.98 (0.79, 1.20) | 0.8202 |  | 0.98 (0.77-1.19) | 0.8477 |
| 916 | Contusion | injuries & poisonings | 568/171,635 | 1.04 (0.73, 1.47) | 0.8354 |  | 1.04 (0.73, 1.47) | 0.8308 |  | 1.08 (0.73-1.44) | 0.6522 |
| 789 | Nausea and vomiting | symptoms | 89/172,114 | 1.08 (0.53, 2.21) | 0.836 |  | 1.05 (0.51, 2.15) | 0.897 |  | 1.09 (0.36-1.81) | 0.8230 |
| 366 | Cataract | sense organs | 5,965/166,238 | 1.00 (0.92, 1.10) | 0.8369 |  | 1.00 (0.93, 1.10) | 0.8094 |  | 1.02 (0.93-1.11) | 0.6802 |
| 79 | Viral infection | infectious diseases | 34/169,432 | 1.14 (0.32, 4.04) | 0.837 |  | 1.15 (0.33, 4.09) | 0.8257 |  | 1.19 (-0.09-2.47) | 0.7932 |
| 195.3 | Malignant neoplasm of head, face, and neck | neoplasms | 49/171,033 | 1.00 (0.39, 3.21) | 0.8429 |  | 1.09 (0.38, 3.15) | 0.8731 |  | 1.08 (0.02-2.15) | 0.8859 |
| 577.1 | Acute pancreatitis | digestive | 654/171,480 | 0.97 (0.71, 1.32) | 0.8441 |  | 1.00 (0.74, 1.38) | 0.9266 |  | 1.04 (0.73-1.35) | 0.7883 |
| 386.1 | Meniere's disease | sense organs | 40/167,296 | 0.89 (0.26, 2.98) | 0.8459 |  | 0.85 (0.25, 2.88) | 0.7957 |  | 0.85 (-0.38-2.07) | 0.7914 |
| 204.12 | Lymphoid leukemia, chronic | neoplasms | 38/171,241 | 0.89 (0.26, 3.07) | 0.8481 |  | 0.90 (0.26, 3.11) | 0.8665 |  | 0.93 (-0.32-2.18) | 0.9126 |
| 331.1 | Hydrocephalus | neurological | 73/170,510 | 0.91 (0.36, 2.32) | 0.8496 |  | 0.92 (0.36, 2.34) | 0.8609 |  | 0.93 (-0.01-1.86) | 0.8765 |
| 496.1 | Emphysema | respiratory | 7,156/143,409 | 0.99 (0.91, 1.08) | 0.8502 |  | 0.95 (0.88, 1.04) | 0.2801 |  | 0.96 (0.80-1.12) | 0.6150 |
| 159.2 | Malignant neoplasm of small intestine, including duodenum | neoplasms | 85/162,514 | 1.09 (0.46, 2.56) | 0.8515 |  | 1.08 (0.46, 2.54) | 0.8632 |  | 1.07 (0.21-1.93) | 0.8758 |
| 378 | Strabismus and other disorders of binocular eye movements | sense organs | 79/171,572 | 1.08 (0.48, 2.45) | 0.8519 |  | 1.00 (0.49, 2.49) | 0.819 |  | 1.17 (0.35-1.98) | 0.7120 |
| 599 | Other symptoms/disorders or the urinary system | genitourinary | 204/171,420 | 1.05 (0.63, 1.74) | 0.852 |  | 1.07 (0.64, 1.77) | 0.8054 |  | 1.08 (0.57-1.58) | 0.7799 |
| 381.11 | Suppurative and unspecified otitis media | sense organs | 348/171,802 | 1.04 (0.66, 1.64) | 0.8535 |  | 1.04 (0.66, 1.64) | 0.8559 |  | 1.11 (0.65-1.56) | 0.6603 |
| 145.2 | Cancer of tongue | neoplasms | 59/171,523 | 0.92 (0.35, 2.38) | 0.8559 |  | 0.88 (0.34, 2.30) | 0.8009 |  | 0.91 (-0.05-1.87) | 0.8500 |
| 440 | Atherosclerosis | circulatory system | 223/171,602 | 0.95 (0.57, 1.59) | 0.8576 |  | 0.98 (0.59, 1.63) | 0.9281 |  | 0.99 (0.48-1.50) | 0.9661 |
| 580.3 | Nephritis and nephropathy without mention of glomerulonephritis | genitourinary | 256/166,533 | 0.96 (0.57, 1.59) | 0.8632 |  | 0.92 (0.55, 1.54) | 0.7529 |  | 0.87 (0.36-1.39) | 0.6056 |
| 580.32 | Nephritis and nephropathy with pathological lesion | genitourinary | 256/166,533 | 0.96 (0.57, 1.59) | 0.8632 |  | 0.92 (0.55, 1.54) | 0.7529 |  | 0.87 (0.36-1.39) | 0.6056 |
| 745 | Pain in joint | musculoskeletal | 174/172,029 | 1.05 (0.60, 1.85) | 0.8646 |  | 1.09 (0.62, 1.93) | 0.7612 |  | 1.09 (0.51-1.66) | 0.7764 |
| 503 | Pulmonary congestion and hypostasis | respiratory | 68/170,426 | 1.07 (0.50, 2.29) | 0.8673 |  | 1.04 (0.48, 2.24) | 0.9157 |  | 0.98 (0.21-1.75) | 0.9605 |
| 720 | Spinal stenosis | musculoskeletal | 591/171,526 | 0.97 (0.70, 1.35) | 0.8687 |  | 1.00 (0.72, 1.39) | 0.9735 |  | 1.03 (0.70-1.35) | 0.8810 |
| 369.5 | Conjunctivitis, infectious | sense organs | 952/169,380 | 0.98 (0.75, 1.28) | 0.8695 |  | 0.98 (0.75, 1.28) | 0.8809 |  | 0.96 (0.69-1.23) | 0.7774 |
| 377 | Disorders of optic nerve and visual pathways | sense organs | 116/171,572 | 1.06 (0.54, 2.08) | 0.8711 |  | 1.05 (0.53, 2.07) | 0.8889 |  | 1.02 (0.34-1.70) | 0.9508 |
| 333 | Extrapyramidal disease and abnormal movement disorders | neurological | 62/170,510 | 1.07 (0.45, 2.57) | 0.8714 |  | 1.07 (0.45, 2.58) | 0.8716 |  | 1.16 (0.28-2.04) | 0.7391 |
| 800.1 | Fracture of neck of femur | injuries & poisonings | 299/168,201 | 0.97 (0.67, 1.40) | 0.8755 |  | 0.95 (0.66, 1.37) | 0.774 |  | 0.93 (0.56-1.30) | 0.7101 |
| 611 | Abnormal findings on mammogram or breast exam | genitourinary | 31/172,098 | 1.00 (0.31, 3.87) | 0.8777 |  | 1.00 (0.32, 3.93) | 0.8672 |  | 1.11 (-0.19-2.41) | 0.8766 |
| 611.3 | Lump or mass in breast | genitourinary | 31/172,098 | 1.00 (0.31, 3.87) | 0.8777 |  | 1.00 (0.32, 3.93) | 0.8672 |  | 1.11 (-0.19-2.41) | 0.8766 |
| 807 | Fracture of ribs | injuries & poisonings | 864/168,201 | 0.98 (0.75, 1.28) | 0.8827 |  | 0.99 (0.75, 1.29) | 0.9152 |  | 0.99 (0.72-1.25) | 0.9129 |
| 751.2 | Congenital anomalies of urinary system | congenital anomalies | 49/172,138 | 0.92 (0.27, 3.07) | 0.8876 |  | 0.89 (0.27, 2.99) | 0.8539 |  | 0.85 (-0.37-2.06) | 0.7875 |
| 159.4 | Malignant neoplasm of retroperitoneum and peritoneum | neoplasms | 41/162,514 | 0.92 (0.27, 3.11) | 0.8895 |  | 0.92 (0.27, 3.13) | 0.8968 |  | 0.89 (-0.34-2.12) | 0.8515 |
| 368 | Visual disturbances | sense organs | 53/172,146 | 0.93 (0.32, 2.68) | 0.8901 |  | 0.97 (0.33, 2.80) | 0.9525 |  | 1.03 (-0.03-2.10) | 0.9505 |
| 442.1 | Aortic aneurysm | circulatory system | 178/171,602 | 1.04 (0.60, 1.80) | 0.8901 |  | 1.06 (0.61, 1.84) | 0.8242 |  | 1.01 (0.46-1.56) | 0.9679 |
| 751 | Genitourinary congenital anomalies | congenital anomalies | 51/172,138 | 0.93 (0.28, 3.1) | 0.9027 |  | 0.90 (0.27, 3.01) | 0.8644 |  | 0.87 (-0.34-2.08) | 0.8225 |
| 145 | Cancer of mouth | neoplasms | 159/171,523 | 0.97 (0.55, 1.71) | 0.9032 |  | 0.96 (0.54, 1.69) | 0.8813 |  | 0.95 (0.38-1.52) | 0.8632 |
| 706 | Diseases of sebaceous glands | dermatologic | 41/172,162 | 1.08 (0.31, 3.81) | 0.9049 |  | 1.08 (0.31, 3.83) | 0.9038 |  | 0.98 (-0.31-2.28) | 0.9779 |
| 706.2 | Sebaceous cyst | dermatologic | 41/172,162 | 1.08 (0.31, 3.81) | 0.9049 |  | 1.08 (0.31, 3.83) | 0.9038 |  | 0.98 (-0.31-2.28) | 0.9779 |
| 214 | Lipoma | neoplasms | 400/171,798 | 1.03 (0.67, 1.57) | 0.9055 |  | 1.02 (0.66, 1.55) | 0.9425 |  | 1.03 (0.60-1.45) | 0.9004 |
| 370.1 | Corneal ulcer | sense organs | 91/169,380 | 0.96 (0.45, 2.04) | 0.9061 |  | 0.92 (0.43, 1.97) | 0.8306 |  | 0.97 (0.20-1.73) | 0.9310 |
| 817 | Concussion | injuries & poisonings | 199/168,416 | 1.03 (0.61, 1.74) | 0.9072 |  | 1.04 (0.62, 1.75) | 0.8867 |  | 1.06 (0.54-1.59) | 0.8172 |
| 535.6 | Duodenitis | digestive | 56/159,961 | 0.93 (0.28, 3.10) | 0.9076 |  | 0.91 (0.27, 3.03) | 0.876 |  | 0.99 (-0.22-2.20) | 0.9860 |
| 426 | Cardiac conduction disorders | circulatory system | 126/168,673 | 1.03 (0.57, 1.86) | 0.915 |  | 1.04 (0.58, 1.88) | 0.8903 |  | 1.09 (0.49-1.68) | 0.7859 |
| 157 | Pancreatic cancer | neoplasms | 464/162,514 | 0.98 (0.70, 1.38) | 0.9151 |  | 0.98 (0.70, 1.38) | 0.9083 |  | 0.99 (0.65-1.34) | 0.9728 |
| 358.1 | Myasthenia gravis | neurological | 34/171,929 | 0.93 (0.21, 4.07) | 0.9216 |  | 0.98 (0.22, 4.30) | 0.9804 |  | 0.95 (-0.53-2.43) | 0.9432 |
| 333.8 | Other degenerative diseases of the basal ganglia | neurological | 31/170,510 | 1.06 (0.31, 3.63) | 0.9278 |  | 1.02 (0.30, 3.51) | 0.9748 |  | 1.09 (-0.15-2.33) | 0.8946 |
| 575.9 | Nonspecific abnormal findings on radiological and other examination of biliary tract | digestive | 143/158,889 | 0.97 (0.51, 1.83) | 0.9278 |  | 0.97 (0.51, 1.82) | 0.9155 |  | 0.99 (0.35-1.63) | 0.9741 |
| 571.81 | Portal hypertension | digestive | 31/170,077 | 1.07 (0.24, 4.74) | 0.9313 |  | 1.04 (0.23, 4.64) | 0.9562 |  | 1.15 (-0.35-2.64) | 0.8583 |
| 272.11 | Hypercholesterolemia | endocrine/  metabolic | 97/170,604 | 1.03 (0.49, 2.19) | 0.9314 |  | 1.08 (0.51, 2.29) | 0.8449 |  | 1.11 (0.35-1.86) | 0.7906 |
| 575.1 | Cholangitis | digestive | 160/158,889 | 0.98 (0.56, 1.69) | 0.9318 |  | 0.99 (0.57, 1.72) | 0.9758 |  | 1.02 (0.46-1.57) | 0.9550 |
| 281 | Other deficiency anemia | hematopoietic | 47/171,112 | 1.04 (0.40, 2.71) | 0.9347 |  | 1.05 (0.40, 2.73) | 0.9227 |  | 1.15 (0.19-2.11) | 0.7748 |
| 479 | Other upper respiratory disease | respiratory | 148/168,697 | 0.98 (0.53, 1.82) | 0.947 |  | 0.93 (0.50, 1.72) | 0.8098 |  | 0.96 (0.33-1.58) | 0.8853 |
| 420.3 | Endocarditis | circulatory system | 227/171,261 | 1.00 (0.64, 1.60) | 0.9553 |  | 1.02 (0.64, 1.60) | 0.9487 |  | 1.00 (0.54-1.46) | 0.9966 |
| 395.1 | Nonrheumatic mitral valve disorders | circulatory system | 448/171,261 | 0.99 (0.70, 1.40) | 0.9565 |  | 1.02 (0.72, 1.44) | 0.9973 |  | 1.00 (0.65-1.35) | 0.9875 |
| 425.1 | Primary/intrinsic cardiomyopathies | circulatory system | 30/171,662 | 0.96 (0.22, 4.25) | 0.9565 |  | 1.00 (0.23, 4.42) | 0.9259 |  | 0.96 (-0.53-2.46) | 0.9606 |
| 585 | Renal failure | genitourinary | 1,530/166,533 | 1.00 (0.83, 1.19) | 0.9582 |  | 1.00 (0.84, 1.21) | 0.9017 |  | 0.99 (0.81-1.17) | 0.9233 |
| 714 | Rheumatoid arthritis and other inflammatory polyarthropathies | musculoskeletal | 730/171,355 | 0.99 (0.76, 1.30) | 0.9595 |  | 0.99 (0.76, 1.30) | 0.9694 |  | 1.01 (0.74-1.28) | 0.9642 |
| 357 | Inflammatory and toxic neuropathy | neurological | 214/171,929 | 0.99 (0.59, 1.66) | 0.962 |  | 0.99 (0.59, 1.67) | 0.9783 |  | 0.99 (0.47-1.51) | 0.9647 |
| 714.1 | Rheumatoid arthritis | musculoskeletal | 729/171,355 | 0.99 (0.76, 1.30) | 0.9639 |  | 1.00 (0.76, 1.30) | 0.9755 |  | 1.01 (0.74-1.28) | 0.9546 |
| 480.5 | Bronchopneumonia and lung abscess | respiratory | 1,191/156,992 | 1.00 (0.81, 1.22) | 0.9688 |  | 0.98 (0.80, 1.20) | 0.8536 |  | 0.99 (0.79-1.19) | 0.9307 |
| 411.3 | Angina pectoris | circulatory system | 810/149,240 | 1.00 (0.79, 1.28) | 0.971 |  | 1.03 (0.81, 1.31) | 0.7925 |  | 1.05 (0.81-1.29) | 0.6820 |
| 204.2 | Myeloid leukemia | neoplasms | 84/171,241 | NA | 0.9712 |  | NA | 0.9711 |  | NA | 0.9806 |
| 573 | Other disorders of liver | digestive | 878/170,077 | 1.00 (0.76, 1.31) | 0.9722 |  | 0.98 (0.75, 1.29) | 0.8972 |  | 1.01 (0.73-1.28) | 0.9649 |
| 195 | Cancer, suspected or other | neoplasms | 230/171,033 | 0.99 (0.62, 1.59) | 0.974 |  | 0.98 (0.61, 1.58) | 0.9457 |  | 1.01 (0.53-1.48) | 0.9831 |
| 442.11 | Abdominal aortic aneurysm | circulatory system | 48/171,602 | 0.98 (0.34, 2.83) | 0.9745 |  | 1.00 (0.35, 2.90) | 0.991 |  | 1.03 (-0.03-2.09) | 0.9564 |
| 440.2 | Atherosclerosis of the extremities | circulatory system | 200/171,602 | 0.99 (0.58, 1.69) | 0.9766 |  | 1.00 (0.60, 1.72) | 0.9581 |  | 1.04 (0.50-1.57) | 0.8979 |
| 164 | Cancer of intrathoracic organs | neoplasms | 77/168,150 | 0.99 (0.39, 2.52) | 0.9785 |  | 0.96 (0.38, 2.46) | 0.9376 |  | 1.04 (0.10-1.98) | 0.9330 |
| 531.2 | Gastric ulcer | digestive | 1,337/158,803 | 1.00 (0.81, 1.22) | 0.9794 |  | 0.97 (0.79, 1.20) | 0.8077 |  | 1.00 (0.79-1.20) | 0.9792 |
| 204.21 | Myeloid leukemia, acute | neoplasms | 45/171,241 | NA | 0.982 |  | NA | 0.9882 |  | NA | 0.9879 |
| 473 | Diseases of the larynx and vocal cords | respiratory | 432/168,697 | 1.00 (0.65, 1.53) | 0.9829 |  | 1.00 (0.65, 1.54) | 0.9892 |  | 1.00 (0.56-1.43) | 0.9897 |
| 381 | Otitis media and Eustachian tube disorders | sense organs | 391/171,802 | 1.00 (0.64, 1.55) | 0.9847 |  | 1.00 (0.64, 1.55) | 0.9858 |  | 1.05 (0.60-1.49) | 0.8402 |
| 381.1 | Otitis media | sense organs | 391/171,802 | 1.00 (0.64, 1.55) | 0.9847 |  | 1.00 (0.64, 1.55) | 0.9858 |  | 1.05 (0.60-1.49) | 0.8402 |
| 70.3 | Viral hepatitis C | infectious diseases | 32/171,523 | NA | 0.9879 |  | NA | 0.9876 |  | NA | 0.9915 |
| 149.3 | Cancer of hypopharynx | neoplasms | 39/169,432 | NA | 0.9879 |  | NA | 0.9917 |  | NA | 0.9873 |
| 245 | Thyroiditis | endocrine/  metabolic | 45/171,651 | NA | 0.9882 |  | NA | 0.988 |  | NA | 0.9878 |
| 592.1 | Cystitis | genitourinary | 357/166,019 | 1.00 (0.68, 1.47) | 0.9899 |  | 0.99 (0.67, 1.47) | 0.9749 |  | 1.06 (0.67-1.45) | 0.7793 |
| 751.21 | Cystic kidney disease | congenital anomalies | 45/172,138 | 1.00 (0.30, 3.39) | 0.9899 |  | 0.98 (0.29, 3.30) | 0.9724 |  | 0.91 (-0.30-2.13) | 0.8852 |
| 306 | Other mental disorder | mental disorders | 40/170,474 | 0.99 (0.29, 3.44) | 0.9928 |  | 0.97 (0.28, 3.38) | 0.9621 |  | 1.09 (-0.16-2.34) | 0.8914 |
| 306.9 | Tension headache | mental disorders | 40/170,474 | 0.99 (0.29, 3.44) | 0.9928 |  | 0.97 (0.28, 3.38) | 0.9621 |  | 1.09 (-0.16-2.34) | 0.8914 |
| 149.2 | Cancer of nasopharynx | neoplasms | 282/171,523 | 1.00 (0.63, 1.59) | 0.9977 |  | 0.97 (0.61, 1.55) | 0.909 |  | 1.01 (0.53-1.48) | 0.9819 |
| 560.3 | Peritoneal or intestinal adhesions | digestive | 46/170,358 | 1.00 (0.35, 2.89) | 0.9987 |  | 1.04 (0.36, 3.02) | 0.937 |  | 1.12 (0.05-2.18) | 0.8363 |

The phenome-wide *P* value was Bonferroni corrected (0.05 divided by the number of diseases in the group), and *P*<9.92*10^-5^ (0.05/504) for men or 9.33*10^-5^ (0.05/536) for women was considered to be statistically significant.

**Model 1:** adjusted for age, study area, highest education, household income, and household size.

**Model 2:** adjusted for covariates in model 1, alcohol drinking, smoking, dietary habits, physical activity, and BMI.

**Model 3:** adjusted for covariates in model 2, history of diabetes, hypertension, respiratory disease, CVD, or cancer at baseline, family history of the analyzed disease (adjusted for only in corresponding analysis), and self-reported satisfaction level of life.

**Table S7 PheWAS results of marital status (living with vs. without a spouse) with 536 diseases in 302,521 women**

| **Phecode** | **Diseases** | **Group** | **Case/conctrol** | **Model 1** | |  | **Model 2** | |  | **Model 3** | |
| --- | --- | --- | --- | --- | --- | --- | --- | --- | --- | --- | --- |
|  |  |  |  | **OR (95%CI)** | ***P*** |  | **OR (95%CI)** | ***P*** |  | **OR (95%CI)** | ***P*** |
| 465 | Acute upper respiratory infections of multiple or unspecified sites | respiratory | 27,644/217,033 | 0.85 (0.80, 0.90) | 1.95E-08 |  | 0.85 (0.80, 0.90) | 2.12E-08 |  | 0.85 (0.79-0.90) | 1.86E-08 |
| 535 | Gastritis and duodenitis | digestive | 19,705/223,512 | 0.87 (0.82, 0.92) | 1.80E-07 |  | 0.86 (0.82, 0.91) | 5.03E-08 |  | 0.86 (0.81-0.91) | 2.08E-08 |
| 386.9 | Dizziness and giddiness (Light-headedness and vertigo) | sense organs | 8,613/233,256 | 0.83 (0.77, 0.89) | 8.24E-07 |  | 0.83 (0.77, 0.90) | 1.34E-06 |  | 0.83 (0.75-0.90) | 8.48E-07 |
| 433.3 | Cerebral ischemia | circulatory system | 23,238/193,417 | 0.90 (0.86, 0.94) | 5.61E-06 |  | 0.90 (0.86, 0.94) | 1.02E-05 |  | 0.90 (0.86-0.95) | 1.43E-05 |
| 433.31 | Transient cerebral ischemia | circulatory system | 6,206/193,417 | 0.84 (0.77, 0.91) | 3.48E-05 |  | 0.84 (0.77, 0.91) | 5.26E-05 |  | 0.84 (0.76-0.93) | 5.87E-05 |
| 295.1 | Schizophrenia | mental disorders | 1,858/240,702 | 1.36 (1.17, 1.57) | 3.88E-05 |  | 1.33 (1.15, 1.54) | 9.79E-05 |  | 1.24 (1.10-1.39) | 0.0035 |
| 295 | Schizophrenia and other psychotic disorders | mental disorders | 1,860/240,702 | 1.35 (1.17, 1.56) | 4.20E-05 |  | 1.33 (1.15, 1.54) | 0.0001 |  | 1.24 (1.10-1.39) | 0.0037 |
| 480 | Pneumonia | respiratory | 16,987/222,776 | 0.90 (0.86, 0.95) | 4.52E-05 |  | 0.90 (0.86, 0.94) | 1.27E-05 |  | 0.90 (0.85-0.95) | 1.03E-05 |
| 497 | Bronchitis | respiratory | 9,225/211,409 | 0.86 (0.80, 0.93) | 9.30E-05 |  | 0.86 (0.79, 0.92) | 5.10E-05 |  | 0.85 (0.77-0.93) | 3.64E-05 |
| 411 | Coronary atherosclerosis | circulatory system | 24,954/209,210 | 0.92 (0.89, 0.96) | 0.0002 |  | 0.92 (0.88, 0.96) | 7.42E-05 |  | 0.93 (0.89-0.97) | 0.0010 |
| 411.4 | Acute pharyngitis | circulatory system | 5,350/217,033 | 0.79 (0.70, 0.89) | 0.0002 |  | 0.79 (0.70, 0.90) | 7.55E-05 |  | 0.79 (0.67-0.92) | 0.0002 |
| 465.2 | Ischemic Heart Disease | respiratory | 34,598/209,210 | 0.93 (0.90, 0.97) | 0.0002 |  | 0.93 (0.90, 0.96) | 0.0002 |  | 0.94 (0.90-0.98) | 0.0015 |
| 591 | Urinary tract infection | genitourinary | 4055/230026 | 0.81 (0.72, 0.90) | 0.0002 |  | 0.81 (0.72, 0.90) | 0.0002 |  | 0.81 (0.70-0.93) | 0.0003 |
| 592 | Cystitis and urethritis | genitourinary | 5,327/230,026 | 0.81 (0.72, 0.90) | 0.0002 |  | 0.81 (0.72, 0.91) | 0.0003 |  | 0.81 (0.70-0.93) | 0.0004 |
| 366 | Cataract | sense organs | 10,626/234,082 | 0.91 (0.86, 0.96) | 0.0004 |  | 0.90 (0.85, 0.95) | 0.0002 |  | 0.90 (0.85-0.96) | 0.0002 |
| 594 | Urinary calculus | genitourinary | 6,885/237,706 | 0.84 (0.77, 0.93) | 0.0004 |  | 0.85 (0.77, 0.93) | 0.0008 |  | 0.85 (0.75-0.95) | 0.0009 |
| 433.1 | Occlusion and stenosis of precerebral arteries | circulatory system | 2,421/193,417 | 0.80 (0.70, 0.91) | 0.0005 |  | 0.79 (0.69, 0.90) | 0.0004 |  | 0.79 (0.66-0.92) | 0.0005 |
| 433.12 | Cerebral atherosclerosis | circulatory system | 2,043/193,417 | 0.78 (0.68, 0.90) | 0.0007 |  | 0.78 (0.67, 0.90) | 0.0006 |  | 0.78 (0.64-0.92) | 0.0006 |
| 592.2 | Urethritis and urethral syndrome | genitourinary | 4,241/230,026 | 0.80 (0.70, 0.91) | 0.0009 |  | 0.81 (0.71, 0.92) | 0.0013 |  | 0.81 (0.68-0.94) | 0.0016 |
| 947 | Urticaria | dermatologic | 778/241,916 | 0.57 (0.40, 0.80) | 0.0012 |  | 0.57 (0.40, 0.80) | 0.0011 |  | 0.57 (0.22-0.91) | 0.0012 |
| 819 | Skull and face fracture and other intercranial injury | injuries & poisonings | 2,228/242,268 | 1.23 (1.08, 1.40) | 0.0015 |  | 1.22 (1.08, 1.39) | 0.0021 |  | 1.18 (1.05-1.31) | 0.0126 |
| 740 | Osteoarthrosis | musculoskeletal | 12,134/232,574 | 0.90 (0.85, 0.96) | 0.0018 |  | 0.92 (0.86, 0.98) | 0.009 |  | 0.92 (0.86-0.98) | 0.0093 |
| 483 | Acute bronchitis and bronchiolitis | respiratory | 6,256/222,776 | 0.87 (0.79, 0.95) | 0.0019 |  | 0.87 (0.80, 0.95) | 0.003 |  | 0.87 (0.78-0.96) | 0.0029 |
| 433 | Cerebrovascular disease | circulatory system | 47,631/193,417 | 0.95 (0.92, 0.98) | 0.0022 |  | 0.95 (0.92, 0.98) | 0.002 |  | 0.95 (0.92-0.98) | 0.0027 |
| 594.1 | Calculus of kidney | genitourinary | 3,285/237,706 | 0.80 (0.69, 0.93) | 0.0025 |  | 0.81 (0.70, 0.93) | 0.0038 |  | 0.81 (0.66-0.95) | 0.0032 |
| 714 | Rheumatoid arthritis and other inflammatory polyarthropathies | musculoskeletal | 2,166/242,429 | 0.79 (0.68, 0.92) | 0.0026 |  | 0.79 (0.68, 0.92) | 0.0026 |  | 0.79 (0.63-0.94) | 0.0020 |
| 714.1 | Rheumatoid arthritis | musculoskeletal | 2,160/242,429 | 0.79 (0.68, 0.92) | 0.0028 |  | 0.79 (0.68, 0.92) | 0.0028 |  | 0.79 (0.64-0.94) | 0.0021 |
| 574.3 | Cholecystitis without cholelithiasis | digestive | 7,248/210,050 | 0.88 (0.81, 0.96) | 0.0033 |  | 0.88 (0.81, 0.96) | 0.0049 |  | 0.88 (0.79-0.97) | 0.0036 |
| 590 | Pyelonephritis | genitourinary | 6,527/230,026 | 0.88 (0.81, 0.96) | 0.0035 |  | 0.88 (0.81, 0.96) | 0.0042 |  | 0.87 (0.79-0.96) | 0.0018 |
| 805 | Fracture of vertebral column without mention of spinal cord injury | injuries & poisonings | 1,327/236,931 | 0.78 (0.66, 0.92) | 0.0035 |  | 0.78 (0.66, 0.92) | 0.004 |  | 0.78 (0.61-0.95) | 0.0047 |
| 339 | Other headache syndromes | neurological | 5,254/239,140 | 0.86 (0.77, 0.95) | 0.0039 |  | 0.86 (0.77, 0.95) | 0.0039 |  | 0.86 (0.75-0.96) | 0.0036 |
| 760 | Back pain | symptoms | 8,048/236,660 | 0.88 (0.80, 0.96) | 0.0041 |  | 0.88 (0.80, 0.96) | 0.0057 |  | 0.88 (0.78-0.97) | 0.0042 |
| 496.3 | Bronchiectasis | respiratory | 919/211,409 | 0.73 (0.59, 0.91) | 0.0046 |  | 0.72 (0.58, 0.89) | 0.0023 |  | 0.70 (0.48-0.92) | 0.0017 |
| 535.8 | Other specified gastritis | digestive | 9,529/223,512 | 0.91 (0.84, 0.97) | 0.0053 |  | 0.90 (0.84, 0.96) | 0.002 |  | 0.89 (0.82-0.96) | 0.0008 |
| 372 | Disorders of conjunctiva | sense organs | 1,834/239,750 | 0.79 (0.66, 0.93) | 0.0056 |  | 0.79 (0.67, 0.94) | 0.0064 |  | 0.79 (0.62-0.96) | 0.0067 |
| 579.2 | Splenomegaly | digestive | 32/242,763 | 3.52 (1.44, 8.60) | 0.0057 |  | 3.50 (1.43, 8.56) | 0.0059 |  | 3.32 (2.43-4.21) | 0.0082 |
| 411.8 | Other chronic ischemic heart disease, unspecified | circulatory system | 15,059/209,210 | 0.93 (0.89, 0.98) | 0.0059 |  | 0.93 (0.88, 0.97) | 0.0019 |  | 0.95 (0.88-1.01) | 0.1078 |
| 740.9 | Osteoarthrosis NOS | musculoskeletal | 11,146/232,574 | 0.91 (0.85, 0.97) | 0.0062 |  | 0.93 (0.87, 0.99) | 0.0217 |  | 0.93 (0.86-0.99) | 0.0225 |
| 480.5 | Bronchopneumonia and lung abscess | respiratory | 2,078/222,776 | 0.81 (0.70, 0.95) | 0.0069 |  | 0.81 (0.70, 0.95) | 0.0073 |  | 0.81 (0.67-0.96) | 0.0075 |
| 729 | Other disorders of soft tissues | musculoskeletal | 1,068/242,934 | 0.74 (0.59, 0.92) | 0.0071 |  | 0.75 (0.60, 0.93) | 0.0096 |  | 0.74 (0.52-0.96) | 0.0078 |
| 729.1 | Rheumatism, unspecified and fibrositis | musculoskeletal | 1,068/242,934 | 0.74 (0.59, 0.92) | 0.0071 |  | 0.75 (0.60, 0.93) | 0.0096 |  | 0.74 (0.52-0.96) | 0.0078 |
| 454.1 | Varicose veins of lower extremity | circulatory system | 886/238,846 | 1.37 (1.08, 1.72) | 0.0082 |  | 1.40 (1.11, 1.77) | 0.0045 |  | 1.40 (1.16-1.63) | 0.0050 |
| 465.4 | Acute laryngitis and tracheitis | respiratory | 3,083/217,033 | 0.82 (0.71, 0.95) | 0.0094 |  | 0.81 (0.70, 0.94) | 0.0066 |  | 0.81 (0.66-0.96) | 0.0063 |
| 433.2 | Occlusion of cerebral arteries | circulatory system | 28,330/193,417 | 0.95 (0.92, 0.99) | 0.0103 |  | 0.95 (0.91, 0.98) | 0.005 |  | 0.95 (0.91-0.99) | 0.0068 |
| 454 | Varicose veins | circulatory system | 899/238,846 | 1.35 (1.07, 1.70) | 0.0105 |  | 1.38 (1.10, 1.74) | 0.0059 |  | 1.37 (1.14-1.61) | 0.0071 |
| 381 | Otitis media and Eustachian tube disorders | sense organs | 733/243,953 | 0.66 (0.47, 0.91) | 0.0109 |  | 0.66 (0.48, 0.91) | 0.0118 |  | 0.66 (0.33-0.98) | 0.0107 |
| 381.11 | Suppurative and unspecified otitis media | sense organs | 653/243,953 | 0.64 (0.45, 0.90) | 0.011 |  | 0.64 (0.45, 0.90) | 0.0116 |  | 0.63 (0.28-0.98) | 0.0097 |
| 381.1 | Otitis media | sense organs | 728/243,953 | 0.66 (0.48, 0.91) | 0.0119 |  | 0.66 (0.48, 0.92) | 0.0129 |  | 0.66 (0.33-0.98) | 0.0116 |
| 574 | Cholelithiasis and cholecystitis | digestive | 33,953/210,050 | 0.95 (0.92, 0.99) | 0.013 |  | 0.95 (0.92, 0.99) | 0.0186 |  | 0.96 (0.92-0.99) | 0.0211 |
| 480.11 | Pneumococcal pneumonia | respiratory | 281/222,776 | 1.48 (1.09, 2.02) | 0.0131 |  | 1.47 (1.08, 2.01) | 0.0145 |  | 1.50 (1.19-1.81) | 0.0106 |
| 800.4 | Fracture of patella | injuries & poisonings | 632/236,931 | 0.70 (0.52, 0.93) | 0.0141 |  | 0.69 (0.52, 0.92) | 0.0123 |  | 0.69 (0.40-0.98) | 0.0118 |
| 433.21 | Cerebral artery occlusion, with cerebral infarction | circulatory system | 25,547/193,417 | 0.95 (0.91, 0.99) | 0.0144 |  | 0.95 (0.91, 0.98) | 0.0068 |  | 0.95 (0.91-0.99) | 0.0099 |
| 242 | Thyrotoxicosis with or without goiter | endocrine/metabolic | 671/241,973 | 0.65 (0.46, 0.92) | 0.0147 |  | 0.64 (0.45, 0.91) | 0.0118 |  | 0.64 (0.29-0.99) | 0.0117 |
| 455 | Hemorrhoids | circulatory system | 4,964/238,846 | 0.87 (0.77, 0.97) | 0.0148 |  | 0.87 (0.78, 0.98) | 0.0175 |  | 0.87 (0.76-0.99) | 0.0176 |
| 531 | Peptic ulcer (excl. esophageal) | digestive | 10,970/233,738 | 0.92 (0.87, 0.99) | 0.0153 |  | 0.90 (0.85, 0.96) | 0.002 |  | 0.90 (0.83-0.96) | 0.0007 |
| 377 | Disorders of optic nerve and visual pathways | sense organs | 151/243,460 | 0.44 (0.22, 0.86) | 0.0162 |  | 0.44 (0.23, 0.87) | 0.0189 |  | 0.45 (-0.23-1.13) | 0.0205 |
| 193 | Thyroid cancer | neoplasms | 542/243,929 | 0.65 (0.45, 0.93) | 0.0192 |  | 0.64 (0.44, 0.93) | 0.0177 |  | 0.64 (0.27-1.00) | 0.0158 |
| 172.2 | Other non-epithelial cancer of skin | neoplasms | 88/244,574 | 1.81 (1.09, 2.99) | 0.0211 |  | 1.81 (1.10, 3.01) | 0.0207 |  | 1.83 (1.32-2.33) | 0.0193 |
| 766 | Neuralgia, neuritis, and radiculitis NOS | symptoms | 2,114/241,933 | 0.82 (0.69, 0.97) | 0.023 |  | 0.81 (0.68, 0.97) | 0.0195 |  | 0.81 (0.63-0.98) | 0.0160 |
| 276 | Disorders of fluid, electrolyte, and acid-base balance | endocrine/metabolic | 1,292/243,403 | 0.82 (0.69, 0.97) | 0.0232 |  | 0.81 (0.68, 0.96) | 0.0175 |  | 0.80 (0.63-0.97) | 0.0122 |
| 276.1 | Electrolyte imbalance | endocrine/metabolic | 1,292/243,403 | 0.82 (0.69, 0.97) | 0.0232 |  | 0.81 (0.68, 0.96) | 0.0175 |  | 0.80 (0.63-0.97) | 0.0122 |
| 362.31 | Separation of retinal layers | sense organs | 376/242,577 | 1.42 (1.04, 1.94) | 0.0268 |  | 1.43 (1.04, 1.95) | 0.0259 |  | 1.44 (1.13-1.75) | 0.0218 |
| 479 | Other upper respiratory disease | respiratory | 362/239,202 | 0.67 (0.46, 0.96) | 0.0276 |  | 0.68 (0.48, 0.98) | 0.0393 |  | 0.69 (0.32-1.05) | 0.0421 |
| 375 | Disorders of lacrimal system | sense organs | 415/243,460 | 0.67 (0.47, 0.96) | 0.0281 |  | 0.67 (0.47, 0.96) | 0.0302 |  | 0.67 (0.31-1.03) | 0.0310 |
| 362.3 | Other nondiabetic retinopathy | sense organs | 377/242,577 | 1.42 (1.04, 1.94) | 0.0282 |  | 1.42 (1.04, 1.94) | 0.0272 |  | 1.44 (1.12-1.75) | 0.0229 |
| 558 | Noninfectious gastroenteritis | digestive | 364/242,736 | 0.60 (0.38, 0.95) | 0.03 |  | 0.59 (0.37, 0.94) | 0.0251 |  | 0.59 (0.12-1.05) | 0.0236 |
| 280.1 | Intracerebral hemorrhage | hematopoietic | 5,061/193,,417 | 1.09 (1.01, 1.18) | 0.0318 |  | 1.07 (0.99, 1.16) | 0.037 |  | 1.06 (0.99-1.14) | 0.1165 |
| 430.2 | Iron deficiency anemias, unspecified or not due to blood loss | circulatory system | 279/242213 | 1.49 (1.04, 2.14) | 0.0318 |  | 1.47 (1.02, 2.12) | 0.0891 |  | 1.48 (1.12-1.85) | 0.0352 |
| 280 | Iron deficiency anemias | hematopoietic | 355/242,213 | 1.42 (1.03, 1.97) | 0.0337 |  | 1.41 (1.02, 1.96) | 0.039 |  | 1.41 (1.08-1.74) | 0.0396 |
| 204.11 | Lymphoid leukemia, acute | neoplasms | 43/243,740 | 2.42 (1.06, 5.49) | 0.0349 |  | 2.49 (1.09, 5.66) | 0.0299 |  | 2.44 (1.62-3.26) | 0.0334 |
| 362 | Other retinal disorders | sense organs | 832/242,577 | 1.25 (1.02, 1.54) | 0.0354 |  | 1.25 (1.01, 1.54) | 0.0362 |  | 1.25 (1.04-1.46) | 0.0346 |
| 425.12 | Other hypertrophic cardiomyopathy | circulatory system | 43/243,466 | 0.12 (0.02, 0.88) | 0.0376 |  | 0.12 (0.02, 0.89) | 0.0386 |  | 0.13 (-1.89-2.15) | 0.0445 |
| 272 | Disorders of lipoid metabolism | endocrine/metabolic | 3,438/241,270 | 0.88 (0.78, 0.99) | 0.038 |  | 0.89 (0.78, 1.00) | 0.054 |  | 0.89 (0.77-1.01) | 0.0625 |
| 272.1 | Hyperlipidemia | endocrine/metabolic | 3,438/241,270 | 0.88 (0.78, 0.99) | 0.038 |  | 0.89 (0.78, 1.00) | 0.054 |  | 0.89 (0.77-1.01) | 0.0625 |
| 501 | Pneumonitis due to inhalation of food or vomitus | respiratory | 38/243,046 | 2.22 (1.02, 4.87) | 0.0457 |  | 2.12 (0.96, 4.65) | 0.0625 |  | 2.16 (1.37-2.95) | 0.0568 |
| 764 | Sciatica | symptoms | 736/241,933 | 0.76 (0.57, 1.00) | 0.0479 |  | 0.76 (0.58, 1.01) | 0.0576 |  | 0.75 (0.47-1.03) | 0.0444 |
| 430 | Intracranial hemorrhage | circulatory system | 5,602/193,417 | 1.08 (1.00, 1.16) | 0.0483 |  | 1.06 (0.98, 1.14) | 0.1299 |  | 1.05 (0.98-1.13) | 0.1725 |
| 536 | Disorders of function of stomach | digestive | 1,814/223,512 | 0.85 (0.72, 1.00) | 0.0513 |  | 0.85 (0.72, 1.01) | 0.0589 |  | 0.85 (0.69-1.02) | 0.0543 |
| 536.8 | Dyspepsia and other specified disorders of function of stomach | digestive | 1,814/223,512 | 0.85 (0.72, 1.00) | 0.0513 |  | 0.85 (0.72, 1.01) | 0.0589 |  | 0.85 (0.69-1.02) | 0.0543 |
| 272.11 | Hypercholesterolemia | endocrine/metabolic | 256/241,270 | 0.61 (0.36, 1.01) | 0.0524 |  | 0.61 (0.37, 1.02) | 0.0607 |  | 0.62 (0.11-1.12) | 0.0617 |
| 428.1 | Congestive heart failure (CHF) NOS | circulatory system | 2,096/242,460 | 0.89 (0.80, 1.00) | 0.0532 |  | 0.88 (0.79, 0.99) | 0.0374 |  | 0.89 (0.77-1.00) | 0.0461 |
| 427.8 | Sinoatrial node dysfunction (Bradycardia) | circulatory system | 114/239,292 | 0.56 (0.32, 1.01) | 0.0533 |  | 0.56 (0.31, 1.00) | 0.049 |  | 0.56 (-0.02-1.14) | 0.0485 |
| 577 | Diseases of pancreas | digestive | 995/243,713 | 0.81 (0.65, 1.00) | 0.0547 |  | 0.81 (0.65, 1.01) | 0.0586 |  | 0.82 (0.60-1.03) | 0.0653 |
| 149.4 | Cancer of larynx | neoplasms | 41/244,316 | 0.30 (0.08, 1.03) | 0.0564 |  | 0.27 (0.08, 0.96) | 0.0424 |  | 0.27 (-0.99-1.54) | 0.0448 |
| 550 | Abdominal hernia | digestive | 35/244,673 | 2.26 (0.98, 5.25) | 0.0565 |  | 2.36 (1.01, 5.50) | 0.0473 |  | 2.34 (1.50-3.19) | 0.0484 |
| 157 | Pancreatic cancer | neoplasms | 471/237,409 | 0.78 (0.60, 1.01) | 0.0587 |  | 0.78 (0.60, 1.02) | 0.0679 |  | 0.79 (0.53-1.05) | 0.0755 |
| 472 | Chronic pharyngitis and nasopharyngitis | respiratory | 1,311/239,202 | 0.79 (0.62, 1.01) | 0.0606 |  | 0.79 (0.62, 1.01) | 0.0611 |  | 0.77 (0.52-1.02) | 0.0433 |
| 285 | Other anemias | hematopoietic | 1,977/242,213 | 0.87 (0.75, 1.01) | 0.0652 |  | 0.86 (0.74, 1.00) | 0.0432 |  | 0.86 (0.71-1.01) | 0.0480 |
| 454.11 | Varicose veins of lower extremity, symptomtic | circulatory system | 52/238,846 | 2.28 (0.94, 5.50) | 0.0679 |  | 2.42 (1.00, 5.84) | 0.0493 |  | 2.36 (1.48-3.25) | 0.0567 |
| 586 | Other disorders of the kidney and ureters | genitourinary | 702/235,289 | 0.78 (0.60, 1.02) | 0.0691 |  | 0.78 (0.60, 1.02) | 0.0664 |  | 0.78 (0.51-1.04) | 0.0570 |
| 577.1 | Acute pancreatitis | digestive | 923/243,713 | 0.81 (0.65, 1.02) | 0.0696 |  | 0.81 (0.65, 1.02) | 0.0743 |  | 0.82 (0.59-1.04) | 0.0814 |
| 530.14 | Reflux esophagitis | digestive | 356/243,979 | 0.72 (0.50, 1.03) | 0.0708 |  | 0.72 (0.50, 1.02) | 0.067 |  | 0.71 (0.35-1.07) | 0.0612 |
| 211 | Benign neoplasm of other parts of digestive system | neoplasms | 1,134/237,769 | 0.83 (0.68, 1.02) | 0.0722 |  | 0.84 (0.69, 1.02) | 0.0756 |  | 0.83 (0.63-1.03) | 0.0633 |
| 574.1 | Cholelithiasis | digestive | 29,039/210,050 | 0.96 (0.92, 1.00) | 0.0726 |  | 0.96 (0.93, 1.01) | 0.0853 |  | 0.97 (0.92-1.01) | 0.1014 |
| 198 | Secondary malignant neoplasm | neoplasms | 406/243,065 | 1.29 (0.98, 1.71) | 0.0728 |  | 1.27 (0.96, 1.68) | 0.0981 |  | 1.27 (0.98-1.55) | 0.1020 |
| 427 | Cardiac dysrhythmias | circulatory system | 5,332/239,292 | 0.92 (0.85, 1.01) | 0.073 |  | 0.92 (0.84, 1.00) | 0.0586 |  | 0.93 (0.84-1.01) | 0.0841 |
| 580.12 | Non-proliferative glomerulonephritis | genitourinary | 67/235,289 | 0.34 (0.10, 1.13) | 0.0788 |  | 0.33 (0.10, 1.11) | 0.0745 |  | 0.32 (-0.89-1.52) | 0.0632 |
| 153.3 | Malignant neoplasm of rectum, rectosigmoid junction, and anus | neoplasms | 1,028/238,002 | 1.17 (0.98, 1.39) | 0.08 |  | 1.17 (0.98, 1.38) | 0.0813 |  | 1.18 (1.01-1.35) | 0.0574 |
| 300.1 | Anxiety disorder | mental disorders | 588/240,702 | 0.77 (0.57, 1.03) | 0.0807 |  | 0.76 (0.56, 1.02) | 0.0673 |  | 0.74 (0.44-1.04) | 0.0505 |
| 290.11 | Alzheimer's disease | mental disorders | 49/244,423 | 1.78 (0.93, 3.40) | 0.0809 |  | 1.74 (0.91, 3.33) | 0.0941 |  | 1.75 (1.10-2.41) | 0.0911 |
| 613 | Other nonmalignant breast conditions | genitourinary | 99/244,609 | 1.87 (0.93, 3.77) | 0.081 |  | 1.85 (0.92, 3.73) | 0.0862 |  | 1.92 (1.21-2.62) | 0.0708 |
| 740.12 | Osteoarthrosis, localized, secondary | musculoskeletal | 91/232,574 | 1.89 (0.92, 3.86) | 0.0819 |  | 1.92 (0.93, 3.94) | 0.0764 |  | 1.90 (1.18-2.62) | 0.0820 |
| 579 | Other symptoms involving abdomen and pelvis | digestive | 159/242,763 | 1.45 (0.95, 2.22) | 0.084 |  | 1.45 (0.95, 2.22) | 0.0843 |  | 1.44 (1.01-1.86) | 0.0941 |
| 284 | Aplastic anemia | hematopoietic | 85/242,213 | 1.68 (0.93, 3.02) | 0.0844 |  | 1.69 (0.94, 3.05) | 0.081 |  | 1.64 (1.05-2.23) | 0.1019 |
| 377.1 | Optic atrophy | sense organs | 63/243,460 | 0.46 (0.18, 1.12) | 0.0868 |  | 0.46 (0.19, 1.13) | 0.0919 |  | 0.46 (-0.44-1.36) | 0.0912 |
| 134 | Helminthiases | infectious diseases | 38/244,648 | 0.27 (0.06, 1.21) | 0.0869 |  | 0.29 (0.07, 1.28) | 0.1014 |  | 0.29 (-1.21-1.78) | 0.1000 |
| 191.11 | Retinal detachments and defects | neoplasms | 346/242,577 | 1.32 (0.96, 1.83) | 0.0901 |  | 1.32 (0.96, 1.83) | 0.0733 |  | 1.34 (1.02-1.67) | 0.0753 |
| 361 | Cancer of brain | sense organs | 298/243,789 | 0.71 (0.48, 1.06) | 0.0901 |  | 0.70 (0.47, 1.03) | 0.0893 |  | 0.68 (0.28-1.08) | 0.0593 |
| 537.1 | Lesions of stomach and duodenum | digestive | 67/223,512 | 0.40 (0.14, 1.16) | 0.0902 |  | 0.39 (0.14, 1.15) | 0.0883 |  | 0.36 (-0.71-1.43) | 0.0615 |
| 519 | Other diseases of respiratory system, not elsewhere classified | respiratory | 1,156/243,552 | 0.82 (0.65, 1.03) | 0.0913 |  | 0.83 (0.66, 1.04) | 0.1009 |  | 0.82 (0.59-1.05) | 0.0878 |
| 519.8 | Other diseases of respiratory system, NEC | respiratory | 1,156/243,552 | 0.82 (0.65, 1.03) | 0.0913 |  | 0.83 (0.66, 1.04) | 0.1009 |  | 0.82 (0.59-1.05) | 0.0878 |
| 740.11 | Osteoarthrosis, localized, primary | musculoskeletal | 899/232,574 | 0.83 (0.68, 1.03) | 0.0928 |  | 0.86 (0.69, 1.06) | 0.1493 |  | 0.85 (0.64-1.06) | 0.1313 |
| 480.1 | Bacterial pneumonia | respiratory | 505/222,776 | 1.22 (0.97, 1.55) | 0.093 |  | 1.22 (0.96, 1.54) | 0.1028 |  | 1.24 (1.00-1.48) | 0.0769 |
| 427.5 | Arrhythmia (cardiac) NOS | circulatory system | 3,260/239,292 | 0.91 (0.82, 1.02) | 0.0934 |  | 0.91 (0.82, 1.01) | 0.0804 |  | 0.92 (0.81-1.02) | 0.1040 |
| 726.1 | Enthesopathy | musculoskeletal | 342/242,934 | 0.67 (0.42, 1.07) | 0.0958 |  | 0.68 (0.42, 1.08) | 0.1 |  | 0.70 (0.23-1.17) | 0.1364 |
| 565 | Anal and rectal conditions | digestive | 1,170/242,654 | 0.82 (0.64, 1.04) | 0.0968 |  | 0.81 (0.64, 1.03) | 0.0893 |  | 0.80 (0.56-1.04) | 0.0729 |
| 331.1 | Hydrocephalus | neurological | 74/242,836 | 0.51 (0.23, 1.13) | 0.0971 |  | 0.51 (0.23, 1.12) | 0.0921 |  | 0.51 (-0.28-1.30) | 0.0971 |
| 464 | Acute sinusitis | respiratory | 49/217,033 | 0.29 (0.07, 1.27) | 0.1 |  | 0.29 (0.07, 1.28) | 0.1033 |  | 0.30 (-1.17-1.77) | 0.1081 |
| 428 | Congestive heart failure; nonhypertensive | circulatory system | 2,245/242,460 | 0.91 (0.82, 1.02) | 0.1025 |  | 0.90 (0.81, 1.01) | 0.069 |  | 0.91 (0.79-1.02) | 0.0826 |
| 174 | Breast cancer | neoplasms | 1,127/239,760 | 0.85 (0.69, 1.03) | 0.1038 |  | 0.84 (0.69, 1.03) | 0.097 |  | 0.90 (0.66-1.14) | 0.3826 |
| 817 | Concussion | injuries & poisonings | 217/242,268 | 0.62 (0.35, 1.11) | 0.1057 |  | 0.63 (0.35, 1.12) | 0.1125 |  | 0.63 (0.05-1.21) | 0.1170 |
| 523.32 | Chronic periodontitis | digestive | 4,656/239,130 | 0.90 (0.79, 1.02) | 0.1075 |  | 0.90 (0.79, 1.02) | 0.1016 |  | 0.90 (0.77-1.03) | 0.0976 |
| 411.9 | Other acute and subacute forms of ischemic heart disease | circulatory system | 907/209,210 | 0.84 (0.68, 1.04) | 0.1076 |  | 0.85 (0.69, 1.05) | 0.1345 |  | 0.85 (0.64-1.06) | 0.1229 |
| 594.8 | Renal colic | genitourinary | 868/237,706 | 0.82 (0.64, 1.05) | 0.1084 |  | 0.82 (0.64, 1.05) | 0.11 |  | 0.83 (0.59-1.08) | 0.1447 |
| 425 | Cardiomyopathy | circulatory system | 411/243,466 | 0.77 (0.55, 1.06) | 0.1098 |  | 0.77 (0.55, 1.06) | 0.1134 |  | 0.78 (0.46-1.11) | 0.1431 |
| 370 | Keratitis | sense organs | 713/239,750 | 0.80 (0.60, 1.06) | 0.1176 |  | 0.80 (0.60, 1.06) | 0.1198 |  | 0.78 (0.50-1.07) | 0.0955 |
| 578.8 | Hemorrhage of rectum and anus | digestive | 164/242,763 | 0.65 (0.37, 1.12) | 0.1203 |  | 0.65 (0.37, 1.12) | 0.121 |  | 0.64 (0.09-1.20) | 0.1192 |
| 599.3 | Dysuria | genitourinary | 72/244,224 | 1.75 (0.86, 3.54) | 0.1227 |  | 1.75 (0.86, 3.55) | 0.122 |  | 1.63 (0.92-2.34) | 0.1776 |
| 425.1 | Primary/intrinsic cardiomyopathies | circulatory system | 409/243,466 | 0.77 (0.56, 1.07) | 0.1234 |  | 0.78 (0.56, 1.08) | 0.1275 |  | 0.79 (0.46-1.12) | 0.1602 |
| 159.2 | Malignant neoplasm of small intestine, including duodenum | neoplasms | 92/237,409 | 0.58 (0.29, 1.17) | 0.1256 |  | 0.60 (0.30, 1.20) | 0.1476 |  | 0.62 (-0.08-1.32) | 0.1818 |
| 747 | Cardiac and circulatory congenital anomalies | congenital anomalies | 160/244,548 | 0.60 (0.32, 1.15) | 0.1257 |  | 0.60 (0.32, 1.15) | 0.1242 |  | 0.59 (-0.06-1.24) | 0.1097 |
| 726 | Peripheral enthesopathies and allied syndromes | musculoskeletal | 343/242,934 | 0.70 (0.44, 1.11) | 0.1263 |  | 0.70 (0.44, 1.11) | 0.132 |  | 0.73 (0.27-1.19) | 0.1773 |
| 977 | Personal history of allergy to medicinal agents | injuries & poisonings | 47/244,661 | 0.32 (0.07, 1.38) | 0.1265 |  | 0.31 (0.07, 1.34) | 0.1155 |  | 0.32 (-1.16-1.81) | 0.1355 |
| 979 | Adverse drug events and drug allergies | injuries & poisonings | 47/244,661 | 0.32 (0.07, 1.38) | 0.1265 |  | 0.31 (0.07, 1.34) | 0.1155 |  | 0.32 (-1.16-1.81) | 0.1355 |
| 433.5 | Cerebral aneurysm | circulatory system | 151/193,417 | 0.62 (0.34, 1.15) | 0.1292 |  | 0.62 (0.34, 1.15) | 0.132 |  | 0.63 (0.01-1.25) | 0.1410 |
| 720 | Spinal stenosis | musculoskeletal | 1,162/243,423 | 0.87 (0.73, 1.04) | 0.135 |  | 0.88 (0.74, 1.06) | 0.1726 |  | 0.88 (0.70-1.06) | 0.1683 |
| 10 | Tuberculosis | infectious diseases | 3,984/240,464 | 1.07 (0.98, 1.18) | 0.1384 |  | 1.05 (0.96, 1.16) | 0.2744 |  | 1.07 (0.96-1.17) | 0.2243 |
| 788 | Syncope and collapse | symptoms | 263/244,445 | 0.74 (0.50, 1.10) | 0.1416 |  | 0.73 (0.49, 1.09) | 0.1217 |  | 0.72 (0.32-1.12) | 0.1057 |
| 530.11 | GERD | digestive | 55/243,979 | 0.51 (0.21, 1.25) | 0.1419 |  | 0.52 (0.21, 1.27) | 0.1535 |  | 0.49 (-0.40-1.39) | 0.1234 |
| 740.1 | Osteoarthritis; localized | musculoskeletal | 1,263/232,574 | 0.88 (0.73, 1.05) | 0.1435 |  | 0.90 (0.75, 1.08) | 0.2445 |  | 0.89 (0.72-1.07) | 0.2190 |
| 275 | Disorders of mineral metabolism | endocrine/metabolic | 208/244,500 | 0.66 (0.37, 1.15) | 0.1439 |  | 0.67 (0.38, 1.18) | 0.1638 |  | 0.68 (0.11-1.24) | 0.1768 |
| 357 | Inflammatory and toxic neuropathy | neurological | 330/244,308 | 1.29 (0.92, 1.80) | 0.1442 |  | 1.28 (0.91, 1.79) | 0.1538 |  | 1.28 (0.94-1.62) | 0.1570 |
| 275.5 | Disorders of calcium/phosphorus metabolism | endocrine/metabolic | 206/244,500 | 0.66 (0.38, 1.16) | 0.1468 |  | 0.67 (0.38, 1.18) | 0.1689 |  | 0.68 (0.12-1.24) | 0.1808 |
| 53 | Herpes zoster | infectious diseases | 1,807/241,496 | 0.90 (0.78, 1.04) | 0.1528 |  | 0.90 (0.78, 1.04) | 0.1599 |  | 0.91 (0.76-1.05) | 0.1984 |
| 150 | Cancer of esophagus | neoplasms | 902/237,409 | 0.87 (0.72, 1.05) | 0.1539 |  | 0.85 (0.71, 1.03) | 0.1037 |  | 0.88 (0.69-1.08) | 0.2055 |
| 300.11 | Generalized anxiety disorder | mental disorders | 491/240,702 | 0.79 (0.57, 1.09) | 0.1545 |  | 0.78 (0.56, 1.08) | 0.1281 |  | 0.76 (0.43-1.08) | 0.0919 |
| 512.8 | Cough | respiratory | 1,809/242,891 | 0.88 (0.75, 1.05) | 0.1553 |  | 0.88 (0.74, 1.04) | 0.1412 |  | 0.89 (0.72-1.06) | 0.1762 |
| 427.11 | Paroxysmal supraventricular tachycardia | circulatory system | 253/239,292 | 0.69 (0.41, 1.16) | 0.157 |  | 0.69 (0.41, 1.16) | 0.1618 |  | 0.69 (0.17-1.22) | 0.1699 |
| 531.1 | Hemorrhage from gastrointestinal ulcer | digestive | 360/233,738 | 1.23 (0.92, 1.63) | 0.1583 |  | 1.20 (0.90, 1.60) | 0.2095 |  | 1.19 (0.91-1.48) | 0.2306 |
| 994 | Sepsis and SIRS | injuries & poisonings | 162/244,546 | 1.34 (0.89, 2.04) | 0.1629 |  | 1.33 (0.88, 2.03) | 0.1746 |  | 1.32 (0.91-1.74) | 0.1879 |
| 994.2 | Sepsis | injuries & poisonings | 162/244,546 | 1.34 (0.89, 2.04) | 0.1629 |  | 1.33 (0.88, 2.03) | 0.1746 |  | 1.32 (0.91-1.74) | 0.1879 |
| 287 | Purpura and other hemorrhagic conditions | hematopoietic | 491/244,217 | 0.80 (0.58, 1.10) | 0.1632 |  | 0.78 (0.57, 1.07) | 0.1256 |  | 0.78 (0.46-1.10) | 0.1245 |
| 191 | Manlignant and unknown neoplasms of brain and nervous system | neoplasms | 370/243,789 | 0.79 (0.56, 1.10) | 0.1634 |  | 0.77 (0.55, 1.08) | 0.1299 |  | 0.75 (0.41-1.09) | 0.1042 |
| 475 | Chronic sinusitis | respiratory | 882/239,202 | 0.83 (0.64, 1.08) | 0.1727 |  | 0.83 (0.63, 1.08) | 0.1582 |  | 0.82 (0.55-1.09) | 0.1421 |
| 512 | Other symptoms of respiratory system | respiratory | 1,817/242,891 | 0.89 (0.75, 1.05) | 0.1785 |  | 0.89 (0.75, 1.05) | 0.1638 |  | 0.90 (0.73-1.07) | 0.2042 |
| 523 | Gingival and periodontal diseases | digestive | 5,132/239,130 | 0.92 (0.82, 1.04) | 0.1838 |  | 0.92 (0.82, 1.04) | 0.1772 |  | 0.92 (0.80-1.04) | 0.1854 |
| 747.1 | Cardiac congenital anomalies | congenital anomalies | 152/244,548 | 0.64 (0.34, 1.23) | 0.1839 |  | 0.64 (0.33, 1.23) | 0.1789 |  | 0.63 (-0.02-1.28) | 0.1572 |
| 38 | Septicemia | infectious diseases | 167/240,464 | 1.32 (0.88, 1.99) | 0.1848 |  | 1.31 (0.87, 1.98) | 0.1978 |  | 1.30 (0.88-1.71) | 0.2164 |
| 377.3 | Optic neuritis/neuropathy | sense organs | 87/243,460 | 0.53 (0.21, 1.36) | 0.1873 |  | 0.54 (0.21, 1.40) | 0.205 |  | 0.56 (-0.38-1.50) | 0.2292 |
| 225 | Benign neoplasm of brain and other parts of nervous system | neoplasms | 251/243,789 | 1.29 (0.88, 1.90) | 0.1888 |  | 1.30 (0.88, 1.90) | 0.1855 |  | 1.28 (0.89-1.66) | 0.2105 |
| 334 | Degenerative disease of the spinal cord | neurological | 32/242,836 | 0.25 (0.03, 1.96) | 0.1896 |  | 0.25 (0.03, 1.91) | 0.1808 |  | 0.25 (-1.79-2.29) | 0.1792 |
| 572 | Ascites (non malignant) | digestive | 166/241,917 | 0.71 (0.43, 1.18) | 0.1915 |  | 0.69 (0.41, 1.14) | 0.1435 |  | 0.69 (0.19-1.20) | 0.1530 |
| 281.9 | Deficiency anemias | hematopoietic | 131/242,213 | 0.59 (0.27, 1.30) | 0.1927 |  | 0.58 (0.26, 1.27) | 0.1741 |  | 0.57 (-0.23-1.37) | 0.1686 |
| 198.6 | Secondary malignancy of bone | neoplasms | 67/243,065 | 1.54 (0.80, 2.93) | 0.1928 |  | 1.49 (0.78, 2.86) | 0.2245 |  | 1.45 (0.78-2.12) | 0.2754 |
| 571 | Chronic liver disease and cirrhosis | digestive | 1,743/241,917 | 0.90 (0.77, 1.05) | 0.1956 |  | 0.90 (0.77, 1.06) | 0.2079 |  | 0.91 (0.75-1.06) | 0.2175 |
| 574.12 | Cholelithiasis with other cholecystitis | digestive | 4,134/210,050 | 0.93 (0.83, 1.04) | 0.1959 |  | 0.93 (0.83, 1.05) | 0.2399 |  | 0.94 (0.83-1.05) | 0.2766 |
| 172 | Skin cancer | neoplasms | 134/244,574 | 1.34 (0.86, 2.08) | 0.2005 |  | 1.33 (0.85, 2.07) | 0.2074 |  | 1.34 (0.90-1.79) | 0.1926 |
| 241.1 | Nontoxic uninodular goiter | endocrine/metabolic | 338/241,973 | 0.71 (0.42, 1.20) | 0.2039 |  | 0.71 (0.42, 1.20) | 0.2027 |  | 0.71 (0.19-1.24) | 0.2106 |
| 571.8 | Liver abscess and sequelae of chronic liver disease | digestive | 212/241,917 | 0.76 (0.49, 1.17) | 0.2062 |  | 0.76 (0.49, 1.17) | 0.2076 |  | 0.78 (0.34-1.21) | 0.2546 |
| 225.1 | Benign neoplasm of brain, cranial nerves, meninges | neoplasms | 243/243,789 | 1.28 (0.87, 1.89) | 0.2083 |  | 1.28 (0.87, 1.89) | 0.2074 |  | 1.27 (0.88-1.66) | 0.2308 |
| 145.3 | Cancer of major salivary glands | neoplasms | 43/244,316 | 1.83 (0.71, 4.68) | 0.2106 |  | 1.84 (0.72, 4.73) | 0.2029 |  | 1.91 (0.96-2.85) | 0.1798 |
| 199 | Neoplasm of uncertain behavior | neoplasms | 968/243,065 | 0.87 (0.71, 1.08) | 0.211 |  | 0.87 (0.71, 1.08) | 0.1996 |  | 0.87 (0.66-1.08) | 0.2089 |
| 204.2 | Myeloid leukemia | neoplasms | 119/243,740 | 1.41 (0.82, 2.42) | 0.2119 |  | 1.43 (0.83, 2.46) | 0.1916 |  | 1.43 (0.89-1.97) | 0.1964 |
| 287.3 | Thrombocytopenia | hematopoietic | 485/244,217 | 0.82 (0.60, 1.12) | 0.2172 |  | 0.80 (0.58, 1.10) | 0.1682 |  | 0.80 (0.48-1.12) | 0.1673 |
| 444 | Fracture of ribs | circulatory system | 771/236,931 | 0.86 (0.67, 1.10) | 0.2228 |  | 0.87 (0.68, 1.10) | 0.2723 |  | 0.87 (0.63-1.11) | 0.2636 |
| 807 | Arterial embolism and thrombosis | injuries & poisonings | 44/244,115 | 1.57 (0.76, 3.25) | 0.2228 |  | 1.51 (0.73, 3.12) | 0.2452 |  | 1.51 (0.79-2.24) | 0.2637 |
| 594.2 | Calculus of lower urinary tract | genitourinary | 219/237,706 | 0.70 (0.39, 1.24) | 0.2238 |  | 0.71 (0.40, 1.26) | 0.2385 |  | 0.70 (0.12-1.27) | 0.2188 |
| 411.1 | Unstable angina (intermediate coronary syndrome) | circulatory system | 408/209,210 | 0.83 (0.62, 1.12) | 0.2259 |  | 0.82 (0.61, 1.11) | 0.2 |  | 0.83 (0.53-1.13) | 0.2256 |
| 523.3 | Periodontitis (acute or chronic) | digestive | 4,853/239,130 | 0.93 (0.82, 1.05) | 0.2293 |  | 0.93 (0.82, 1.05) | 0.2224 |  | 0.92 (0.80-1.05) | 0.2153 |
| 427.2 | Atrial fibrillation and flutter | circulatory system | 77/239,292 | 1.42 (0.80, 2.51) | 0.231 |  | 1.37 (0.77, 2.43) | 0.2837 |  | 1.40 (0.82-1.97) | 0.2568 |
| 394.4 | Acute rheumatic heart disease | circulatory system | 624/243,375 | 0.86 (0.67, 1.10) | 0.2332 |  | 0.82 (0.64, 1.06) | 0.1284 |  | 0.82 (0.57-1.07) | 0.1113 |
| 790.6 | Other abnormal blood chemistry | symptoms | 200/244,476 | 0.78 (0.52, 1.18) | 0.2338 |  | 0.79 (0.52, 1.19) | 0.2554 |  | 0.78 (0.37-1.20) | 0.2424 |
| 458.9 | Hypotension NOS | circulatory system | 719/243,976 | 0.83 (0.62, 1.13) | 0.2364 |  | 0.83 (0.61, 1.12) | 0.2235 |  | 0.84 (0.54-1.14) | 0.2435 |
| 344 | Other paralytic syndromes | neurological | 80/242,836 | 0.59 (0.25, 1.41) | 0.2373 |  | 0.60 (0.25, 1.44) | 0.2513 |  | NA | NA |
| 153 | Colorectal cancer | neoplasms | 1,925/238,002 | 1.08 (0.95, 1.22) | 0.2375 |  | 1.07 (0.95, 1.22) | 0.269 |  | 1.10 (0.98-1.23) | 0.1309 |
| 191.1 | Cancer of brain and nervous system | neoplasms | 336/243,789 | 0.80 (0.56, 1.15) | 0.2379 |  | 0.79 (0.55, 1.13) | 0.2021 |  | 0.77 (0.41-1.13) | 0.1594 |
| 599.2 | Retention of urine | genitourinary | 117/244,224 | 0.71 (0.39, 1.27) | 0.2438 |  | 0.69 (0.39, 1.24) | 0.2196 |  | 0.70 (0.12-1.29) | 0.2364 |
| 327.3 | Sleep apnea | neurological | 43/243,197 | 0.42 (0.10, 1.84) | 0.2511 |  | 0.43 (0.10, 1.88) | 0.2625 |  | 0.40 (-1.08-1.88) | 0.2276 |
| 365 | Glaucoma | sense organs | 1,272/242,577 | 0.91 (0.77, 1.07) | 0.2513 |  | 0.90 (0.77, 1.06) | 0.216 |  | 0.91 (0.74-1.07) | 0.2292 |
| 586.2 | Cyst of kidney, acquired | genitourinary | 479/235,289 | 0.84 (0.62, 1.13) | 0.2556 |  | 0.84 (0.63, 1.14) | 0.2707 |  | 0.84 (0.54-1.14) | 0.2541 |
| 560 | Intestinal obstruction without mention of hernia | digestive | 1,402/242,736 | 0.91 (0.78, 1.07) | 0.2582 |  | 0.90 (0.77, 1.06) | 0.2031 |  | 0.90 (0.75-1.06) | 0.2158 |
| 172.1 | Melanomas of skin, dx or hx | neoplasms | 49/244,574 | 0.57 (0.21, 1.52) | 0.2584 |  | 0.56 (0.21, 1.49) | 0.2444 |  | 0.57 (-0.42-1.56) | 0.2626 |
| 172.11 | Melanomas of skin | neoplasms | 49/244,574 | 0.57 (0.21, 1.52) | 0.2584 |  | 0.56 (0.21, 1.49) | 0.2444 |  | 0.57 (-0.42-1.56) | 0.2626 |
| 250.1 | Type 1 diabetes | endocrine/metabolic | 381/219,067 | 1.19 (0.88, 1.62) | 0.2594 |  | 1.19 (0.88, 1.62) | 0.2636 |  | 1.14 (0.63-1.66) | 0.6126 |
| 707 | Chronic ulcer of skin | dermatologic | 88/244,620 | 0.73 (0.42, 1.26) | 0.2611 |  | 0.72 (0.41, 1.24) | 0.2355 |  | 0.72 (0.17-1.27) | 0.2410 |
| 707.1 | Decubitus ulcer | dermatologic | 88/244,620 | 0.73 (0.42, 1.26) | 0.2611 |  | 0.72 (0.41, 1.24) | 0.2355 |  | 0.72 (0.17-1.27) | 0.2410 |
| 276.14 | Hypopotassemia | endocrine/metabolic | 997/243,403 | 0.90 (0.74, 1.09) | 0.2676 |  | 0.88 (0.73, 1.07) | 0.2149 |  | 0.87 (0.68-1.07) | 0.1675 |
| 800 | Fracture of lower limb | injuries & poisonings | 2,427/236,931 | 0.94 (0.83, 1.05) | 0.2699 |  | 0.93 (0.83, 1.05) | 0.2565 |  | 0.93 (0.81-1.05) | 0.2339 |
| 174.3 | Neoplasm of uncertain behavior of breast | neoplasms | 113/239,760 | 1.48 (0.74, 2.96) | 0.2701 |  | 1.47 (0.74, 2.95) | 0.2732 |  | 1.44 (0.74-2.13) | 0.3092 |
| 165 | Cancer within the respiratory system | neoplasms | 2,694/241,965 | 0.94 (0.84, 1.05) | 0.2712 |  | 0.91 (0.82, 1.02) | 0.1009 |  | 0.92 (0.81-1.03) | 0.1281 |
| 159.4 | Malignant neoplasm of retroperitoneum and peritoneum | neoplasms | 63/237,409 | 0.64 (0.29, 1.42) | 0.2753 |  | 0.64 (0.29, 1.40) | 0.2636 |  | 0.65 (-0.14-1.44) | 0.2838 |
| 165.1 | Cancer of bronchus; lung | neoplasms | 2,674/241,965 | 0.94 (0.84, 1.05) | 0.2771 |  | 0.91 (0.82, 1.02) | 0.1037 |  | 0.92 (0.81-1.03) | 0.1319 |
| 721 | Spondylosis and allied disorders | musculoskeletal | 103/243,423 | 0.68 (0.34, 1.36) | 0.2783 |  | 0.68 (0.34, 1.37) | 0.2782 |  | 0.69 (-0.01-1.39) | 0.2928 |
| 149 | Cancer of larynx, pharynx, nasal cavities | neoplasms | 253/244,316 | 0.80 (0.53, 1.20) | 0.2797 |  | 0.77 (0.51, 1.17) | 0.2207 |  | 0.79 (0.38-1.20) | 0.2662 |
| 159 | Malignant neoplasm of other and ill-defined sites within the digestive organs and peritoneum | neoplasms | 578/237,409 | 0.88 (0.70, 1.11) | 0.2841 |  | 0.88 (0.70, 1.12) | 0.2943 |  | 0.89 (0.65-1.12) | 0.3101 |
| 458 | Hypotension | circulatory system | 729/243,976 | 0.85 (0.63, 1.14) | 0.2848 |  | 0.85 (0.63, 1.14) | 0.268 |  | 0.85 (0.56-1.15) | 0.2910 |
| 721.2 | Spondylosis with myelopathy | musculoskeletal | 101/243,423 | 0.69 (0.34, 1.38) | 0.2922 |  | 0.69 (0.34, 1.38) | 0.2928 |  | 0.69 (-0.01-1.40) | 0.3082 |
| 840 | Sprains and strains | injuries & poisonings | 311/244,395 | 0.75 (0.43, 1.29) | 0.2939 |  | 0.74 (0.43, 1.28) | 0.2791 |  | 0.75 (0.21-1.30) | 0.3107 |
| 427.7 | Tachycardia NOS | circulatory system | 306/239,292 | 1.21 (0.85, 1.73) | 0.2965 |  | 1.19 (0.83, 1.70) | 0.3422 |  | 1.19 (0.83-1.55) | 0.3480 |
| 290.12 | Dementia with cerebral degenerations | mental disorders | 58/244,423 | 0.72 (0.39, 1.33) | 0.2969 |  | 0.69 (0.37, 1.29) | 0.2444 |  | 0.67 (0.05-1.29) | 0.2137 |
| 568 | Other disorders of peritoneum | digestive | 213/242,654 | 0.73 (0.41, 1.32) | 0.2996 |  | 0.73 (0.40, 1.32) | 0.294 |  | 0.73 (0.14-1.32) | 0.3028 |
| 427.6 | Premature beats | circulatory system | 142/239,292 | 1.30 (0.79, 2.12) | 0.3003 |  | 1.29 (0.79, 2.11) | 0.3101 |  | 1.26 (0.77-1.75) | 0.3599 |
| 379.2 | Disorders of vitreous body | sense organs | 364/243,460 | 0.83 (0.58, 1.18) | 0.3023 |  | 0.83 (0.58, 1.17) | 0.2872 |  | 0.82 (0.47-1.17) | 0.2732 |
| 327 | Sleep disorders | neurological | 1,474/243,197 | 0.90 (0.74, 1.10) | 0.3029 |  | 0.90 (0.74, 1.09) | 0.2709 |  | 0.87 (0.68-1.07) | 0.1809 |
| 580 | Nephritis; nephrosis; renal sclerosis | genitourinary | 1,409/235,289 | 0.91 (0.76, 1.09) | 0.3045 |  | 0.90 (0.75, 1.09) | 0.28 |  | 0.90 (0.72-1.08) | 0.2518 |
| 362.8 | Retinal hemorrhage/ischemia | sense organs | 119/242,577 | 1.32 (0.77, 2.25) | 0.3083 |  | 1.28 (0.75, 2.18) | 0.3599 |  | 1.30 (0.77-1.83) | 0.3368 |
| 244 | Hypothyroidism | endocrine/metabolic | 216/241,973 | 0.78 (0.49, 1.25) | 0.3098 |  | 0.78 (0.49, 1.26) | 0.3112 |  | 0.80 (0.33-1.27) | 0.3508 |
| 79 | Viral infection | infectious diseases | 50/241,496 | 0.57 (0.19, 1.69) | 0.3111 |  | 0.58 (0.20, 1.73) | 0.3302 |  | 0.58 (-0.51-1.67) | 0.3283 |
| 568.1 | Peritoneal adhesions (postoperative) (postinfection) | digestive | 209/242,654 | 0.74 (0.41, 1.33) | 0.3122 |  | 0.74 (0.41, 1.33) | 0.3087 |  | 0.74 (0.15-1.33) | 0.3189 |
| 250.42 | Other abnormal glucose | endocrine/metabolic | 767/219,067 | 0.87 (0.65, 1.15) | 0.3125 |  | 0.87 (0.66, 1.16) | 0.3427 |  | 0.82 (0.45-1.19) | 0.2924 |
| 592.1 | Cystitis | genitourinary | 1,394/230,026 | 0.90 (0.74, 1.10) | 0.313 |  | 0.91 (0.74, 1.11) | 0.3339 |  | 0.92 (0.72-1.12) | 0.3922 |
| 285.2 | Anemia of chronic disease | hematopoietic | 43/242,213 | 0.60 (0.22, 1.62) | 0.3147 |  | 0.56 (0.21, 1.53) | 0.2595 |  | 0.55 (-0.45-1.56) | 0.2484 |
| 366.2 | Senile cataract | sense organs | 3,973/234,082 | 0.96 (0.88, 1.04) | 0.3156 |  | 0.95 (0.88, 1.04) | 0.2626 |  | 0.95 (0.87-1.04) | 0.2533 |
| 595 | Hydronephrosis | genitourinary | 225/237,706 | 1.27 (0.79, 2.03) | 0.3199 |  | 1.29 (0.80, 2.06) | 0.2927 |  | 1.30 (0.83-1.77) | 0.2718 |
| 250.2 | Type 2 diabetes | endocrine/metabolic | 25,152/219,067 | 0.98 (0.94, 1.02) | 0.3276 |  | 0.98 (0.94, 1.02) | 0.2883 |  | 0.97 (0.89-1.06) | 0.5584 |
| 804 | Fracture of hand or wrist | injuries & poisonings | 130/236,931 | 0.66 (0.29, 1.51) | 0.3277 |  | 0.67 (0.29, 1.52) | 0.3356 |  | 0.66 (-0.16-1.48) | 0.3255 |
| 783 | Fever of unknown origin | symptoms | 825/243,883 | 0.90 (0.73, 1.11) | 0.3312 |  | 0.89 (0.73, 1.10) | 0.286 |  | 0.89 (0.68-1.10) | 0.2627 |
| 830 | Dislocation | injuries & poisonings | 450/244,241 | 0.86 (0.64, 1.17) | 0.3318 |  | 0.87 (0.64, 1.18) | 0.3727 |  | 0.87 (0.57-1.17) | 0.3706 |
| 612.2 | Hypertrophy of breast (Gynecomastia) | genitourinary | 273/242,411 | 1.28 (0.77, 2.11) | 0.3396 |  | 1.26 (0.76, 2.09) | 0.3671 |  | 1.22 (0.71-1.73) | 0.4401 |
| 474.2 | Chronic tonsillitis and adenoiditis | respiratory | 661/239,202 | 1.17 (0.85, 1.62) | 0.3424 |  | 1.17 (0.84, 1.62) | 0.3502 |  | 1.17 (0.84-1.50) | 0.3478 |
| 573 | Other disorders of liver | digestive | 1,082/241,917 | 0.90 (0.73, 1.11) | 0.3439 |  | 0.90 (0.73, 1.11) | 0.3035 |  | 0.89 (0.68-1.11) | 0.3003 |
| 401.1 | Essential hypertension | circulatory system | 1,113,74/133,117 | 0.99 (0.96, 1.01) | 0.347 |  | 1.01 (0.98, 1.04) | 0.4548 |  | 1.00 (0.93-1.07) | 0.9028 |
| 495 | Asthma | respiratory | 1,595/211,409 | 1.07 (0.92, 1.25) | 0.3471 |  | 1.05 (0.91, 1.22) | 0.4993 |  | 0.92 (0.72-1.13) | 0.4597 |
| 555 | Inflammatory bowel disease and other gastroenteritis and colitis | digestive | 148/242,736 | 0.76 (0.43, 1.35) | 0.3479 |  | 0.74 (0.42, 1.32) | 0.3084 |  | 0.75 (0.17-1.32) | 0.3229 |
| 555.2 | Ulcerative colitis | digestive | 148/242,736 | 0.76 (0.43, 1.35) | 0.3479 |  | 0.74 (0.42, 1.32) | 0.3084 |  | 0.75 (0.17-1.32) | 0.3229 |
| 560.4 | Other intestinal obstruction | digestive | 1,369/242,736 | 0.93 (0.79, 1.09) | 0.3491 |  | 0.92 (0.78, 1.07) | 0.282 |  | 0.92 (0.76-1.08) | 0.2952 |
| 571.6 | Primary biliary cirrhosis | digestive | 34/241,917 | 0.49 (0.11, 2.17) | 0.3493 |  | 0.49 (0.11, 2.17) | 0.35 |  | 0.49 (-0.99-1.97) | 0.3454 |
| 204.1 | Lymphoid leukemia | neoplasms | 86/243,740 | 1.33 (0.73, 2.40) | 0.3516 |  | 1.35 (0.75, 2.46) | 0.3185 |  | 1.33 (0.73-1.93) | 0.3494 |
| 389 | Hearing loss | sense organs | 401/244,307 | 0.85 (0.59, 1.21) | 0.3529 |  | 0.85 (0.60, 1.22) | 0.3765 |  | 0.85 (0.49-1.21) | 0.3733 |
| 575 | Other biliary tract disease | digestive | 1,413/210,050 | 0.92 (0.77, 1.10) | 0.3533 |  | 0.92 (0.77, 1.10) | 0.3739 |  | 0.92 (0.74-1.11) | 0.3889 |
| 426.2 | Atrioventricular [AV] block | circulatory system | 65/239,292 | 0.71 (0.35, 1.47) | 0.3565 |  | 0.70 (0.34, 1.44) | 0.331 |  | 0.69 (-0.03-1.42) | 0.3168 |
| 531.4 | Peptic ulcer, site unspecified | digestive | 305/233,738 | 0.85 (0.61, 1.20) | 0.359 |  | 0.85 (0.60, 1.19) | 0.342 |  | 0.84 (0.49-1.18) | 0.3098 |
| 564.1 | Irritable Bowel Syndrome | digestive | 36/242,736 | 0.55 (0.15, 1.98) | 0.3593 |  | 0.53 (0.15, 1.91) | 0.3291 |  | 0.52 (-0.77-1.81) | 0.3190 |
| 540.1 | Appendicitis | digestive | 1,522/243,146 | 0.91 (0.75, 1.11) | 0.3631 |  | 0.91 (0.75, 1.11) | 0.3489 |  | 0.91 (0.71-1.11) | 0.3569 |
| 250 | Diabetes mellitus | endocrine/metabolic | 25,252/219,067 | 0.98 (0.94, 1.02) | 0.3636 |  | 0.98 (0.94, 1.02) | 0.3244 |  | 0.98 (0.90-1.07) | 0.6907 |
| 401 | Hypertension | circulatory system | 111,591/133,117 | 0.99 (0.96, 1.02) | 0.3652 |  | 1.01 (0.98, 1.04) | 0.4398 |  | 1.00 (0.93-1.07) | 0.9677 |
| 709.7 | Unspecified diffuse connective tissue disease | dermatologic | 114/244,304 | 0.67 (0.29, 1.60) | 0.3705 |  | 0.65 (0.27, 1.53) | 0.3218 |  | 0.64 (-0.23-1.50) | 0.3053 |
| 803.1 | Fracture of humerus | injuries & poisonings | 1,047/236,931 | 0.92 (0.77, 1.10) | 0.3732 |  | 0.92 (0.77, 1.11) | 0.382 |  | 0.92 (0.74-1.10) | 0.3661 |
| 585.32 | End stage renal disease | genitourinary | 146/235,289 | 0.78 (0.45, 1.35) | 0.3771 |  | 0.76 (0.44, 1.32) | 0.335 |  | 0.75 (0.20-1.31) | 0.3183 |
| 745 | Pain in joint | musculoskeletal | 403/244,305 | 0.86 (0.61, 1.21) | 0.3795 |  | 0.89 (0.63, 1.26) | 0.5141 |  | 0.88 (0.54-1.23) | 0.4821 |
| 496.21 | Obstructive chronic bronchitis | respiratory | 21,505/211,409 | 1.02 (0.98, 1.06) | 0.3832 |  | 1.00 (0.95, 1.04) | 0.8344 |  | 0.94 (0.85-1.03) | 0.1567 |
| 797 | Shock | symptoms | 144/244,564 | 0.80 (0.48, 1.32) | 0.384 |  | 0.81 (0.49, 1.34) | 0.4169 |  | 0.81 (0.30-1.31) | 0.4109 |
| 296.2 | Depression | mental disorders | 476/240,702 | 0.86 (0.61, 1.21) | 0.3844 |  | 0.85 (0.61, 1.19) | 0.34 |  | 0.82 (0.48-1.15) | 0.2369 |
| 296.22 | Major depressive disorder | mental disorders | 476/240,702 | 0.86 (0.61, 1.21) | 0.3844 |  | 0.85 (0.61, 1.19) | 0.34 |  | 0.82 (0.48-1.15) | 0.2369 |
| 430.1 | Subarachnoid hemorrhage | circulatory system | 568/193,417 | 0.90 (0.70, 1.15) | 0.3861 |  | 0.89 (0.69, 1.14) | 0.3535 |  | 0.89 (0.64-1.14) | 0.3483 |
| 612 | Breast conditions, congenital or relating to hormones | genitourinary | 284/242,411 | 1.24 (0.75, 2.05) | 0.3938 |  | 1.23 (0.74, 2.03) | 0.426 |  | 1.19 (0.68-1.70) | 0.4969 |
| 145 | Cancer of mouth | neoplasms | 143/244,316 | 1.23 (0.76, 2.00) | 0.3959 |  | 1.22 (0.75, 1.98) | 0.4216 |  | 1.27 (0.78-1.75) | 0.3384 |
| 801.1 | Fracture of foot | injuries & poisonings | 225/236,931 | 0.81 (0.51, 1.31) | 0.3964 |  | 0.81 (0.50, 1.30) | 0.3861 |  | 0.80 (0.33-1.28) | 0.3685 |
| 522.1 | Pulpitis and necrosis of tooth pulp | digestive | 590/239,130 | 0.86 (0.61, 1.22) | 0.3968 |  | 0.86 (0.61, 1.22) | 0.4073 |  | 0.86 (0.51-1.20) | 0.3885 |
| 288.1 | Decreased white blood cell count | hematopoietic | 671/243,568 | 0.88 (0.65, 1.18) | 0.3971 |  | 0.88 (0.65, 1.18) | 0.3895 |  | 0.88 (0.58-1.18) | 0.4037 |
| 288.11 | Neutropenia | hematopoietic | 671/243,568 | 0.88 (0.65, 1.18) | 0.3971 |  | 0.88 (0.65, 1.18) | 0.3895 |  | 0.88 (0.58-1.18) | 0.4037 |
| 331.9 | Cerebral degeneration, unspecified | neurological | 310/242,836 | 1.13 (0.85, 1.48) | 0.3996 |  | 1.10 (0.84, 1.46) | 0.4854 |  | 1.12 (0.84-1.40) | 0.4311 |
| 349 | Other and unspecified disorders of the nervous system | neurological | 148/242,836 | 1.25 (0.74, 2.12) | 0.4001 |  | 1.24 (0.73, 2.09) | 0.4269 |  | 1.20 (0.68-1.73) | 0.4874 |
| 430.3 | Subdural hemorrhage | circulatory system | 79/193,417 | 0.76 (0.41, 1.43) | 0.4009 |  | 0.74 (0.40, 1.39) | 0.3471 |  | 0.75 (0.13-1.38) | 0.3798 |
| 803 | Fracture of upper limb | injuries & poisonings | 3,210/236,931 | 0.95 (0.85, 1.07) | 0.4022 |  | 0.95 (0.85, 1.07) | 0.4134 |  | 0.95 (0.84-1.07) | 0.4059 |
| 709.2 | Sicca syndrome | dermatologic | 61/244,304 | 1.46 (0.60, 3.56) | 0.4096 |  | 1.41 (0.58, 3.43) | 0.4519 |  | 1.38 (0.48-2.27) | 0.4846 |
| 244.4 | Hypothyroidism NOS | endocrine/metabolic | 206/241,973 | 0.82 (0.51, 1.31) | 0.4108 |  | 0.82 (0.51, 1.32) | 0.4101 |  | 0.84 (0.36-1.31) | 0.4611 |
| 540 | Appendiceal conditions | digestive | 1,562/243,146 | 0.92 (0.76, 1.12) | 0.4133 |  | 0.92 (0.76, 1.12) | 0.3978 |  | 0.92 (0.73-1.12) | 0.4170 |
| 281 | Other deficiency anemia | hematopoietic | 165/242,213 | 0.78 (0.43, 1.41) | 0.4136 |  | 0.77 (0.43, 1.40) | 0.3938 |  | 0.77 (0.18-1.37) | 0.3998 |
| 379 | Other disorders of eye | sense organs | 570/243,460 | 0.90 (0.69, 1.17) | 0.4157 |  | 0.89 (0.69, 1.16) | 0.4072 |  | 0.89 (0.62-1.15) | 0.3745 |
| 530.1 | Esophagitis, GERD and related diseases | digestive | 721/243,979 | 0.91 (0.71, 1.15) | 0.4213 |  | 0.90 (0.71, 1.15) | 0.4088 |  | 0.90 (0.66-1.14) | 0.4034 |
| 345 | Epilepsy, recurrent seizures, convulsions | neurological | 298/242,836 | 1.16 (0.81, 1.67) | 0.4217 |  | 1.12 (0.78, 1.61) | 0.5467 |  | 1.12 (0.75-1.48) | 0.5575 |
| 575.8 | Other disorders of biliary tract | digestive | 360/210,050 | 0.87 (0.62, 1.22) | 0.4219 |  | 0.87 (0.61, 1.22) | 0.4152 |  | 0.88 (0.53-1.22) | 0.4551 |
| 580.2 | Nephrotic syndrome without mention of glomerulonephritis | genitourinary | 302/235,289 | 0.86 (0.59, 1.25) | 0.423 |  | 0.86 (0.59, 1.26) | 0.4426 |  | 0.86 (0.49-1.24) | 0.4483 |
| 214 | Lipoma | neoplasms | 662/244,040 | 0.88 (0.64, 1.21) | 0.4242 |  | 0.89 (0.64, 1.22) | 0.4546 |  | 0.87 (0.55-1.19) | 0.4023 |
| 535.6 | Duodenitis | digestive | 71/223,512 | 0.67 (0.26, 1.78) | 0.427 |  | 0.67 (0.25, 1.76) | 0.4147 |  | 0.67 (-0.31-1.64) | 0.4140 |
| 228 | Hemangioma and lymphangioma, any site | neoplasms | 232/244,476 | 0.81 (0.47, 1.38) | 0.4308 |  | 0.78 (0.45, 1.34) | 0.3635 |  | 0.81 (0.27-1.35) | 0.4422 |
| 427.21 | Atrial fibrillation | circulatory system | 64/239,292 | 1.30 (0.68, 2.50) | 0.4315 |  | 1.25 (0.65, 2.42) | 0.5018 |  | 1.28 (0.62-1.94) | 0.4622 |
| 395.2 | Nonrheumatic aortic valve disorders | circulatory system | 54/243,375 | 0.70 (0.29, 1.71) | 0.433 |  | 0.70 (0.28, 1.70) | 0.4254 |  | 0.70 (-0.20-1.59) | 0.4341 |
| 427.9 | Palpitations | circulatory system | 1,171/239,292 | 0.92 (0.75, 1.13) | 0.4341 |  | 0.92 (0.75, 1.13) | 0.4175 |  | 0.92 (0.71-1.12) | 0.4053 |
| 70.9 | Hepatitis NOS | infectious diseases | 525/241,496 | 0.88 (0.63, 1.22) | 0.4346 |  | 0.88 (0.63, 1.21) | 0.424 |  | 0.87 (0.54-1.20) | 0.3964 |
| 300 | Anxiety disorders | mental disorders | 1,798/2407,02 | 0.94 (0.80, 1.10) | 0.4362 |  | 0.93 (0.79, 1.09) | 0.3635 |  | 0.92 (0.76-1.08) | 0.2870 |
| 571.5 | Other chronic nonalcoholic liver disease | digestive | 1,521/241,917 | 0.94 (0.79, 1.11) | 0.4384 |  | 0.94 (0.79, 1.11) | 0.4667 |  | 0.94 (0.77-1.11) | 0.4520 |
| 613.1 | Inflammatory disease of breast | genitourinary | 88/244,609 | 1.39 (0.60, 3.23) | 0.4401 |  | 1.37 (0.59, 3.19) | 0.4603 |  | 1.43 (0.58-2.27) | 0.4104 |
| 696 | Psoriasis and related disorders | dermatologic | 45/242,320 | 0.62 (0.18, 2.10) | 0.4466 |  | 0.65 (0.19, 2.19) | 0.4866 |  | 0.64 (-0.58-1.86) | 0.4704 |
| 696.4 | Psoriasis | dermatologic | 45/242,320 | 0.62 (0.18, 2.10) | 0.4466 |  | 0.65 (0.19, 2.19) | 0.4866 |  | 0.64 (-0.58-1.86) | 0.4704 |
| 696.41 | Psoriasis vulgaris | dermatologic | 45/242,320 | 0.62 (0.18, 2.10) | 0.4466 |  | 0.65 (0.19, 2.19) | 0.4866 |  | 0.64 (-0.58-1.86) | 0.4704 |
| 386.2 | Peripheral or central vertigo | sense organs | 428/233,256 | 0.87 (0.62, 1.24) | 0.448 |  | 0.89 (0.63, 1.25) | 0.4981 |  | 0.88 (0.53-1.22) | 0.4644 |
| 189.4 | Malignant neoplasm of other urinary organs | neoplasms | 53/244,225 | 0.72 (0.31, 1.68) | 0.4495 |  | 0.72 (0.31, 1.69) | 0.4491 |  | 0.70 (-0.15-1.55) | 0.4117 |
| 394 | Rheumatic disease of the heart valves | circulatory system | 1,262/243,375 | 0.94 (0.79, 1.11) | 0.4506 |  | 0.91 (0.77, 1.08) | 0.2643 |  | 0.90 (0.73-1.07) | 0.2095 |
| 386 | Vertiginous syndromes and other disorders of vestibular system | sense organs | 3,424/233,256 | 0.96 (0.85, 1.07) | 0.4571 |  | 0.96 (0.85, 1.08) | 0.4724 |  | 0.96 (0.84-1.07) | 0.4526 |
| 212 | Benign neoplasm of respiratory and intrathoracic organs | neoplasms | 31/244,677 | 0.61 (0.17, 2.25) | 0.4592 |  | 0.60 (0.16, 2.20) | 0.4398 |  | 0.60 (-0.70-1.89) | 0.4364 |
| 395.1 | Nonrheumatic mitral valve disorders | circulatory system | 36/243,375 | 1.42 (0.56, 3.62) | 0.4595 |  | 1.41 (0.55, 3.58) | 0.4746 |  | 1.37 (0.44-2.30) | 0.5079 |
| 416 | Cardiomegaly | circulatory system | 76/243,236 | 0.78 (0.41, 1.50) | 0.4607 |  | 0.76 (0.40, 1.46) | 0.4109 |  | 0.76 (0.12-1.41) | 0.4167 |
| 575.7 | Other disorders of gallbladder | digestive | 467/210,050 | 0.86 (0.58, 1.28) | 0.4642 |  | 0.86 (0.58, 1.28) | 0.4695 |  | 0.86 (0.47-1.26) | 0.4656 |
| 250.23 | Type 2 diabetes with ophthalmic manifestations | endocrine/metabolic | 65/219,067 | 0.73 (0.32, 1.69) | 0.4652 |  | 0.71 (0.31, 1.64) | 0.4207 |  | NA | NA |
| 369 | Infection of the eye | sense organs | 1,896/239,750 | 0.94 (0.79, 1.12) | 0.4664 |  | 0.93 (0.79, 1.11) | 0.4428 |  | 0.93 (0.75-1.10) | 0.4050 |
| 427.1 | Paroxysmal tachycardia, unspecified | circulatory system | 299/239,292 | 0.85 (0.55, 1.32) | 0.4684 |  | 0.85 (0.55, 1.32) | 0.4678 |  | 0.86 (0.42-1.30) | 0.4955 |
| 790 | Nonspecific findings on examination of blood | symptoms | 32/244,476 | 0.63 (0.18, 2.23) | 0.4693 |  | 0.64 (0.18, 2.29) | 0.4975 |  | 0.65 (-0.61-1.92) | 0.5130 |
| 327.4 | Insomnia | neurological | 656/243,197 | 1.11 (0.83, 1.48) | 0.4706 |  | 1.11 (0.84, 1.48) | 0.4657 |  | 1.10 (0.82-1.39) | 0.5000 |
| 145.2 | Cancer of tongue | neoplasms | 41/244,316 | 0.69 (0.25, 1.90) | 0.4719 |  | 0.68 (0.24, 1.87) | 0.4517 |  | 0.69 (-0.33-1.71) | 0.4786 |
| 477 | Epistaxis or throat hemorrhage | respiratory | 307/239,202 | 1.14 (0.80, 1.60) | 0.4725 |  | 1.13 (0.80, 1.59) | 0.5035 |  | 1.16 (0.81-1.51) | 0.4092 |
| 801 | Fracture of ankle and foot | injuries & poisonings | 242/236,931 | 0.85 (0.54, 1.33) | 0.4729 |  | 0.84 (0.54, 1.33) | 0.4637 |  | 0.84 (0.38-1.29) | 0.4497 |
| 585.2 | Renal failure NOS | genitourinary | 993/235,289 | 0.94 (0.78, 1.12) | 0.4761 |  | 0.92 (0.77, 1.10) | 0.3459 |  | 0.92 (0.74-1.10) | 0.3423 |
| 274.11 | Gouty arthropathy | endocrine/metabolic | 147/244,134 | 1.17 (0.76, 1.79) | 0.4783 |  | 1.16 (0.75, 1.77) | 0.5059 |  | 1.16 (0.74-1.59) | 0.4855 |
| 599 | Other symptoms/disorders or the urinary system | genitourinary | 366/244,224 | 0.88 (0.62, 1.25) | 0.4821 |  | 0.88 (0.62, 1.25) | 0.4777 |  | 0.87 (0.52-1.22) | 0.4400 |
| 586.4 | Stricture/obstruction of ureter | genitourinary | 173/235,289 | 0.82 (0.46, 1.44) | 0.4836 |  | 0.81 (0.46, 1.42) | 0.4558 |  | 0.79 (0.22-1.36) | 0.4192 |
| 149.2 | Cancer of nasopharynx | neoplasms | 181/244,316 | 0.84 (0.52, 1.37) | 0.486 |  | 0.82 (0.51, 1.34) | 0.4323 |  | 0.86 (0.37-1.34) | 0.5300 |
| 378.5 | Paralytic strabismus | sense organs | 56/243,460 | 0.73 (0.30, 1.79) | 0.4896 |  | 0.73 (0.30, 1.79) | 0.4903 |  | 0.71 (-0.19-1.60) | 0.4469 |
| 226 | Benign neoplasm of thyroid glands | neoplasms | 166/243,929 | 1.21 (0.70, 2.08) | 0.4905 |  | 1.21 (0.70, 2.08) | 0.4948 |  | 1.19 (0.65-1.74) | 0.5262 |
| 700 | Corns and callosities | dermatologic | 30/244,674 | 0.64 (0.18, 2.29) | 0.4961 |  | 0.66 (0.19, 2.36) | 0.526 |  | 0.70 (-0.58-1.97) | 0.5814 |
| 537 | Other disorders of stomach and duodenum | digestive | 187/223,512 | 0.85 (0.53, 1.36) | 0.4981 |  | 0.84 (0.53, 1.35) | 0.4811 |  | 0.82 (0.35-1.30) | 0.4179 |
| 204 | Leukemia | neoplasms | 472/243,740 | 1.10 (0.84, 1.43) | 0.4983 |  | 1.10 (0.84, 1.43) | 0.4924 |  | 1.10 (0.83-1.37) | 0.4749 |
| 332 | Parkinson's disease | neurological | 445/242,836 | 0.92 (0.71, 1.19) | 0.5024 |  | 0.92 (0.71, 1.19) | 0.5072 |  | 0.92 (0.66-1.18) | 0.5317 |
| 442.8 | Aneurysm of other specified artery | circulatory system | 35/244,115 | 1.40 (0.51, 3.85) | 0.5141 |  | 1.35 (0.49, 3.71) | 0.5621 |  | 1.37 (0.36-2.39) | 0.5403 |
| 374 | Other disorders of eyelids | sense organs | 428/239,750 | 0.91 (0.67, 1.22) | 0.517 |  | 0.90 (0.67, 1.21) | 0.4819 |  | 0.90 (0.60-1.19) | 0.4709 |
| 754 | Congenital musculoskeletal deformities of spine | congenital anomalies | 233/244,474 | 0.86 (0.55, 1.35) | 0.5184 |  | 0.89 (0.57, 1.39) | 0.5936 |  | 0.87 (0.41-1.32) | 0.5327 |
| 420.1 | Myocarditis | circulatory system | 412/243,466 | 1.12 (0.80, 1.56) | 0.5194 |  | 1.10 (0.79, 1.54) | 0.5701 |  | 1.10 (0.76-1.43) | 0.5884 |
| 752 | Nervous system congenital anomalies | congenital anomalies | 232/244,456 | 0.86 (0.55, 1.35) | 0.5206 |  | 0.89 (0.57, 1.39) | 0.5968 |  | 0.87 (0.41-1.32) | 0.5343 |
| 752.1 | Neural tube defects | congenital anomalies | 232/244,456 | 0.86 (0.55, 1.35) | 0.5206 |  | 0.89 (0.57, 1.39) | 0.5968 |  | 0.87 (0.41-1.32) | 0.5343 |
| 374.1 | Ectropion or entropion | sense organs | 211/239,750 | 0.88 (0.60, 1.30) | 0.5212 |  | 0.87 (0.59, 1.28) | 0.4895 |  | 0.87 (0.48-1.26) | 0.4898 |
| 306 | Other mental disorder | mental disorders | 101/240,702 | 1.24 (0.64, 2.43) | 0.5248 |  | 1.24 (0.63, 2.42) | 0.5322 |  | 1.23 (0.56-1.90) | 0.5470 |
| 306.9 | Tension headache | mental disorders | 101/240,702 | 1.24 (0.64, 2.43) | 0.5248 |  | 1.24 (0.63, 2.42) | 0.5322 |  | 1.23 (0.56-1.90) | 0.5470 |
| 522 | Diseases of pulp and periapical tissues | digestive | 652/239,130 | 0.90 (0.66, 1.24) | 0.5249 |  | 0.91 (0.66, 1.25) | 0.5541 |  | 0.90 (0.58-1.21) | 0.4950 |
| 496.2 | Chronic bronchitis | respiratory | 23,796/211,409 | 1.01 (0.97, 1.06) | 0.5269 |  | 0.99 (0.95, 1.03) | 0.6602 |  | 0.94 (0.87-1.02) | 0.1245 |
| 530 | Diseases of esophagus | digestive | 729/243,979 | 0.93 (0.73, 1.18) | 0.5282 |  | 0.92 (0.73, 1.17) | 0.5173 |  | 0.92 (0.68-1.16) | 0.5110 |
| 473 | Diseases of the larynx and vocal cords | respiratory | 615/239,202 | 1.11 (0.80, 1.54) | 0.5292 |  | 1.10 (0.79, 1.53) | 0.5648 |  | 1.10 (0.77-1.43) | 0.5734 |
| 611 | Abnormal findings on mammogram or breast exam | genitourinary | 1,125/242,411 | 0.91 (0.69, 1.21) | 0.5322 |  | 0.92 (0.69, 1.22) | 0.5622 |  | 0.94 (0.65-1.22) | 0.6455 |
| 611.3 | Lump or mass in breast | genitourinary | 1,125/242,411 | 0.91 (0.69, 1.21) | 0.5322 |  | 0.92 (0.69, 1.22) | 0.5622 |  | 0.94 (0.65-1.22) | 0.6455 |
| 481 | Influenza | respiratory | 34/222,776 | 0.62 (0.13, 2.84) | 0.5334 |  | 0.62 (0.13, 2.85) | 0.5369 |  | 0.62 (-0.91-2.16) | 0.5460 |
| 574.11 | Cholelithiasis with acute cholecystitis | digestive | 984/210,050 | 0.93 (0.74, 1.17) | 0.5386 |  | 0.93 (0.74, 1.17) | 0.5392 |  | 0.94 (0.71-1.16) | 0.5682 |
| 70.4 | Chronic hepatitis | infectious diseases | 194/241,496 | 1.16 (0.72, 1.87) | 0.5391 |  | 1.17 (0.73, 1.89) | 0.5093 |  | 1.17 (0.70-1.65) | 0.5097 |
| 195.1 | Malignant neoplasm, other | neoplasms | 278/243,065 | 1.11 (0.79, 1.56) | 0.5405 |  | 1.10 (0.78, 1.55) | 0.5778 |  | 1.09 (0.75-1.43) | 0.6023 |
| 573.7 | Abnormal results of function study of liver | digestive | 539/241,917 | 0.91 (0.66, 1.24) | 0.5422 |  | 0.90 (0.66, 1.24) | 0.5248 |  | 0.89 (0.57-1.20) | 0.4525 |
| 510 | Other diseases of lung | respiratory | 84/244,624 | 0.84 (0.47, 1.49) | 0.5426 |  | 0.80 (0.45, 1.42) | 0.4444 |  | 0.80 (0.22-1.38) | 0.4583 |
| 578 | Gastrointestinal hemorrhage | digestive | 1,795/242,763 | 0.96 (0.84, 1.10) | 0.5446 |  | 0.95 (0.83, 1.09) | 0.4601 |  | 0.95 (0.81-1.09) | 0.4712 |
| 241 | Nontoxic nodular goiter | endocrine/metabolic | 1,730/241,973 | 0.95 (0.79, 1.13) | 0.5448 |  | 0.94 (0.79, 1.12) | 0.4921 |  | 0.95 (0.77-1.12) | 0.5487 |
| 798 | Malaise and fatigue | symptoms | 7,349/237,359 | 0.97 (0.88, 1.07) | 0.5477 |  | 0.96 (0.87, 1.06) | 0.4616 |  | 0.96 (0.86-1.06) | 0.3837 |
| 743 | Osteoporosis, osteopenia and pathological fracture | musculoskeletal | 84/244,624 | 0.84 (0.48, 1.47) | 0.5478 |  | 0.85 (0.49, 1.49) | 0.58 |  | 0.84 (0.28-1.40) | 0.5525 |
| 159.3 | Malignant neoplasm of gallbladder and extrahepatic bile ducts | neoplasms | 409/237,409 | 0.92 (0.70, 1.21) | 0.5495 |  | 0.92 (0.70, 1.21) | 0.5618 |  | 0.92 (0.65-1.20) | 0.5709 |
| 507 | Pleurisy; pleural effusion | respiratory | 671/243,046 | 0.93 (0.74, 1.17) | 0.5499 |  | 0.91 (0.73, 1.15) | 0.4303 |  | 0.92 (0.69-1.14) | 0.4492 |
| 535.2 | Atrophic gastritis | digestive | 282/223,512 | 0.87 (0.56, 1.37) | 0.5527 |  | 0.86 (0.55, 1.34) | 0.505 |  | 0.88 (0.43-1.33) | 0.5902 |
| 250.4 | Abnormal glucose | endocrine/metabolic | 862/219,067 | 0.93 (0.72, 1.20) | 0.5532 |  | 0.94 (0.72, 1.21) | 0.6226 |  | 0.87 (0.54-1.20) | 0.4026 |
| 208 | Benign neoplasm of colon | neoplasms | 934/241,904 | 0.94 (0.76, 1.16) | 0.5588 |  | 0.94 (0.76, 1.15) | 0.5426 |  | 0.93 (0.72-1.14) | 0.4830 |
| 442.1 | Aortic aneurysm | circulatory system | 94/244,115 | 0.85 (0.48, 1.50) | 0.5655 |  | 0.83 (0.47, 1.47) | 0.5228 |  | 0.82 (0.25-1.40) | 0.5111 |
| 710 | Osteomyelitis, periostitis, and other infections involving bone | musculoskeletal | 77/242,351 | 0.80 (0.38, 1.71) | 0.569 |  | 0.80 (0.37, 1.69) | 0.5518 |  | 0.78 (0.03-1.54) | 0.5242 |
| 710.1 | Osteomyelitis | musculoskeletal | 77/242,351 | 0.80 (0.38, 1.71) | 0.569 |  | 0.80 (0.37, 1.69) | 0.5518 |  | 0.78 (0.03-1.54) | 0.5242 |
| 710.19 | Unspecified osteomyelitis | musculoskeletal | 77/242,351 | 0.80 (0.38, 1.71) | 0.569 |  | 0.80 (0.37, 1.69) | 0.5518 |  | 0.78 (0.03-1.54) | 0.5242 |
| 502 | Postinflammatory pulmonary fibrosis | respiratory | 63/243,046 | 0.82 (0.41, 1.65) | 0.5723 |  | 0.84 (0.42, 1.69) | 0.6229 |  | 0.85 (0.15-1.55) | 0.6491 |
| 303.1 | Dissociative disorder | mental disorders | 195/240,702 | 0.85 (0.48, 1.50) | 0.5767 |  | 0.83 (0.47, 1.47) | 0.5261 |  | 0.80 (0.23-1.37) | 0.4417 |
| 223 | Benign neoplasm of kidney and other urinary organs | neoplasms | 70/235,186 | 0.76 (0.29, 2.00) | 0.5809 |  | 0.77 (0.29, 2.01) | 0.588 |  | 0.77 (-0.20-1.73) | 0.5888 |
| 567 | Peritonitis and retroperitoneal infections | digestive | 148/242,654 | 1.15 (0.70, 1.91) | 0.5813 |  | 1.14 (0.69, 1.89) | 0.6139 |  | 1.15 (0.64-1.66) | 0.5846 |
| 427.61 | Supraventricular premature beats | circulatory system | 54/239,292 | 0.76 (0.29, 2.00) | 0.5819 |  | 0.76 (0.29, 1.98) | 0.5706 |  | 0.73 (-0.23-1.70) | 0.5286 |
| 170.2 | Cancer of connective tissue | neoplasms | 109/244,440 | 0.83 (0.42, 1.63) | 0.5832 |  | 0.83 (0.42, 1.64) | 0.5964 |  | 0.82 (0.14-1.50) | 0.5682 |
| 362.2 | Degeneration of macula and posterior pole of retina | sense organs | 258/242,577 | 0.89 (0.60, 1.34) | 0.5833 |  | 0.90 (0.60, 1.35) | 0.6152 |  | 0.88 (0.48-1.28) | 0.5356 |
| 362.29 | Macular degeneration (senile) of retina NOS | sense organs | 258/242,577 | 0.89 (0.60, 1.34) | 0.5833 |  | 0.90 (0.60, 1.35) | 0.6152 |  | 0.88 (0.48-1.28) | 0.5356 |
| 585 | Renal failure | genitourinary | 1,579/235,289 | 0.96 (0.83, 1.11) | 0.5884 |  | 0.94 (0.82, 1.09) | 0.4103 |  | 0.94 (0.80-1.09) | 0.4300 |
| 369.5 | Conjunctivitis, infectious | sense organs | 1,737/239,750 | 0.95 (0.79, 1.14) | 0.5898 |  | 0.95 (0.79, 1.14) | 0.5581 |  | 0.94 (0.76-1.12) | 0.5146 |
| 274 | Gout and other crystal arthropathies | endocrine/metabolic | 574/244,134 | 0.93 (0.73, 1.20) | 0.5907 |  | 0.93 (0.73, 1.20) | 0.5777 |  | 0.92 (0.67-1.17) | 0.5333 |
| 274.1 | Gout | endocrine/metabolic | 574/244,134 | 0.93 (0.73, 1.20) | 0.5907 |  | 0.93 (0.73, 1.20) | 0.5777 |  | 0.92 (0.67-1.17) | 0.5333 |
| 70.2 | Viral hepatitis B | infectious diseases | 360/241,496 | 1.11 (0.76, 1.61) | 0.5922 |  | 1.09 (0.75, 1.58) | 0.652 |  | 1.08 (0.71-1.46) | 0.6830 |
| 516 | Abnormal sputum | respiratory | 172/244,536 | 0.88 (0.54, 1.42) | 0.6 |  | 0.85 (0.52, 1.37) | 0.4996 |  | 0.86 (0.37-1.34) | 0.5285 |
| 516.1 | Hemoptysis | respiratory | 172/244,536 | 0.88 (0.54, 1.42) | 0.6 |  | 0.85 (0.52, 1.37) | 0.4996 |  | 0.86 (0.37-1.34) | 0.5285 |
| 358.1 | Myasthenia gravis | neurological | 50/244,308 | 1.28 (0.51, 3.24) | 0.6005 |  | 1.26 (0.50, 3.20) | 0.6227 |  | 1.22 (0.29-2.15) | 0.6727 |
| 530.12 | Ulcer of esophagus | digestive | 58/243,979 | 0.78 (0.31, 1.97) | 0.6059 |  | 0.78 (0.31, 1.95) | 0.5892 |  | 0.81 (-0.11-1.74) | 0.6611 |
| 913 | Toxic effect of venom | injuries & poisonings | 230/244,478 | 1.14 (0.69, 1.90) | 0.6091 |  | 1.13 (0.68, 1.88) | 0.6433 |  | 1.14 (0.63-1.65) | 0.6181 |
| 358 | Myoneural disorders | neurological | 51/244,308 | 1.27 (0.50, 3.21) | 0.6093 |  | 1.25 (0.50, 3.17) | 0.6316 |  | 1.22 (0.29-2.14) | 0.6810 |
| 198.1 | Secondary malignancy of lymph nodes | neoplasms | 131/243,065 | 1.14 (0.69, 1.87) | 0.6108 |  | 1.12 (0.68, 1.84) | 0.6682 |  | 1.13 (0.63-1.63) | 0.6249 |
| 580.13 | Acute glomerulonephritis, NOS | genitourinary | 93/235,289 | 0.83 (0.40, 1.72) | 0.6136 |  | 0.81 (0.39, 1.69) | 0.5775 |  | 0.81 (0.08-1.55) | 0.5853 |
| 195 | Cancer, suspected or other | neoplasms | 320/243,065 | 1.09 (0.79, 1.49) | 0.6142 |  | 1.08 (0.78, 1.48) | 0.6439 |  | 1.07 (0.75-1.39) | 0.6838 |
| 414 | Other forms of chronic heart disease | circulatory system | 1,806/209,210 | 0.97 (0.84, 1.11) | 0.6187 |  | 0.95 (0.83, 1.09) | 0.5002 |  | 0.96 (0.82-1.10) | 0.5819 |
| 337 | Disorders of the autonomic nervous system | neurological | 192/242,836 | 1.13 (0.69, 1.85) | 0.6229 |  | 1.11 (0.68, 1.81) | 0.6763 |  | 1.12 (0.63-1.62) | 0.6520 |
| 189.12 | Malignant neoplasm of renal pelvis | neoplasms | 30/244,225 | 0.76 (0.25, 2.31) | 0.6257 |  | 0.76 (0.25, 2.33) | 0.6342 |  | 0.77 (-0.35-1.88) | 0.6429 |
| 371.1 | Uveitis, noninfectious or NOS | sense organs | 199/239,750 | 1.12 (0.70, 1.78) | 0.6309 |  | 1.10 (0.69, 1.76) | 0.675 |  | 1.09 (0.62-1.56) | 0.7178 |
| 281.1 | Megaloblastic anemia | hematopoietic | 36/242,213 | 1.25 (0.50, 3.13) | 0.6357 |  | 1.23 (0.49, 3.09) | 0.6529 |  | 1.23 (0.31-2.15) | 0.6641 |
| 362.4 | Retinal vascular changes and abnomalities | sense organs | 34/242,577 | 1.27 (0.47, 3.42) | 0.6383 |  | 1.24 (0.46, 3.36) | 0.667 |  | 1.23 (0.24-2.23) | 0.6790 |
| 204.12 | Lymphoid leukemia, chronic | neoplasms | 36/243,740 | 0.80 (0.30, 2.08) | 0.6415 |  | 0.82 (0.31, 2.17) | 0.6953 |  | 0.82 (-0.15-1.79) | 0.6853 |
| 411.2 | Myocardial infarction | circulatory system | 753/209,210 | 0.96 (0.79, 1.15) | 0.6449 |  | 0.93 (0.77, 1.13) | 0.4778 |  | 0.94 (0.75-1.13) | 0.4948 |
| 509.2 | Respiratory insufficiency | respiratory | 659/243,046 | 1.05 (0.85, 1.30) | 0.6451 |  | 1.01 (0.81, 1.24) | 0.9558 |  | 0.99 (0.78-1.20) | 0.9155 |
| 41.21 | Rheumatic fever / chorea | infectious diseases | 69/240,,464 | 1.17 (0.59, 2.32) | 0.6466 |  | 1.19 (0.60, 2.36) | 0.6133 |  | 1.16 (0.48-1.85) | 0.6648 |
| 348 | Other conditions of brain | neurological | 230/242836 | 0.91 (0.60, 1.38) | 0.6471 |  | 0.89 (0.58, 1.35) | 0.5697 |  | 0.87 (0.45-1.29) | 0.5203 |
| 153.2 | Colon cancer | neoplasms | 1,209/238,002 | 1.04 (0.89, 1.22) | 0.6482 |  | 1.03 (0.88, 1.21) | 0.7304 |  | 1.07 (0.91-1.23) | 0.4160 |
| 427.4 | Cardiac arrest and ventricular fibrillation | circulatory system | 53/239,292 | 1.16 (0.61, 2.18) | 0.6501 |  | 1.12 (0.60, 2.11) | 0.7191 |  | 1.14 (0.50-1.77) | 0.6938 |
| 427.42 | Cardiac arrest | circulatory system | 53/239,292 | 1.16 (0.61, 2.18) | 0.6501 |  | 1.12 (0.60, 2.11) | 0.7191 |  | 1.14 (0.50-1.77) | 0.6938 |
| 348.2 | Cerebral edema and compression of brain | neurological | 41/242,836 | 1.22 (0.52, 2.87) | 0.6549 |  | 1.19 (0.50, 2.81) | 0.6962 |  | 1.22 (0.36-2.09) | 0.6469 |
| 580.1 | Glomerulonephritis | genitourinary | 777/235,289 | 0.95 (0.75, 1.20) | 0.6629 |  | 0.94 (0.75, 1.19) | 0.62 |  | 0.94 (0.70-1.17) | 0.5781 |
| 401.21 | Hypertensive heart disease | circulatory system | 1,261/133,117 | 0.97 (0.83, 1.13) | 0.6649 |  | 0.97 (0.83, 1.13) | 0.7203 |  | 0.87 (0.53-1.22) | 0.4394 |
| 386.21 | Central origin vertigo | sense organs | 233/233,256 | 0.90 (0.55, 1.46) | 0.6657 |  | 0.91 (0.56, 1.48) | 0.7005 |  | 0.92 (0.43-1.40) | 0.7218 |
| 323 | Encephalitis | neurological | 68/244,623 | 0.84 (0.38, 1.85) | 0.666 |  | 0.83 (0.38, 1.83) | 0.647 |  | 0.87 (0.08-1.67) | 0.7405 |
| 575.1 | Cholangitis | digestive | 354/210,050 | 0.93 (0.66, 1.31) | 0.6666 |  | 0.93 (0.66, 1.32) | 0.6907 |  | 0.92 (0.57-1.27) | 0.6406 |
| 340 | Migraine | neurological | 396/239,140 | 1.08 (0.76, 1.54) | 0.6676 |  | 1.08 (0.76, 1.54) | 0.6688 |  | 1.07 (0.71-1.42) | 0.7178 |
| 371.3 | Inflammation of eyelids | sense organs | 147/239,750 | 1.13 (0.64, 2.01) | 0.6679 |  | 1.15 (0.65, 2.03) | 0.6345 |  | 1.16 (0.58-1.73) | 0.6174 |
| 800.2 | Fracture of unspecified part of femur | injuries & poisonings | 732/236,931 | 0.96 (0.79, 1.16) | 0.6763 |  | 0.95 (0.78, 1.15) | 0.6109 |  | 0.95 (0.76-1.14) | 0.6044 |
| 577.2 | Chronic pancreatitis | digestive | 63/243,713 | 0.82 (0.33, 2.05) | 0.6782 |  | 0.82 (0.33, 2.05) | 0.6703 |  | 0.82 (-0.09-1.74) | 0.6788 |
| 155.1 | Malignant neoplasm of liver, primary | neoplasms | 144/237,409 | 0.91 (0.57, 1.44) | 0.6789 |  | 0.90 (0.57, 1.44) | 0.6687 |  | 0.91 (0.45-1.38) | 0.7043 |
| 352.2 | Facial nerve disorders [CN7] | neurological | 907/243,158 | 0.95 (0.77, 1.19) | 0.6809 |  | 0.96 (0.77, 1.20) | 0.7416 |  | 0.96 (0.73-1.18) | 0.6872 |
| 509.1 | Respiratory failure | respiratory | 665/243,046 | 1.04 (0.85, 1.29) | 0.6823 |  | 1.00 (0.81, 1.23) | 0.9958 |  | 0.98 (0.77-1.19) | 0.8756 |
| 296 | Mood disorders | mental disorders | 502/240,702 | 0.94 (0.68, 1.28) | 0.6836 |  | 0.92 (0.67, 1.26) | 0.6183 |  | 0.89 (0.58-1.21) | 0.4819 |
| 427.3 | Other specified cardiac dysrhythmias | circulatory system | 320/239,292 | 0.93 (0.64, 1.34) | 0.6854 |  | 0.92 (0.63, 1.33) | 0.6501 |  | 0.93 (0.55-1.30) | 0.6834 |
| 509 | Respiratory failure, insufficiency, arrest | respiratory | 675/243,046 | 1.04 (0.85, 1.28) | 0.689 |  | 1.00 (0.81, 1.23) | 0.9981 |  | 0.98 (0.77-1.19) | 0.8653 |
| 426 | Cardiac conduction disorders | circulatory system | 112/239,292 | 0.90 (0.52, 1.56) | 0.6971 |  | 0.88 (0.50, 1.53) | 0.6457 |  | 0.87 (0.32-1.43) | 0.6354 |
| 41 | Bacterial infection NOS | infectious diseases | 100/240,464 | 1.12 (0.63, 1.98) | 0.7033 |  | 1.12 (0.63, 1.99) | 0.6917 |  | 1.11 (0.53-1.68) | 0.7296 |
| 580.14 | Chronic glomerulonephritis, NOS | genitourinary | 622/235,289 | 1.05 (0.81, 1.35) | 0.7068 |  | 1.05 (0.81, 1.35) | 0.7309 |  | 1.04 (0.78-1.29) | 0.7830 |
| 473.1 | Chronic laryngitis | respiratory | 141/239,202 | 1.14 (0.58, 2.22) | 0.7069 |  | 1.16 (0.59, 2.27) | 0.6666 |  | 1.16 (0.49-1.83) | 0.6670 |
| 204.21 | Myeloid leukemia, acute | neoplasms | 70/243,740 | 1.15 (0.54, 2.48) | 0.7136 |  | 1.16 (0.54, 2.50) | 0.698 |  | 1.16 (0.39-1.93) | 0.7048 |
| 260 | Protein-calorie malnutrition | endocrine/metabolic | 1,145/243,559 | 1.04 (0.83, 1.31) | 0.7138 |  | 1.04 (0.83, 1.31) | 0.7262 |  | 1.05 (0.82-1.28) | 0.6588 |
| 564 | Functional digestive disorders | digestive | 80/242,736 | 1.14 (0.57, 2.29) | 0.7148 |  | 1.06 (0.52, 2.13) | 0.8789 |  | 0.98 (0.28-1.69) | 0.9624 |
| 224 | Benign neoplasm of eye | neoplasms | 344/243,789 | 0.94 (0.69, 1.29) | 0.7157 |  | 0.94 (0.69, 1.28) | 0.7063 |  | 0.94 (0.63-1.25) | 0.6905 |
| 523.31 | Acute periodontitis | digestive | 281/239,130 | 1.08 (0.72, 1.62) | 0.7163 |  | 1.10 (0.73, 1.66) | 0.6439 |  | 1.10 (0.69-1.51) | 0.6518 |
| 743.13 | Other specified osteoporosis | musculoskeletal | 75/244,624 | 0.90 (0.50, 1.61) | 0.7181 |  | 0.90 (0.50, 1.61) | 0.7278 |  | 0.89 (0.31-1.47) | 0.6903 |
| 352 | Disorders of other cranial nerves | neurological | 1,100/243,158 | 0.96 (0.79, 1.17) | 0.72 |  | 0.97 (0.80, 1.18) | 0.7794 |  | 0.97 (0.77-1.16) | 0.7262 |
| 743.1 | Osteoporosis | musculoskeletal | 81/244,624 | 0.90 (0.51, 1.59) | 0.7201 |  | 0.91 (0.52, 1.61) | 0.7537 |  | 0.90 (0.34-1.46) | 0.7139 |
| 70.3 | Viral hepatitis C | infectious diseases | 75/241,496 | 1.14 (0.56, 2.34) | 0.7208 |  | 1.14 (0.55, 2.33) | 0.7255 |  | 1.17 (0.45-1.89) | 0.6653 |
| 470 | Septal Deviations/Turbinate Hypertrophy | respiratory | 230/239,202 | 1.09 (0.68, 1.75) | 0.7213 |  | 1.10 (0.69, 1.77) | 0.6814 |  | 1.10 (0.63-1.58) | 0.6829 |
| 367 | Disorders of refraction and accommodation; blindness and low vision | sense organs | 119/244,589 | 1.13 (0.58, 2.20) | 0.728 |  | 1.15 (0.59, 2.24) | 0.6876 |  | 1.13 (0.46-1.80) | 0.7181 |
| 41.2 | Streptococcus infection | infectious diseases | 72/240,464 | 1.13 (0.57, 2.23) | 0.7299 |  | 1.14 (0.58, 2.25) | 0.7039 |  | 1.10 (0.42-1.78) | 0.7862 |
| 585.3 | Chronic renal failure [CKD] | genitourinary | 910/235,289 | 0.97 (0.80, 1.17) | 0.7317 |  | 0.95 (0.78, 1.15) | 0.5786 |  | 0.95 (0.76-1.14) | 0.6045 |
| 751 | Genitourinary congenital anomalies | congenital anomalies | 62/244,629 | 1.17 (0.48, 2.87) | 0.7325 |  | 1.17 (0.48, 2.87) | 0.7301 |  | 1.15 (0.26-2.05) | 0.7535 |
| 751.2 | Congenital anomalies of urinary system | congenital anomalies | 62/244,629 | 1.17 (0.48, 2.87) | 0.7325 |  | 1.17 (0.48, 2.87) | 0.7301 |  | 1.15 (0.26-2.05) | 0.7535 |
| 580.3 | Nephritis and nephropathy without mention of glomerulonephritis | genitourinary | 483/235,289 | 0.94 (0.67, 1.32) | 0.7336 |  | 0.94 (0.67, 1.32) | 0.7051 |  | 0.93 (0.58-1.27) | 0.6589 |
| 580.32 | Nephritis and nephropathy with pathological lesion | genitourinary | 483/235,289 | 0.94 (0.67, 1.32) | 0.7336 |  | 0.94 (0.67, 1.32) | 0.7051 |  | 0.93 (0.58-1.27) | 0.6589 |
| 194 | Cancer of other endocrine glands | neoplasms | 51/243,929 | 0.84 (0.31, 2.26) | 0.7364 |  | 0.84 (0.31, 2.26) | 0.7275 |  | 0.81 (-0.18-1.80) | 0.6742 |
| 352.1 | Trigeminal nerve disorders [CN5] | neurological | 196/243,158 | 1.08 (0.70, 1.65) | 0.7392 |  | 1.08 (0.70, 1.65) | 0.7294 |  | 1.08 (0.65-1.51) | 0.7229 |
| 351 | Other peripheral nerve disorders | neurological | 395/243,158 | 0.94 (0.65, 1.36) | 0.7491 |  | 0.94 (0.65, 1.36) | 0.7407 |  | 0.93 (0.56-1.30) | 0.7079 |
| 401.2 | Hypertensive heart and/or renal disease | circulatory system | 1,452/133,117 | 0.98 (0.85, 1.13) | 0.7512 |  | 0.98 (0.85, 1.13) | 0.7623 |  | 0.93 (0.60-1.25) | 0.6521 |
| 695 | Erythematous conditions | dermatologic | 168/24,4473 | 1.09 (0.63, 1.90) | 0.7518 |  | 1.09 (0.62, 1.89) | 0.7686 |  | 1.05 (0.49-1.60) | 0.8688 |
| 531.2 | Gastric ulcer | digestive | 1,408/233,738 | 0.97 (0.82, 1.16) | 0.7619 |  | 0.96 (0.81, 1.14) | 0.6273 |  | 0.95 (0.78-1.12) | 0.5484 |
| 395 | Heart valve disorders | circulatory system | 91/243,375 | 0.91 (0.48, 1.71) | 0.7643 |  | 0.90 (0.48, 1.69) | 0.7364 |  | 0.89 (0.26-1.52) | 0.7184 |
| 575.9 | Nonspecific abnormal findings on radiological and other examination of biliary tract | digestive | 95/210,050 | 0.91 (0.51, 1.64) | 0.7651 |  | 0.91 (0.51, 1.64) | 0.761 |  | 0.92 (0.34-1.51) | 0.7880 |
| 585.1 | Acute renal failure | genitourinary | 70/235,289 | 1.10 (0.59, 2.03) | 0.7657 |  | 1.06 (0.57, 1.97) | 0.8457 |  | 1.09 (0.47-1.70) | 0.7954 |
| 164 | Cancer of intrathoracic organs | neoplasms | 60/241,965 | 1.13 (0.50, 2.59) | 0.7667 |  | 1.14 (0.50, 2.62) | 0.753 |  | 1.13 (0.30-1.96) | 0.7763 |
| 290 | Delirium dementia and amnestic and other cognitive disorders | mental disorders | 284/244,423 | 1.04 (0.79, 1.37) | 0.77 |  | 1.03 (0.78, 1.36) | 0.8318 |  | 1.02 (0.75-1.30) | 0.8684 |
| 189.1 | Cancer of kidney and renal pelvis | neoplasms | 254/244,225 | 0.95 (0.65, 1.38) | 0.7724 |  | 0.94 (0.65, 1.37) | 0.7485 |  | 0.95 (0.57-1.32) | 0.7741 |
| 202.2 | Non-Hodgkins lymphoma | neoplasms | 467/243,740 | 1.04 (0.78, 1.39) | 0.7724 |  | 1.04 (0.78, 1.38) | 0.7936 |  | 1.05 (0.76-1.33) | 0.7480 |
| 379.1 | Scleritis and episcleritis | sense organs | 57/243,460 | 0.88 (0.36, 2.13) | 0.7743 |  | 0.89 (0.37, 2.15) | 0.791 |  | 0.88 (-0.01-1.76) | 0.7740 |
| 420 | Carditis | circulatory system | 855/243,466 | 1.03 (0.83, 1.27) | 0.7814 |  | 1.01 (0.82, 1.25) | 0.9083 |  | 1.02 (0.81-1.23) | 0.8675 |
| 280.2 | Iron deficiency anemia secondary to blood loss (chronic) | hematopoietic | 82/242,213 | 1.11 (0.54, 2.25) | 0.7818 |  | 1.12 (0.55, 2.28) | 0.7603 |  | 1.10 (0.39-1.81) | 0.7934 |
| 578.9 | Hemorrhage of gastrointestinal tract | digestive | 1,631/242,763 | 0.98 (0.85, 1.13) | 0.7833 |  | 0.97 (0.84, 1.12) | 0.6659 |  | 0.97 (0.83-1.11) | 0.6797 |
| 751.21 | Cystic kidney disease | congenital anomalies | 53/244,629 | 1.15 (0.43, 3.06) | 0.7844 |  | 1.16 (0.43, 3.09) | 0.7701 |  | 1.13 (0.15-2.11) | 0.8108 |
| 803.2 | Fracture of radius and ulna | injuries & poisonings | 1,857/236,931 | 0.98 (0.85, 1.13) | 0.789 |  | 0.98 (0.84, 1.13) | 0.7559 |  | 0.97 (0.83-1.12) | 0.7077 |
| 245 | Thyroiditis | endocrine/metabolic | 225/241,973 | 0.93 (0.53, 1.61) | 0.7893 |  | 0.93 (0.53, 1.62) | 0.7963 |  | 0.94 (0.39-1.50) | 0.8377 |
| 70 | Viral hepatitis | infectious diseases | 762/241,496 | 0.97 (0.75, 1.25) | 0.7934 |  | 0.96 (0.74, 1.24) | 0.7354 |  | 0.95 (0.69-1.21) | 0.6868 |
| 916 | Contusion | injuries & poisonings | 504/244,204 | 1.05 (0.75, 1.47) | 0.7953 |  | 1.04 (0.74, 1.46) | 0.8181 |  | 1.01 (0.67-1.35) | 0.9363 |
| 939 | Atopic/contact dermatitis due to other or unspecified | dermatologic | 2,104/241,916 | 0.98 (0.83, 1.15) | 0.7963 |  | 0.99 (0.84, 1.16) | 0.8552 |  | 0.99 (0.83-1.15) | 0.9204 |
| 370.1 | Corneal ulcer | sense organs | 90/239,750 | 1.08 (0.59, 1.98) | 0.8044 |  | 1.10 (0.60, 2.01) | 0.7654 |  | 1.06 (0.45-1.66) | 0.8616 |
| 202 | Cancer of other lymphoid, histiocytic tissue | neoplasms | 468/243,740 | 1.04 (0.78, 1.38) | 0.8053 |  | 1.03 (0.78, 1.37) | 0.8255 |  | 1.04 (0.76-1.33) | 0.7778 |
| 531.3 | Duodenal ulcer | digestive | 414/233,738 | 0.96 (0.70, 1.32) | 0.8064 |  | 0.95 (0.69, 1.30) | 0.7549 |  | 0.95 (0.64-1.26) | 0.7499 |
| 727.4 | Ganglion and cyst of synovium, tendon, and bursa | musculoskeletal | 235/242,934 | 1.06 (0.64, 1.77) | 0.8078 |  | 1.05 (0.63, 1.75) | 0.845 |  | 1.07 (0.56-1.58) | 0.7854 |
| 800.1 | Fracture of neck of femur | injuries & poisonings | 523/236,931 | 1.03 (0.83, 1.26) | 0.8109 |  | 1.04 (0.84, 1.27) | 0.7432 |  | 1.04 (0.83-1.24) | 0.7410 |
| 428.2 | Heart failure NOS | circulatory system | 197/242,460 | 1.04 (0.73, 1.49) | 0.811 |  | 1.00 (0.70, 1.42) | 0.9879 |  | 1.00 (0.64-1.36) | 0.9923 |
| 471 | Nasal polyps | respiratory | 270/239,202 | 1.06 (0.67, 1.65) | 0.8115 |  | 1.06 (0.68, 1.66) | 0.803 |  | 1.06 (0.61-1.51) | 0.8150 |
| 200 | Myeloproliferative disease | neoplasms | 75/243,740 | 1.08 (0.56, 2.09) | 0.8125 |  | 1.08 (0.56, 2.09) | 0.8135 |  | 1.09 (0.43-1.75) | 0.7967 |
| 251.1 | Hypoglycemia | endocrine/metabolic | 282/218,933 | 0.96 (0.70, 1.32) | 0.8152 |  | 0.93 (0.68, 1.28) | 0.6722 |  | 1.15 (0.68-1.62) | 0.5622 |
| 198.2 | Secondary malignancy of respiratory organs | neoplasms | 74/243,065 | 1.08 (0.55, 2.11) | 0.8231 |  | 1.03 (0.53, 2.01) | 0.9292 |  | 1.03 (0.36-1.70) | 0.9366 |
| 195.3 | Malignant neoplasm of head, face, and neck | neoplasms | 35/243,065 | 0.89 (0.32, 2.46) | 0.8248 |  | 0.89 (0.32, 2.46) | 0.8215 |  | 0.89 (-0.13-1.91) | 0.8246 |
| 446.9 | Arteritis NOS | circulatory system | 78/244,115 | 1.08 (0.55, 2.10) | 0.8258 |  | 1.07 (0.55, 2.09) | 0.833 |  | 1.03 (0.37-1.70) | 0.9199 |
| 715 | Other inflammatory spondylopathies | musculoskeletal | 70/242,429 | 0.89 (0.30, 2.59) | 0.827 |  | 0.89 (0.30, 2.59) | 0.825 |  | 0.87 (-0.21-1.95) | 0.8042 |
| 715.2 | Ankylosing spondylitis | musculoskeletal | 70/242,429 | 0.89 (0.30, 2.59) | 0.827 |  | 0.89 (0.30, 2.59) | 0.825 |  | 0.87 (-0.21-1.95) | 0.8042 |
| 565.1 | Anal and rectal polyp | digestive | 309/242,654 | 1.04 (0.72, 1.52) | 0.8277 |  | 1.03 (0.71, 1.50) | 0.879 |  | 1.02 (0.65-1.40) | 0.9020 |
| 214.1 | Lipoma of skin and subcutaneous tissue | neoplasms | 58/244,040 | 0.88 (0.25, 3.01) | 0.8335 |  | 0.91 (0.27, 3.14) | 0.8868 |  | 0.92 (-0.32-2.15) | 0.8887 |
| 442 | Other aneurysm | circulatory system | 244/244,115 | 0.96 (0.66, 1.40) | 0.8346 |  | 0.94 (0.64, 1.37) | 0.7407 |  | 0.94 (0.56-1.32) | 0.7573 |
| 360 | Disorders of the globe | sense organs | 33/242,577 | 0.89 (0.29, 2.73) | 0.8351 |  | 0.87 (0.28, 2.67) | 0.8018 |  | 0.89 (-0.24-2.02) | 0.8355 |
| 594.3 | Calculus of ureter | genitourinary | 2,662/237,706 | 0.98 (0.85, 1.14) | 0.8369 |  | 1.00 (0.86, 1.15) | 0.9477 |  | 0.99 (0.84-1.14) | 0.9187 |
| 711.1 | Pyogenic arthritis | musculoskeletal | 44/242,351 | 1.09 (0.47, 2.52) | 0.8408 |  | 1.10 (0.47, 2.54) | 0.827 |  | 1.14 (0.30-1.99) | 0.7536 |
| 727 | Other disorders of synovium, tendon, and bursa | musculoskeletal | 238/242,934 | 1.05 (0.63, 1.75) | 0.843 |  | 1.04 (0.63, 1.73) | 0.877 |  | 1.06 (0.55-1.57) | 0.8139 |
| 496 | Chronic airway obstruction | respiratory | 25,106/211,409 | 1.00 (0.96, 1.05) | 0.847 |  | 0.98 (0.94, 1.02) | 0.376 |  | 0.93 (0.86-0.99) | 0.0273 |
| 189.11 | Malignant neoplasm of kidney, except pelvis | neoplasms | 234/244,225 | 0.96 (0.65, 1.42) | 0.8477 |  | 0.96 (0.65, 1.42) | 0.8286 |  | 0.96 (0.57-1.36) | 0.8556 |
| 446 | Polyarteritis nodosa and allied conditions | circulatory system | 85/244,115 | 1.06 (0.56, 2.02) | 0.8482 |  | 1.07 (0.56, 2.03) | 0.8422 |  | 1.03 (0.39-1.67) | 0.9295 |
| 560.3 | Peritoneal or intestinal adhesions | digestive | 35/242,736 | 0.91 (0.34, 2.40) | 0.8483 |  | 0.87 (0.33, 2.30) | 0.7776 |  | 0.90 (-0.08-1.88) | 0.8333 |
| 474 | Acute and chronic tonsillitis | respiratory | 1,846/239,202 | 1.02 (0.85, 1.22) | 0.8524 |  | 1.02 (0.85, 1.23) | 0.8232 |  | 1.03 (0.84-1.21) | 0.7940 |
| 686 | Other local infections of skin and subcutaneous tissue | dermatologic | 52/244,646 | 1.08 (0.47, 2.51) | 0.8525 |  | 1.07 (0.46, 2.49) | 0.8685 |  | 1.09 (0.24-1.93) | 0.8445 |
| 569 | Other disorders of intestine | digestive | 552/242,654 | 0.98 (0.75, 1.27) | 0.8539 |  | 0.95 (0.73, 1.24) | 0.7176 |  | 0.94 (0.68-1.20) | 0.6459 |
| 189 | Cancer of urinary organs (incl. kidney and bladder) | neoplasms | 483/244,225 | 0.98 (0.75, 1.27) | 0.8578 |  | 0.97 (0.74, 1.25) | 0.7918 |  | 0.97 (0.70-1.23) | 0.7948 |
| 348.8 | Encephalopathy, not elsewhere classified | neurological | 173/242,836 | 0.96 (0.59, 1.56) | 0.8601 |  | 0.94 (0.57, 1.53) | 0.7937 |  | 0.91 (0.42-1.40) | 0.7033 |
| 331 | Other cerebral degenerations | neurological | 381/242,836 | 1.02 (0.79, 1.33) | 0.8613 |  | 1.01 (0.78, 1.31) | 0.9585 |  | 1.02 (0.76-1.28) | 0.8882 |
| 189.2 | Cancer of bladder | neoplasms | 198/244,225 | 1.03 (0.71, 1.52) | 0.8614 |  | 1.02 (0.69, 1.50) | 0.9274 |  | 1.02 (0.64-1.41) | 0.9089 |
| 198.4 | Secondary malignant neoplasm of liver | neoplasms | 37/243,065 | 1.09 (0.41, 2.86) | 0.8652 |  | 1.06 (0.40, 2.80) | 0.9004 |  | 1.08 (0.10-2.05) | 0.8838 |
| 695.42 | Systemic lupus erythematosus | dermatologic | 143/244,471 | 0.94 (0.49, 1.83) | 0.8657 |  | 0.94 (0.49, 1.82) | 0.8611 |  | 0.89 (0.23-1.56) | 0.7416 |
| 592.11 | Acute cystitis | genitourinary | 314/230,026 | 0.97 (0.66, 1.42) | 0.8658 |  | 0.97 (0.66, 1.43) | 0.8935 |  | 0.97 (0.59-1.36) | 0.8899 |
| 401.3 | Other hypertensive complications | circulatory system | 273/133,117 | 1.03 (0.71, 1.49) | 0.868 |  | 1.01 (0.70, 1.47) | 0.941 |  | 1.42 (0.66-2.18) | 0.3669 |
| 711 | Arthropathy associated with infections | musculoskeletal | 45/242,351 | 1.07 (0.46, 2.48) | 0.8686 |  | 1.08 (0.47, 2.50) | 0.8522 |  | 1.13 (0.29-1.97) | 0.7762 |
| 368.9 | Subjective visual disturbances | sense organs | 83/244,587 | 1.09 (0.38, 3.14) | 0.8701 |  | 1.08 (0.38, 3.12) | 0.8811 |  | 1.08 (0.02-2.14) | 0.8862 |
| 333 | Extrapyramidal disease and abnormal movement disorders | neurological | 68/242,836 | 1.06 (0.51, 2.21) | 0.871 |  | 1.10 (0.53, 2.29) | 0.8012 |  | 1.11 (0.38-1.85) | 0.7743 |
| 577.3 | Cyst and pseudocyst of pancreas | digestive | 34/243,713 | 1.08 (0.42, 2.81) | 0.8759 |  | 1.00 (0.38, 2.62) | 0.9939 |  | 1.01 (0.05-1.97) | 0.9829 |
| 523.1 | Gingivitis | digestive | 255/239,130 | 0.96 (0.61, 1.53) | 0.8799 |  | 0.96 (0.60, 1.52) | 0.8597 |  | 0.98 (0.52-1.45) | 0.9354 |
| 151 | Cancer of stomach | neoplasms | 1,337/237,409 | 1.01 (0.87, 1.18) | 0.8809 |  | 1.00 (0.86, 1.16) | 0.9837 |  | 1.02 (0.87-1.17) | 0.8287 |
| 204.4 | Multiple myeloma | neoplasms | 119/243,740 | 1.04 (0.61, 1.77) | 0.8814 |  | 1.06 (0.62, 1.81) | 0.8272 |  | 1.06 (0.52-1.60) | 0.8303 |
| 401.22 | Hypertensive chronic kidney disease | circulatory system | 193/133,117 | 0.97 (0.63, 1.50) | 0.8862 |  | 0.97 (0.62, 1.51) | 0.8914 |  | 1.52 (0.55-2.50) | 0.3976 |
| 287.31 | Primary thrombocytopenia | hematopoietic | 61/244,217 | 0.94 (0.41, 2.15) | 0.889 |  | 0.93 (0.41, 2.13) | 0.8622 |  | 0.93 (0.10-1.76) | 0.8576 |
| 803.3 | Fracture of clavicle or scapula | injuries & poisonings | 367/236,931 | 0.97 (0.67, 1.42) | 0.8922 |  | 0.99 (0.68, 1.44) | 0.9462 |  | 1.01 (0.63-1.39) | 0.9538 |
| 687 | Symptoms affecting skin | dermatologic | 95/244,613 | 0.95 (0.44, 2.05) | 0.8924 |  | 0.95 (0.44, 2.05) | 0.8965 |  | 1.00 (0.23-1.77) | 0.9931 |
| 293.1 | Swelling, mass, or lump in head and neck [Space-occupying lesion, intracranial NOS] | mental disorders | 254/244,444 | 1.03 (0.69, 1.53) | 0.8938 |  | 1.01 (0.68, 1.51) | 0.9586 |  | 1.02 (0.62-1.42) | 0.9099 |
| 709 | Diffuse diseases of connective tissue | dermatologic | 177/244,304 | 0.96 (0.53, 1.75) | 0.8954 |  | 0.94 (0.51, 1.70) | 0.8277 |  | 0.92 (0.32-1.52) | 0.7883 |
| 290.13 | Senile dementia | mental disorders | 141/244,423 | 0.97 (0.64, 1.47) | 0.8966 |  | 0.97 (0.64, 1.47) | 0.8826 |  | 0.97 (0.55-1.38) | 0.8797 |
| 53.1 | Herpes zoster with nervous system complications | infectious diseases | 50/241,496 | 0.95 (0.41, 2.19) | 0.9024 |  | 0.90 (0.39, 2.07) | 0.8018 |  | 0.92 (0.08-1.76) | 0.8540 |
| 394.2 | Mitral valve disease | circulatory system | 777/243,375 | 1.01 (0.82, 1.25) | 0.9072 |  | 0.98 (0.79, 1.21) | 0.8488 |  | 0.96 (0.75-1.18) | 0.7212 |
| 411.3 | Angina pectoris | circulatory system | 1,246/209,210 | 0.99 (0.85, 1.16) | 0.9086 |  | 0.98 (0.84, 1.15) | 0.7888 |  | 0.98 (0.82-1.14) | 0.8386 |
| 79.9 | Viremia, NOS | infectious diseases | 34/241,496 | 0.94 (0.30, 2.93) | 0.9141 |  | 0.97 (0.31, 3.03) | 0.9627 |  | 0.96 (-0.19-2.11) | 0.9465 |
| 289.4 | Lymphadenitis | hematopoietic | 451/243,568 | 1.02 (0.70, 1.48) | 0.9149 |  | 1.03 (0.71, 1.50) | 0.8639 |  | 1.03 (0.66-1.40) | 0.8768 |
| 371 | Inflammation of the eye | sense organs | 398/239,750 | 1.02 (0.72, 1.43) | 0.9197 |  | 1.02 (0.72, 1.43) | 0.9233 |  | 1.01 (0.67-1.35) | 0.9631 |
| 706 | Diseases of sebaceous glands | dermatologic | 37/244,671 | 0.94 (0.28, 3.21) | 0.9219 |  | 0.96 (0.28, 3.28) | 0.9507 |  | 0.90 (-0.34-2.14) | 0.8687 |
| 706.2 | Sebaceous cyst | dermatologic | 37/244,671 | 0.94 (0.28, 3.21) | 0.9219 |  | 0.96 (0.28, 3.28) | 0.9507 |  | 0.90 (-0.34-2.14) | 0.8687 |
| 525 | Other diseases of the teeth and supporting structures | digestive | 91/239,130 | 0.97 (0.54, 1.74) | 0.9232 |  | 1.02 (0.57, 1.84) | 0.9345 |  | 1.08 (0.49-1.68) | 0.7901 |
| 695.4 | Lupus (localized and systemic) | dermatologic | 146/244,471 | 1.03 (0.55, 1.95) | 0.9243 |  | 1.03 (0.55, 1.94) | 0.9296 |  | 0.97 (0.33-1.61) | 0.9263 |
| 728.7 | Fasciitis | musculoskeletal | 141/242,934 | 0.97 (0.50, 1.86) | 0.9254 |  | 0.97 (0.51, 1.87) | 0.9326 |  | 0.97 (0.32-1.63) | 0.9368 |
| 374.3 | Ptosis of eyelid | sense organs | 39/239,750 | 0.96 (0.36, 2.55) | 0.9274 |  | 0.93 (0.35, 2.48) | 0.881 |  | 0.95 (-0.03-1.94) | 0.9258 |
| 782.3 | Edema | symptoms | 447/244,261 | 0.99 (0.73, 1.34) | 0.9284 |  | 0.99 (0.73, 1.34) | 0.9392 |  | 0.97 (0.66-1.28) | 0.8565 |
| 440.2 | Atherosclerosis of the extremities | circulatory system | 136/244,115 | 1.02 (0.65, 1.60) | 0.9299 |  | 0.99 (0.63, 1.56) | 0.9725 |  | 1.00 (0.54-1.45) | 0.9930 |
| 378 | Strabismus and other disorders of binocular eye movements | sense organs | 87/243,460 | 0.97 (0.48, 1.96) | 0.9309 |  | 0.97 (0.48, 1.96) | 0.9307 |  | 0.96 (0.25-1.67) | 0.9103 |
| 522.5 | Periapical abscess | digestive | 38/239,130 | 0.96 (0.30, 3.00) | 0.9399 |  | 0.99 (0.32, 3.11) | 0.9867 |  | 0.89 (-0.25-2.02) | 0.8351 |
| 504 | Other alveolar and parietoalveolar pneumonopathy | respiratory | 219/243,046 | 1.01 (0.70, 1.47) | 0.9406 |  | 1.02 (0.71, 1.48) | 0.9047 |  | 1.01 (0.64-1.38) | 0.9520 |
| 420.2 | Pericarditis | circulatory system | 110/243,466 | 0.98 (0.55, 1.74) | 0.9416 |  | 0.97 (0.54, 1.73) | 0.9168 |  | 0.99 (0.41-1.57) | 0.9819 |
| 199.4 | Neurofibromatosis | neoplasms | 56/243,065 | 1.04 (0.29, 3.71) | 0.9475 |  | 1.06 (0.30, 3.78) | 0.9271 |  | 1.07 (-0.20-2.34) | 0.9169 |
| 415 | Pulmonary heart disease | circulatory system | 1,403/243,236 | 1.00 (0.87, 1.14) | 0.9492 |  | 0.96 (0.83, 1.11) | 0.5792 |  | 0.93 (0.79-1.08) | 0.3340 |
| 440 | Atherosclerosis | circulatory system | 173/244,115 | 0.99 (0.66, 1.48) | 0.9528 |  | 0.96 (0.64, 1.44) | 0.8465 |  | 0.97 (0.56-1.38) | 0.8757 |
| 535.1 | Acute gastritis | digestive | 77/223,512 | 0.98 (0.47, 2.05) | 0.9537 |  | 0.94 (0.45, 1.99) | 0.881 |  | 0.95 (0.20-1.69) | 0.8884 |
| 496.1 | Emphysema | respiratory | 7,181/211,409 | 1.00 (0.93, 1.07) | 0.9554 |  | 0.97 (0.91, 1.05) | 0.4819 |  | 0.89 (0.75-1.02) | 0.0771 |
| 189.21 | Malignant neoplasm of bladder | neoplasms | 182/244,225 | 0.99 (0.66, 1.47) | 0.958 |  | 0.98 (0.65, 1.46) | 0.9061 |  | 0.98 (0.58-1.38) | 0.9286 |
| 789 | Nausea and vomiting | symptoms | 187/244,521 | 0.99 (0.66, 1.48) | 0.9602 |  | 0.97 (0.65, 1.46) | 0.8904 |  | 0.99 (0.58-1.39) | 0.9451 |
| 170.1 | Bone cancer | neoplasms | 166/244,440 | 1.01 (0.64, 1.60) | 0.9667 |  | 1.00 (0.63, 1.60) | 0.9838 |  | 0.99 (0.52-1.46) | 0.9699 |
| 415.2 | Chronic pulmonary heart disease | circulatory system | 1,393/243,236 | 1.00 (0.87, 1.15) | 0.9669 |  | 0.96 (0.84, 1.11) | 0.5959 |  | 0.93 (0.79-1.08) | 0.3475 |
| 353 | Nerve root and plexus disorders | neurological | 58/243,158 | 0.98 (0.33, 2.90) | 0.9699 |  | 0.98 (0.33, 2.91) | 0.975 |  | 0.97 (-0.11-2.06) | 0.9574 |
| 368 | Visual disturbances | sense organs | 113/244,587 | 0.99 (0.44, 2.21) | 0.9722 |  | 0.99 (0.44, 2.22) | 0.9762 |  | 0.99 (0.18-1.80) | 0.9869 |
| 593 | Hematuria | genitourinary | 321/230,026 | 0.99 (0.67, 1.46) | 0.9724 |  | 0.99 (0.67, 1.46) | 0.9629 |  | 0.97 (0.58-1.36) | 0.8758 |
| 365.2 | Primary angle-closure glaucoma | sense organs | 329/242,577 | 1.00 (0.74, 1.37) | 0.9755 |  | 1.00 (0.73, 1.36) | 0.9956 |  | 1.01 (0.70-1.32) | 0.9346 |
| 575.2 | Obstruction of bile duct | digestive | 178/210,050 | 0.99 (0.64, 1.53) | 0.9763 |  | 0.99 (0.64, 1.53) | 0.9756 |  | 1.00 (0.57-1.44) | 0.9840 |
| 201 | Hodgkin's disease | neoplasms | 33/243,740 | 0.99 (0.37, 2.62) | 0.9778 |  | 0.96 (0.36, 2.57) | 0.9427 |  | 0.96 (-0.03-1.94) | 0.9285 |
| 158 | Neoplasm of unspecified nature of digestive system | neoplasms | 52/237,409 | 1.01 (0.42, 2.41) | 0.9786 |  | 1.03 (0.43, 2.46) | 0.9504 |  | 1.06 (0.19-1.93) | 0.8955 |
| 420.3 | Endocarditis | circulatory system | 344/243,466 | 1.00 (0.74, 1.35) | 0.979 |  | 0.97 (0.72, 1.31) | 0.842 |  | 0.98 (0.67-1.28) | 0.8717 |
| 733.6 | Costochondritis | musculoskeletal | 44/244,643 | 1.01 (0.39, 2.60) | 0.9803 |  | 0.94 (0.36, 2.42) | 0.8963 |  | 0.94 (0.00-1.88) | 0.8987 |
| 386.1 | Meniere's disease | sense organs | 173/233,256 | 0.99 (0.61, 1.63) | 0.9838 |  | 0.99 (0.60, 1.63) | 0.9741 |  | 1.01 (0.51-1.51) | 0.9680 |
| 155 | Cancer of liver and intrahepatic bile duct | neoplasms | 1,146/237,409 | 1.00 (0.85, 1.18) | 0.9876 |  | 0.98 (0.83, 1.16) | 0.8237 |  | 0.98 (0.81-1.14) | 0.7891 |
| 474.1 | Acute tonsillitis | respiratory | 1,155/239,202 | 1.00 (0.80, 1.25) | 0.9882 |  | 1.00 (0.80, 1.26) | 0.9869 |  | 1.00 (0.78-1.23) | 0.9790 |
| 574.2 | Calculus of bile duct | digestive | 257/210,050 | 1.00 (0.67, 1.47) | 0.9898 |  | 1.01 (0.68, 1.49) | 0.9791 |  | 1.02 (0.63-1.41) | 0.9307 |
| 290.1 | Dementias | mental disorders | 244/244,423 | 1.00 (0.74, 1.36) | 0.9919 |  | 0.98 (0.72, 1.34) | 0.922 |  | 0.98 (0.67-1.28) | 0.8751 |
| 170 | Cancer of bone and connective tissue | neoplasms | 268/244,440 | 1.00 (0.68, 1.47) | 0.995 |  | 1.00 (0.68, 1.47) | 0.9989 |  | 0.99 (0.60-1.37) | 0.9534 |

The phenome-wide *P* value was Bonferroni corrected (0.05 divided by the number of diseases in the group), and *P*<9.92*10^-5^ (0.05/504) for men or 9.33*10^-5^ (0.05/536) for women was considered to be statistically significant.

**Model 1:** adjusted for age, study area, highest education, household income, and household size.

**Model 2:** adjusted for covariates in model 1, alcohol drinking, smoking, dietary habits, physical activity, and BMI.

**Model 3:** adjusted for covariates in model 2, history of diabetes, hypertension, respiratory disease, CVD, or cancer at baseline, family history of the analyzed disease (adjusted for only in corresponding analysis), self-reported satisfaction level of life, and menopausal status.

**Table S8 Incident cases and person-years during the follow-up**

| **Diseases** | **Men** | | |  | **Women** | | |
| --- | --- | --- | --- | --- | --- | --- | --- |
|  | **Cases** | **Person-years** | **Cases/1,000 person-years** |  | **Cases** | **Person-years** | **Cases/1,000 person-years** |
| **Mental and behavioural disorders** |  |  |  |  |  |  |  |
| With a spouse | 1,814 | 2,071,108 | 0.9 |  | 3,967 | 2,933,701.1 | 1.4 |
| Without a spouse | 208 | 143,236.6 | 1.5 |  | 496 | 344,056.5 | 1.4 |
| **Schizophrenia, schizotypal and delusional disorders** |  |  |  |  |  |  |  |
| With a spouse | 263 | 2,080,384.1 | 0.1 |  | 451 | 2,955,681 | 0.2 |
| Without a spouse | 54 | 143,962.9 | 0.4 |  | 67 | 346,500.2 | 0.2 |
| **Mood [affective] disorders** |  |  |  |  |  |  |  |
| With a spouse | 241 | 2,080,676.3 | 0.1 |  | 563 | 2,955,356.9 | 0.2 |
| Without a spouse | 21 | 144,109.8 | 0.1 |  | 68 | 346,497.5 | 0.2 |
| **Neurotic, stress-related and somatoform disorders** |  |  |  |  |  |  |  |
| With a spouse | 945 | 2,075,688.4 | 0.5 |  | 2711 | 2,941,072.6 | 0.9 |
| Without a spouse | 76 | 143,803.2 | 0.5 |  | 274 | 345,203.9 | 0.8 |
| **CVD** |  |  |  |  |  |  |  |
| With a spouse | 43,552 | 1,821,213.7 | 23.9 |  | 56,233 | 2,619,278.9 | 21.5 |
| Without a spouse | 4,585 | 123,892 | 37 |  | 10,610 | 279,684.9 | 37.9 |
| **IH** |  |  |  |  |  |  |  |
| With a spouse | 4,601 | 2,035,702.5 | 2.3 |  | 3,844 | 2,930,889.5 | 1.3 |
| Without a spouse | 757 | 141,657.3 | 5.3 |  | 1,048 | 339,450.2 | 3.1 |
| **Cerebrovascular disease** |  |  |  |  |  |  |  |
| With a spouse | 29,531 | 1,930,560.7 | 15.3 |  | 38,912 | 2,781,983.4 | 14 |
| Without a spouse | 3,159 | 132,700.9 | 23.8 |  | 7,668 | 311,473.1 | 24.6 |
| **IS** |  |  |  |  |  |  |  |
| With a spouse | 18,285 | 1,972,791.7 | 9.3 |  | 21,145 | 2,854,976.1 | 7.4 |
| Without a spouse | 1,843 | 136,092.1 | 13.5 |  | 4,751 | 322,046.7 | 14.8 |
| **IHD** |  |  |  |  |  |  |  |
| With a spouse | 16,726 | 1,973,319.1 | 8.5 |  | 21,631 | 2,802,398.8 | 7.7 |
| Without a spouse | 1,745 | 136,604 | 12.8 |  | 4,584 | 311,673.5 | 14.7 |
| **Cancer** |  |  |  |  |  |  |  |
| With a spouse | 13,438 | 2,050,657 | 6.6 |  | 12,846 | 2,917,931 | 4.4 |
| Without a spouse | 1,372 | 143,103.6 | 9.6 |  | 2,297 | 341,179.9 | 6.7 |
| **Gastric cancer** |  |  |  |  |  |  |  |
| With a spouse | 2,207 | 2,080,099.1 | 1.1 |  | 1,022 | 2,968,426.6 | 0.3 |
| Without a spouse | 252 | 145,267.3 | 1.7 |  | 235 | 348,295.8 | 0.7 |
| **Lung cancer** |  |  |  |  |  |  |  |
| With a spouse | 3,514 | 2,080,814.6 | 1.7 |  | 2,162 | 2,967,420.3 | 0.7 |
| Without a spouse | 352 | 145,361.2 | 2.4 |  | 470 | 348,245 | 1.3 |
| **Liver cancer** |  |  |  |  |  |  |  |
| With a spouse | 1,897 | 2,084,128.5 | 0.9 |  | 927 | 2,970,230.5 | 0.3 |
| Without a spouse | 179 | 145,626.9 | 1.2 |  | 204 | 348,620.6 | 0.6 |
| **Breast cancer** |  |  |  |  |  |  |  |
| With a spouse | NA | NA | NA |  | 2,298 | 2,956,412.7 | 0.8 |
| Without a spouse | NA | NA | NA |  | 263 | 347,098 | 0.8 |
| **Diseases of the respiratory system** |  |  |  |  |  |  |  |
| With a spouse | 35,180 | 1,679,557.8 | 20.9 |  | 51,469 | 2,445,777 | 21 |
| Without a spouse | 2,654 | 110,794.5 | 24 |  | 6,101 | 278,944.9 | 21.9 |
| **COPD** |  |  |  |  |  |  |  |
| With a spouse | 5,553 | 1,903,786.8 | 2.9 |  | 5,324 | 2,782,360 | 1.9 |
| Without a spouse | 799 | 124,706.9 | 6.4 |  | 1,287 | 312,486.1 | 4.1 |
| **Bronchitis** |  |  |  |  |  |  |  |
| With a spouse | 9,438 | 1,980,485 | 4.8 |  | 14,741 | 2,830,856.6 | 5.2 |
| Without a spouse | 986 | 136,493.5 | 7.2 |  | 1,999 | 328,575.5 | 6.1 |
| **Pneumonia** |  |  |  |  |  |  |  |
| With a spouse | 12,035 | 2,045,103.3 | 5.9 |  | 14,668 | 2,917,138 | 5 |
| Without a spouse | 1,190 | 141,992.6 | 8.4 |  | 2,662 | 339,788.7 | 7.8 |
| **Acute upper respiratory infection** |  |  |  |  |  |  |  |
| With a spouse | 17,196 | 1,950,775.1 | 8.8 |  | 26,240 | 2,757,244.1 | 9.5 |
| Without a spouse | 1,177 | 137,228.2 | 8.6 |  | 2,376 | 330,845.9 | 7.2 |
| **Diseases of the digestive system** |  |  |  |  |  |  |  |
| With a spouse | 29,819 | 1,710,470.7 | 17.4 |  | 42,674 | 2,416,760.5 | 17.7 |
| Without a spouse | 2,230 | 122,664.8 | 18.2 |  | 4,877 | 282,558.9 | 17.3 |
| **Gastritis and duodenitis** |  |  |  |  |  |  |  |
| With a spouse | 10,312 | 1,911,979.7 | 5.4 |  | 18,038 | 2,777,268.7 | 6.5 |
| Without a spouse | 839 | 134,821.7 | 6.2 |  | 2,089 | 326,935.5 | 6.4 |
| **Cholelithiasis and cholecystitis** |  |  |  |  |  |  |  |
| With a spouse | 4,551 | 1,981,432.4 | 2.3 |  | 10,438 | 2,697,785.3 | 3.9 |
| Without a spouse | 322 | 139,703.7 | 2.3 |  | 1,125 | 312,783 | 3.6 |
| **Diseases of the genitourinary system** |  |  |  |  |  |  |  |
| With a spouse | 16,596 | 2,004,073.3 | 8.3 |  | 38,044 | 2,733,895.7 | 13.9 |
| Without a spouse | 1,313 | 139,847.1 | 9.4 |  | 2,756 | 333,612.5 | 8.3 |
| **Urethritis and urethral syndrome** |  |  |  |  |  |  |  |
| With a spouse | 1,289 | 2,075,979.5 | 0.6 |  | 4,119 | 2,935,922.2 | 1.4 |
| Without a spouse | 100 | 145,014.4 | 0.7 |  | 322 | 346,099.2 | 0.9 |
| **Renal failure** |  |  |  |  |  |  |  |
| With a spouse | 1,301 | 2,056,662.5 | 0.6 |  | 1,166 | 2,920,393.7 | 0.4 |
| Without a spouse | 129 | 143,904.9 | 0.9 |  | 260 | 342,006.1 | 0.8 |
| **CKD** |  |  |  |  |  |  |  |
| With a spouse | 1,061 | 2,057,086.6 | 0.5 |  | 1,226 | 2,919,333.9 | 0.4 |
| Without a spouse | 97 | 143,956.6 | 0.7 |  | 215 | 342,060.2 | 0.6 |
| **T2D** |  |  |  |  |  |  |  |
| With a spouse | 3,957 | 1,962,456.4 | 2 |  | 6,120 | 2,784,918.8 | 2.2 |
| Without a spouse | 265 | 137,637.8 | 1.9 |  | 797 | 314,470.2 | 2.5 |
| **Hypertension** |  |  |  |  |  |  |  |
| With a spouse | 5,228 | 1,310,930.9 | 4 |  | 8,408 | 1,992,568.7 | 4.2 |
| Without a spouse | 354 | 82,176.2 | 4.3 |  | 1,210 | 183,999.2 | 6.6 |
| **Dorsalgia** |  |  |  |  |  |  |  |
| With a spouse | 5,863 | 2,044,954 | 2.9 |  | 8,033 | 2,911,656.2 | 2.8 |
| Without a spouse | 361 | 143,322.6 | 2.5 |  | 731 | 343,826.4 | 2.1 |
| **Injury & poisoning** |  |  |  |  |  |  |  |
| With a spouse | 13,212 | 1978166.5 | 6.7 |  | 18,227 | 2853985.1 | 6.4 |
| Without a spouse | 939 | 138085.4 | 6.8 |  | 2,532 | 333858.7 | 7.6 |

**Abbreviations:** CVD, cardiovascular disease; IH, intracerebral hemorrhage; IHD, ischemic heart disease; IS, ischemic stroke; COPD, chronic obstructive pulmonary disease; CKD, chronic kidney disease; T2D, type 2 diabetes.

**Table S9 Adjusted hazard ratios of marital status (lived without vs. with a spouse) with 30 disease categories stratified by sex**

| **Disease categories** | **Men** | |  | **Women** | | ***P* for interaction*** |
| --- | --- | --- | --- | --- | --- | --- |
|  | **HR (95% CI)** | ***P*** |  | **HR (95% CI)** | ***P*** |  |
| **Mental and behavioural disorders** |  |  |  |  |  | < 0.0001 |
| Model 1 | 1.36 (1.17-1.58) | < 0.0001 |  | 1.03 (0.93-1.13) | 0.6083 |  |
| Model 2 | 1.36 (1.17-1.58) | < 0.0001 |  | 1.02 (0.92-1.13) | 0.6998 |  |
| Model 3 | 1.31 (1.13-1.53) | 0.0004 |  | 1.01 (0.92-1.12) | 0.7761 |  |
| **Schizophrenia, schizotypal and delusional disorders** |  |  |  |  |  | 0.0028 |
| Model 1 | 2.68 (1.94-3.72) | < 0.0001 |  | 1.58 (1.19-2.08) | 0.0014 |  |
| Model 2 | 2.71 (1.95-3.77) | < 0.0001 |  | 1.56 (1.18-2.07) | 0.0017 |  |
| Model 3 | 2.55 (1.83-3.56) | < 0.0001 |  | 1.49 (1.13-1.97) | 0.0051 |  |
| **Mood [affective] disorders** |  |  |  |  |  | 0.3481 |
| Model 1 | 1.43 (0.89-2.28) | 0.1375 |  | 1.10 (0.84-1.44) | 0.4839 |  |
| Model 2 | 1.42 (0.89-2.26) | 0.1446 |  | 1.09 (0.84-1.43) | 0.5184 |  |
| Model 3 | 1.37 (0.85-2.19) | 0.1945 |  | 1.08 (0.83-1.41) | 0.5741 |  |
| **Neurotic, stress-related and somatoform disorders** |  |  |  |  |  | 0.3991 |
| Model 1 | 1.00 (0.79-1.28) | 0.9757 |  | 0.97 (0.85-1.11) | 0.6307 |  |
| Model 2 | 0.99 (0.77-1.26) | 0.9054 |  | 0.96 (0.84-1.10) | 0.5545 |  |
| Model 3 | 0.94 (0.74-1.20) | 0.6228 |  | 0.95 (0.83-1.09) | 0.4679 |  |
| **CVD** |  |  |  |  |  | < 0.0001 |
| Model 1 | 1.08 (1.05-1.12) | < 0.0001 |  | 0.99 (0.96-1.01) | 0.2372 |  |
| Model 2 | 1.10 (1.07-1.13) | < 0.0001 |  | 0.98 (0.96-1.01) | 0.1331 |  |
| Model 3 | 1.07 (1.04-1.10) | < 0.0001 |  | 0.98 (0.96-1.00) | 0.0528 |  |
| **IH** |  |  |  |  |  | 0.0012 |
| Model 1 | 1.32 (1.22-1.43) | < 0.0001 |  | 1.09 (1.02-1.18) | 0.0190 |  |
| Model 2 | 1.34 (1.23-1.45) | < 0.0001 |  | 1.08 (1.00-1.17) | 0.0391 |  |
| Model 3 | 1.26 (1.16-1.36) | < 0.0001 |  | 1.07 (0.99-1.15) | 0.0939 |  |
| **Cerebrovascular disease** |  |  |  |  |  | < 0.0001 |
| Model 1 | 1.09 (1.05-1.13) | < 0.0001 |  | 0.98 (0.96-1.01) | 0.2289 |  |
| Model 2 | 1.10 (1.06-1.15) | < 0.0001 |  | 0.98 (0.95-1.01) | 0.1498 |  |
| Model 3 | 1.07 (1.03-1.12) | 0.0002 |  | 0.98 (0.95-1.00) | 0.0630 |  |
| **IS** |  |  |  |  |  | < 0.0001 |
| Model 1 | 1.05 (1.00-1.10) | 0.0575 |  | 0.98 (0.94-1.01) | 0.1705 |  |
| Model 2 | 1.07 (1.02-1.12) | 0.0091 |  | 0.97 (0.94-1.00) | 0.0762 |  |
| Model 3 | 1.04 (0.99-1.10) | 0.1006 |  | 0.96 (0.93-1.00) | 0.0388 |  |
| **IHD** |  |  |  |  |  | < 0.0001 |
| Model 1 | 1.05 (1.00-1.11) | 0.0708 |  | 0.96 (0.93-0.99) | 0.0167 |  |
| Model 2 | 1.07 (1.01-1.12) | 0.0136 |  | 0.95 (0.92-0.99) | 0.0071 |  |
| Model 3 | 1.05 (0.99-1.10) | 0.0779 |  | 0.95 (0.92-0.98) | 0.0048 |  |
| **Cancer** |  |  |  |  |  | < 0.0001 |
| Model 1 | 1.06 (1.00-1.13) | 0.0337 |  | 0.99 (0.94-1.04) | 0.6328 |  |
| Model 2 | 1.06 (1.00-1.12) | 0.0452 |  | 0.98 (0.93-1.03) | 0.4312 |  |
| Model 3 | 1.06 (1.00-1.12) | 0.0432 |  | 0.98 (0.94-1.03) | 0.4484 |  |
| **Gastric cancer** |  |  |  |  |  | 0.1464 |
| Model 1 | 1.14 (1.00-1.31) | 0.0546 |  | 1.02 (0.88-1.20) | 0.7626 |  |
| Model 2 | 1.14 (0.99-1.30) | 0.0647 |  | 1.02 (0.87-1.19) | 0.8191 |  |
| Model 3 | 1.14 (1.00-1.30) | 0.0589 |  | 1.02 (0.87-1.19) | 0.8287 |  |
| **Lung cancer** |  |  |  |  |  | 0.0123 |
| Model 1 | 1.05 (0.94-1.18) | 0.3677 |  | 0.94 (0.84-1.05) | 0.2442 |  |
| Model 2 | 1.04 (0.93-1.16) | 0.5068 |  | 0.91 (0.82-1.02) | 0.1054 |  |
| Model 3 | 1.04 (0.93-1.17) | 0.4793 |  | 0.92 (0.82-1.02) | 0.1225 |  |
| **Liver cancer** |  |  |  |  |  | 0.4809 |
| Model 1 | 1.03 (0.88-1.21) | 0.6863 |  | 1.02 (0.87-1.20) | 0.8015 |  |
| Model 2 | 1.03 (0.88-1.20) | 0.7440 |  | 1.01 (0.85-1.19) | 0.9402 |  |
| Model 3 | 1.03 (0.88-1.21) | 0.7250 |  | 1.00 (0.85-1.18) | 0.9859 |  |
| **Breast cancer** |  |  |  |  |  | 0.1585 |
| Model 1 | NA | NA |  | 0.93 (0.81-1.07) | 0.3058 |  |
| Model 2 | NA | NA |  | 0.94 (0.82-1.07) | 0.3487 |  |
| Model 3 | NA | NA |  | 0.94 (0.82-1.08) | 0.4184 |  |
| **Diseases of the respiratory system** |  |  |  |  |  | 0.0060 |
| Model 1 | 0.95 (0.92-0.99) | 0.0254 |  | 0.95 (0.92-0.98) | 0.0005 |  |
| Model 2 | 0.95 (0.91-0.99) | 0.0174 |  | 0.95 (0.92-0.98) | 0.0003 |  |
| Model 3 | 0.95 (0.91-0.99) | 0.0179 |  | 0.94 (0.92-0.97) | 0.0001 |  |
| **COPD** |  |  |  |  |  | < 0.0001 |
| Model 1 | 1.22 (1.13-1.32) | < 0.0001 |  | 0.99 (0.92-1.05) | 0.6718 |  |
| Model 2 | 1.20 (1.11-1.30) | < 0.0001 |  | 0.97 (0.91-1.04) | 0.4266 |  |
| Model 3 | 1.20 (1.11-1.30) | < 0.0001 |  | 0.97 (0.91-1.04) | 0.3831 |  |
| **Bronchitis** |  |  |  |  |  | < 0.0001 |
| Model 1 | 1.07 (1.00-1.14) | 0.0604 |  | 0.92 (0.87-0.96) | 0.0007 |  |
| Model 2 | 1.06 (0.99-1.13) | 0.1199 |  | 0.91 (0.87-0.96) | 0.0004 |  |
| Model 3 | 1.04 (0.97-1.11) | 0.2729 |  | 0.90 (0.86-0.95) | 0.0001 |  |
| **Pneumonia** |  |  |  |  |  | < 0.0001 |
| Model 1 | 1.03 (0.97-1.09) | 0.3704 |  | 0.91 (0.87-0.95) | < 0.0001 |  |
| Model 2 | 1.02 (0.96-1.08) | 0.5266 |  | 0.90 (0.86-0.94) | < 0.0001 |  |
| Model 3 | 1.02 (0.96-1.08) | 0.5844 |  | 0.90 (0.86-0.94) | < 0.0001 |  |
| **Acute upper respiratory infection** |  |  |  |  |  | 0.2893 |
| Model 1 | 0.95 (0.89-1.01) | 0.0972 |  | 0.92 (0.88-0.96) | 0.0002 |  |
| Model 2 | 0.94 (0.89-1.00) | 0.0711 |  | 0.92 (0.88-0.96) | 0.0002 |  |
| Model 3 | 0.94 (0.89-1.00) | 0.0614 |  | 0.91 (0.87-0.95) | 0.0001 |  |
| **Diseases of the digestive system** |  |  |  |  |  | 0.0397 |
| Model 1 | 0.97 (0.93-1.02) | 0.2261 |  | 0.93 (0.90-0.96) | < 0.0001 |  |
| Model 2 | 0.97 (0.93-1.02) | 0.2228 |  | 0.93 (0.90-0.96) | < 0.0001 |  |
| Model 3 | 0.97 (0.93-1.02) | 0.2019 |  | 0.93 (0.90-0.96) | < 0.0001 |  |
| **Gastritis and duodenitis** |  |  |  |  |  | 0.1508 |
| Model 1 | 1.03 (0.95-1.10) | 0.4804 |  | 0.89 (0.85-0.94) | < 0.0001 |  |
| Model 2 | 1.01 (0.94-1.09) | 0.7566 |  | 0.89 (0.85-0.93) | < 0.0001 |  |
| Model 3 | 1.01 (0.94-1.09) | 0.8220 |  | 0.88 (0.84-0.93) | < 0.0001 |  |
| **Cholelithiasis and cholecystitis** |  |  |  |  |  | 0.3839 |
| Model 1 | 0.92 (0.81-1.03) | 0.1386 |  | 0.91 (0.850.97) | 0.0041 |  |
| Model 2 | 0.94 (0.84-1.06) | 0.2956 |  | 0.91 (0.85-0.97) | 0.0057 |  |
| Model 3 | 0.94 (0.83-1.05) | 0.2676 |  | 0.91 (0.85-0.97) | 0.0039 |  |
| **Diseases of the genitourinary system** |  |  |  |  |  | < 0.0001 |
| Model 1 | 0.96 (0.91-1.02) | 0.1911 |  | 0.88 (0.85-0.92) | < 0.0001 |  |
| Model 2 | 0.97 (0.91-1.03) | 0.2799 |  | 0.88 (0.85-0.92) | < 0.0001 |  |
| Model 3 | 0.97 (0.91-1.03) | 0.2771 |  | 0.89 (0.85-0.93) | < 0.0001 |  |
| **Urethritis and urethral syndrome** |  |  |  |  |  | 0.0222 |
| Model 1 | 1.13 (0.91-1.40) | 0.2572 |  | 0.85 (0.75-0.96) | 0.0088 |  |
| Model 2 | 1.13 (0.91-1.40) | 0.2655 |  | 0.85 (0.75-0.96) | 0.0105 |  |
| Model 3 | 1.14 (0.92-1.42) | 0.2160 |  | 0.86 (0.76-0.97) | 0.0127 |  |
| **Renal failure** |  |  |  |  |  | 0.3410 |
| Model 1 | 1.07 (0.89-1.29) | 0.4864 |  | 0.97 (0.84-1.12) | 0.6654 |  |
| Model 2 | 1.09 (0.91-1.32) | 0.3563 |  | 0.95 (0.82-1.10) | 0.5005 |  |
| Model 3 | 1.07 (0.89-1.30) | 0.4603 |  | 0.95 (0.82-1.10) | 0.4839 |  |
| **CKD** |  |  |  |  |  | 0.2878 |
| Model 1 | 1.00 (0.81-1.25) | 0.9667 |  | 0.97 (0.83-1.14) | 0.6948 |  |
| Model 2 | 1.03 (0.83-1.28) | 0.7709 |  | 0.96 (0.82-1.12) | 0.6004 |  |
| Model 3 | 1.00 (0.81-1.24) | 0.9856 |  | 0.95 (0.81-1.12) | 0.5389 |  |
| **T2D** |  |  |  |  |  | 0.8018 |
| Model 1 | 0.96 (0.85-1.10) | 0.5656 |  | 0.94 (0.87-1.01) | 0.1083 |  |
| Model 2 | 1.05 (0.92-1.19) | 0.4748 |  | 0.95 (0.88-1.03) | 0.2186 |  |
| Model 3 | 1.03 (0.91-1.17) | 0.6396 |  | 0.94 (0.87-1.02) | 0.1289 |  |
| **Hypertension** |  |  |  |  |  | 0.9863 |
| Model 1 | 0.93 (0.83-1.03) | 0.1739 |  | 0.99 (0.93-1.06) | 0.7507 |  |
| Model 2 | 0.95 (0.85-1.07) | 0.3983 |  | 0.99 (0.93-1.06) | 0.8759 |  |
| Model 3 | 0.96 (0.86-1.07) | 0.4455 |  | 0.99 (0.93-1.06) | 0.8027 |  |
| **Dorsalgia** |  |  |  |  |  | 0.0308 |
| Model 1 | 0.85 (0.77-0.95) | 0.0051 |  | 0.91 (0.84-0.99) | 0.0204 |  |
| Model 2 | 0.86 (0.77-0.96) | 0.0070 |  | 0.91 (0.84-0.99) | 0.0245 |  |
| Model 3 | 0.85 (0.76-0.96) | 0.0056 |  | 0.90 (0.83-0.98) | 0.0147 |  |
| **Injury & poisoning** |  |  |  |  |  | 0.0012 |
| Model 1 | 0.96 (0.90-1.03) | 0.2474 |  | 0.93 (0.88-0.97) | 0.0010 |  |
| Model 2 | 0.96 (0.90-1.03) | 0.2357 |  | 0.93 (0.89-0.97) | 0.0014 |  |
| Model 3 | 0.96 (0.90-1.03) | 0.2743 |  | 0.93 (0.88-0.97) | 0.0010 |  |

**Abbreviations:** CVD, cardiovascular disease; IH, intracerebral hemorrhage; IHD, ischemic heart disease; IS, ischemic stroke; COPD, chronic obstructive pulmonary disease; CKD, chronic kidney disease; T2D, type 2 diabetes.

**Model 1:** adjusted for age, study area, highest education, household income, and household size.

**Model 2:** adjusted for covariates in model 1, alcohol drinking, smoking, dietary habits, physical activity, and BMI.

**Model 3:** adjusted for covariates in model 2, history of diabetes, hypertension, respiratory disease, CVD, or cancer at baseline, self-reported satisfaction level of life, and family history of the analyzed disease (adjusted for only in corresponding analysis), self-reported satisfaction level of life, and menopausal status (for women only).

* *P* values for interaction in model 3.

**Table S10 Adjusted hazard ratios (aHRs) of marital status (lived without vs. with a spouse) with 30 disease categories stratified by sex and areas**

| **Disease categories** | **Urban men** | |  | **Rural men** | | ***P* for interaction*** |  | **Urban women** | |  | **Rural women** | | ***P* for interaction*** |
| --- | --- | --- | --- | --- | --- | --- | --- | --- | --- | --- | --- | --- | --- |
|  | **HR (95% CI)** | ***P*** |  | **HR (95% CI)** | ***P*** |  |  | **HR (95% CI)** | ***P*** |  | **HR (95% CI)** | ***P*** |  |
| **Mental and behavioural disorders** |  |  |  |  |  | 0.1589 |  |  |  |  |  |  | 0.0055 |
| Model 1 | 1.34 (1.00-1.78) | 0.0463 |  | 1.38 (1.15-1.65) | 0.0004 |  |  | 1.06 (0.90-1.24) | 0.4831 |  | 0.99 (0.86-1.13) | 0.8334 |  |
| Model 2 | 1.33 (1.00-1.77) | 0.0508 |  | 1.37 (1.14-1.63) | 0.0006 |  |  | 1.06 (0.90-1.24) | 0.4998 |  | 0.98 (0.86-1.12) | 0.7510 |  |
| Model 3 | 1.32 (0.99-1.76) | 0.0564 |  | 1.30 (1.09-1.56) | 0.0041 |  |  | 1.06 (0.90-1.24) | 0.4888 |  | 0.97 (0.85-1.11) | 0.6403 |  |
| **Schizophrenia, schizotypal and delusional disorders** |  |  |  |  |  | 0.3270 |  |  |  |  |  |  | 0.0341 |
| Model 1 | 2.68 (1.55-4.66) | 0.0004 |  | 2.68 (1.78-4.03) | < 0.0001 |  |  | 2.11 (1.40-3.19) | 0.0004 |  | 1.17 (0.78-1.72) | 0.4568 |  |
| Model 2 | 2.74 (1.58-4.76) | 0.0003 |  | 2.71 (1.80-4.08) | < 0.0001 |  |  | 2.11 (1.39-3.18) | 0.0004 |  | 1.15 (0.76-1.74) | 0.5051 |  |
| Model 3 | 2.63 (1.50-4.61) | 0.0008 |  | 2.50 (1.65-3.78) | < 0.0001 |  |  | 2.07 (1.37-3.14) | 0.0006 |  | 1.09 (0.72-1.64) | 0.6941 |  |
| **Mood [affective] disorders** |  |  |  |  |  | 0.8290 |  |  |  |  |  |  | 0.2451 |
| Model 1 | 1.18 (0.50-2.80) | 0.7083 |  | 1.60 (0.91-2.80) | 0.0992 |  |  | 1.21 (0.83-1.75) | 0.3265 |  | 1.02 (0.68-1.52) | 0.9338 |  |
| Model 2 | 1.15 (0.48-2.73) | 0.7525 |  | 1.57 (0.70-2.75) | 0.1147 |  |  | 1.20 (0.83-1.74) | 0.3387 |  | 1.00 (0.67-1.50) | 0.9863 |  |
| Model 3 | 1.14 (0.48-2.71) | 0.7656 |  | 1.44 (0.81-2.53) | 0.2115 |  |  | 1.21 (0.83-1.77) | 0.3103 |  | 0.97 (0.65-1.45) | 0.8858 |  |
| **Neurotic, stress-related and somatoform disorders** |  |  |  |  |  | 0.0865 |  |  |  |  |  |  | 0.0686 |
| Model 1 | 1.19 (0.67-2.13) | 0.5561 |  | 0.97 (0.74-1.27) | 0.9500 |  |  | 1.00 (0.77-1.29) | 0.9952 |  | 0.95 (0.81-1.11) | 0.5130 |  |
| Model 2 | 1.17 (0.65-2.09) | 0.5988 |  | 0.95 (0.73-1.25) | 0.9400 |  |  | 1.00 (0.77-1.29) | 0.9866 |  | 0.94 (0.80-1.10) | 0.4496 |  |
| Model 3 | 1.17 (0.65-2.09) | 0.6016 |  | 0.89 (0.68-1.17) | 0.9300 |  |  | 0.99 (0.77-1.28) | 0.9484 |  | 0.93 (0.79-1.09) | 0.3677 |  |
| **CVD** |  |  |  |  |  | 0.6527 |  |  |  |  |  |  | < 0.0001 |
| Model 1 | 1.07 (1.01-1.14) | 0.0150 |  | 1.09 (1.05-1.13) | < 0.0001 |  |  | 1.00 (0.96-1.03) | 0.8184 |  | 0.96 (0.93-0.99) | 0.0166 |  |
| Model 2 | 1.08 (1.02-1.14) | 0.0081 |  | 1.10 (1.06-1.15) | < 0.0001 |  |  | 0.99 (0.96-1.02) | 0.5529 |  | 0.96 (0.93-0.99) | 0.0117 |  |
| Model 3 | 1.06 (1.00-1.13) | 0.0336 |  | 1.07 (1.03-1.11) | 0.0013 |  |  | 0.99 (0.96-1.02) | 0.4396 |  | 0.95 (0.92-0.98) | 0.0017 |  |
| **IH** |  |  |  |  |  | 0.0168 |  |  |  |  |  |  | 0.0009 |
| Model 1 | 1.13 (0.93-1.37) | 0.2283 |  | 1.37 (1.26-1.51) | < 0.0001 |  |  | 1.01 (0.87-1.16) | 0.9444 |  | 1.13 (1.03-1.24) | 0.0082 |  |
| Model 2 | 1.14 (0.94-1.38) | 0.1994 |  | 1.39 (1.28-1.53) | < 0.0001 |  |  | 0.99 (0.86-1.14) | 0.9112 |  | 1.12 (1.02-1.23) | 0.0151 |  |
| Model 3 | 1.10 (0.91-1.34) | 0.3212 |  | 1.30 (1.19-1.42) | < 0.0001 |  |  | 0.98 (0.85-1.13) | 0.7821 |  | 1.10 (1.00-1.20) | 0.0408 |  |
| **Cerebrovascular disease** |  |  |  |  |  | 0.2592 |  |  |  |  |  |  | < 0.0001 |
| Model 1 | 1.08 (1.01-1.16) | 0.0168 |  | 1.08 (1.03-1.14) | 0.0007 |  |  | 1.00 (0.96-1.04) | 0.9257 |  | 0.94 (0.90-0.98) | 0.0013 |  |
| Model 2 | 1.09 (1.02-1.17) | 0.0086 |  | 1.10 (1.05-1.16) | < 0.0001 |  |  | 0.99 (0.96-1.03) | 0.6522 |  | 0.94 (0.90-0.97) | 0.0012 |  |
| Model 3 | 1.08 (1.01-1.16) | 0.0192 |  | 1.06 (1.01-1.11) | 0.0116 |  |  | 0.99 (0.95-1.03) | 0.5293 |  | 0.93 (0.89-0.96) | < 0.0001 |  |
| **IS** |  |  |  |  |  | 0.2938 |  |  |  |  |  |  | < 0.0001 |
| Model 1 | 1.08 (1.00-1.17) | 0.0483 |  | 1.03 (0.96-1.09) | 0.4297 |  |  | 1.01 (0.97-1.06) | 0.6041 |  | 0.91 (0.86-0.96 | 0.0007 |  |
| Model 2 | 1.09 (1.01-1.18) | 0.0267 |  | 1.05 (0.99-1.12) | 0.1315 |  |  | 1.00 (0.96-1.05) | 0.9497 |  | 0.90 (0.85-0.96) | 0.0004 |  |
| Model 3 | 1.08 (1.00-1.17) | 0.0534 |  | 1.01 (0.95-1.0) | 0.7019 |  |  | 1.00 (0.95-1.04) | 0.9131 |  | 0.89 (0.84-0.95) | 0.0001 |  |
| **IHD** |  |  |  |  |  | < 0.0001 |  |  |  |  |  |  | 0.8441 |
| Model 1 | 0.97 (0.88-1.06) | 0.4813 |  | 1.09 (1.03-1.16) | 0.0058 |  |  | 0.98 (0.94-1.03) | 0.4813 |  | 0.93 (0.89-0.98) | 0.0074 |  |
| Model 2 | 0.98 (0.89-1.07) | 0.6216 |  | 1.11 (1.04-1.18) | 0.0009 |  |  | 0.98 (0.93-1.03) | 0.3580 |  | 0.93 (0.88-0.98) | 0.0045 |  |
| Model 3 | 0.96 (0.88-1.06) | 0.4460 |  | 1.09 (1.02-1.16) | 0.0097 |  |  | 0.98 (0.93-1.02) | 0.3252 |  | 0.92 (0.87-0.97) | 0.0015 |  |
| **Cancer** |  |  |  |  |  | 0.1833 |  |  |  |  |  |  | 0.4067 |
| Model 1 | 1.01 (0.92-1.12) | 0.8324 |  | 1.10 (1.02-1.18) | 0.0143 |  |  | 1.03 (0.97-1.10) | 0.3757 |  | 0.93 (0.87-1.00) | 0.0606 |  |
| Model 2 | 1.00 (0.91-1.10) | 0.9882 |  | 1.10 (1.02-1.18) | 0.0141 |  |  | 1.02 (0.96-1.09) | 0.5299 |  | 0.92 (0.86-1.00) | 0.0393 |  |
| Model 3 | 1.00 (0.91-1.10) | 0.9889 |  | 1.10 (1.02-1.18) | 0.0123 |  |  | 1.02 (0.96-1.09) | 0.4797 |  | 0.92 (0.85-0.99) | 0.0321 |  |
| **Gastric cancer** |  |  |  |  |  | 0.1317 |  |  |  |  |  |  | 0.1848 |
| Model 1 | 1.07 (0.85-1.35) | 0.5798 |  | 1.18 (1.10-1.39) | 0.0551 |  |  | 1.01 (0.82-1.24) | 0.9482 |  | 1.05 (0.82-1.33) | 0.7113 |  |
| Model 2 | 1.05 (0.83-1.33) | 0.6649 |  | 1.17 (0.99-1.39) | 0.0600 |  |  | 1.00 (0.82-1.23) | 0.9847 |  | 1.04 (0.82-1.31) | 0.7725 |  |
| Model 3 | 1.06 (0.84-1.33) | 0.6423 |  | 1.18 (1.00-1.39) | 0.0559 |  |  | 1.01 (0.82-1.24) | 0.9588 |  | 1.03 (0.81-1.30) | 0.8193 |  |
| **Lung cancer** |  |  |  |  |  | 0.7205 |  |  |  |  |  |  | 0.0405 |
| Model 1 | 0.97 (0.80-1.17) | 0.7226 |  | 1.10 (0.96-1.27) | 0.1725 |  |  | 0.97 (0.84-1.12) | 0.6767 |  | 0.85 (0.72-1.02) | 0.0802 |  |
| Model 2 | 0.93 (0.77-1.13) | 0.4732 |  | 1.10 (0.95-1.27) | 0.1910 |  |  | 0.95 (0.82-1.09) | 0.4439 |  | 0.83 (0.70-1.00) | 0.0452 |  |
| Model 3 | 0.94 (0.78-1.14) | 0.5363 |  | 1.10 (0.95-1.27) | 0.2104 |  |  | 0.95 (0.82-1.10) | 0.4899 |  | 0.83 (0.70-1.00) | 0.0443 |  |
| **Liver cancer** |  |  |  |  |  | 0.8791 |  |  |  |  |  |  | 0.5726 |
| Model 1 | 1.02 (0.78-1.34) | 0.8727 |  | 1.04 (0.86-1.27) | 0.6807 |  |  | 0.96 (0.76-1.23) | 0.7619 |  | 1.09 (0.87-1.37) | 0.4604 |  |
| Model 2 | 1.02 (0.78-1.33) | 0.8898 |  | 1.04 (0.85-1.26) | 0.7305 |  |  | 0.95 (0.75-1.21) | 0.6835 |  | 1.07 (0.85-1.35) | 0.5600 |  |
| Model 3 | 1.02 (0.78-1.34) | 0.8802 |  | 1.03 (0.85-1.26) | 0.7391 |  |  | 0.95 (0.75-1.22) | 0.6974 |  | 1.06 (0.84-1.33) | 0.6263 |  |
| **Breast cancer** |  |  |  |  |  | 0.3776 |  |  |  |  |  |  | 0.0228 |
| Model 1 | NA | NA |  | 0.41 (0.05-3.14) | 0.3918 |  |  | 0.99 (0.84-1.15) | 0.8698 |  | 0.78 (0.57-1.06) | 0.1104 |  |
| Model 2 | NA | NA |  | 0.41 (0.05-3.13) | 0.3890 |  |  | 0.99 (0.85-1.16) | 0.9318 |  | 0.78 (0.57-1.06) | 0.1118 |  |
| Model 3 | NA | NA |  | 0.44 (0.06-3.35) | 0.4257 |  |  | 1.00 (0.85-1.17) | 0.9842 |  | 0.79 (0.58-1.08) | 0.1384 |  |
| **Diseases of the respiratory system** |  |  |  |  |  | < 0.0001 |  |  |  |  |  |  | < 0.0001 |
| Model 1 | 0.86 (0.82-0.90) | <0.0001 |  | 0.96 (0.93-0.99) | 0.0040 |  |  | 0.88 (0.83-0.92) | < 0.0001 |  | 0.96 (0.92-0.99) | 0.0152 |  |
| Model 2 | 0.85 (0.82-0.89) | <0.0001 |  | 0.96 (0.93-0.98) | 0.0023 |  |  | 0.88 (0.83-0.92) | < 0.0001 |  | 0.96 (0.92-0.99) | 0.0170 |  |
| Model 3 | 0.86 (0.83-0.90) | <0.0001 |  | 0.95 (0.92-0.97) | 0.0001 |  |  | 0.87 (0.83-0.92) | < 0.0001 |  | 0.95 (0.92-0.99) | 0.0069 |  |
| **COPD** |  |  |  |  |  | 0.7945 |  |  |  |  |  |  | 0.6967 |
| Model 1 | 1.11 (0.93-1.34) | 0.2533 |  | 1.24 (1.14-1.35) | < 0.0001 |  |  | 0.94 (0.83-1.07) | 0.3288 |  | 1.00 (0.92-1.08) | 0.9919 |  |
| Model 2 | 1.08 (0.90-1.30) | 0.392 |  | 1.23 (1.13-1.33) | < 0.0001 |  |  | 0.92 (0.81-1.04) | 0.1915 |  | 0.99 (0.91-1.07) | 0.7936 |  |
| Model 3 | 1.08 (0.90-1.30) | 0.3954 |  | 1.22 (1.12-1.33) | < 0.0001 |  |  | 0.91 (0.80-1.04) | 0.1551 |  | 0.99 (0.91-1.07) | 0.7528 |  |
| **Bronchitis** |  |  |  |  |  | 0.0062 |  |  |  |  |  |  | 0.0031 |
| Model 1 | 0.98 (0.84-1.15) | 0.8395 |  | 1.08 (1.01-1.17) | 0.0367 |  |  | 0.76 (0.69-0.83) | < 0.0001 |  | 0.95 (0.89-1.01) | 0.1074 |  |
| Model 2 | 0.96 (0.82-1.12) | 0.5787 |  | 1.08 (1.00-1.16) | 0.0583 |  |  | 0.76 (0.69-0.83) | < 0.0001 |  | 0.95 (0.89-1.01) | 0.0924 |  |
| Model 3 | 0.96 (0.82-1.12) | 0.5682 |  | 1.06 (0.98-1.14) | 0.1622 |  |  | 0.76 (0.69-0.83) | < 0.0001 |  | 0.94 (0.88-1.00) | 0.0389 |  |
| **Pneumonia** |  |  |  |  |  | 0.2740 |  |  |  |  |  |  | < 0.0001 |
| Model 1 | 0.98 (0.88-1.10) | 0.7814 |  | 1.07 (0.99-1.15) | 0.2091 |  |  | 0.95 (0.89-1.02) | 0.1602 |  | 0.86 (0.81-0.92) | < 0.0001 |  |
| Model 2 | 0.97 (0.87-1.08) | 0.6061 |  | 1.06 (0.99-1.15) | 0.2790 |  |  | 0.95 (0.89-1.01) | 0.1246 |  | 0.86 (0.80-0.91) | < 0.0001 |  |
| Model 3 | 0.97 (0.87-1.08) | 0.5410 |  | 1.06 (0.98-1.15) | 0.2684 |  |  | 0.95 (0.89-1.01) | 0.1174 |  | 0.85 (0.80-0.91) | < 0.0001 |  |
| **Acute upper respiratory infection** |  |  |  |  |  | < 0.0001 |  |  |  |  |  |  | < 0.0001 |
| Model 1 | 0.77 (0.63-0.95) | 0.0144 |  | 0.97 (0.91-1.04) | 0.3864 |  |  | 0.67 (0.60-0.75) | < 0.0001 |  | 0.95 (0.90-1.00) | 0.0418 |  |
| Model 2 | 0.75 (0.61-0.92) | 0.0052 |  | 0.97 (0.91-1.03) | 0.3565 |  |  | 0.68 (0.61-0.76) | < 0.0001 |  | 0.95 (0.90-1.00) | 0.0422 |  |
| Model 3 | 0.75 (0.61-0.92) | 0.0064 |  | 0.97 (0.91-1.03) | 0.3045 |  |  | 0.68 (0.61-0.76) | < 0.0001 |  | 0.94 (0.89-0.99) | 0.0158 |  |
| **Diseases of the digestive system** |  |  |  |  |  | < 0.0001 |  |  |  |  |  |  | < 0.0001 |
| Model 1 | 1.00 (0.91-1.09) | 0.9593 |  | 0.97 (0.92-1.02) | 0.1666 |  |  | 0.92 (0.87-0.97) | 0.0012 |  | 0.92 (0.88-0.96) | 0.0001 |  |
| Model 2 | 1.00 (0.91-1.09) | 0.9272 |  | 0.97 (0.92-1.02) | 0.1780 |  |  | 0.92 (0.87-0.97) | 0.0012 |  | 0.92 (0.88-0.96) | 0.0001 |  |
| Model 3 | 0.99 (0.91-1.09) | 0.9042 |  | 0.97 (0.92-1.02) | 0.1601 |  |  | 0.92 (0.87-0.97) | 0.0011 |  | 0.91 (0.88-0.95) | < 0.0001 |  |
| **Gastritis and duodenitis** |  |  |  |  |  | 0.0136 |  |  |  |  |  |  | 0.0862 |
| Model 1 | 0.98 (0.80-1.18) | 0.7988 |  | 1.04 (0.96-1.12) | 0.3878 |  |  | 0.80 (0.73-0.88) | < 0.0001 |  | 0.91 (0.86-0.97) | 0.0018 |  |
| Model 2 | 0.96 (0.79-1.16) | 0.6642 |  | 1.02 (0.94-1.11) | 0.6001 |  |  | 0.80 (0.73-0.88) | < 0.0001 |  | 0.91 (0.86-0.96) | 0.0013 |  |
| Model 3 | 0.95 (0.78-1.16) | 0.6301 |  | 1.02 (0.94-1.10) | 0.6716 |  |  | 0.79 (0.72-0.87) | < 0.0001 |  | 0.90 (0.85-0.96) | 0.0006 |  |
| **Cholelithiasis and cholecystitis** |  |  |  |  |  | 0.6374 |  |  |  |  |  |  | 0.4732 |
| Model 1 | 0.85 (0.65-1.09) | 0.1975 |  | 0.93 (0.82-1.07) | 0.3169 |  |  | 0.93 (0.82-1.05) | 0.2450 |  | 0.90 (0.83-0.97) | 0.0061 |  |
| Model 2 | 0.86 (0.67-1.12) | 0.2655 |  | 0.96 (0.84-1.10) | 0.5752 |  |  | 0.93 (0.81-1.05) | 0.2318 |  | 0.90 (0.83-0.97) | 0.0091 |  |
| Model 3 | 0.86 (0.66-1.10) | 0.2306 |  | 0.96 (0.84-1.10) | 0.5883 |  |  | 0.93 (0.82-1.05) | 0.2500 |  | 0.89 (0.82-0.97) | 0.0050 |  |
| **Diseases of the genitourinary system** |  |  |  |  |  | 0.0131 |  |  |  |  |  |  | < 0.0001 |
| Model 1 | 0.96 (0.85-1.08) | 0.4512 |  | 0.96 (0.90-1.03) | 0.2301 |  |  | 0.89 (0.83-0.96) | 0.0021 |  | 0.87 (0.83-0.92) | < 0.0001 |  |
| Model 2 | 0.96 (0.85-1.08) | 0.4875 |  | 0.97 (0.91-1.04) | 0.3509 |  |  | 0.89 (0.83-0.96) | 0.0023 |  | 0.87 (0.83-0.92) | < 0.0001 |  |
| Model 3 | 0.95 (0.85-1.08) | 0.4412 |  | 0.96 (0.90-1.03) | 0.2156 |  |  | 0.89 (0.83-0.96) | 0.0023 |  | 0.88 (0.83-0.93) | < 0.0001 |  |
| **Urethritis and urethral syndrome** |  |  |  |  |  | 0.0735 |  |  |  |  |  |  | 0.0147 |
| Model 1 | 1.33 (0.65-2.70) | 0.4298 |  | 1.12 (0.88-1.39) | 0.3323 |  |  | 0.30 (0.17-0.53) | < 0.0001 |  | 0.91 (0.80-1.03) | 0.1320 |  |
| Model 2 | 1.30 (0.64-2.64) | 0.4672 |  | 1.12 (0.89-1.10) | 0.3334 |  |  | 0.30 (0.17-0.53) | < 0.0001 |  | 0.91 (0.80-1.03) | 0.1505 |  |
| Model 3 | 1.29 (0.63-2.63) | 0.4861 |  | 1.14 (0.91-1.42) | 0.2604 |  |  | 0.30 (0.17-0.53) | < 0.0001 |  | 0.92 (0.81-1.04) | 0.1697 |  |
| **Renal failure** |  |  |  |  |  | 0.4463 |  |  |  |  |  |  | 0.2891 |
| Model 1 | 0.92 (0.66-1.28) | 0.6392 |  | 1.15 (0.92-1.45) | 0.2262 |  |  | 1.01 (0.82-1.24) | 0.9365 |  | 0.90 (0.72-1.12) | 0.3486 |  |
| Model 2 | 0.94 (0.68-1.31) | 0.7092 |  | 1.19 (0.94-1.49) | 0.1467 |  |  | 0.99 (0.80-1.21) | 0.9089 |  | 0.89 (0.71-1.11) | 0.2898 |  |
| Model 3 | 0.94 (0.68-1.31) | 0.7199 |  | 1.14 (0.91-1.44) | 0.2592 |  |  | 0.98 (0.80-1.20) | 0.8546 |  | 0.88 (0.71-1.10) | 0.2775 |  |
| **CKD** |  |  |  |  |  | 0.8719 |  |  |  |  |  |  | 0.0448 |
| Model 1 | 0.91 (0.63-1.32) | 0.6209 |  | 1.06 (0.82-1.38) | 0.6535 |  |  | 1.03 (0.82-1.29) | 0.8138 |  | 0.93 (0.73-1.17) | 0.5216 |  |
| Model 2 | 0.93 (0.64-1.35) | 0.7122 |  | 1.09 (0.84-1.43) | 0.5073 |  |  | 1.02 (0.81-1.27) | 0.8888 |  | 0.92 (0.73-1.16) | 0.4721 |  |
| Model 3 | 0.93 (0.64-1.35) | 0.6983 |  | 1.04 (0.79-1.35) | 0.7909 |  |  | 1.01 (0.80-1.26) | 0.9464 |  | 0.92 (0.72-1.16) | 0.4606 |  |
| **T2D** |  |  |  |  |  | 0.4604 |  |  |  |  |  |  | 0.1037 |
| Model 1 | 0.98 (0.78-1.23) | 0.8294 |  | 0.95 (0.81-1.11) | 0.5260 |  |  | 0.94 (0.83-1.06) | 0.3080 |  | 0.92 (0.83-1.03) | 0.1403 |  |
| Model 2 | 1.03 (0.82-1.30) | 0.7906 |  | 1.05 (0.90-1.23) | 0.5412 |  |  | 0.94 (0.83-1.07) | 0.3449 |  | 0.94 (0.85-1.05) | 0.2576 |  |
| Model 3 | 1.00 (0.80-1.26) | 0.9738 |  | 1.04 (0.89-1.22) | 0.5986 |  |  | 0.92 (0.81-1.05) | 0.2113 |  | 0.94 (0.84-1.04) | 0.2112 |  |
| **Hypertension** |  |  |  |  |  | < 0.0001 |  |  |  |  |  |  | < 0.0001 |
| Model 1 | 1.10 (0.91-1.32) | 0.3204 |  | 0.85 (0.74-0.98) | 0.0247 |  |  | 1.09 (1.00-1.19) | 0.0471 |  | 0.90 (0.82-1.00) | 0.0488 |  |
| Model 2 | 1.12 (0.93-1.35) | 0.2415 |  | 0.89 (0.78-1.02) | 0.0896 |  |  | 1.09 (1.00-1.19) | 0.0580 |  | 0.91 (0.83-1.01) | 0.0801 |  |
| Model 3 | 1.12 (0.93-1.35) | 0.2316 |  | 0.89 (0.79-1.03) | 0.1100 |  |  | 1.09 (1.00-1.19) | 0.0602 |  | 0.91 (0.82-1.00) | 0.0582 |  |
| **Dorsalgia** |  |  |  |  |  | 0.0027 |  |  |  |  |  |  | 0.0022 |
| Model 1 | 0.89 (0.58-1.36) | 0.5955 |  | 0.85 (0.76-0.96) | 0.0063 |  |  | 0.68 (0.56-0.83) | 0.0002 |  | 0.93 (0.85-1.02) | 0.1114 |  |
| Model 2 | 0.86 (0.56-1.32) | 0.4962 |  | 0.86 (0.77-0.96) | 0.0094 |  |  | 0.69 (0.57-0.85) | 0.0003 |  | 0.93 (0.85-1.02) | 0.1228 |  |
| Model 3 | 0.88 (0.57-1.34) | 0.5434 |  | 0.85 (0.76-0.96) | 0.0068 |  |  | 0.69 (0.57-0.85) | 0.0003 |  | 0.92 (0.84-1.01) | 0.0829 |  |
| **Injury & poisoning** |  |  |  |  |  | < 0.0001 |  |  |  |  |  |  | < 0.0001 |
| Model 1 | 1.01 (0.86-1.19) | 0.8808 |  | 0.95 (0.88-1.03) | 0.1902 |  |  | 0.93 (0.86-1.00) | 0.0498 |  | 0.91 (0.86-0.96) | 0.0014 |  |
| Model 2 | 1.00 (0.85-1.17) | 0.9902 |  | 0.95 (0.88-1.03) | 0.2096 |  |  | 0.93 (0.86-1.00) | 0.0607 |  | 0.91 (0.86-0.97) | 0.0022 |  |
| Model 3 | 1.00 (0.85-1.17) | 0.9625 |  | 0.96 (0.89-1.03) | 0.2737 |  |  | 0.93 (0.86-1.00) | 0.0568 |  | 0.91 (0.86-0.96) | 0.0015 |  |

**Abbreviations:** CVD, cardiovascular disease; IH, intracerebral hemorrhage; IHD, ischemic heart disease; IS, ischemic stroke; COPD, chronic obstructive pulmonary disease; CKD, chronic kidney disease; T2D, type 2 diabetes.

**Model 1:** adjusted for age, study area, highest education, household income, and household size.

**Model 2:** adjusted for covariates in model 1, alcohol drinking, smoking, dietary habits, physical activity, and BMI.

**Model 3:** adjusted for covariates in model 2, history of diabetes, hypertension, respiratory disease, CVD, or cancer at baseline, self-reported satisfaction level of life, and family history of the analyzed disease (adjusted for only in corresponding analysis), self-reported satisfaction level of life, and menopausal status (for women only).

* *P* values for interaction in model 3.

**Table S11 Sex-stratified sensitivity analyses for associations of marital status and 30 disease categories after excluding those who followed for no more than two years (lived without vs. with a spouse)**

| **Disease categories** | **Men** | |  | **Women** | |
| --- | --- | --- | --- | --- | --- |
|  | **HR (95%CI)** | ***P*** |  | **HR (95%CI)** | ***P*** |
| **Mental and behavioural disorders** | 1.41 (1.18-1.68) | 0.0001 |  | 1.02 (0.90-1.14) | 0.7781 |
| **Schizophrenia, schizotypal and delusional disorders** | 2.49 (1.71-3.64) | < 0.0001 |  | 1.53 (1.12-2.07) | 0.0067 |
| **Mood [affective] disorders** | 1.35 (0.77-2.34) | 0.2933 |  | 1.01 (0.74-1.38) | 0.9319 |
| **Neurotic, stress-related and somatoform disorders** | 1.11 (0.83-1.47) | 0.4922 |  | 0.96 (0.83-1.13) | 0.6460 |
| **CVD** | 1.08 (1.04-1.11) | < 0.0001 |  | 0.96 (0.94-0.99) | 0.0019 |
| **IH** | 1.29 (1.18-1.4) | < 0.0001 |  | 1.06 (0.98-1.15) | 0.1735 |
| **Cerebrovascular disease** | 1.08 (1.04-1.13) | 0.0002 |  | 0.96 (0.93-0.99) | 0.0031 |
| **IS** | 1.05 (0.99-1.10) | 0.0820 |  | 0.95 (0.92-0.98) | 0.0043 |
| **IHD** | 1.06 (1.00-1.12) | 0.0500 |  | 0.93 (0.90-0.97) | 0.0001 |
| **Cancer** | 1.04 (0.98-1.11) | 0.2158 |  | 0.96 (0.91-1.01) | 0.1291 |
| **Gastric cancer** | 1.09 (0.94-1.27) | 0.2542 |  | 0.98 (0.82-1.15) | 0.7735 |
| **Lung cancer** | 1.04 (0.92-1.17) | 0.5636 |  | 0.90 (0.80-1.01) | 0.0795 |
| **Liver cancer** | 1.03 (0.86-1.23) | 0.7604 |  | 0.99 (0.83-1.19) | 0.9469 |
| **Breast cancer** | NA | NA |  | 0.94 (0.81-1.09) | 0.4191 |
| **Diseases of the respiratory system** | 0.94 (0.90-0.99) | 0.0150 |  | 0.93 (0.90-0.96) | ,<0.0001 |
| **COPD** | 1.20 (1.10-1.30) | < 0.0001 |  | 0.97 (0.91-1.04) | 0.4301 |
| **Bronchitis** | 1.04 (0.96-1.12) | 0.3431 |  | 0.87 (0.83-0.92) | <0.0001 |
| **Pneumonia** | 1.00 (0.94-1.07) | 0.9334 |  | 0.90 (0.86-0.94) | <0.0001 |
| **Acute upper respiratory infection** | 0.92 (0.85-1.00) | 0.0523 |  | 0.86 (0.81-0.91) | <0.0001 |
| **Diseases of the digestive system** | 0.97 (0.92-1.02) | 0.2583 |  | 0.92 (0.88-0.95) | <0.0001 |
| **Gastritis and duodenitis** | 1.02 (0.94-1.10) | 0.7021 |  | 0.85 (0.80-0.90) | <0.0001 |
| **Cholelithiasis and cholecystitis** | 0.97 (0.85-1.10) | 0.5971 |  | 0.90 (0.83-0.96) | 0.0031 |
| **Diseases of the genitourinary system** | 0.97 (0.91-1.03) | 0.3593 |  | 0.88 (0.84-0.92) | <0.0001 |
| **Urethritis and urethral syndrome** | 1.29 (0.99-1.67) | 0.0596 |  | 0.81 (0.69-0.96) | 0.0139 |
| **Renal failure** | 1.11 (0.91-1.35) | 0.2895 |  | 0.93 (0.80-1.09) | 0.3683 |
| **CKD** | 1.01 (0.80-1.26) | 0.9581 |  | 0.93 (0.79-1.10) | 0.4071 |
| **T2D** | 1.05 (0.92-1.20) | 0.4874 |  | 0.97 (0.91-1.04) | 0.4301 |
| **Hypertension** | 0.98 (0.87-1.11) | 0.7551 |  | 0.97 (0.91-1.05) | 0.4687 |
| **Dorsalgia** | 0.85 (0.75-0.97) | 0.0148 |  | 0.85 (0.77-0.94) | 0.0016 |
| **Injury & poisoning** | 0.95 (0.88-1.03) | 0.2020 |  | 0.92 (0.87-0.96) | 0.0004 |

**Abbreviations:** CVD, cardiovascular disease; IH, intracerebral hemorrhage; IHD, ischemic heart disease; IS, ischemic stroke; COPD, chronic obstructive pulmonary disease; CKD, chronic kidney disease; T2D, type 2 diabetes.

**Model:** adjusted for age, study area, highest education, household income, household size**,** alcohol drinking, smoking, dietary habits, physical activity, BMI, history of diabetes, hypertension, respiratory disease, CVD, or cancer at baseline, self-reported satisfaction level of life, and family history of the analyzed disease (adjusted for only in corresponding analysis), self-reported satisfaction level of life, and menopausal status (for women only).

**Table S12 Sex-stratified analyses for associations of four-group marital status (live with a spouse, widowed, separated/divorced, and un****married) and 30 disease categories**

| **Disease categories** | **Men** | |  | **Women** | |
| --- | --- | --- | --- | --- | --- |
|  | **HR (95%CI)** | ***P*** |  | **HR (95%CI)** | ***P*** |
| **Mental and behavioural disorders** |  |  |  |  |  |
| Widowed | 1.05 (0.85-1.30) | 0.6440 |  | 0.94 (0.84-1.06) | 0.3077 |
| Separated/Divorced | 1.65 (1.23-2.22) | 0.0008 |  | 1.30 (1.02-1.65) | 0.0308 |
| Unmarried | 1.74 (1.32-2.28) | 0.0001 |  | 1.91 (1.20-3.06) | 0.0069 |
| **Schizophrenia, schizotypal and delusional disorders** |  |  |  |  |  |
| Widowed | 1.14 (0.61-2.13) | 0.6783 |  | 1.15 (0.81-1.61) | 0.4348 |
| Separated/Divorced | 3.60 (2.09-6.19) | <0.0001 |  | 1.77 (0.99-3.16) | 0.0546 |
| Unmarried | 4.50 (2.79-7.25) | <0.0001 |  | 7.53 (3.97-14.31) | <0.0001 |
| **Mood [affective] disorders** |  |  |  |  |  |
| Widowed | 1.49 (0.83-2.67) | 0.1800 |  | 1.02 (0.76-1.37) | 0.8959 |
| Separated/Divorced | 1.10 (0.39-3.05) | 0.8595 |  | 1.47 (0.78-2.75) | 0.2346 |
| Unmarried | 1.21 (0.43-3.38) | 0.7210 |  | 0.99 (0.14-7.12) | 0.9913 |
| **Neurotic, stress-related and somatoform disorders** |  |  |  |  |  |
| Widowed | 0.82 (0.58-1.15) | 0.2482 |  | 0.88 (0.76-1.03) | 0.1032 |
| Separated/Divorced | 1.26 (0.78-2.02) | 0.3422 |  | 1.34 (0.99-1.81) | 0.0601 |
| Unmarried | 0.95 (0.60-1.52) | 0.8404 |  | 1.02 (0.45-2.29) | 0.9630 |
| **CVD** |  |  |  |  |  |
| Widowed | 1.02 (0.98-1.06) | 0.2485 |  | 0.96 (0.94-0.99) | 0.0017 |
| Separated/Divorced | 1.19 (1.10-1.28) | <0.0001 |  | 1.15 (1.08-1.24) | <0.0001 |
| Unmarried | 1.19 (1.11-1.29) | <0.0001 |  | 0.95 (0.79-1.15) | 0.6202 |
| **IH** |  |  |  |  |  |
| Widowed | 1.16 (1.06-1.28) | 0.0016 |  | 1.06 (0.98-1.15) | 0.1168 |
| Separated/Divorced | 1.49 (1.21-1.83) | 0.0002 |  | 1.08 (0.76-1.54) | 0.6520 |
| Unmarried | 1.61 (1.34-1.94) | <0.0001 |  | 1.29 (0.58-2.88) | 0.5395 |
| **Cerebrovascular disease** |  |  |  |  |  |
| Widowed | 1.03 (0.99-1.08) | 0.1685 |  | 0.97 (0.94-0.99) | 0.0139 |
| Separated/Divorced | 1.17 (1.06-1.28) | 0.0009 |  | 1.11 (1.02-1.21) | 0.0140 |
| Unmarried | 1.21 (1.10-1.33) | 0.0001 |  | 0.89 (0.72-1.12) | 0.3291 |
| **IS** |  |  |  |  |  |
| Widowed | 1.00(0.95-1.06) | 0.9346 |  | 0.95 (0.92-0.99) | 0.0066 |
| Separated/Divorced | 1.15 (1.02-1.29) | 0.0206 |  | 1.13 (1.02-1.26) | 0.0221 |
| Unmarried | 1.18 (1.04-1.35) | 0.0126 |  | 0.93 (0.70-1.23) | 0.6218 |
| **IHD** |  |  |  |  |  |
| Widowed | 0.98 (0.93-1.05) | 0.5906 |  | 0.93 (0.90-0.97) | 0.0002 |
| Separated/Divorced | 1.19 (1.06-1.35) | 0.0033 |  | 1.16 (1.04-1.28) | 0.0061 |
| Unmarried | 1.29 (1.13-1.46) | 0.0004 |  | 1.01 (0.76-1.33) | 0.9507 |
| **Cancer** |  |  |  |  |  |
| Widowed | 1.04 (0.97-1.11) | 0.2445 |  | 0.97 (0.93-1.03) | 0.3195 |
| Separated/Divorced | 1.09 (0.94-1.27) | 0.2316 |  | 1.01 (0.88-1.17) | 0.8703 |
| Unmarried | 1.13 (0.98-1.32) | 0.0998 |  | 1.40 (1.03-1.91) | 0.0293 |
| **Gastric cancer** |  |  |  |  |  |
| Widowed | 1.10 (0.94-1.28) | 0.2279 |  | 1.03 (0.88-1.21) | 0.6887 |
| Separated/Divorced | 1.23 (0.83-1.80) | 0.3029 |  | 0.88 (0.47-1.66) | 0.6933 |
| Unmarried | 1.45 (1.01-2.07) | 0.0426 |  | 0.61 (0.09-4.35) | 0.6211 |
| **Lung cancer** |  |  |  |  |  |
| Widowed | 1.01 (0.88-1.15) | 0.9223 |  | 0.94 (0.84-1.05) | 0.2506 |
| Separated/Divorced | 1.21 (0.92-1.59) | 0.1746 |  | 0.75 (0.49-1.15) | 0.1920 |
| Unmarried | 1.08 (0.80-1.47) | 0.6017 |  | 0.70 (0.22-2.17) | 0.5352 |
| **Liver cancer** |  |  |  |  |  |
| Widowed | 0.95 (0.78-1.15) | 0.6046 |  | 1.03 (0.87-1.22) | 0.7372 |
| Separated/Divorced | 1.21 (0.85-1.72) | 0.2959 |  | 0.71 (0.35-1.45) | 0.3489 |
| Unmarried | 1.17 (0.81-1.70) | 0.4009 |  | NA | NA |
| **Breast cancer** |  |  |  |  |  |
| Widowed | NA | NA |  | 0.93 (0.79-1.09) | 0.3476 |
| Separated/Divorced | NA | NA |  | 0.95 (0.71-1.26) | 0.7096 |
| Unmarried | NA | NA |  | 1.42 (0.82-2.47) | 0.2107 |
| **Diseases of the respiratory system** |  |  |  |  |  |
| Widowed | 0.96 (0.92-1.01) | 0.1346 |  | 0.94 (0.91-0.97) | 0.0001 |
| Separated/Divorced | 0.92 (0.84-1.02) | 0.1060 |  | 0.96 (0.87-1.06) | 0.4570 |
| Unmarried | 0.94 (0.86-1.03) | 0.1934 |  | 0.94 (0.74-1.19) | 0.6105 |
| **COPD** |  |  |  |  |  |
| Widowed | 1.09 (0.99-1.19) | 0.0674 |  | 0.97 (0.90-1.03) | 0.3201 |
| Separated/Divorced | 1.30 (1.05-1.61) | 0.0147 |  | 1.02 (0.74-1.40) | 0.9109 |
| Unmarried | 1.74 (1.48-2.05) | <0.0001 |  | 1.61 (0.86-3.00) | 0.1347 |
| **Bronchitis** |  |  |  |  |  |
| Widowed | 0.98 (0.90-1.06) | 0.5755 |  | 0.90 (0.86-0.95) | 0.0001 |
| Separated/Divorced | 1.05 (0.88-1.25) | 0.6088 |  | 0.93 (0.77-1.12) | 0.4510 |
| Unmarried | 1.29 (1.12-1.48) | 0.0003 |  | 0.99 (0.64-1.54) | 0.9626 |
| **Pneumonia** |  |  |  |  |  |
| Widowed | 0.97 (0.90-1.04) | 0.3663 |  | 0.88 (0.84-0.93) | <0.0001 |
| Separated/Divorced | 1.08 (0.93-1.26) | 0.3061 |  | 1.06 (0.92-1.23) | 0.4243 |
| Unmarried | 1.20 (1.04-1.38) | 0.0134 |  | 1.10 (0.76-1.59) | 0.6029 |
| **Acute upper respiratory infection** |  |  |  |  |  |
| Widowed | 1.00 (0.92-1.07) | 0.9141 |  | 0.92 (0.88-0.97) | 0.0008 |
| Separated/Divorced | 0.86 (0.73-1.00) | 0.0518 |  | 0.78 (0.65-0.95) | 0.0113 |
| Unmarried | 0.86 (0.76-0.98) | 0.0189 |  | 0.67 (0.43-1.02) | 0.0632 |
| **Diseases of the digestive system** |  |  |  |  |  |
| Widowed | 0.97 (0.91-1.02) | 0.2310 |  | 0.92 (0.89-0.95) | <0.0001 |
| Separated/Divorced | 0.97 (0.88-1.08) | 0.6153 |  | 1.04 (0.94-1.14) | 0.4566 |
| Unmarried | 0.97 (0.88-1.07) | 0.5704 |  | 0.77 (0.59-1.01) | 0.0613 |
| **Gastritis and duodenitis** |  |  |  |  |  |
| Widowed | 0.98 (0.90-1.08) | 0.7363 |  | 0.87 (0.83-0.92) | <0.0001 |
| Separated/Divorced | 0.99 (0.84-1.18) | 0.9546 |  | 0.99 (0.85-1.15) | 0.9194 |
| Unmarried | 1.09 (0.94-1.26) | 0.2637 |  | 0.68 (0.42-1.10) | 0.1145 |
| **Cholelithiasis and cholecystitis** |  |  |  |  |  |
| Widowed | 0.89 (0.77-1.03) | 0.1077 |  | 0.89 (0.83-0.96) | 0.0018 |
| Separated/Divorced | 0.89 (0.67-1.19) | 0.4272 |  | 1.09 (0.87-1.37) | 0.4534 |
| Unmarried | 1.15 (0.91-1.46) | 0.2344 |  | 0.67 (0.32-1.41) | 0.2911 |
| **Diseases of the genitourinary system** |  |  |  |  |  |
| Widowed | 0.99 (0.92-1.06) | 0.7918 |  | 0.87 (0.83-0.91) | <0.0001 |
| Separated/Divorced | 0.97 (0.85-1.12) | 0.6867 |  | 1.07 (0.96-1.19) | 0.2228 |
| Unmarried | 0.88 (0.77-1.01) | 0.0646 |  | 0.71 (0.52-0.98) | 0.0350 |
| **Urethritis and urethral syndrome** |  |  |  |  |  |
| Widowed | 1.21 (0.93-1.56) | 0.1531 |  | 0.87 (0.77-0.99) | 0.0295 |
| Separated/Divorced | 1.13 (0.67-1.90) | 0.6397 |  | 0.75 (0.42-1.32) | 0.3166 |
| Unmarried | 0.90 (0.56-1.44) | 0.6620 |  | NA | NA |
| **Renal failure** |  |  |  |  |  |
| Widowed | 0.96 (0.77-1.21) | 0.7554 |  | 0.91 (0.78-1.07) | 0.2466 |
| Separated/Divorced | 1.24 (0.81-1.91) | 0.3275 |  | 1.40 (0.89-2.21) | 0.1496 |
| Unmarried | 1.40 (0.92-2.13) | 0.1174 |  | 1.26 (0.40-3.92) | 0.6952 |
| **CKD** |  |  |  |  |  |
| Widowed | 0.93 (0.71-1.22) | 0.6117 |  | 0.94 (0.79-1.11) | 0.4466 |
| Separated/Divorced | 0.97 (0.59-1.61) | 0.9149 |  | 1.20 (0.76-1.89) | 0.4375 |
| Unmarried | 1.27 (0.81-2.02)) | 0.3000 |  | 0.39 (0.05-2.78) | 0.3479 |
| **T2D** |  |  |  |  |  |
| Widowed | 0.94 (0.79-1.10) | 0.4380 |  | 0.97 (0.90-1.03) | 0.3201 |
| Separated/Divorced | 1.15 (0.87-1.53) | 0.3164 |  | 1.02 (0.74-1.40) | 0.9109 |
| Unmarried | 1.25 (0.95-1.64) | 0.1099 |  | 1.61 (0.86-3.00) | 0.1347 |
| **Hypertension** |  |  |  |  |  |
| Widowed | 0.92 (0.80-1.06) | 0.2413 |  | 0.96 (0.90-1.03) | 0.2652 |
| Separated/Divorced | 1.20 (0.95-1.51) | 0.1175 |  | 1.19 (1.01-1.41) | 0.0375 |
| Unmarried | 0.84 (0.64-1.11) | 0.2236 |  | 1.18 (0.80-1.76) | 0.4045 |
| **Dorsalgia** |  |  |  |  |  |
| Widowed | 0.93 (0.81-1.06) | 0.2888 |  | 0.90 (0.83-0.98) | 0.0191 |
| Separated/Divorced | 0.71 (0.53-0.96) | 0.0268 |  | 1.03 (0.75-1.41) | 0.8434 |
| Unmarried | 0.75 (0.59-0.95) | 0.0156 |  | 0.26 (0.07-1.05) | 0.0579 |
| **Injury & poisoning** |  |  |  |  |  |
| Widowed | 1.01 (0.93-1.10) | 0.7744 |  | 0.92 (0.88-0.96) | 0.0004 |
| Separated/Divorced | 0.94 (0.80-1.10) | 0.4550 |  | 1.01 (0.86-1.20) | 0.8668 |
| Unmarried | 0.82 (0.70-0.96) | 0.0112 |  | 1.13 (0.75-1.70) | 0.5725 |

**Abbreviations:** CVD, cardiovascular disease; IH, intracerebral hemorrhage; IHD, ischemic heart disease; IS, ischemic stroke; COPD, chronic obstructive pulmonary disease; CKD, chronic kidney disease; T2D, type 2 diabetes.

**Model:** adjusted for age, study area, highest education, household income, household size**,** alcohol drinking, smoking, dietary habits, physical activity, BMI, history of diabetes, hypertension, respiratory disease, CVD, or cancer at baseline, family history of the analyzed disease (adjusted for only in corresponding analysis), self-reported satisfaction level of life, and menopausal status (for women only).

**Table S13 Adjusted hazard ratios (aHRs) of marital status (lived without vs. with a spouse) with 30 disease categories stratified by sex and birth cohort**

|  | **Men** | | | | | |  | **Women** | | | | | |
| --- | --- | --- | --- | --- | --- | --- | --- | --- | --- | --- | --- | --- | --- |
| **Disease categories** | **< 1955** | |  | **>=1955** | | ***P* for interaction*** |  | **< 1955** | |  | **>=1955** | | ***P* for interaction*** |
|  | **HR (95% CI)** | ***P*** |  | **HR (95% CI)** | ***P*** |  |  | **HR (95% CI)** | ***P*** |  | **HR (95% CI)** | ***P*** |  |
| **Mental and behavioural disorders** |  |  |  |  |  | < 0.0001 |  |  |  |  |  |  | < 0.0001 |
| Model 1 | 1.16 (0.96-1.40) | 0.1200 |  | 2.08 (1.55-2.79) | < 0.0001 |  |  | 0.98 (0.87-1.10) | 0.6935 |  | 1.2 (0.96-1.50) | 0.1172 |  |
| Model 2 | 1.16 (0.96-1.39) | 0.1247 |  | 2.04 (1.52-2.74) | < 0.0001 |  |  | 0.97 (0.86-1.09) | 0.5924 |  | 1.2 (0.96-1.50) | 0.1173 |  |
| Model 3 | 1.13 (0.93-1.36) | 0.2139 |  | 1.99 (1.48-2.67) | < 0.0001 |  |  | 0.97 (0.86-1.09) | 0.5612 |  | 1.19 (0.95-1.49) | 0.1371 |  |
| **Schizophrenia, schizotypal and delusional disorders** |  |  |  |  |  | 0.0174 |  |  |  |  |  |  | 0.0390 |
| Model 1 | 1.69 (1.05-2.71) | 0.0311 |  | 5.03 (3.06-8.27) | < 0.0001 |  |  | 1.25 (0.87-1.80) | 0.2291 |  | 2.08 (1.28-3.38) | 0.0030 |  |
| Model 2 | 1.69 (1.05-2.72) | 0.0313 |  | 5.09 (3.10-8.37) | < 0.0001 |  |  | 1.23 (0.85-1.77) | 0.2672 |  | 2.07 (1.27-3.35) | 0.0032 |  |
| Model 3 | 1.64 (1.01-2.65) | 0.0445 |  | 4.71 (2.84-7.79) | < 0.0001 |  |  | 1.18 (0.82-1.70) | 0.3778 |  | 1.96 (1.21-3.19) | 0.0064 |  |
| **Mood [affective] disorders** |  |  |  |  |  | 0.6984 |  |  |  |  |  |  | 0.1799 |
| Model 1 | 1.38 (0.79-2.40) | 0.2566 |  | 1.45 (0.55-3.85) | 0.4511 |  |  | 0.96 (0.70-1.32) | 0.7996 |  | 1.83 (1.04-3.23) | 0.0360 |  |
| Model 2 | 1.37 (0.79-2.38) | 0.2667 |  | 1.42 (0.54-3.77) | 0.4806 |  |  | 0.95 (0.69-1.30) | 0.7387 |  | 1.83 (1.04-3.22) | 0.0366 |  |
| Model 3 | 1.33 (0.76-2.32) | 0.3154 |  | 1.35 (0.51-3.59) | 0.5459 |  |  | 0.94 (0.68-1.29) | 0.6973 |  | 1.80 (1.02-3.18) | 0.0426 |  |
| **Neurotic, stress-related and somatoform disorders** |  |  |  |  |  | 0.1059 |  |  |  |  |  |  | 0.0042 |
| Model 1 | 0.94 (0.70-1.25) | 0.6540 |  | 1.15 (0.71-1.87) | 0.5741 |  |  | 0.94 (0.80-1.09) | 0.4142 |  | 1.07 (0.79-1.43) | 0.6623 |  |
| Model 2 | 0.92 (0.68-1.23) | 0.5598 |  | 1.13 (0.69-1.84) | 0.6308 |  |  | 0.93 (0.80-1.09) | 0.3676 |  | 1.07 (0.79-1.43) | 0.6718 |  |
| Model 3 | 0.87 (0.64-1.16) | 0.3415 |  | 1.10 (0.68-1.80) | 0.6962 |  |  | 0.93 (0.79-1.08) | 0.3318 |  | 1.05 (0.78-1.41) | 0.7499 |  |
| **CVD** |  |  |  |  |  | < 0.0001 |  |  |  |  |  |  | < 0.0001 |
| Model 1 | 1.06 (1.03-1.10) | 0.0005 |  | 1.16 (1.06-1.27) | 0.0008 |  |  | 0.98 (0.95-1.00) | 0.0499 |  | 1.06 (0.99-1.14) | 0.0791 |  |
| Model 2 | 1.08 (1.04-1.11) | < 0.0001 |  | 1.19 (1.09-1.30) | 0.0001 |  |  | 0.97 (0.95-0.99) | 0.0133 |  | 1.07 (1.00-1.15) | 0.0386 |  |
| Model 3 | 1.05 (1.01-1.09) | 0.0066 |  | 1.16 (1.06-1.26) | 0.0011 |  |  | 0.96 (0.94-0.99) | 0.0036 |  | 1.08 (1.01-1.16) | 0.0223 |  |
| **IH** |  |  |  |  |  | 0.1715 |  |  |  |  |  |  | 0.5253 |
| Model 1 | 1.29 (1.19-1.41) | < 0.0001 |  | 1.44 (1.09-1.92) | 0.0113 |  |  | 1.09 (1.01-1.18) | 0.0341 |  | 1.17 (0.86-1.61) | 0.3171 |  |
| Model 2 | 1.31 (1.20-1.42) | < 0.0001 |  | 1.50 (1.13-1.99) | 0.0056 |  |  | 1.07 (0.99-1.16) | 0.0855 |  | 1.19 (0.87-1.63) | 0.2805 |  |
| Model 3 | 1.24 (1.14-1.35) | < 0.0001 |  | 1.36 (1.02-1.81) | 0.0353 |  |  | 1.06 (0.98-1.14) | 0.1610 |  | 1.20 (0.88-1.64) | 0.2491 |  |
| **Cerebrovascular disease** |  |  |  |  |  | < 0.0001 |  |  |  |  |  |  | < 0.0001 |
| Model 1 | 1.07 (1.02-1.11) | 0.0023 |  | 1.16 (1.04-1.29) | 0.0090 |  |  | 0.98 (0.95-1.00) | 0.0891 |  | 1.04 (0.96-1.13) | 0.3415 |  |
| Model 2 | 1.08 (1.04-1.13) | 0.0002 |  | 1.19 (1.06-1.32) | 0.0022 |  |  | 0.97 (0.94-1.00) | 0.0370 |  | 1.05 (0.97-1.14) | 0.2283 |  |
| Model 3 | 1.06 (1.01-1.10) | 0.0112 |  | 1.15 (1.03-1.28) | 0.0140 |  |  | 0.96 (0.94-0.99) | 0.0127 |  | 1.06 (0.98-1.15) | 0.1406 |  |
| **IS** |  |  |  |  |  | < 0.0001 |  |  |  |  |  |  | < 0.0001 |
| Model 1 | 1.02 (0.96-1.07) | 0.5346 |  | 1.23 (1.06-1.42) | 0.0061 |  |  | 0.97 (0.93-1.00) | 0.0758 |  | 1.07 (0.96-1.21) | 0.2197 |  |
| Model 2 | 1.03 (0.98-1.09) | 0.2235 |  | 1.26 (1.09-1.46) | 0.0022 |  |  | 0.96 (0.92-0.99) | 0.0215 |  | 1.10 (0.98-1.23) | 0.1102 |  |
| Model 3 | 1.01 (0.96-1.07) | 0.7131 |  | 1.23 (1.06-1.42) | 0.0064 |  |  | 0.95 (0.92-0.99) | 0.0090 |  | 1.13 (1.01-1.27) | 0.0395 |  |
| **IHD** |  |  |  |  |  | < 0.0001 |  |  |  |  |  |  | < 0.0001 |
| Model 1 | 1.03 (0.97-1.09) | 0.3065 |  | 1.11 (0.96-1.29) | 0.1709 |  |  | 0.95 (0.92-0.99) | 0.0105 |  | 1.02 (0.92-1.15) | 0.6696 |  |
| Model 2 | 1.05 (0.99-1.11) | 0.1123 |  | 1.14 (0.98-1.32) | 0.0934 |  |  | 0.94 (0.91-0.98) | 0.0021 |  | 1.05 (0.93-1.17) | 0.4387 |  |
| Model 3 | 1.03 (0.97-1.09) | 0.3067 |  | 1.11 (0.95-1.29) | 0.1791 |  |  | 0.94 (0.91-0.98) | 0.0013 |  | 1.05 (0.94-1.18) | 0.3623 |  |
| **Cancer** |  |  |  |  |  | 0.9995 |  |  |  |  |  |  | 0.9047 |
| Model 1 | 1.06 (1.00-1.13) | 0.0444 |  | 1.00 (0.83-1.22) | 0.9635 |  |  | 0.99 (0.94-1.04) | 0.5897 |  | 1.00 (0.87-1.15) | 0.9531 |  |
| Model 2 | 1.06 (1.00-1.13) | 0.0551 |  | 1.01 (0.83-1.22) | 0.9523 |  |  | 0.98 (0.93-1.03) | 0.4275 |  | 1.00 (0.87-1.15) | 0.9998 |  |
| Model 3 | 1.06 (1.00-1.13) | 0.0575 |  | 1.02 (0.84-1.23) | 0.8780 |  |  | 0.98 (0.93-1.03) | 0.4305 |  | 1.00 (0.87-1.16) | 0.9469 |  |
| **Gastric cancer** |  |  |  |  |  | 0.2092 |  |  |  |  |  |  | 0.0288 |
| Model 1 | 1.17 (1.02-1.35) | 0.0266 |  | 0.90 (0.50-1.61) | 0.7273 |  |  | 1.07 (0.91-1.25) | 0.4354 |  | 0.52 (0.23-1.16) | 0.1094 |  |
| Model 2 | 1.16 (1.01-1.34) | 0.0340 |  | 0.92 (0.51-1.64) | 0.7682 |  |  | 1.06 (0.90-1.25) | 0.4624 |  | 0.52 (0.23-1.15) | 0.1066 |  |
| Model 3 | 1.16 (1.01-1.34) | 0.0335 |  | 0.94 (0.52-1.68) | 0.8261 |  |  | 1.06 (0.91-1.25) | 0.4466 |  | 0.50 (0.22-1.11) | 0.0898 |  |
| **Lung cancer** |  |  |  |  |  | 0.5567 |  |  |  |  |  |  | 0.2540 |
| Model 1 | 1.04 (0.92-1.17) | 0.5339 |  | 1.02 (0.68-1.52) | 0.9252 |  |  | 0.95 (0.85-1.06) | 0.3662 |  | 0.84 (0.54-1.32) | 0.4586 |  |
| Model 2 | 1.03 (0.91-1.16) | 0.6420 |  | 0.99 (0.66-1.48) | 0.9742 |  |  | 0.93 (0.83-1.04) | 0.1800 |  | 0.83 (0.53-1.30) | 0.4162 |  |
| Model 3 | 1.03 (0.91-1.16) | 0.6164 |  | 1.01 (0.67-1.50) | 0.9806 |  |  | 0.93 (0.83-1.04) | 0.1956 |  | 0.84 (0.54-1.33) | 0.4645 |  |
| **Liver cancer** |  |  |  |  |  | 0.8932 |  |  |  |  |  |  | 0.3660 |
| Model 1 | 1.03 (0.87-1.23) | 0.7349 |  | 0.95 (0.61-1.49) | 0.8353 |  |  | 1.01 (0.85-1.20) | 0.8913 |  | 1.15 (0.65-2.03) | 0.6260 |  |
| Model 2 | 1.02 (0.86-1.22) | 0.7809 |  | 0.95 (0.61-1.49) | 0.8319 |  |  | 1.00 (0.84-1.19) | 0.9973 |  | 1.13 (0.64-2.00) | 0.6718 |  |
| Model 3 | 1.03 (0.86-1.22) | 0.7576 |  | 0.96 (0.61-1.50) | 0.8509 |  |  | 1.00 (0.84-1.19) | 0.9968 |  | 1.09 (0.61-1.92) | 0.7776 |  |
| **Breast cancer** |  |  |  |  |  | 0.7201 |  |  |  |  |  |  | 0.7490 |
| Model 1 | 0.28 (0.04-2.05) | 0.2081 |  | NA | 0.9981 |  |  | 0.96 (0.81-1.13) | 0.6260 |  | 0.88 (0.67-1.15) | 0.3324 |  |
| Model 2 | 0.27 (0.04-2.03) | 0.2043 |  | NA | 0.9980 |  |  | 0.96 (0.81-1.13) | 0.6252 |  | 0.89 (0.68-1.16) | 0.3868 |  |
| Model 3 | 0.29 (0.04-2.16) | 0.2268 |  | NA | 0.9980 |  |  | 0.96 (0.82-1.14) | 0.6638 |  | 0.91 (0.69-1.19) | 0.4778 |  |
| **Diseases of the respiratory system** |  |  |  |  |  | 0.0217 |  |  |  |  |  |  | 0.0074 |
| Model 1 | 0.98 (0.94-1.03) | 0.4448 |  | 0.85 (0.78-0.94) | 0.0011 |  |  | 0.94 (0.91-0.97) | <0.0001 |  | 1.00 (0.93-1.08) | 0.9543 |  |
| Model 2 | 0.98 (0.93-1.02) | 0.3528 |  | 0.85 (0.77-0.93) | 0.0007 |  |  | 0.94 (0.91-0.97) | 0.0001 |  | 1.00 (0.93-1.08) | 0.9353 |  |
| Model 3 | 0.98 (0.93-1.02) | 0.3312 |  | 0.85 (0.77-0.94) | 0.0009 |  |  | 0.93 (0.90-0.96) | <0.0001 |  | 0.99 (0.92-1.07) | 0.8775 |  |
| **COPD** |  |  |  |  |  | < 0.0001 |  |  |  |  |  |  | 0.4520 |
| Model 1 | 1.19 (1.10-1.29) | <0.0001 |  | 1.50 (1.12-2.01) | 0.0061 |  |  | 0.98 (0.91-1.04) | 0.4712 |  | 1.00 (0.74-1.35) | 0.9794 |  |
| Model 2 | 1.17 (1.08-1.27) | 0.0001 |  | 1.46 (1.09-1.95) | 0.0106 |  |  | 0.96 (0.90-1.03) | 0.2921 |  | 0.99 (0.73-1.33) | 0.9289 |  |
| Model 3 | 1.17 (1.08-1.27) | 0.0001 |  | 1.46 (1.09-1.95) | 0.0110 |  |  | 0.96 (0.90-1.03) | 0.2492 |  | 0.99 (0.73-1.33) | 0.9272 |  |
| **Bronchitis** |  |  |  |  |  | 0.0734 |  |  |  |  |  |  | 0.6455 |
| Model 1 | 1.08 (1.00-1.16) | 0.0448 |  | 0.93 (0.76-1.14) | 0.4704 |  |  | 0.89 (0.84-0.94) | <0.0001 |  | 1.03 (0.89-1.20) | 0.6960 |  |
| Model 2 | 1.07 (0.99-1.15) | 0.0881 |  | 0.91 (0.75-1.12) | 0.3904 |  |  | 0.89 (0.84-0.94) | <0.0001 |  | 1.03 (0.89-1.20) | 0.6982 |  |
| Model 3 | 1.05 (0.97-1.13) | 0.2124 |  | 0.90 (0.74-1.11) | 0.3222 |  |  | 0.88 (0.84-0.93) | <0.0001 |  | 1.01 (0.87-1.18) | 0.8740 |  |
| **Pneumonia** |  |  |  |  |  | < 0.0001 |  |  |  |  |  |  | < 0.0001 |
| Model 1 | 1.02 (0.95-1.09) | 0.6209 |  | 1.07 (0.89-1.28) | 0.4809 |  |  | 0.89 (0.85-0.93) | <0.0001 |  | 1.03 (0.89-1.18) | 0.7182 |  |
| Model 2 | 1.01 (0.95-1.08) | 0.7479 |  | 1.05 (0.88-1.26) | 0.5833 |  |  | 0.89 (0.85-0.93) | <0.0001 |  | 1.03 (0.89-1.18) | 0.7276 |  |
| Model 3 | 1.01 (0.94-1.08) | 0.8261 |  | 1.05 (0.88-1.27) | 0.5735 |  |  | 0.89 (0.84-0.93) | <0.0001 |  | 1.02 (0.88-1.18) | 0.8093 |  |
| **Acute upper respiratory infection** |  |  |  |  |  | < 0.0001 |  |  |  |  |  |  | < 0.0001 |
| Model 1 | 1.01 (0.95-1.09) | 0.7057 |  | 0.72 (0.62-0.84) | < 0.0001 |  |  | 0.91 (0.86-0.95) | 0.0001 |  | 0.96 (0.85-1.08) | 0.4985 |  |
| Model 2 | 1.01 (0.94-1.08) | 0.7919 |  | 0.72 (0.62-0.84) | < 0.0001 |  |  | 0.91 (0.86-0.95) | 0.0002 |  | 0.96 (0.85-1.09) | 0.5289 |  |
| Model 3 | 1.01 (0.94-1.08) | 0.7731 |  | 0.71 (0.61-0.83) | < 0.0001 |  |  | 0.90 (0.86-0.95) | <0.0001 |  | 0.95 (0.84-1.07) | 0.3761 |  |
| **Diseases of the digestive system** |  |  |  |  |  | 0.0093 |  |  |  |  |  |  | < 0.0001 |
| Model 1 | 0.99 (0.94-1.04) | 0.6418 |  | 0.91 (0.82-1.00) | 0.0485 |  |  | 0.90 (0.87-0.93) | <0.0001 |  | 1.06 (0.98-1.15) | 0.1288 |  |
| Model 2 | 0.99 (0.94-1.04) | 0.5950 |  | 0.91 (0.83-1.00) | 0.0564 |  |  | 0.90 (0.87-0.94) | <0.0001 |  | 1.06 (0.98-1.15) | 0.1197 |  |
| Model 3 | 0.98 (0.93-1.04) | 0.5674 |  | 0.91 (0.82-1.00) | 0.0533 |  |  | 0.90 (0.87-0.93) | <0.0001 |  | 1.06 (0.98-1.14) | 0.1442 |  |
| **Gastritis and duodenitis** |  |  |  |  |  | 0.0862 |  |  |  |  |  |  | 0.0020 |
| Model 1 | 1.08 (0.99-1.17) | 0.0795 |  | 0.81 (0.68-0.96) | 0.0171 |  |  | 0.86 (0.82-0.91) | <0.0001 |  | 1.05 (0.92-1.18) | 0.4828 |  |
| Model 2 | 1.06 (0.98-1.15) | 0.1509 |  | 0.80 (0.67-0.95) | 0.0102 |  |  | 0.86 (0.82-0.91) | <0.0001 |  | 1.04 (0.92-1.17) | 0.5691 |  |
| Model 3 | 1.06 (0.97-1.15) | 0.1783 |  | 0.79 (0.67-0.94) | 0.0095 |  |  | 0.86 (0.81-0.91) | <0.0001 |  | 1.03 (0.91-1.17) | 0.6238 |  |
| **Cholelithiasis and cholecystitis** |  |  |  |  |  | 0.4829 |  |  |  |  |  |  | 0.0300 |
| Model 1 | 0.92 (0.80-1.05) | 0.2138 |  | 0.83 (0.64-1.09) | 0.1763 |  |  | 0.87 (0.81-0.94) | 0.0003 |  | 1.19 (1.01-1.40) | 0.0405 |  |
| Model 2 | 0.94 (0.82-1.07) | 0.3463 |  | 0.86 (0.66-1.12) | 0.2734 |  |  | 0.87 (0.81-0.94) | 0.0002 |  | 1.21 (1.02-1.42) | 0.0243 |  |
| Model 3 | 0.94 (0.82-1.07) | 0.3377 |  | 0.85 (0.65-1.12) | 0.2485 |  |  | 0.87 (0.81-0.94) | 0.0002 |  | 1.21 (1.02-1.42) | 0.0264 |  |
| **Diseases of the genitourinary system** |  |  |  |  |  | 0.0070 |  |  |  |  |  |  | 0.0191 |
| Model 1 | 0.99 (0.92-1.05) | 0.7008 |  | 0.91 (0.79-1.05) | 0.1876 |  |  | 0.86 (0.82-0.91) | <0.0001 |  | 0.95 (0.87-1.03) | 0.2151 |  |
| Model 2 | 0.99 (0.93-1.06) | 0.7835 |  | 0.92 (0.80-1.06) | 0.2711 |  |  | 0.86 (0.82-0.91) | <0.0001 |  | 0.95 (0.87-1.04) | 0.2577 |  |
| Model 3 | 0.99 (0.93-1.06) | 0.8020 |  | 0.92 (0.80-1.06) | 0.2365 |  |  | 0.86 (0.82-0.91) | <0.0001 |  | 0.97 (0.89-1.05) | 0.4155 |  |
| **Urethritis and urethral syndrome** |  |  |  |  |  | 0.0006 |  |  |  |  |  |  | < 0.0001 |
| Model 1 | 1.06 (0.83-1.36) | 0.6397 |  | 1.28 (0.80-2.05) | 0.3111 |  |  | 0.81 (0.71-0.93) | 0.0022 |  | 0.93 (0.65-1.31) | 0.6701 |  |
| Model 2 | 1.06 (0.83-1.36) | 0.6313 |  | 1.28 (0.79-2.05) | 0.3154 |  |  | 0.82 (0.72-0.93) | 0.0030 |  | 0.93 (0.65-1.32) | 0.6792 |  |
| Model 3 | 1.07 (0.84-1.38) | 0.5656 |  | 1.30 (0.81-2.10) | 0.2748 |  |  | 0.82 (0.72-0.94) | 0.0037 |  | 0.95 (0.67-1.34) | 0.7566 |  |
| **Renal failure** |  |  |  |  |  | 0.0906 |  |  |  |  |  |  | 0.0007 |
| Model 1 | 1.01 (0.83-1.25) | 0.8890 |  | 1.21 (0.72-2.04) | 0.4752 |  |  | 0.98 (0.84-1.15) | 0.8325 |  | 0.85 (0.50-1.45) | 0.5546 |  |
| Model 2 | 1.04 (0.85-1.28) | 0.7045 |  | 1.23 (0.73-2.07) | 0.4355 |  |  | 0.96 (0.82-1.12) | 0.5757 |  | 0.86 (0.51-1.47) | 0.5909 |  |
| Model 3 | 1.03 (0.84-1.26) | 0.7980 |  | 1.17 (0.70-1.97) | 0.5499 |  |  | 0.95 (0.82-1.11) | 0.5513 |  | 0.89 (0.52-1.51) | 0.6641 |  |
| **CKD** |  |  |  |  |  | 0.2339 |  |  |  |  |  |  | 0.4752 |
| Model 1 | 0.94 (0.74-1.19) | 0.5955 |  | 1.34 (0.83-2.17) | 0.2270 |  |  | 1.00 (0.84-1.19) | 0.9884 |  | 0.87 (0.55-1.38) | 0.5477 |  |
| Model 2 | 0.97 (0.76-1.23) | 0.7982 |  | 1.34 (0.83-2.17) | 0.2284 |  |  | 0.98 (0.82-1.16) | 0.8110 |  | 0.88 (0.55-1.39) | 0.5780 |  |
| Model 3 | 0.94 (0.74-1.20) | 0.6448 |  | 1.27 (0.78-2.05) | 0.3359 |  |  | 0.97 (0.82-1.16) | 0.7574 |  | 0.85 (0.54-1.36) | 0.4991 |  |
| **T2D** |  |  |  |  |  | 0.0008 |  |  |  |  |  |  | 0.0040 |
| Model 1 | 0.88 (0.75-1.02) | 0.0902 |  | 1.39 (1.06-1.82) | 0.0173 |  |  | 0.97 (0.89-1.05) | 0.4119 |  | 0.78 (0.61-1.01) | 0.0597 |  |
| Model 2 | 0.93 (0.80-1.08) | 0.3625 |  | 1.62 (1.24-2.13) | 0.0005 |  |  | 0.97 (0.89-1.06) | 0.4679 |  | 0.84 (0.65-1.08) | 0.1713 |  |
| Model 3 | 0.92 (0.79-1.07) | 0.2971 |  | 1.59 (1.21-2.09) | 0.0010 |  |  | 0.96 (0.88-1.04) | 0.3241 |  | 0.84 (0.65-1.08) | 0.1686 |  |
| **Hypertension** |  |  |  |  |  | < 0.0001 |  |  |  |  |  |  | < 0.0001 |
| Model 1 | 0.92 (0.81-1.04) | 0.1782 |  | 1.01 (0.78-1.30) | 0.9317 |  |  | 0.98 (0.91-1.05) | 0.5132 |  | 1.02 (0.87-1.20) | 0.7868 |  |
| Model 2 | 0.93 (0.82-1.06) | 0.2898 |  | 1.07 (0.83-1.37) | 0.6227 |  |  | 0.98 (0.91-1.05) | 0.5581 |  | 1.05 (0.89-1.23) | 0.5685 |  |
| Model 3 | 0.94 (0.83-1.06) | 0.3164 |  | 1.07 (0.83-1.39) | 0.5775 |  |  | 0.97 (0.91-1.05) | 0.4684 |  | 1.06 (0.91-1.25) | 0.4520 |  |
| **Dorsalgia** |  |  |  |  |  | < 0.0001 |  |  |  |  |  |  | < 0.0001 |
| Model 1 | 0.91 (0.80-1.04) | 0.1606 |  | 0.75 (0.59-0.95) | 0.0192 |  |  | 0.89 (0.82-0.98) | 0.0137 |  | 0.93 (0.74-1.16) | 0.4973 |  |
| Model 2 | 0.92 (0.81-1.04) | 0.1849 |  | 0.76 (0.59-0.96) | 0.0216 |  |  | 0.90 (0.82-0.98) | 0.0213 |  | 0.93 (0.74-1.16) | 0.5008 |  |
| Model 3 | 0.91 (0.80-1.04) | 0.1603 |  | 0.75 (0.59-0.95) | 0.0177 |  |  | 0.89 (0.82-0.98) | 0.0141 |  | 0.92 (0.73-1.15) | 0.4571 |  |
| **Injury & poisoning** |  |  |  |  |  | 0.1906 |  |  |  |  |  |  | 0.4627 |
| Model 1 | 0.98 (0.90-1.06) | 0.5958 |  | 0.93 (0.8-1.08) | 0.3582 |  |  | 0.90 (0.86-0.95) | 0.0001 |  | 1.13 (0.98-1.29) | 0.0841 |  |
| Model 2 | 0.97 (0.90-1.06) | 0.5281 |  | 0.94 (0.81-1.09) | 0.4246 |  |  | 0.91 (0.86-0.95) | 0.0001 |  | 1.13 (0.99-1.29) | 0.0812 |  |
| Model 3 | 0.98 (0.90-1.06) | 0.5804 |  | 0.94 (0.81-1.10) | 0.4282 |  |  | 0.90 (0.86-0.95) | <0.0001 |  | 1.13 (0.99-1.30) | 0.0679 |  |

**Abbreviations:** CVD, cardiovascular disease; IH, intracerebral hemorrhage; IHD, ischemic heart disease; IS, ischemic stroke; COPD, chronic obstructive pulmonary disease; CKD, chronic kidney disease; T2D, type 2 diabetes.

**Model 1:** adjusted for age, study area, highest education, household income, and household size.

**Model 2:** adjusted for covariates in model 1, alcohol drinking, smoking, dietary habits, physical activity, and BMI.

**Model 3:** adjusted for covariates in model 2, history of diabetes, hypertension, respiratory disease, CVD, or cancer at baseline, self-reported satisfaction level of life, and family history of the analyzed disease (adjusted for only in corresponding analysis), self-reported satisfaction level of life, and menopausal status (for women only).

* *P* values for interaction in model 3.

**Table S14 Death records of patients during the follow-up**

| **Patients** | **Men** | | |  | **Women** | | |
| --- | --- | --- | --- | --- | --- | --- | --- |
|  | **Patients at baseline** | **Death** **cases during the follow-up** | **Death /1,000 person-year** |  | **Patients at baseline** | **Death cases during the follow-up** | **Death /1,000 person-year** |
| **Mental and behavioural disorders** |  |  |  |  |  |  |  |
| Psychosocial disorder | 551 | 98 | 17.3 |  | 1,355 | 149 | 10.1 |
| Neurasthenia | 1,542 | 197 | 11.8 |  | 4,157 | 273 | 5.9 |
| **CVD** |  |  |  |  |  |  |  |
| Coronary heart disease | 5,717 | 1,830 | 33.1 |  | 9,755 | 1,692 | 16.5 |
| Stroke | 4,911 | 2,031 | 46.3 |  | 3,973 | 1,107 | 28.5 |
| Rheumatic heart disease | 3,048 | 609 | 18.9 |  | 7,576 | 737 | 8.8 |
| **Cancer** |  |  |  |  |  |  |  |
| Lung | 98 | 51 | 68 |  | 31 | 15 | 59.6 |
| Esophagus | 197 | 100 | 66.1 |  | 99 | 27 | 28.3 |
| Stomach | 185 | 76 | 48.8 |  | 79 | 27 | 37.2 |
| Liver | 29 | 15 | 68.3 |  | 8 | 3 | 42.9 |
| Intestine | 160 | 58 | 41.1 |  | 151 | 40 | 27.5 |
| Breast | NA | NA | NA |  | 567 | 123 | 21.8 |
| Prostate | 5 | 1 | 17.8 |  | NA | NA | NA |
| Cervix | NA | NA | NA |  | 353 | 65 | 17.7 |
| Others | 292 | 122 | 48.7 |  | 322 | 63 | 19.4 |
| **Diseases of the respiratory system** |  |  |  |  |  |  |  |
| Pulmonary tuberculosis | 4,236 | 964 | 22.6 |  | 3,423 | 443 | 12.1 |
| Asthma | 1,214 | 341 | 28.1 |  | 1,592 | 197 | 11.4 |
| Chronic bronchitis/emphysema | 6,542 | 2,077 | 32.8 |  | 6,746 | 1,150 | 16.1 |
| **Diseases of the digestive system** |  |  |  |  |  |  |  |
| Chronic hepatitis/cirrhosis | 3,641 | 679 | 18.2 |  | 2,552 | 230 | 8.2 |
| Peptic ulcer | 11,189 | 1,511 | 12.7 |  | 8,826 | 630 | 6.5 |
| Cholelithiasis/cholecystitis | 8,168 | 1,170 | 13.6 |  | 22,829 | 2,062 | 8.3 |
| **Diseases of the genitourinary system** |  |  |  |  |  |  |  |
| Chronic kidney disease | 2,663 | 409 | 14.9 |  | 4,912 | 441 | 8.3 |
| **Other important diseases** |  |  |  |  |  |  |  |
| Diabetes | 11,683 | 3,020 | 26.3 |  | 18,617 | 3,566 | 18.6 |
| Hypertension | 78,832 | 15,868 | 19.6 |  | 101,756 | 12,717 | 11.6 |
| Fracture | 18,508 | 2,088 | 10.5 |  | 16,936 | 1,313 | 7.1 |
| Head injury | 3,496 | 413 | 11 |  | 2,157 | 138 | 5.8 |
| Arthritis | 3,048 | 609 | 18.9 |  | 7,576 | 737 | 8.8 |
| **Patients with any kind of disease*** | 123,329 | 22,167 | 17.3 |  | 159,481 | 16,999 | 9.8 |

***** “Any kind of disease” refers to the following diseases collected at baseline in CKB: diabetes, coronary heart disease, stroke, hypertension, rheumatic heart disease, pulmonary tuberculosis, chronic bronchitis/emphysema/pulmonary heart disease, asthma, chronic hepatitis/cirrhosis, peptic ulcer, cholelithiasis/cholecystitis, chronic kidney disease, fracture, arthritis, psychosocial disorder, neurasthenia, brain trauma, and malignant tumor (lung, esophagus, stomach, liver, intestine, breast, prostate, cervix, and others).

Lung, liver, and prostate cancer were excluded from the disease-specific mortality risk analyses based on the statistical power consideration. If the sample size of the reference group in the Log-rank test was 154, no less than eight samples were required in the other group to ensure statistical power of 0·8 to find an HR of 3·0 (PASS 2021 v21.0.5).

**Table S15 Sex-stratified sensitivity analyses for associations of marital status and mortality risks of patients after excluding those who followed for no more than two years (lived without vs. with a spouse)**

| **Diseases at baseline** | **Men** | |  | **Women** | |
| --- | --- | --- | --- | --- | --- |
|  | **HR (95%CI)** | ***P*** |  | **HR (95%CI)** | ***P*** |
| **Mental and behavioural disorders** |  |  |  |  |  |
| Psychosocial disorder | 0.50 (0.21-1.22) | 0.1294 |  | 0.95 (0.56-1.59) | 0.8375 |
| Neurasthenia | 1.53 (0.94-2.49) | 0.0867 |  | 0.87 (0.62-1.22) | 0.4489 |
| **CVD** |  |  |  |  |  |
| Coronary heart disease | 1.21 (1.03-1.41) | 0.0203 |  | 1.03 (0.91-1.17) | 0.6017 |
| Stroke | 1.31 (1.12-1.52) | 0.0006 |  | 0.89 (0.76-1.05) | 0.1595 |
| Rheumatic heart disease | 1.23 (0.94-1.62) | 0.1346 |  | 1.04 (0.85-1.26) | 0.7216 |
| **Cancer** |  |  |  |  |  |
| Esophagus cancer | 0.65 (0.23-1.83) | 0.4140 |  | 1.45 (0.15-13.99) | 0.7493 |
| Stomach cancer | 7.51 (1.00-56.58) | 0.0502 |  | NA | 0.9942 |
| Intestine cancer | 0.16 (0.00-17.47) | 0.4401 |  | 15.85 (0.55-453.51) | 0.1063 |
| Breast cancer | NA | NA |  | 1.26 (0.62-2.54) | 0.5222 |
| Cervix cancer | NA | NA |  | NA | NA |
| Other cancer | 0.73 (0.22-2.40) | 0.6064 |  | 1.97 (0.59-6.56) | 0.2688 |
| **Diseases of the respiratory system** |  |  |  |  |  |
| Pulmonary tuberculosis | 1.45 (1.19-1.77) | 0.0003 |  | 1.02 (0.79-1.31) | 0.8873 |
| Asthma | 1.61 (1.13-2.30) | 0.0087 |  | 1.15 (0.77-1.71) | 0.4922 |
| Chronic bronchitis/emphysema | 1.31 (1.14-1.50) | 0.0001 |  | 1.00 (0.86-1.16) | 0.9867 |
| **Diseases of the digestive system** |  |  |  |  |  |
| Chronic hepatitis/cirrhosis | 1.58 (1.17-2.13) | 0.0028 |  | 1.05 (0.68-1.61) | 0.8209 |
| Peptic ulcer | 1.22 (1.01-1.47) | 0.0359 |  | 0.99 (0.79-1.22) | 0.8923 |
| Cholelithiasis/cholecystitis | 1.36 (1.10-1.68) | 0.0041 |  | 1.11 (0.99-1.24) | 0.0848 |
| **Diseases of the genitourinary system** |  |  |  |  |  |
| CKD | 0.81 (0.51-1.27) | 0.3546 |  | 0.91 (0.68-1.21) | 0.5076 |
| **Other important diseases** |  |  |  |  |  |
| Diabetes | 1.29 (1.13-1.46) | 0.0001 |  | 1.00 (0.91-1.09) | 0.9548 |
| Hypertension | 1.27 (1.21-1.33) | <0.0001 |  | 1.02 (0.97-1.06) | 0.4351 |
| Fracture | 1.41 (1.22-1.62) | <0.0001 |  | 1.06 (0.93-1.22) | 0.3817 |
| Head injury | 1.22 (0.87-1.72) | 0.2484 |  | 0.93 (0.56-1.57) | 0.7966 |
| Arthritis | 1.22 (0.93-1.61) | 0.1453 |  | 1.04 (0.85-1.26) | 0.7015 |

**Abbreviations:** CVD, cardiovascular disease; CKD, chronic kidney disease.

**Model:** adjusted for age, study area, highest education, household income, household size**,** alcohol drinking, smoking, dietary habits, physical activity, BMI, self-reported satisfaction level of life, and family history of the analyzed disease (adjusted for only in corresponding analysis), self-reported satisfaction level of life, and menopausal status (for women only).

**Table S16 Sex-stratified analyses for associations of four-group marital status (married/cohabitated, widowed, separated/divorced, and unmarried) and mortality risks of patients**

| **Diseases at baseline** | **Men** | |  | **Women** | |
| --- | --- | --- | --- | --- | --- |
|  | **HR (95%CI)** | ***P*** |  | **HR (95%CI)** | ***P*** |
| **Mental and behavioural disorders** |  |  |  |  |  |
| **Psychosocial disorder** |  |  |  |  |  |
| Widowed | 0.59 (0.18-1.90) | 0.3739 |  | 0.94 (0.54-1.65) | 0.8307 |
| Separated/Divorced | 0.33 (0.08-1.33) | 0.1183 |  | 0.42 (0.08-2.09) | 0.2864 |
| Unmarried | 0.38 (0.10-1.55) | 0.1796 |  | 0.81 (0.16-4.17) | 0.8015 |
| **Neurasthenia** |  |  |  |  |  |
| Widowed | 1.50 (0.86-2.61) | 0.1556 |  | 0.88 (0.62-1.23) | 0.4492 |
| Separated/Divorced | 2.00 (0.83-4.79) | 0.1222 |  | NA | 0.9949 |
| Unmarried | 2.84 (0.83-9.68) | 0.0953 |  | 2.59 (0.58-11.55) | 0.2127 |
| **CVD** |  |  |  |  |  |
| **Coronary heart disease** |  |  |  |  |  |
| Widowed | 1.12 (0.95-1.32) | 0.1671 |  | 1.01 (0.90-1.14) | 0.8097 |
| Separated/Divorced | 1.45 (0.95-2.21) | 0.0877 |  | 1.39 (0.80-2.41) | 0.2450 |
| Unmarried | 1.58 (0.99-2.52) | 0.0565 |  | 0.93 (0.29-2.93) | 0.9001 |
| **Stroke** |  |  |  |  |  |
| Widowed | 1.17 (0.99-1.37) | 0.0636 |  | 0.92 (0.79-1.07) | 0.2644 |
| Separated/Divorced | 2.09 (1.48-2.94) | <0.0001 |  | 0.92 (0.39-2.15) | 0.8474 |
| Unmarried | 1.61 (1.06-2.46) | 0.0269 |  | NA | 0.9930 |
| **Rheumatic heart disease** |  |  |  |  |  |
| Widowed | 1.17 (0.88-1.57) | 0.2847 |  | 1.07 (0.89-1.29) | 0.4756 |
| Separated/Divorced | 1.55 (0.73-3.32) | 0.2571 |  | 1.54 (0.76-3.09) | 0.2275 |
| Unmarried | 1.33 (0.70-2.52) | 0.3770 |  | 0.55 (0.07-4.20) | 0.5634 |
| **Cancer** |  |  |  |  |  |
| **Esophagus cancer** |  |  |  |  |  |
| Widowed | 1.15 (0.53-2.49) | 0.7212 |  | 2.25 (0.47-10.82) | 0.3105 |
| Separated/Divorced | NA | NA |  | NA | NA |
| Unmarried | 1.36 (0.12-15.03) | 0.7998 |  | NA | NA |
| **Stomach cancer** |  |  |  |  |  |
| Widowed | 0.54 (0.14-2.16) | 0.3864 |  | NA | NA |
| Separated/Divorced | NA | NA |  | NA | NA |
| Unmarried | NA | NA |  | NA | NA |
| **Intestine cancer** |  |  |  |  |  |
| Widowed | 5.05 (0.29-86.57) | 0.2643 |  | 1.64 (0.29-9.21) | 0.5743 |
| Separated/Divorced | 2.41 (0.03-191.35) | 0.6942 |  | NA | NA |
| Unmarried | NA | NA |  | NA | NA |
| **Breast cancer** |  |  |  |  |  |
| Widowed | NA | NA |  | 1.38 (0.65-2.94) | 0.3978 |
| Separated/Divorced | NA | NA |  | 1.57 (0.39-6.36) | 0.5258 |
| Unmarried | NA | NA |  | NA | NA |
| **Cervix cancer** |  |  |  |  |  |
| Widowed | NA | NA |  | NA | NA |
| Separated/Divorced | NA | NA |  | NA | NA |
| Unmarried | NA | NA |  | NA | NA |
| **Other cancer** |  |  |  |  |  |
| Widowed | 0.78 (0.26-2.41) | 0.6725 |  | 1.71 (0.45-6.46) | 0.4308 |
| Separated/Divorced | 0.83 (0.10-6.64) | 0.8604 |  | 5.30 (0.61-46.24) | 0.1314 |
| Unmarried | 2.42 (0.05-114.01) | 0.6529 |  | NA | 0.9999 |
| **Diseases of the respiratory system** |  |  |  |  |  |
| **Pulmonary tuberculosis** |  |  |  |  |  |
| Widowed | 1.33 (1.08-1.65) | 0.0087 |  | 1.04 (0.82-1.33) | 0.7340 |
| Separated/Divorced | 2.21 (1.42-3.43) | 0.0004 |  | 0.75 (0.29-1.96) | 0.5532 |
| Unmarried | 2.17 (1.34-3.52) | 0.0017 |  | 0.61 (0.08-4.47) | 0.6262 |
| **Asthma** |  |  |  |  |  |
| Widowed | 1.26 (0.85-1.87) | 0.2523 |  | 1.15 (0.78-1.69) | 0.4930 |
| Separated/Divorced | 3.08 (1.45-6.53) | 0.0034 |  | 1.44 (0.28-7.45) | 0.6627 |
| Unmarried | 1.62 (0.70-3.79) | 0.2622 |  | NA | 0.9986 |
| **Chronic bronchitis/emphysema** |  |  |  |  |  |
| Widowed | 1.30 (1.13-1.49) | 0.0003 |  | 1.04 (0.90-1.19) | 0.6304 |
| Separated/Divorced | 1.54 (1.03-2.30) | 0.0335 |  | 0.37 (0.12-1.19) | 0.0952 |
| Unmarried | 1.70 (1.26-2.29) | 0.0005 |  | NA | 0.9932 |
| **Diseases of the digestive system** |  |  |  |  |  |
| **Chronic hepatitis/cirrhosis** |  |  |  |  |  |
| Widowed | 1.29 (0.92-1.82) | 0.1449 |  | 1.19 (0.79-1.78) | 0.4039 |
| Separated/Divorced | 1.81 (1.00-3.28) | 0.0497 |  | 0.43 (0.06-3.33) | 0.4216 |
| Unmarried | 1.49 (0.80-2.79) | 0.2086 |  | NA | 0.9987 |
| **Peptic ulcer** |  |  |  |  |  |
| Widowed | 1.21 (1.00-1.47) | 0.0541 |  | 1.04 (0.84-1.28) | 0.7242 |
| Separated/Divorced | 1.58 (1.02-2.44) | 0.0390 |  | 0.57 (0.18-1.83) | 0.3458 |
| Unmarried | 1.38 (0.76-2.49) | 0.2904 |  | 0.69 (0.09-5.05) | 0.7144 |
| **Cholelithiasis/cholecystitis** |  |  |  |  |  |
| Widowed | 1.18 (0.94-1.49) | 0.1576 |  | 1.11 (0.99-1.23) | 0.0733 |
| Separated/Divorced | 1.60 (1.00-2.57) | 0.0524 |  | 1.43 (0.92-2.24) | 0.1141 |
| Unmarried | 1.62 (0.94-2.79) | 0.0843 |  | 1.30 (0.48-3.54) | 0.6124 |
| **Diseases of the genitourinary system** |  |  |  |  |  |
| **Chronic kidney disease** |  |  |  |  |  |
| Widowed | 0.87 (0.53-1.43) | 0.5849 |  | 0.91 (0.69-1.19) | 0.4870 |
| Separated/Divorced | 0.66 (0.26-1.69) | 0.3840 |  | 0.74 (0.27-2.07) | 0.5704 |
| Unmarried | 0.64 (0.19-2.13) | 0.4709 |  | 1.19 (0.16-8.99) | 0.8670 |
| **Other important diseases** |  |  |  |  |  |
| **Diabetes** |  |  |  |  |  |
| Widowed | 1.14 (1.00-1.31) | 0.0546 |  | 1.00 (0.92-1.08) | 0.9370 |
| Separated/Divorced | 2.10 (1.64-2.69) | <0.0001 |  | 1.01 (0.63-1.61) | 0.9710 |
| Unmarried | 1.43 (1.00-2.05) | 0.0526 |  | 0.41 (0.10-1.64) | 0.2065 |
| **Hypertension** |  |  |  |  |  |
| Widowed | 1.16 (1.10-1.22) | <0.0001 |  | 1.02 (0.98-1.07) | 0.2763 |
| Separated/Divorced | 1.73 (1.53-1.95) | <0.0001 |  | 0.92 (0.70-1.21) | 0.5566 |
| Unmarried | 1.70 (1.52-1.90) | <0.0001 |  | 1.36 (0.83-2.22) | 0.2273 |
| **Fracture** |  |  |  |  |  |
| Widowed | 1.13 (0.95-1.33) | 0.1583 |  | 1.08 (0.95-1.24) | 0.2466 |
| Separated/Divorced | 1.92 (1.44-2.55) | <0.0001 |  | 0.89 (0.43-1.82) | 0.7407 |
| Unmarried | 2.14 (1.57-2.92) | <0.0001 |  | 1.82 (0.57-5.86) | 0.3125 |
| **Head injury** |  |  |  |  |  |
| Widowed | 0.89 (0.59-1.35) | 0.5900 |  | 0.82 (0.49-1.39) | 0.4683 |
| Separated/Divorced | 2.37 (1.35-4.14) | 0.0026 |  | 1.12 (0.32-3.91) | 0.8622 |
| Unmarried | 1.43 (0.62-3.31) | 0.3980 |  | NA | 0.9968 |
| **Arthritis** |  |  |  |  |  |
| Widowed | 1.17 (0.87-1.56) | 0.3016 |  | 1.07 (0.89-1.30) | 0.4644 |
| Separated/Divorced | 1.55 (0.72-3.31) | 0.2609 |  | 1.53 (0.76-3.07) | 0.2333 |
| Unmarried | 1.34 (0.71-2.53) | 0.3698 |  | 0.55 (0.07-4.21) | 0.5652 |

**Abbreviations:** CVD, cardiovascular disease; CKD, chronic kidney disease.

**Model:** adjusted for age, study area, highest education, household income, household size**,** alcohol drinking, smoking, dietary habits, physical activity, BMI, self-reported satisfaction level of life, and family history of the analyzed disease (adjusted for only in corresponding analysis), self-reported satisfaction level of life, and menopausal status (for women only).

**Table S17 Adjusted hazard ratios (aHRs) of marital status (lived without vs. with a spouse) for mortality risks of patients stratified by sex and birth cohort**

|  | **Men** | | | | | |  | **Women** | | | | | |
| --- | --- | --- | --- | --- | --- | --- | --- | --- | --- | --- | --- | --- | --- |
| **Disease categories** | **< 1955** | |  | **>=1955** | | ***P* for interaction*** |  | **< 1955** | |  | **>=1955** | | ***P* for interaction*** |
|  | **HR (95% CI)** | ***P*** |  | **HR (95% CI)** | ***P*** |  |  | **HR (95% CI)** | ***P*** |  | **HR (95% CI)** | ***P*** |  |
| **Mental and behavioural disorders** |  |  |  |  |  |  |  |  |  |  |  |  |  |
| Psychosocial disorder | 0.80 (0.25-2.57) | 0.7041 |  | 0.54 (0.04-7.56) | 0.6508 | 0.0860 |  | 0.77 (0.43-1.39) | 0.3817 |  | 1.10 (0.25-4.74) | 0.9016 | 0.4874 |
| Neurasthenia | 1.67 (1.03-2.71) | 0.0369 |  | 3.68 (0.22-61.45) | 0.3640 | 0.0129 |  | 0.84 (0.60-1.18) | 0.3202 |  | 0.07 (0.01-1.02) | 0.0515 | 0.3208 |
| **CVD** |  |  |  |  |  |  |  |  |  |  |  |  |  |
| Coronary heart disease | 1.18 (1.01-1.37) | 0.0327 |  | 2.09 (0.63-6.91) | 0.2295 | 0.6363 |  | 1.03 (0.91-1.16) | 0.6527 |  | 1.76 (0.70-4.44) | 0.2301 | 0.0519 |
| Stroke | 1.24 (1.07-1.44) | 0.0048 |  | NA | NA | 0.0007 |  | 0.92 (0.79-1.06) | 0.2532 |  | 0.70 (0.22-2.22) | 0.5414 | 0.9876 |
| Rheumatic heart disease | 1.22 (0.94-1.59) | 0.1416 |  | 1.79 (0.52-6.25) | 0.3582 | 0.1678 |  | 1.03 (0.86-1.25) | 0.7239 |  | 1.81 (0.65-5.05) | 0.2590 | 0.0089 |
| **Cancer** |  |  |  |  |  |  |  |  |  |  |  |  |  |
| Esophagus cancer | 1.10 (0.51-2.36) | 0.8123 |  | NA | NA | 0.9980 |  | 2.68 (0.58-12.28) | 0.2044 |  | NA | NA | NA |
| Stomach cancer | 0.51 (0.13-2.07) | 0.3485 |  | NA | 0.9741 | 0.5021 |  | NA | NA |  | NA | NA | NA |
| Intestine cancer | NA | 0.4616 |  | NA | 0.8924 | NA |  | 1.64 (0.28-9.46) | 0.5828 |  | NA | NA | NA |
| Breast cancer | NA | NA |  | NA | NA | NA |  | 1.16 (0.52-2.61) | 0.7171 |  | NA | 0.9995 | 0.0671 |
| Cervix cancer | NA | NA |  | NA | NA | NA |  | NA | NA |  | NA | NA | NA |
| Other cancer | 0.94 (0.33-2.71) | 0.9115 |  | NA | 0.9978 | 0.2200 |  | 1.15 (0.29-4.50) | 0.8385 |  | NA | 0.9982 | 0.3403 |
| **Diseases of the respiratory system** |  |  |  |  |  |  |  |  |  |  |  |  |  |
| Pulmonary tuberculosis | 1.41 (1.16-1.71) | 0.0006 |  | 1.83 (0.79-4.25) | 0.1609 | 0.0147 |  | 1.03 (0.81-1.31) | 0.8043 |  | 1.51 (0.36-6.34) | 0.5752 | 0.7498 |
| Asthma | 1.31 (0.91-1.87) | 0.1432 |  | 3.58 (0.55-23.35) | 0.1820 | 0.0158 |  | 1.13 (0.77-1.68) | 0.5305 |  | NA | 0.9981 | 0.0490 |
| Chronic bronchitis/emphysema | 1.33 (1.17-1.51) | <0.0001 |  | 3.68 (1.45-9.32) | 0.0060 | 0.0106 |  | 1.01 (0.87-1.16) | 0.9096 |  | 1.5 (0.40-5.65) | 0.5477 | 0.8405 |
| **Diseases of the digestive system** |  |  |  |  |  |  |  |  |  |  |  |  |  |
| Chronic hepatitis/cirrhosis | 1.58 (1.16-2.17) | 0.0041 |  | 1.38 (0.69-2.77) | 0.3640 | 0.8026 |  | 1.06 (0.69-1.63) | 0.7797 |  | 2.17 (0.62-7.53) | 0.2229 | 0.1448 |
| Peptic ulcer | 1.20 (1.00-1.44) | 0.0446 |  | 1.76 (0.91-3.42) | 0.0953 | 0.2210 |  | 0.99 (0.80-1.23) | 0.9589 |  | 1.64 (0.56-4.78) | 0.3674 | 0.7068 |
| Cholelithiasis/cholecystitis | 1.29 (1.05-1.59) | 0.0155 |  | 0.89 (0.35-2.26) | 0.8104 | 0.9741 |  | 1.10 (0.98-1.22) | 0.1049 |  | 1.83 (1.08-3.08) | 0.0234 | 0.0156 |
| **Diseases of the genitourinary system** |  |  |  |  |  |  |  |  |  |  |  |  |  |
| CKD | 0.79 (0.51-1.24) | 0.3129 |  | 1.05 (0.21-5.34) | 0.9523 | 0.4948 |  | 0.87 (0.65-1.15) | 0.3241 |  | 1.31 (0.51-3.36) | 0.5771 | 0.0333 |
| **Other important diseases** |  |  |  |  |  |  |  |  |  |  |  |  |  |
| Diabetes | 1.26 (1.11-1.43) | 0.0003 |  | 1.78 (1.17-2.72) | 0.0074 | 0.0348 |  | 0.99 (0.91-1.07) | 0.7308 |  | 1.21 (0.69-2.12) | 0.5026 | 0.6988 |
| Hypertension | 1.24 (1.19-1.30) | <0.0001 |  | 1.35 (1.09-1.67) | 0.0060 | <0.0001 |  | 1.02 (0.98-1.07) | 0.3065 |  | 1.16 (0.87-1.56) | 0.3121 | 0.4803 |
| Fracture | 1.29 (1.11-1.50) | 0.0008 |  | 1.37 (0.88-2.13) | 0.1592 | 0.0087 |  | 1.08 (0.94-1.23) | 0.2752 |  | 1.04 (0.45-2.39) | 0.9208 | 0.8314 |
| Head injury | 1.00 (0.70-1.44) | 0.9801 |  | 2.02 (0.82-4.98) | 0.1245 | 0.1257 |  | 0.83 (0.48-1.43) | 0.5091 |  | NA | NA | 0.8335 |
| Arthritis | 1.22 (0.93-1.59) | 0.1510 |  | 1.80 (0.52-6.28) | 0.3557 | 0.1754 |  | 1.04 (0.86-1.25) | 0.7097 |  | 1.78 (0.64-4.97) | 0.2688 | 0.0086 |

**Abbreviations:** CVD, cardiovascular disease; CKD, chronic kidney disease.

**Model:** adjusted for age, study area, highest education, household income, household size**,** alcohol drinking, smoking, dietary habits, physical activity, BMI, self-reported satisfaction level of life, and family history of the analyzed disease (adjusted for only in corresponding analysis), self-reported satisfaction level of life, and menopausal status (for women only).

**Figure S1. Flow chart of study participants in the CKB cohort**


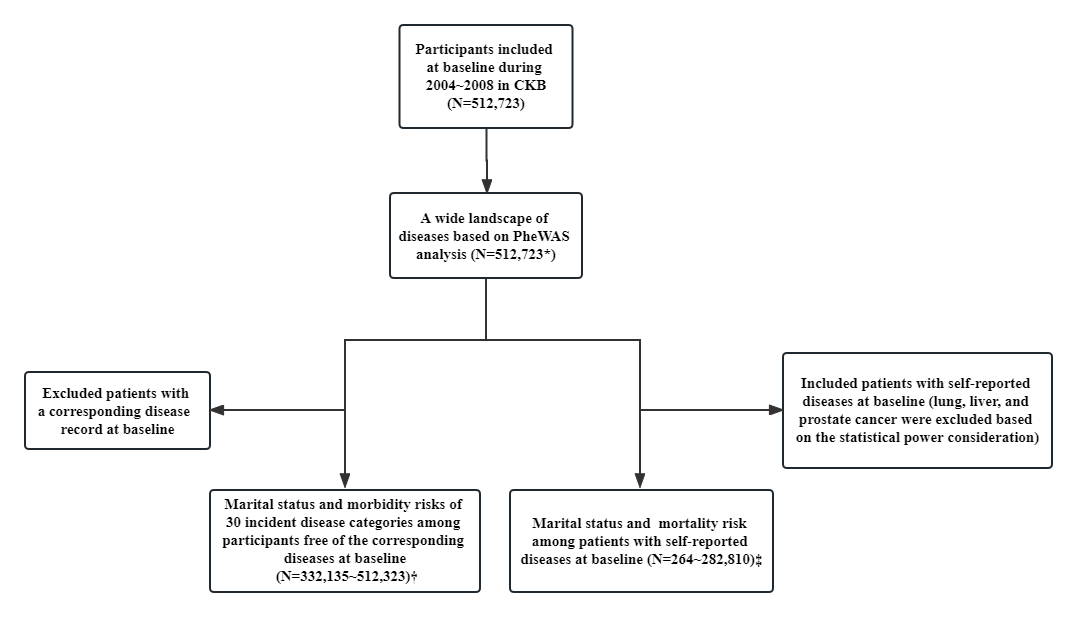


* The total number consisted of 210,202 men and 302,521 women.

† The minimum sample size consisted of 131,370 men and 200,765 women to analyze the morbidity risk of developing hypertension. The maximum sample size consisted of 210,202 men and 302,521 women to analyze the morbidity risk of pneumonia, acute upper respiratory infection, urethritis and urethral syndrome, and dorsalgia (back pain in Phecode).

‡ The minimum sample size consisted of 185 men and 79 women to analyze the mortality risk of patients with stomach cancer at baseline. The maximum sample size consisted of 123,329 men and 159,481 women to analyze the mortality risk of patients with any self-reported diseases at baseline in CKB.

**Figure S2. Volcano plot** **based on PheWAS results of** **marital status in men lived without vs. with a spouse**


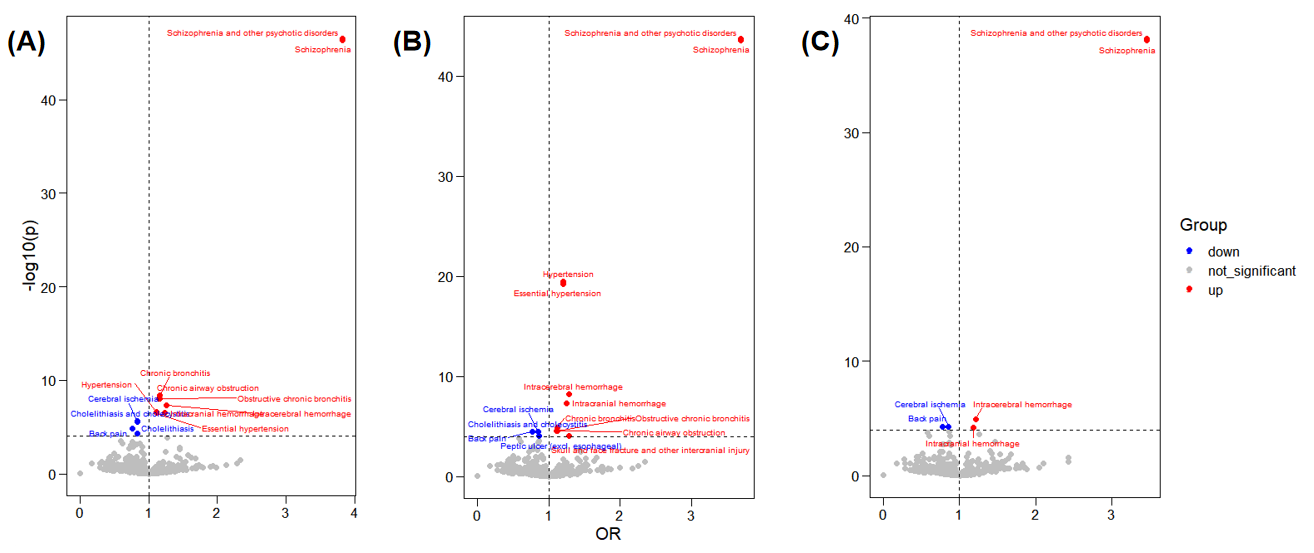


**Panel A:** PheWAS results of model 1, adjusted for age, study area, highest education, household income, and household size.

**Panel B:** PheWAS results of model 2, adjusted for covariates in model 1, alcohol drinking, smoking, dietary habits, physical activity, and BMI.

**Panel C:** PheWAS results of model 3, adjusted for covariates in model 2, history of diabetes, hypertension, respiratory disease, CVD, or cancer at baseline, self-reported satisfaction level of life, and family history of the analyzed disease (adjusted for only in corresponding analysis), and self-reported satisfaction level of life.

**Figure S3. Volcano plot of based on PheWAS results of marital status in women lived without vs. with a spouse**

**
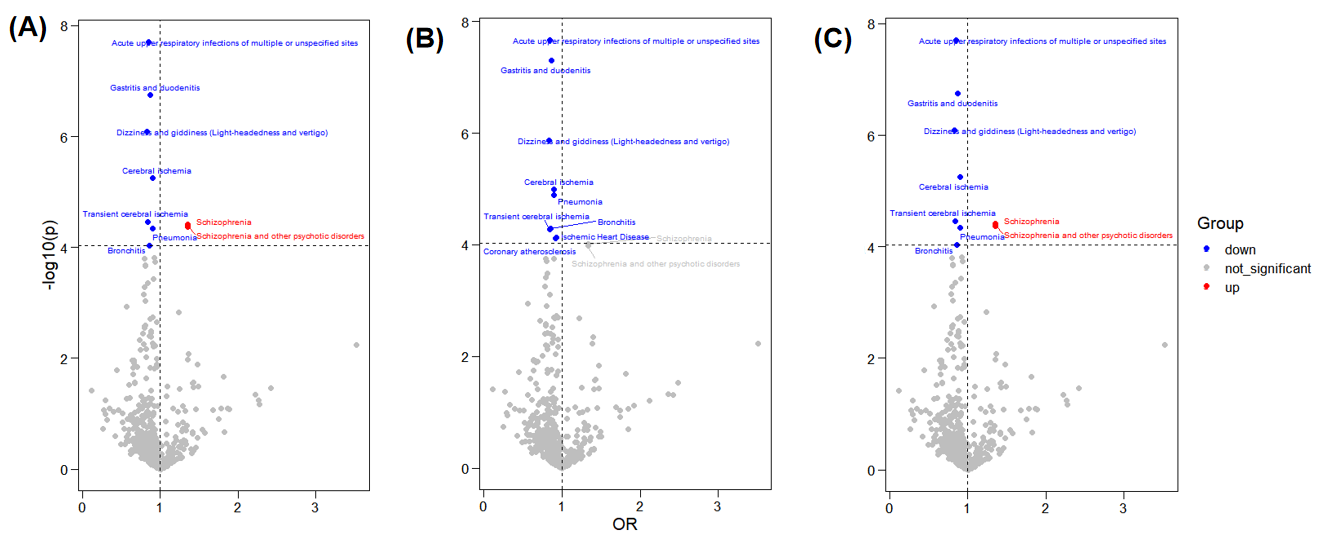
**

**Panel A:** PheWAS results of model 1, adjusted for age, study area, highest education, household income, and household size.

**Panel B:** PheWAS results of model 2, adjusted for covariates in model 1, alcohol drinking, smoking, dietary habits, physical activity, and BMI.

**Panel C:** PheWAS results of model 3, adjusted for covariates in model 2, history of diabetes, hypertension, respiratory disease, CVD, or cancer at baseline, self-reported satisfaction level of life, and family history of the analyzed disease (adjusted for only in corresponding analysis), self-reported satisfaction level of life, and menopausal status.
