# Supplementary material for: A wide landscape of morbidity and mortality risk associated with marital status in 0.5 million Chinese men and women: a prospective cohort study
Source: Lancet Reg Health West Pac. 2023 Nov 7;42:100948. doi: 10.1016/j.lanwpc.2023.100948 (PMC10865043; doi:10.1016/j.lanwpc.2023.100948)
Supplement: Translated Abstract [file mmc2.docx]

*This translation in Chinese was submitted by the authors and we reproduce it as supplied. It has not been peer reviewed. Our editorial processes have only been applied to the original abstract in English, which should serve as reference for this manuscript.*

**摘要**

**背景**：目前缺乏婚姻状况对长期健康影响的全面描述。

**方法**：使用中国慢性病前瞻性队列研究（China Kadoorie Biobank，CKB）的基线（2004-2008年）和随访信息（ICD 10编码事件，截至2017年12月31日），分性别进行婚姻状况（有配偶 vs. 无配偶）的全表型组关联研究（phenome-wide association，PheWAS）分析。我们估计了调整后的风险比（adjusted hazard ratios，aHR），以评估婚姻状况与PheWAS中显著的疾病或与中国2017年性别特异性前十大死因疾病的发病风险之间的关联。此外，我们还在基线患有主要慢性病的参与者中评估了婚姻状况与死亡风险之间的关系。

**结果：**在长达11·1年的中位随访期内，记录了210,202名30-79岁男性和302,521名女性的共1,946,380起健康事件。在全表型组水平上，婚姻状况分别与男性的13种疾病（*p* < 9·92×10^-5^）和女性的9种疾病（*p* < 9·33×10^-5^）具有显著关联。在最终模型中调整了所有疾病特异性协变量后发现，与其他参与者相比，没有配偶的参与者患精神分裂症、分裂型和妄想症的风险增加（男性aHR[95%CI]：2·55，[1·83-3·56]；女性1·49，[1·13-1·97]）。除了上述疾病，在没有配偶的男性中观察到总的精神和行为障碍（1·31，1·31-1·53）、心血管疾病（1·07，1·04-1·10）和癌症（1·06，1·00-1·12）的发生风险增加，而没有配偶的女性泌尿生殖系统疾病（0·89，0·85-0·93）、伤害和中毒（0·93，0·88-0·97）的发生风险较低。在282,810名基线时患有主要慢性病的参与者中，有39,166人死亡。对于男性患者，在21种疾病中发现了有12种疾病对应的无配偶患者死亡率增加，对于女性患者，则在23种疾病中观察到1种疾病对应的无配偶患者死亡率增加。对于基线时有任何自我报告疾病的患者，与有配偶的患者相比，无配偶的男性和女性的死亡率风险aHR（95%CI）分别为1·29（1·24-1·34）和1·04（1·00-1·07）（*p_交互_* < 0·0001）。

**解释**：在中国中老年人中，婚姻状况与发病和死亡风险的长期关联是多样性的，无配偶生活造成的不利影响在男性中更为严重。婚姻状况可能是健康需求的一个影响因素。
